# Synthesis, Crystal Structure, Hirshfeld Surface, Vibrational Thermal Analyses, and NLO Properties of a New Hybrid Material: 2,4-Diamino-6-Phenyl-1,3,5-Triazinium Perchlorate, Supported by DFT Calculations

**DOI:** 10.3390/molecules31193369

**Published:** 2026-09-22

**Authors:** Louiza Ouksel, Rima Kiche, Lotfi Boudjema, Hakima Ait Youcef, Abdeltif Amrane, Thierry Roisnel, Riadh Bourzami

**Affiliations:** 1 Laboratory of Electrochemistry and Materials (LEM), Department of Process Engineering, Faculty of Technology, University Ferhat Abbas Setif-1, Sétif 19000, Algeria; l.ouksel@univ-setif.dz (L.O.); kiche.rima@univ-setif.dz (R.K.); 2 Department of Nature and Life Sciences, Faculty of Biology, University Ferhat Abbas Setif-1, Sétif 19000, Algeria; 3 Mechatronics Laboratory (LMETR)—E1764200, Optics and Precision Mechanics Institute, University Ferhat Abbas Sétif-1, Sétif 19000, Algeria; lotfi.boudjema@gmail.com; 4 Department of Chemistry, University College London, 20 Gordon Street, London WC1H 0AJ, UK; 5 ICAMM Lab—Independent Laboratory for Molecular Modeling and Materials Research, F-13013 Marseille, France; 6 Laboratoire d’Electrochimie, d’Ingénierie Moléculaire et de Catalyse Redox (LEIMCR), Faculté de Technologie, University Ferhat Abbas Sétif-1, Sétif 19000, Algeria; hakimaaityoucef@yahoo.fr; 7 École Nationale Supérieure de Chimie de Rennes, University Rennes, CNRS, ISCR (Institut des Sciences Chimiques de Rennes)—UMR 6226, F-35000 Rennes, France; abdeltif.amrane@univ-rennes.fr; 8 University Rennes, CNRS, ISCR (Institut des Sciences Chimiques de Rennes)—UMR 6226, F-35000 Rennes, France; thierry.roisnel@univ-rennes.fr

**Keywords:** benzoguanamine, organic–inorganic, X-ray crystallography, Hirshfeld surface analysis, supramolecular 3D network, DFT calculations

## Abstract

A novel organic–inorganic ionic crystal 2,4-diamino-6-phenyl-1,3,5-triaziniumperchlorate (C_9_H_10_N_5_^+^·ClO_4_^−^) was synthesized via the reaction of benzoguanamine with perchloric acid. Its structure was affined by single-crystal XRD and characterized by UV-Vis and FT-IR spectroscopies. It crystallizes in a monoclinic system, with *P*2_1_/*c* space group and lattice parameters of *a* = 13.7975 (6), *b* = 5.0711 (2), *c* = 16.6471 (9) Å, and β = 91.869 (2) ° and a cell volume of V = 1164.15 (9) Å^3^. Additionally, the 3D-supramolecular network illustrates a supramolecular layer-by-layer arrangement. The two-dimensional fingerprint plots and Hirshfeld surface analysis showed that H∙∙∙O/O∙∙∙H dominates the interactions, contributing with 33.4%. The UV-Vis spectrum revealed two strong absorption bands at 204 and 247nm, indicating π→π* and n→π* electronic transitions, respectively, which were corroborated by TD-DFT calculations. FT-IR spectroscopy confirmed key vibrational modes consistent with triazinium cations and perchlorate anions, demonstrating strong agreement with the affined structure. TGA/DTG demonstrated enhanced thermal stability until 290 °C compared to benzoguanamine, with a four-stage degradation profile, illustrating robust thermal resilience. Moreover, BSSE-corrected interaction-energy calculations revealed that the charge-assisted cation–anion N-H···O hydrogen bond is the main cohesive force in the crystal, with a strong stabilization energy of −248.8 kJ mol^−1^, while π-associated contacts mainly contribute to layered supramolecular packing. C_9_H_10_N_5_^+^·ClO_4_^−^ exhibits enhanced NLO properties in comparison to urea, due to the cooperative effect of ionic hydrogen bonding and ordered π-stacking.

## 1. Introduction

Organic–inorganic hybrid materials are an advanced class of functional materials combining organic and inorganic components. Their key advantage is the synergistic interaction that leads to enhanced or novel properties. This synergy enables the design of materials with tailored characteristics for diverse industrial applications. Recent research focuses on nanoscale structure–property relationships to better control material performance [1]. Precise engineering at submicrometer levels improves mechanical, optical, and electronic properties [2]. Hybrid perovskites have revolutionized optoelectronics with tunable band gaps and strong photophysical behavior. Hybrid metamaterials exhibit switchable mechanical properties, enabling smart and adaptive systems [3,4,5]. Advanced synthesis methods have improved applications in sensing, catalysis, and energy technologies [6]. Emerging fields include magnetic and quantum hybrids for spintronics and next-generation computing. Challenges such as stability and sustainability are driving the development of greener and lead-free hybrid materials [7].

The ability of hybrids to successfully combine the frequently disparate qualities of organic and inorganic components into a single material is their most evident benefit. As a result, their appeal extends far beyond mechanical strength, encompassing improved thermal and chemical resistance, optical properties, and more [8,9,10,11,12,13,14,15,16,17]. In particular, organic–inorganic hybrid materials incorporating the 1,3,5-triazine ring have shown enhanced or novel properties that make them highly suitable for advanced industrial uses. These materials exhibit thermal stability and are widely used as fire-retardant additives for polymeric materials as well as in the formation of hybrid materials [18,19,20,21].

Moreover, hybrids play a vital role in the synthesis of supramolecular structures and high-molecular architectures. The triazine core functions as both a hydrogen-bond donor and acceptor, facilitate the construction of one-, two-, and three-dimensional supramolecular motifs in combination with various carboxylic acids. Their derivatives are also known to form hydrogen-bonded self-assemblies with well-defined surface structures, which have been effectively used as templates in surface-based supramolecular chemistry [22,23,24,25,26,27,28].

1,3,5-triazines derivatives are widely recognized for their low reactivity and good thermal stability. These compounds are particularly suitable for the formation of organic–inorganic hybrids capable of generating well-defined non-covalent architectures through multiple hydrogen bonds, owing to their complementary arrays of hydrogen-bonding sites. Structurally, triazines are planar, nitrogen-rich compounds, with the ability to form both mono- and diprotonated cationic species. Their use in the design of hydrogen-bonded supramolecular assemblies is well-established [27,28,29].

In this context, we report the synthesis of a hybrid ionic compound based on a 1,3,5-triazine derivative: 2,4-diamino-6-phenyl-1,3,5-triazinium (also known as benzoguanamine). Benzoguanamine is a white crystalline solid known for its excellent chemical and structural stability. It finds extensive use in the production of thermosetting resins, resin modifiers, enamels, thermoplastic resins, and plastic additives. It serves as a stabilizer for water-soluble formaldehyde solutions and as a chemical intermediate in the synthesis of pesticides, pharmaceuticals, and dyes [30].

Due to its superior resistance to detergents, benzoguanamine is also employed in amino resins used for laundry coatings [31]. In industrial paint formulations, it is incorporated into melamine alkyd-based enamels to enhance gloss, flexibility, and alkali resistance. It is particularly useful in the preparation of daylight fluorescent paints, contributing to improved weathering durability. Likewise, it works effectively as a stabilizer in aqueous formaldehyde solutions, preventing the precipitation of formaldehyde polymers [32].

In this paper, and as a continuation of our previous work on hybrid ionic materials [10,11,12,13,28,29], we report here the synthesis, the structure, and the properties of a new organic–inorganic ionic material: a single crystal of 2,4-diamino-6-phenyl-1,3,5-triazinium perchlorate. The presence of a phenyl substituent enhances the aromatic character and extends the π-electron conjugation of the molecule, while increasing its hydrophobicity compared to triazine derivatives substituted with a hydroxy group. The two amino groups act as effective hydrogen-bond donors, promoting the formation of intermolecular N-H···O with the oxygen atoms of the perchlorate anion. Consequently, crystal stacking is primarily stabilized by cation–anion electrostatic interactions and by a network of hydrogen bonds, with additional contributions from weak π-π stacking interactions between neighboring phenyl rings when favorable molecular orientations occur. Due to its relatively small molecular size and the oxidizing nature of the perchlorate anion, this compound exhibits a simpler supramolecular architecture than phosphate-based triazinium salts and may exhibit distinct thermal and physicochemical properties [33,34].

The molecular structure was determined via single-crystal X-ray diffraction (SXRD), allowing us to analyze in detail the resulting three-dimensional supramolecular network. To further investigate the nature of intermolecular interactions, Hirshfeld surface (HS) analysis was conducted, providing both qualitative and quantitative insights. In addition, spectroscopic techniques, such as UV-Visible and FT-IR spectroscopy, were employed to characterize the optical and vibrational behavior of hybrid materials. UV-Vis spectroscopy offered insights into electronic transitions, while FT-IR spectroscopy enabled the identification of functional groups and validation of structural features. These tools, when combined with theoretical calculations, strengthen the understanding of structure–property relationships in such hybrid systems. Finally, the thermal behavior of this hybrid material was investigated by thermogravimetric and differential analysis (TGA/DTG) techniques providing key information on its stability and decomposition profile.

## 2. Experimental Setup

### 2.1. Synthesis and Crystal Growth

The starting compounds were analytical-grade benzoguanamine and perchloric acid, which were used as received, without further purification. A total of 10 mmol of benzoguanamine was dissolved in 30 mL of hot distilled water under continuous stirring. Separately, 30 mmol of perchloric acid was diluted in 15 mL of distilled water, then added dropwise to a hot aqueous solution of benzoguanamine. The resulting mixture was heated under reflux for 6 h, after which it was allowed to cool to room temperature, leading to the formation of a white precipitate. The solid was then filtered, washed several times with ethanol, and air-dried. The single crystals of 2,4-diamino-6-phenyl-1,3,5-triazinium perchlorate (C_9_H_10_N_5_^+^·ClO_4_^−^) (Scheme 1) were grown from the aqueous solution using a slow solvent-evaporation process. The synthesis was performed several times, and no significant differences were observed between the runs.

### 2.2. Single-Crystal X-Ray Diffraction (SXRD)

SXRD was carried out at 150K, utilizing a Bruker D8 Venture photon diffractometer, equipped with a Mo (Kα1) anticathode and a graphite monochromator, giving an incident wavelength of λ = 0.71073 Å. The refinements were performed by the method of full-matrix least squares utilizing SHELXL-2017 software [35]. All observed reflections and data reduction were used to refine the unit cell by means of CrysAlisRed software [36]. Finally, the structures plotted in this paper were created using Mercury software [12]. In addition, the SXRD data were deposited at the Cambridge Crystallographic Data Centre (CCDC), under the deposition number 2412879.

The crystals were growth in water by adopting the slow solvent-evaporation method at room temperature, where well-defined crystals appeared after four days. Crystal growth was reproducible.

### 2.3. Hirschfield Surface Analysis

The 3-dimensional Hirshfeld surfaces mapped using d_norm_, shape index, and curvedness, along with the corresponding 2-dimensional fingerprint plots of the refined crystal structure, were generated using CrystalExplorer-17.5 software [37].

### 2.4. UV-Vis and FT-IR Spectroscopies

The UV-Vis spectrum of C_9_H_10_N_5_^+^·ClO_4_^−^ was measured at room temperature in the range of 200–800 nm in distilled water using a JASCO V680 spectrophotometer. The infrared spectrum was recorded using a FT-IR 4200 JASCO spectrometer, in the wavenumber range of 600–4000 cm^−1^, at room temperature.

### 2.5. Thermal Analysis

The thermal behavior of the title single crystal was studied over the temperature range from room temperature to 1200 °C utilizing simultaneous thermogravimetric analysis and differential thermal analysis (TGA–DTG) techniques. Measurements were performed under a nitrogen atmosphere at a heating rate of 20 °C/min, using Perkin Elmer TGA 4000 apparatus.

### 2.6. Quantum Calculations Details

Gaussian-09 software was utilized for theoretical calculations based on the density functional theory (DFT), considering three Beck exchange parameters correlated to LYP functional (Lee, Yang and Parr) and 6-311G++(d, p) basis sets. The optimized molecular structures were investigated, as well as the vibrational modes, in which no symmetry restrictions were used for calculating the infrared spectra. The output frequencies were corrected by a factor of 0.96. Additionally, global quantum chemical descriptors (GQCDs) were computed from the frontier molecular orbitals energies.

In addition, the intermolecular interaction energies of the four selected dimers (Figure 10) were evaluated using a DFT-based counterpoise approach in ORCA software in order to quantify the main cohesive motifs identified from the crystallographic and Hirshfeld analyses. Single-point calculations were performed at the unrestricted Kohn–Sham wB97X-D3/aug-cc-pVTZ level using NoSym, VeryTight SCF, and SlowConv. Basis-set superposition error (BSSE) was corrected using the Boys–Bernardi counterpoise scheme, by computing the full dimer energy together with the two monomer-in-dimer-basis energies containing ghost functions. The BSSE-corrected interaction energy was obtained according to:

(1) $$\mathrm{BSSE}\;\mathrm{corrected}\;\mathrm{interaction}\;\mathrm{energy}\;=\;E_{\mathrm{AB}}^{\mathrm{AB}}\;-\;(E_{A}^{\mathrm{AB}}+E_{B}^{\mathrm{AB}})$$

$E_{\mathrm{AB}}^{\mathrm{AB}}$ is the total energy of the full dimer AB calculated in the full dimer basis set. $E_{A}^{\mathrm{AB}}$ and $E_{B}^{\mathrm{AB}}$ are the energies of fragments A and B, respectively, computed in the same dimer basis set using ghost orbitals from the other fragment. Thus, Formula (1) gives the interaction energy corrected for basis-set superposition error (BSSE).

According to the sign convention adopted here, a negative BSSE-corrected interaction energy indicates an attractive and stabilizing molecular pair, whereas a positive value indicates that the isolated dimer geometry is energetically unfavorable relative to the separated fragments.

## 3. Results and Discussion

### 3.1. SXRD Study

The single-crystal X-ray analysis of C_9_H_10_N_5_^+^·ClO_4_^−^ indicates that the asymmetric unit was formed by one perchlorate anion (ClO_4_^−^) and one 2,4-diamino-6-phenyl-1,3,5-triazinium cation (C_9_H_10_N_5_^+^), as depicted in Figure 1a. Table 1 summarizes the crystal data and refinement parameters, while Table S1 collects selected geometric features, including bond lengths, bond angles, and torsion angles. All structural parameters fell within the expected ranges, with no observed anomalies [12,13,14,15,16,17,18,19,20,21,22,23,24,25,26,27,28,29,30,31,32,33,34,35,36,37,38,39]. In addition, the material crystallized in a monoclinic system (Figure 1b), with lattice parameters *a* = 13.7975 (6), *b* = 5.0711 (2), *c* = 16.6471 (9) Å, and β = 91.869 (2) ° and *P*2_1_/*c* symmetry group. Furthermore, the SXRD analysis highlighted that the crystal structure is stabilized primarily by weak intermolecular interactions, particularly hydrogen bonds. Considering a maximum donor–acceptor distance of 3.5 Å, twelve intermolecular hydrogen bonds per unit cell were identified, and their geometric parameters are provided in Table S2. This extended hydrogen-bonding network not only stabilizes the crystal structure, but also gives rise to a three-dimensional supramolecular framework, characteristic of type I organic–inorganic hybrid materials, where discrete organic and inorganic components are interconnected via non-covalent interactions.

#### Three-Dimensional Supramolecular Network

Figure 2 presents the 3D supramolecular network of the material under investigation. It shows that the network was formed by supramolecular chains extending along the a axis ([1 0 0] direction) as shown in (Figure 2a). These chains consisted of alternating C_9_H_10_N_5_^+^ cations and ClO_4_^−^ anions, where each anion links adjacent cations through the following hydrogen bonds: C7—H7···O4 and N4—H4N···O4. In addition, the two largest supramolecular ring motifs observed were designated as $R_{4}^{4}(18)$ and $R_{4}^{4}(12)$. Each of these rings is centered and formed around a symmetry center and contributes to the parallel and oppositely oriented arrangement of adjacent chains (Figure 2b).

Perpendicular to this direction, along the b axis ([0 1 0] direction), the crystal structure displayed parallel-displaced π–π stacking interactions (Figure 2c), with a centroid-to-centroid distance of 3.472 Å between benzene rings. Notably, two additional hydrogen bonds, N4—H4O···O1 and N4—H4N···O1, were observed between the stacked molecules. The stacked molecules form two supramolecular organic layers, as shown in the Figure 2d. These layers are interconnected by a network of intermolecular hydrogen bonds involving N5—H5N···N2, N5—H5N···N4, N5—H5N···N4, and N4—H4N···N5, forming a supramolecular layered arrangement with an interlayer angle of 69.86°, in which the anions are located between each two successive supramolecular organic layers (Figure 2d). In conclusion, the synthetized compound can be classified as a type I organic–inorganic hybrid material, characterized by a layer-by-layer self-assembled supramolecular architecture.

For comparison, the hybrid perchlorate salt C_9_H_10_N_5_^+^.ClO_4_^−^ based on 2,6-diaminopyridinium cations was reported to crystallize in the monoclinic space group P2_1_, also exhibiting structural stabilization through N–H···O hydrogen bonds between protonated amine groups and perchlorate oxygen atoms. In both systems, the ClO_4_^−^ anion retains a quasi-regular tetrahedral geometry with a Cl–O bond length consistent with typical literature values, while the C–N and C–C bond lengths within the organic cations remain within the expected ranges for related derivatives [40].

### 3.2. Hirschfield Surface Analysis (HS)

The molecular HS mapped with d_norm_, shape index, and curvedness are shown in Figure 3. The molecular HS of C_9_H_10_N_5_^+^·ClO_4_^−^ was generated using a standard (high) surface resolution, with the 3D d_norm_ surfaces and the intermolecular contacts illustrated in Figure 3a, mapped over a fixed color scale range of −0.42 (red) to +1.08 Å (blue). The shape index is mapped in the color range of −1.0 to +1.0 Å (Figure 3b) and curvedness is in the range of −4.0 to +0.4 Å (Figure 3c). For each of the structures that were computed, the surfaces were demonstrated to be transparent to yellow in color with the same orientation. Using the 3D d_norm_ surface, very close intermolecular interactions were found.

The shape index’s information is compatible with 2D fingerprint plots, and it is particularly sensitive to even the smallest changes in the surface shape. The nearest neighbor coordination environment of a molecule was identified from the color patches on the Hirshfeld surface depending on their closeness to adjacent molecules (Figure 3d).

Quantitative measures (Table 2) such as volume (VH = 285.12 Å^3^), area (SH = 279.39 Å^2^), globularity (G = 0.75), and asphericity (Ω = 0.13) were also computed using the HS. The globularity is reported to be <1, which suggests that the molecular surface is not a sphere [41], and asphericity is a measure of structural anisotropy [42,43].

Additionally, Crystal Explorer offers tools for dividing fingerprint plots into contributions via specific types of interactions. Currently, the user can only highlight interactions according to the type of atom (C, N, O, etc.). The selection of interactions is based on the sort of atoms present in the HS and those outside of it. By using the “include reciprocal contacts” option, it is also possible to include the interactions with the switched atoms on the inside and outside. The interactions (O∙∙∙H contacts) with hydrogen atoms outside of the HS and oxygen atoms inside it were chosen, including reciprocal contacts (H∙∙∙O contacts).

Figure 4 shows that the parts of the fingerprint corresponding to these interactions remained colored while the rest of the plot was grayed out, and simultaneously, the displayed HS coloring was grayed out with the exception of the regions involving the interaction. A percentage contribution of the interaction is reported based on the total area of these regions divided by the entire surface area of the HS.

The 2D fingerprint plots of C_9_H_10_N_5_^+^.ClO_4_^−^ are presented in Figure 4, showing that the principal intermolecular interactions are H∙∙∙O/O∙∙∙H contacts at 33.4% represented by the largest region in the 2D fingerprint plot (Figure 4b) (di = 1.68 Å and de = 1.7 Å), H∙∙∙H contacts at 20.5% represented by a large area (Figure 2c), and H∙∙∙C/C∙∙∙H at 19.2% represented by a large area at the top and small area at the bottom in the right and left sides, respectively; these contacts are marked with red and green ellipses, respectively (Figure 4d). The N∙∙∙H/H∙∙∙N contacts with 11.8% are represented by large areas on the left and the right sides, respectively, and are also marked with a green and red ellipse, respectively (Figure 4e). Additionally, other weak contributions are remarked as N∙∙∙O/O∙∙∙N contacts with 6.1% (Figure 4f), O∙∙∙C/C∙∙∙O with 4.8% (Figure 4g), O∙∙∙O with 1.6% (Figure 4h), C∙∙∙C with 1.0% (Figure 4i), N∙∙∙C/C∙∙∙N with 0.9% (Figure 4j), and N∙∙∙N with 0.8% (Figure 4k). These results clearly demonstrate that H···O and O···H hydrogen-bonding contacts dominated the intermolecular landscape, followed by H···H and H···C/C···H interactions, which also contributed significantly to the overall molecular packing.

### 3.3. Spectroscopy Studies

#### 3.3.1. UV-Visible Spectroscopy

The UV-Visible absorption spectrum of the hybrid crystal 2,4-diamino-6-phenyl-1,3,5-triazinium perchlorate (C_9_H_10_N_5_^+^·ClO_4_^−^) displays two distinct and intense absorption bands at 204 nm and 247 nm, as illustrated in Figure 5. These bands are characteristic of electronic transitions within the molecule’s extended π-conjugated system.

The absorption peak at 204 nm was attributed to π→π* transitions, which involve the excitation of electrons from bonding π orbitals to antibonding π* orbitals. This type of transition typically occurs in molecules with delocalized π-electron systems, such as the fused triazine and phenyl rings present in the cationic moiety. In contrast, the absorption at 247 nm was assigned to n→π* transitions, involving the promotion of nonbonding electrons, primarily from lone pairs on nitrogen atoms into π* antibonding orbitals. These n→π* transitions are more sensitive to environmental effects and often exhibit lower intensity compared to π→π* transitions due to their symmetry-forbidden nature in isolated systems. This behavior is consistent with that reported for materials with a similar structural nature [11,12,29].

While the experimental data showed two main absorption bands, the TD-DFT (Figure 6) simulations predicted a series of singlet–singlet transitions within the range of 208–337 nm, which were in good agreement with the measured spectrum but also revealed some differences worth discussing. Notably, the calculated transitions at 208.01 nm and 212.76 nm closely corresponded to the experimental band at 204 nm, confirming its dominant π→π* and mixed n→π* characters. However, the broader experimental band observed at 247 nm did not match a single distinct transition in the calculated spectrum. Instead, it likely arose from a convolution of multiple calculated transitions located at 266.12 nm, 286.63 nm, 304.22 nm, and 336.99 nm. These discrepancies may be attributed to several factors. Firstly, TD-DFT calculations are known to slightly overestimate excitation wavelengths, particularly for n→π* transitions, due to limitations in the exchange-correlation functionals used. Secondly, solvent effects and potential molecular aggregation in the experimental aqueous medium may induce spectral broadening or subtle wavelength shift phenomena that are only partially captured by the implicit solvation models employed in the simulations. Lastly, the experimental spectra also include vibronic coupling and thermal effects, which are not considered in vertical excitation energy calculations. Despite these differences, the TD-DFT calculations reproduce the overall profile of the experimental spectrum reasonably well, especially in terms of the nature and sequence of the dominant transitions.

A detailed analysis of the theoretical excitations is summarized in Table 3, providing further insight into the electronic structure of the compound. The most intense transition at 208.01 nm was attributed to HOMO→LUMO+2, with a 50.1% contribution, consistent with a strong π→π* character localized over the triazine–phenyl framework. A similar transition was observed at 212.76 nm, corresponding to HOMO-3→LUMO+2, with a 48.7% contribution. Here, the HOMO-3 orbital includes a lone pair density from nitrogen atoms, suggesting a mixed n→π* and π→π* nature. A weaker excitation was predicted at 266.12 nm (HOMO-4→LUMO, 10.9%), likely involving more localized or mixed orbital interactions. In contrast, the transition at 286.63 nm (HOMO-3→LUMO) exhibited a strong 90.8% contribution, reflecting a nearly pure n→π* transition dominated by lone pairs from nitrogen centers. At 304.22 nm, the HOMO-2→LUMO transition contributed 69.4%, indicating a significant π→π* character involving conjugated aromatic orbitals. Finally, the transition at 336.99 nm (HOMO-1→LUMO, 24.7%) showed a more moderate contribution, likely resulting from a shared excitation between overlapping orbitals.

In summary, the UV-Vis absorption behavior of the hybrid crystal 2,4-diamino-6-phenyl-1,3,5-triazinium perchlorate (C_9_H_10_N_5_^+^·ClO_4_^−^) appears to be primarily influenced by a combination of π→π* and n→π* electronic transitions, involving delocalized π systems and lone pairs from heteroatoms. While some differences remain between the experimental and calculated spectra, the TD-DFT results offer a reasonable level of agreement and help to elucidate the likely molecular origins of the main absorption bands. The complementary use of experimental data and theoretical modeling provides useful insight into the electronic structure of the compound and highlights the potential of TD-DFT methods despite their limitations for interpreting the optical properties of complex molecular systems. Overall, these findings suggest that the extended π conjugation within the molecule plays a significant role in shaping its absorption characteristics, which may be of interest in the future development of materials for optoelectronic, photonic, or molecular-sensing applications.

#### 3.3.2. FT-IR Spectroscopy

The FT-IR spectrum of the synthesized hybrid crystal C_9_H_10_N_5_^+^·ClO_4_^−^ (Figure 7) provides key insights into its molecular vibrations and intermolecular interactions. The most significant absorption bands corresponded to characteristic vibrational modes of the organic cation (C_9_H_10_N_5_^+^), the perchlorate anion (ClO_4_^−^), and hydrogen-bonded interactions. The detailed band assignments are summarized in Table 4. For a deeper interpretation, the experimental spectrum was compared with the theoretically calculated vibrational frequencies.

Broad absorption features observed in the range of 3430–3050 cm^−1^ were attributed to N–H and C–H stretching vibrations [10], as well as hydrogen bonding involving NH_2_ groups [11]. These bands are typical of triazine-based systems and indicate strong intermolecular interactions, consistent with the supramolecular hydrogen-bonding network observed in SXRD and Hirshfeld surface analyses. In comparison, the calculated spectrum reproduces these stretching modes but displays sharper and slightly higher-frequency bands. The observed red shift and significant broadening in the experimental spectrum arise from hydrogen-bonding interactions and solid-state effects within the crystal lattice, which are not accounted for in gas-phase harmonic calculations.

The bending vibration of NH_2_ groups appeared prominently at 1669 cm^−1^, while the bands in the region of 1630–1420 cm^−1^ corresponded to the stretching vibrations of the triazine ring, particularly the C=N and C–N bonds [44]. These modes are well-reproduced in the theoretical spectrum, showing good agreement in both positions and relative intensities. Minor frequency deviations (typically within the range of 10–30 cm^−1^) can be attributed to the harmonic approximation employed in the DFT calculations and the absence of intermolecular interactions in the theoretical model.

Additionally, the ClO_4_^−^ anion exhibited a distinct and intense band at 1064 cm^−1^, characteristic of its symmetric stretching mode [13]. The calculated spectrum successfully predicts a strong vibrational mode in this region, confirming the presence of the perchlorate anion. However, slight shifts and subtle band-shape modifications observed experimentally suggest a reduction in the ideal tetrahedral symmetry of ClO_4_^−^ in the solid state, likely due to hydrogen bonding and electrostatic interactions with the organic cation. Such symmetry-lowering effects are typical in hybrid ionic crystals and further support the structural arrangement determined by crystallographic analysis.

Overall, the IR spectroscopic analysis, reinforced by theoretical calculations, confirms the structural integrity and functional group environment of the synthesized compound. The remaining discrepancies are mainly attributed to harmonic approximation limitations and crystal packing effects, particularly hydrogen-bonding interactions in the solid state. This comprehensive vibrational characterization contributes a valuable insight into the compound’s potential in optoelectronic, sensor, or coordination chemistry applications.

### 3.4. Mapping Electrostatic Potential (MEP)

The Figure 8 presents the total electron density surfaces mapped with the electrostatic potential (MEP) of the asymmetric unit of the compound under investigation, which exhibits positive potential indicated by the blue color around the cationic geometric zones, where the highest potential can be observed around the nitrogen atoms, giving a symmetric value of the electrostatic potential: 9.48 × 10^−2^ V. In return, the negative potential indicated by the red color is localized around the anionic moieties, giving the lowest potential value of −9.48 × 10^−2^ V. The dipolar moment was found to be 18.372 Debye. The high values were consistent with the non-homogeneity of the electrostatic potential.

### 3.5. Thermogravimetric Studies

TGA and DTG curves characteristic of the starting material and the synthesized hybrid ionic material are shown in Figure 9a and Figure 9b, respectively.

It can be seen from the figure that the starting material, benzoguanamine, is thermally stable until 200 °C, and its decomposition occurs in one stage accompanied by rapid weight loss that takes place in the temperature range of between 205 °C and 325 °C, with a weight loss value of 100% corresponding to the DTG peak at 324 °C. The TGA curve of the starting material illustrates that the benzoguanamine is thermally stable until 200 °C, followed by complete decomposition centered at 324 °C, as shown by the non-asymmetric DTG curve. Benzoguanamine is characterized by multi-stage decomposition occurring in a narrow temperature range, presenting the absence of intermediate stable byproducts. This result aligns with reports on nitrogen-rich triazine compounds, which often show rapid, single-stage degradation via amino and ring fragmentation [12].

The TGA curve of the hybrid material showed enhanced thermal stability up to 290 °C, similar to benzoguanamine. There was no mass loss observed around 100 °C, which indicated the absence of water in this hybrid [45]. A more gradual decomposition can be seen, occurring in four stages. The first, the second, and the third stages took place in the temperature from 290 to 640 °C with a significant weight-loss of 68.42%, accompanied by DTG peaks at 327, 362, and 553 °C, which are attributed primarily to the progressive degradation of the organic cation. These peaks can be interpreted as stepwise processes: the initial peak (327 °C) corresponds to deamination with possible NH_3_ release [46]; the second peak (362 °C) reflects the fragmentation of the triazine ring [47]; and the third peak (553 °C) can be assigned to the further breakdown and carbonization of the remaining aromatic structure, leading to the formation of carbonaceous residues [48]. The broad temperature range and multiple DTG maxima suggest a complex degradation pathway influenced by strong hydrogen bonding and electrostatic interactions within the crystal lattice.

The last decomposition stage that started at 640 °C and is associated with a DTG peak at 771 °C can be assigned to the gradual breakdown of the perchlorate anion. As a strong oxidizing agent, ClO_4_^−^ decomposes by releasing oxygen and reducing to lower chlorine oxide species, ultimately forming chloride-containing residues. This high-temperature step further confirms the stabilizing effect of ionic interactions in the hybrid crystal.

Additionally, T50% was 492 °C for the ionic material; while T50% was 302 °C for the starting compound.

The produced triazinium perchlorate hybrid shows remarkable thermal stability when compared to other triazine-based materials documented in the literature. The inherent thermal endurance of the triazine core is demonstrated by 1,3,5-triazine derivatives such as TCZT and TIDT, which exhibit 5% weight-loss temperatures in the range of around 370–384 °C [49].

### 3.6. Quantitative Intermolecular-Interaction Energy Analysis

To complement the crystallographic and Hirshfeld surface analyses, four dominant intermolecular motifs were selected as representative of supramolecular organization: (i) the cation–anion perchlorate O---H-N hydrogen-bonded pair (Figure 10A), (ii) a parallel face-to-face π---π interaction between carbon-based aromatic rings (Figure 10B), (iii) face-to-face π---π contact involving the N-C-N-C heteroaromatic ring (Figure 10C), and (iv) a perpendicular π-contact between carbon-based aromatic rings (Figure 10D). The corresponding characteristic intermolecular distances are 2.32 Å, 3.60 Å, 3.76 Å, and 3.93 Å. This selection is fully consistent with the experimental structure, which reveals a layered assembly sustained by strong hydrogen bonding and aromatic packing. In particular, the Hirshfeld surface analysis shows that H---O/O---H contacts dominate the intermolecular landscape (33.4%), while the SXRD study identifies parallel-displaced aromatic stacking with a centroid-to-centroid distance of 3.472 Å, confirming the structural relevance of both ionic and π-associated contacts.

The most relevant intermolecular distances are indicated for each dimer. The corresponding BSSE-corrected interaction energies are summarized in Table 5.

It is important to distinguish between the geometrical occurrence of an intermolecular contact in the crystal and its pairwise energetic contribution. Hirshfeld surface percentages quantify the relative occurrence of different contact types over the molecular surface, whereas the interaction energies calculated for extracted dimers determine whether a selected molecular pair is attractive or repulsive when considered independently. Similarly, a short intermolecular or centroid-to-centroid distance confirms the structural occurrence of a contact but does not necessarily demonstrate that the corresponding isolated dimer is energetically stabilizing. The counterpoise-corrected calculations were therefore used to establish the energetic character of each representative crystallographic pair.

The BSSE-corrected interaction energies reveal a clearly unbalanced energetic hierarchy. The cation–anion O···H–N pair exhibits a strongly negative interaction energy of −248.8 kJ mol^−1^, demonstrating that this motif is highly stabilizing. This value reflects not only the N–H···O hydrogen bond, but also the strong electrostatic attraction between the positively charged triazinium cation and the perchlorate anion, together with induction, polarization, and dispersion contributions. Accordingly, this interaction is more accurately described as a charge-assisted cation–anion hydrogen-bonded motif. Its dominant energetic role is consistent with the short H···O contact and with the Hirshfeld surface analysis, in which H···O/O···H contacts account for the largest contribution, namely 33.4% of the molecular surface. In contrast, the three selected π-associated dimers exhibit positive BSSE-corrected interaction energies of +148.9 kJ mol^−1^ for the parallel carbon-ring arrangement, +118.8 kJ mol^−1^ for the face-to-face contact involving the N–C–N–C heteroaromatic ring, and +165.5 kJ mol^−1^ for the perpendicular carbon-ring arrangement. With the energy convention used in this work, a positive interaction energy indicates that the isolated dimer, frozen in its crystallographic geometry, is energetically less favorable than the two infinitely separated fragments. These molecular pairs must therefore be regarded as repulsive or destabilizing when considered independently and should not be described as attractive or stabilizing π–π interactions. Among the three arrangements, the heteroaromatic face-to-face dimer exhibits the smallest energetic penalty, whereas the perpendicular carbon-ring contact is the most unfavorable. The presence of these energetically unfavorable isolated pairs in the experimental crystal structure does not imply that the complete lattice is unstable. Crystal packing results from the collective balance of many simultaneous interactions rather than from the energy of a single extracted dimer. In the periodic solid, each ionic unit participates in several charge-assisted N–H···O hydrogen bonds, additional N–H···N and C–H···O contacts, long-range electrostatic interactions, dispersion interactions with multiple neighboring molecules, and steric packing constraints. The strongly favorable cation–anion interaction network can therefore compensate for the energetic penalty associated with individual aromatic-pair geometries. Consequently, the π-associated contacts are interpreted here as geometrically relevant packing motifs that determine the orientation and propagation of the organic layers, rather than as independent sources of lattice stabilization.

Because the three π-associated interaction energies are positive, it would not be physically meaningful to express them as positive percentages of the total attractive stabilization energy. Such a calculation would incorrectly treat repulsive energetic penalties as stabilizing contributions. For descriptive comparison only, the absolute magnitudes of the three positive π-dimer energies correspond to approximately 59.8%, 47.7%, and 66.5% of the magnitude of the cation–anion interaction energy. These percentages do not represent cohesive-energy contributions; they only compare the magnitudes of the repulsive pair energies with the magnitude of the attractive cation–anion interaction. A rigorous separation into electrostatic, exchange–repulsion, induction, and dispersion terms would require a dedicated energy-decomposition analysis and is beyond the scope of the present study.

Overall, the calculations establish that the charge-assisted cation–anion O···H–N motif is the principal energetic anchor of the crystal structure, whereas the π-associated contacts mainly describe the geometrical organization of the layered packing. The present results therefore distinguish clearly between energetic cohesion and structural propagation: the former is dominated by the hydrogen-bonded ionic network, while the latter is influenced by the relative orientation of the aromatic cations within the complete periodic environment.

### 3.7. Nonlinear Optical (NLO) Properties

Molecular materials’ nonlinear optical (NLO) properties have received a lot of attention in recent decades; molecular materials with high NLO effects can be utilized in several optic and optoelectronic applications [50,51,52,53]. The NLO properties were calculated for a single isolated molecule, which is not centrosymmetric, rather than for the crystal structure, which is centrosymmetric.

In this study, some quantum parameters describing the NLO behavior of the isolated molecule were computed using DFT, such as the polarizability (α), polarizability anisotropy (∆α) describing how a molecule’s electronic cloud can be distorted by an external electric field, and the first-order hyper-polarizability (β_tot_) describing the molecule’s first nonlinear order response. These quantum NLO’s parameters are associated with numerous nonlinear optics phenomena [54]. Formulas (2)–(4) provide their definitions, and the obtained values are listed in Table 6:

<α> = 1/3(α_xx_+ α_yy_ + α_zz_) (2)

Δα = 1/2[(α_xx_ − α_yy_)^2^ + (α_xx_ − α_zz_)^2^ + (α_yy_ − α_zz)_^2^]^1/2^ (3)

β_tot_ = [(β_xxx_ + β_xyy_ + β_xzz_)^2^ + (β_yyy_ + β_yxx_ + β_yzz_)^2^ + (β_zzz_ + β_zxx_ + β_zyy_)^2^]^1/2^ (4)

According to the results, C_9_H_10_N_5_^+^·ClO_4_^−^ has the higher linear polarizability and also higher anisotropy polarizability than urea, which is a model molecule employed as a reference for the NLO features of molecular systems, showing that the computed β_tot_ value was roughly 50-times higher than that of urea, confirming nonlinear activity.

Moreover, the strongly polarized supramolecular architecture is particularly significant for C_9_H_10_N_5_^+^·ClO_4_^−^. The presence of a dominant charge-assisted hydrogen-bonding network induces pronounced asymmetry in electron density distribution, which is a key requirement for second-order nonlinear optical (NLO) activity in non-centrosymmetric systems. This behavior is consistent with the measured large-dipole moment (18.372 Debye), indicating strong intrinsic molecular polarity. Such a highly polar value is directly associated with enhanced first-order hyperpolarizability (β), which governs second-harmonic generation and related second-order NLO processes in molecular materials [55,56].

In addition, the ionic hydrogen bond within the 3D network of C_9_H_10_N_5_^+^·ClO_4_^−^ acts as the principal pathway for charge displacement under an external electromagnetic field, thereby reinforcing molecular hyperpolarizability and facilitating cooperative polarization effects. Meanwhile, although the π-associated interactions are not energetically stabilizing on a pairwise basis, they play an essential structural role by enforcing directional packing and maintaining long-range order. Such supramolecular organization is critical because efficient macroscopic NLO responses require not only large β values but also non-centrosymmetric and well-aligned molecular arrangements to avoid the cancellation of dipolar contributions in the bulk crystal [57,58].

Therefore, C_9_H_10_N_5_^+^·ClO_4_^−^ can be understood as a synergistic NLO active system in which the strongly polar hydrogen-bonded ionic network provides the dominant source of nonlinearity, while π-stacking interactions ensure structural coherence and directional alignment. This combination leads to an optimized balance between molecular polarity and crystal packing, both of which are essential for efficient second-order nonlinear optical performance.

## 4. Conclusions

A novel organic–inorganic ionic compound, 2,4-diamino-6-phenyl-1,3,5-triazinium perchlorate (C_9_H_10_N_5_^+^·ClO_4_^−^), was successfully synthesized and structurally characterized. Single-crystal X-ray diffraction revealed that the compound crystallizes in the monoclinic system, space group P2_1_/c, with the lattice parameters a = 13.7975 (6), b = 5.0711 (2), c = 16.6471 (9) Å, and β = 91.869 (2) °. The analysis of crystal packing shows the formation of a three-dimensional supramolecular network, built through layer-by-layer self-assembly mediated by hydrogen bonding. Hirshfeld surface analysis and the corresponding two-dimensional fingerprint plots highlight that H···O/O···H hydrogen bonds were the predominant intermolecular interactions, contributing 33.4% to the total Hirshfeld surface, indicating their critical role in the structural stabilization of the material. These findings confirm the hybrid nature of the compound and underline the importance of non-covalent interactions in shaping its supramolecular architecture. BSSE-corrected interaction-energy calculations further confirmed that O---H-N hydrogen bonding is the dominant cohesive interaction in the crystal structure, while π-associated contacts mainly support layered supramolecular packing. The UV-Vis analysis confirmed the presence of characteristic π→π* and n→π* transitions at 204 and 247 nm, respectively, and showed strong agreement with TD-DFT computational predictions. Furthermore, FT-IR spectroscopy highlighted key vibrational features including NH2 bending, triazine-ring stretching, and perchlorate stretching modes. These spectroscopic findings reinforce the structural results obtained from XRD. Finally, thermal analysis was also investigated using TGA/DTG techniques, illustrating enhanced thermal properties in comparison to the starting benzoguanamine compound, resulting in increased thermal stability up to 290 °C and a more gradual decomposition occurring in four stages. This gradual degradation profile suggests robust thermal resilience, advantageous for high-temperature applications. Collectively, the structural, spectroscopic, and thermal data highlight the material’s potential in optoelectronics, sensing technologies, and hybrid material engineering, driven by its tailored supramolecular architecture and stability. Future research directions and potential applications have been highlighted, including prospects in optoelectronics, sensing, and hybrid functional materials. Further studies may focus on tuning supramolecular interactions to tailor properties and combine experimental and computational approaches to better understand structure–property relationships and charge-transfer mechanisms.

## Figures and Tables

**Scheme 1:**
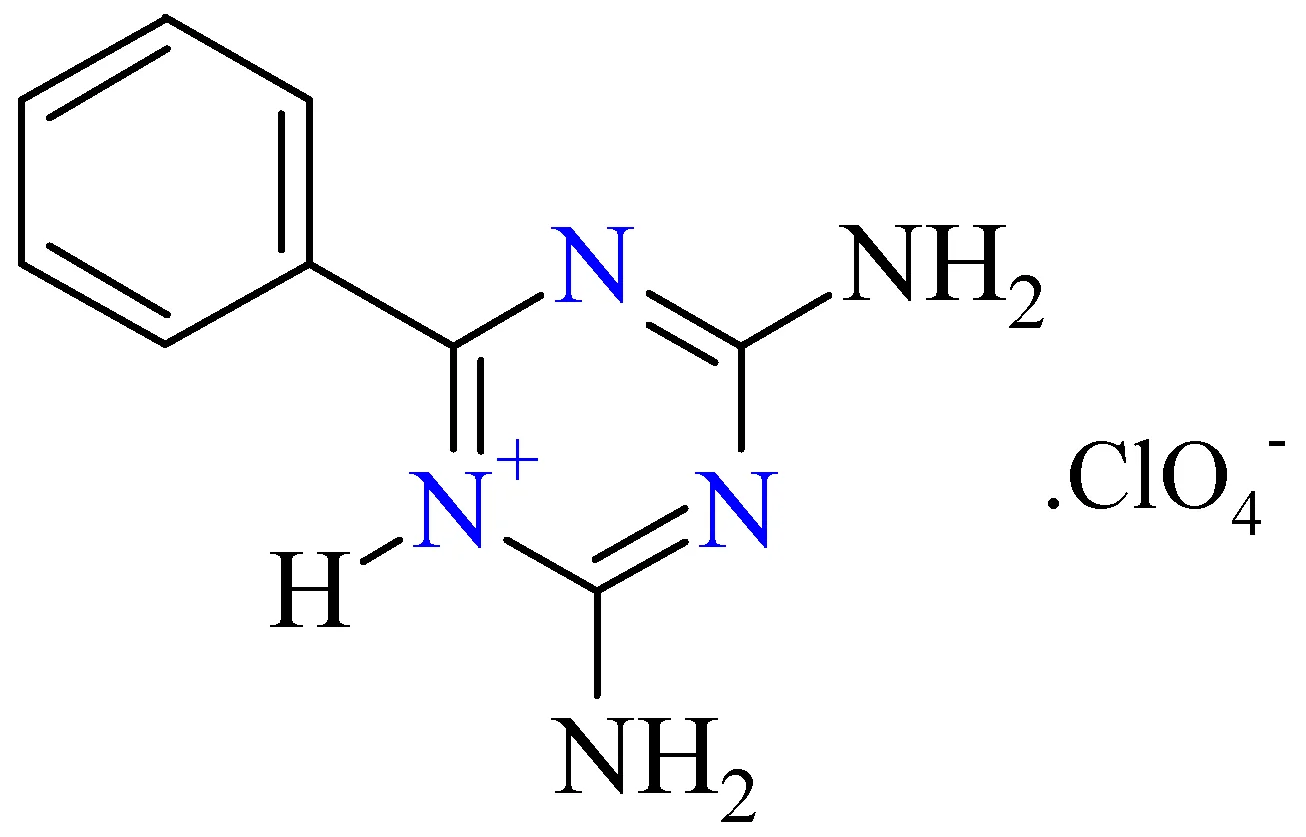
Developed empirical formula for 2,4-diamino-6-phenyl-1,3,5-triaziniumperchlorate.

**Figure 1:**
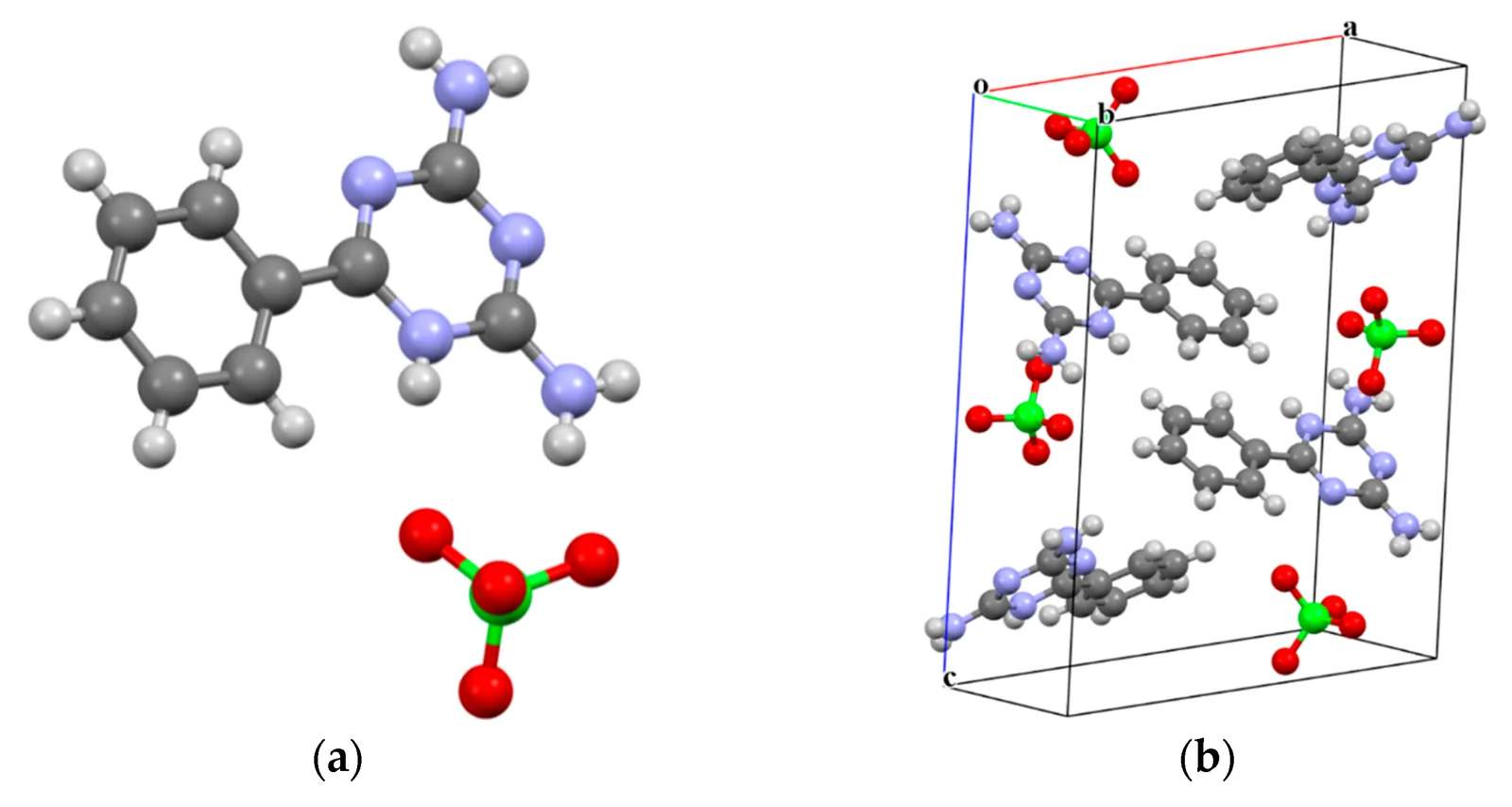
(**a**) Asymmetric unit; (**b**) unit cell of 2,4-diamino-6-phenyl-1,3,5-triaziniumperchlorate.

**Figure 2:**
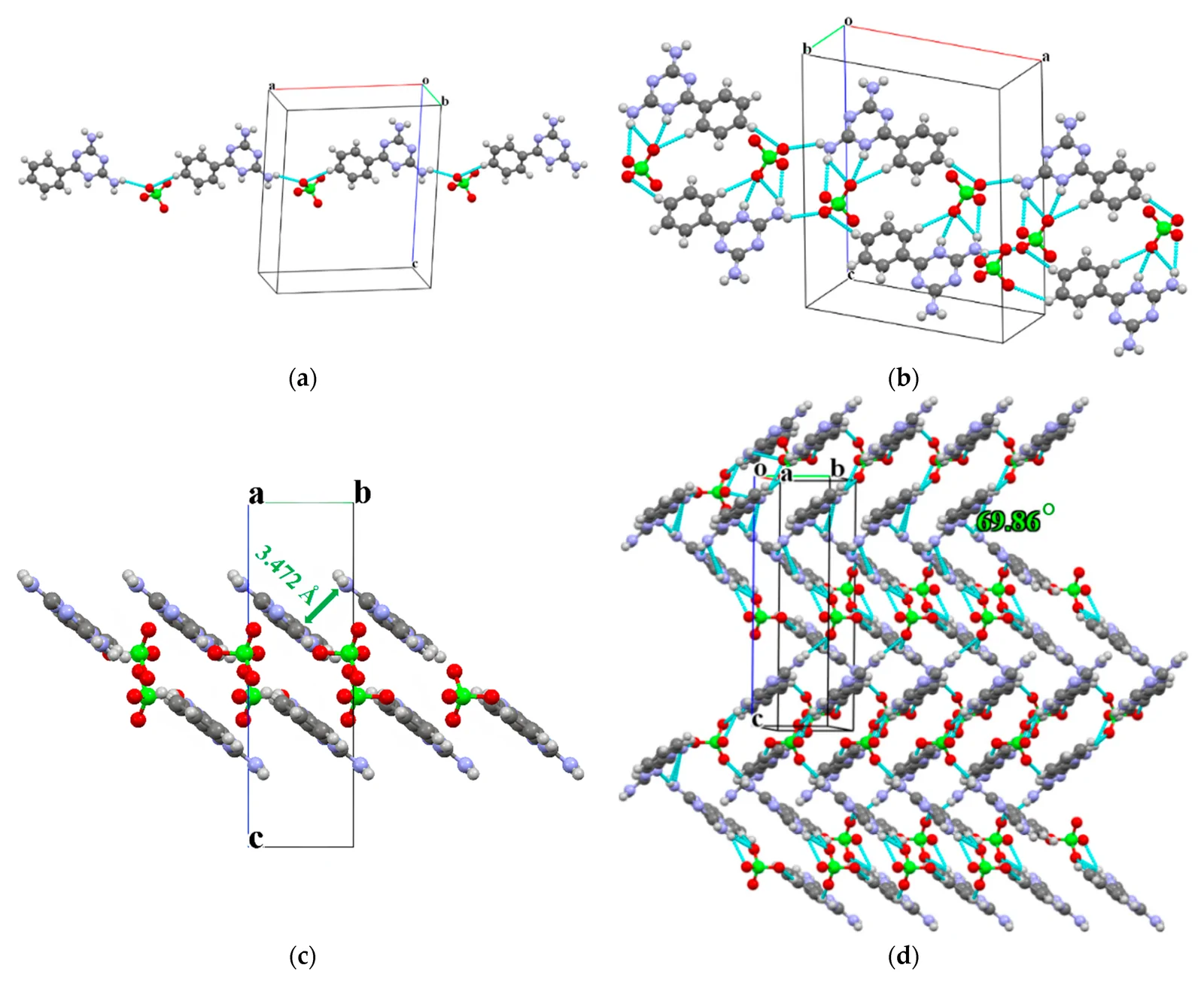
(**a**) Supramolecular chains along the [1 0 0] direction, (**b**) supramolecular parallel-displaced π-stacking along the [0 1 0] direction, (**c**) supramolecular network along the [0 1 0] direction, and (**d**) 3D supramolecular network, illustrating the layer-by-layer arrangement along the direction [0 0 1].

**Figure 3:**
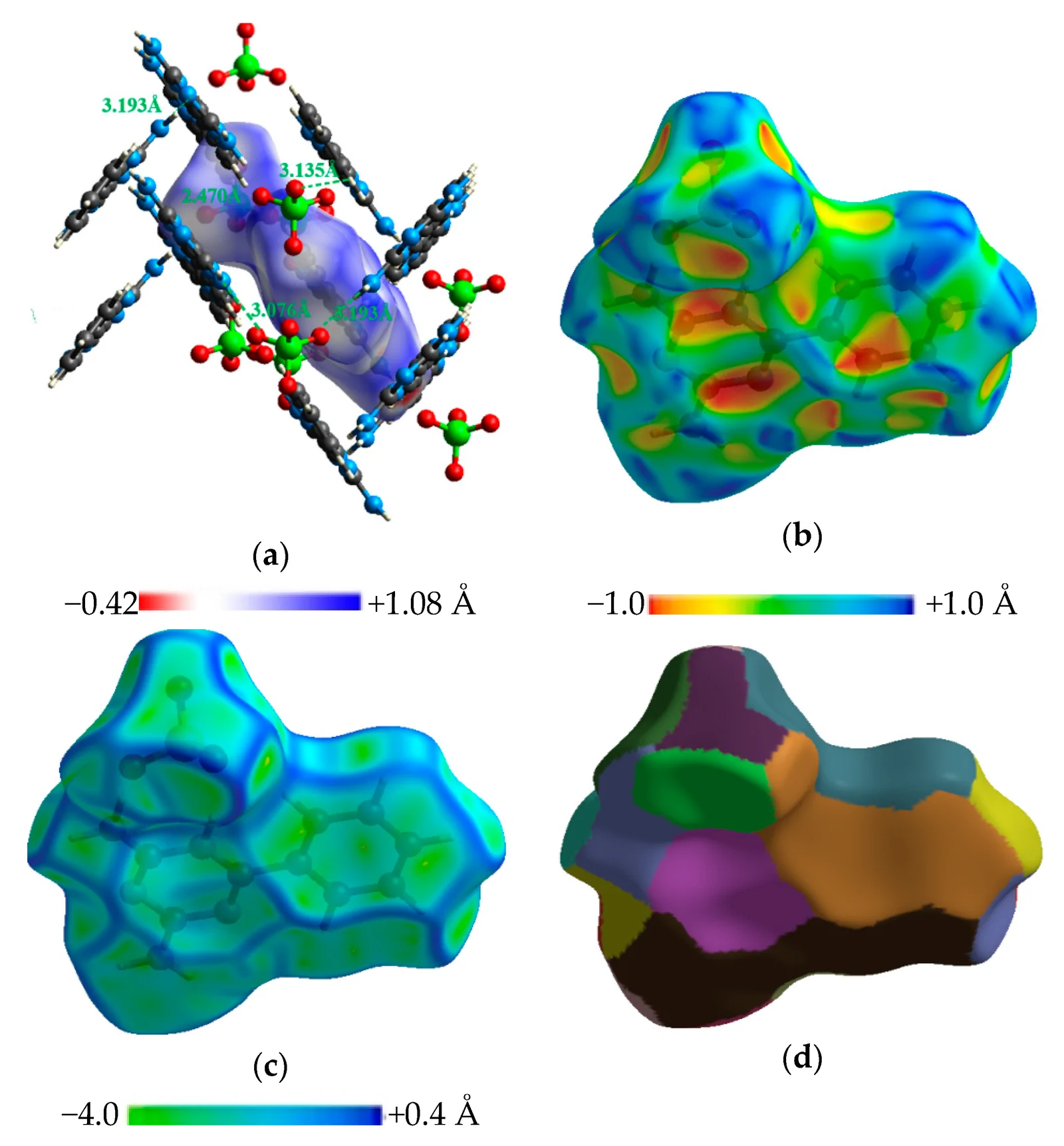
Hirshfeld surfaces of C_9_H_10_N_5_^+^·ClO_4_^−^: (**a**) 3D d_norm_ surface showing the various intermolecular contacts in the crystal, (**b**) surface index, (**c**) curvedness, and (**d**) fragment patch.

**Figure 4:**
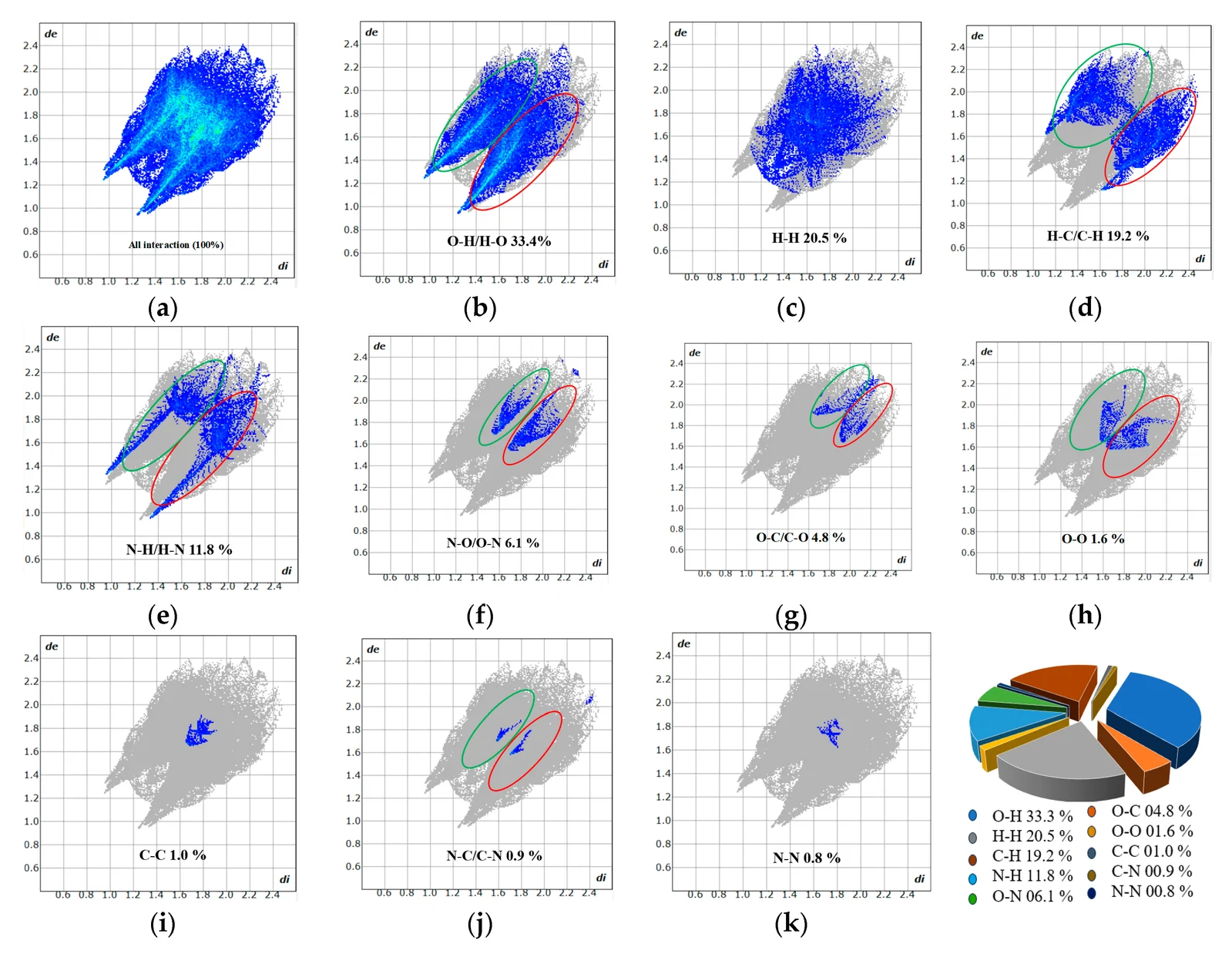
Two-dimensional fingerprint plots of C_9_H_10_N_5_^+^·ClO_4_^−^: showing the percentages of contacts contributing to the total Hirshfeld surface area of the molecule ($d_{i}$ = internal distance, $d_{e}$ = external distance).

**Figure 5:**
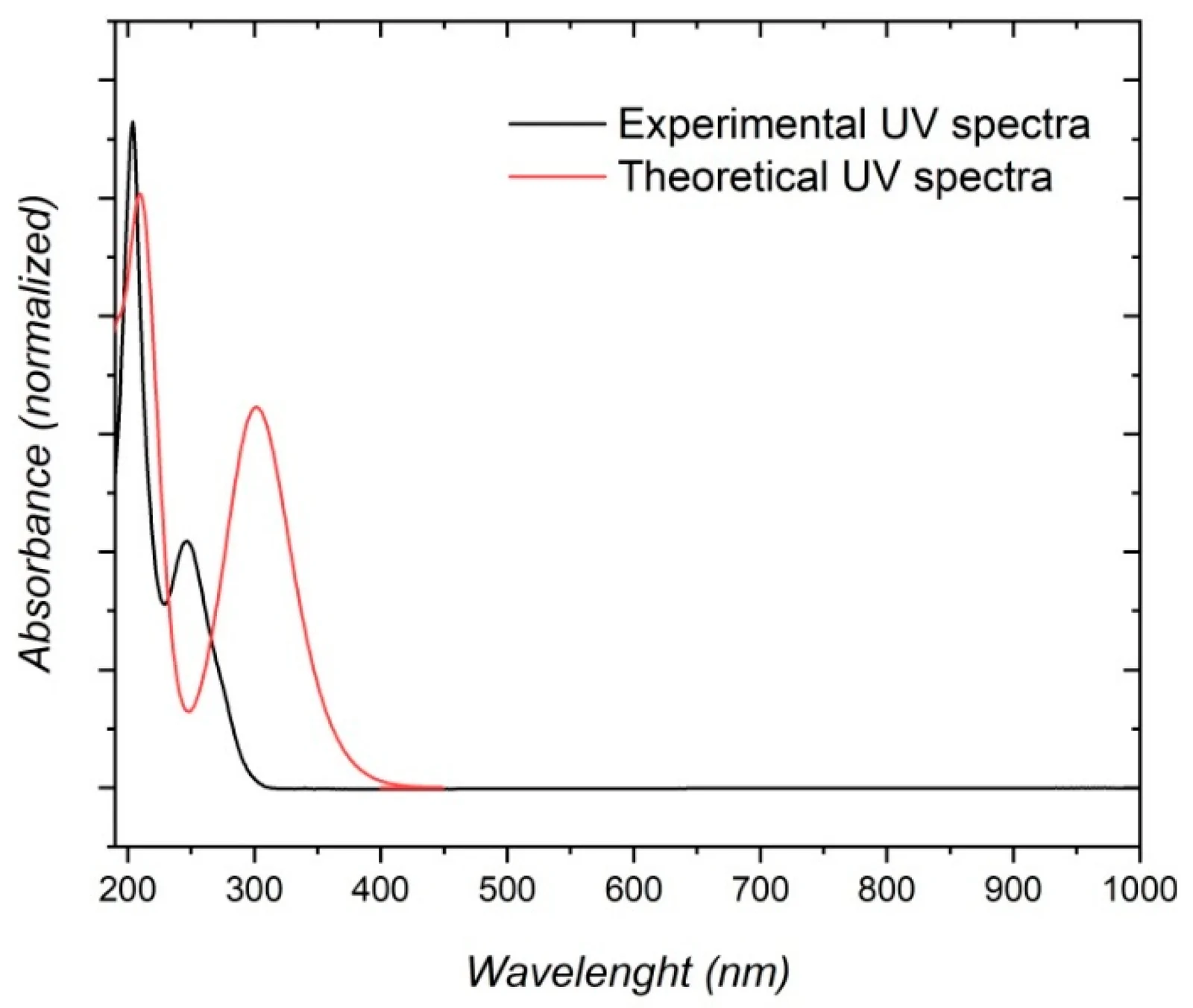
UV-Vis analysis: comparison of simulated and experimental spectra of 2,4-diamino-6-phenyl-1,3,5-triaziniumperchlorate.

**Figure 6:**
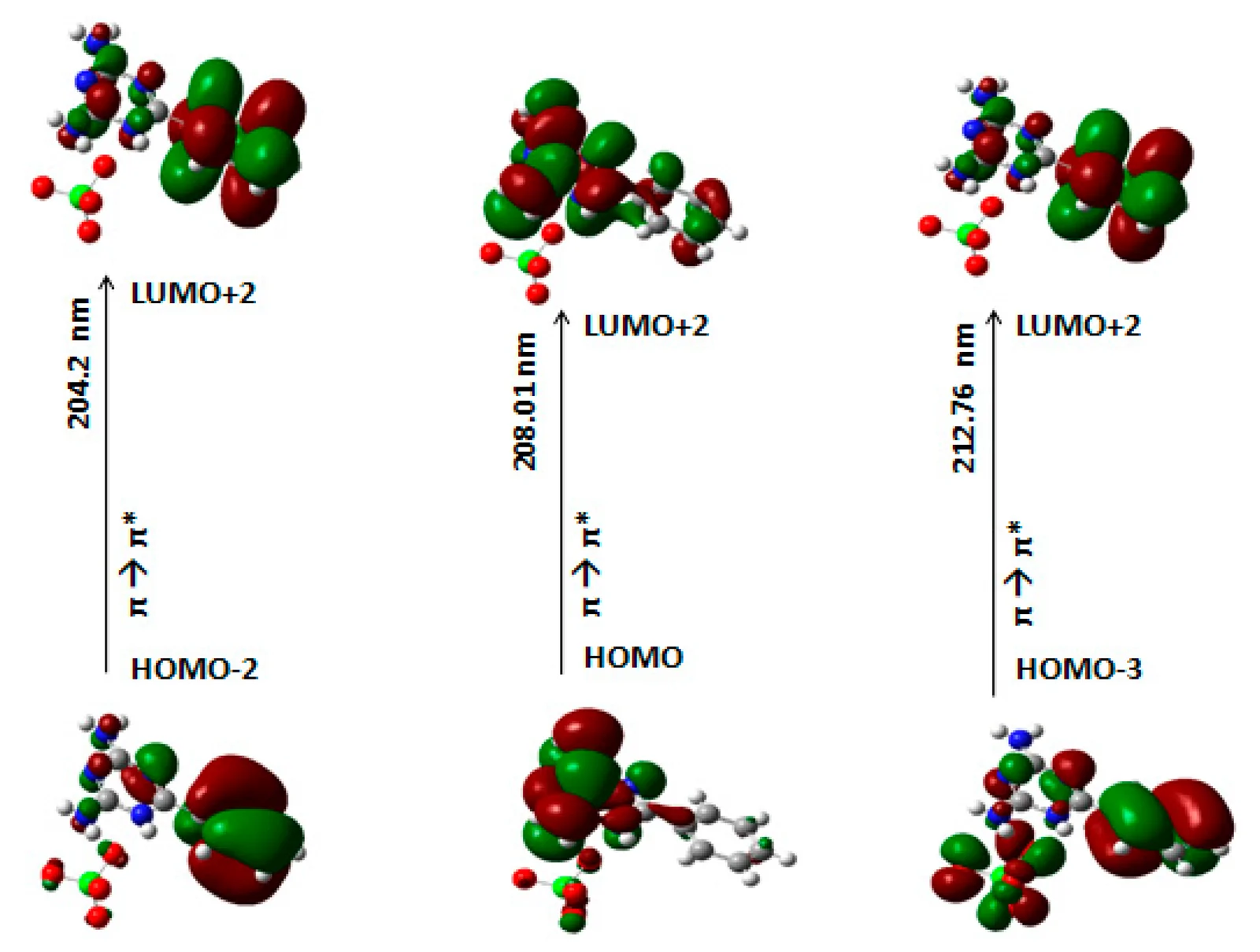

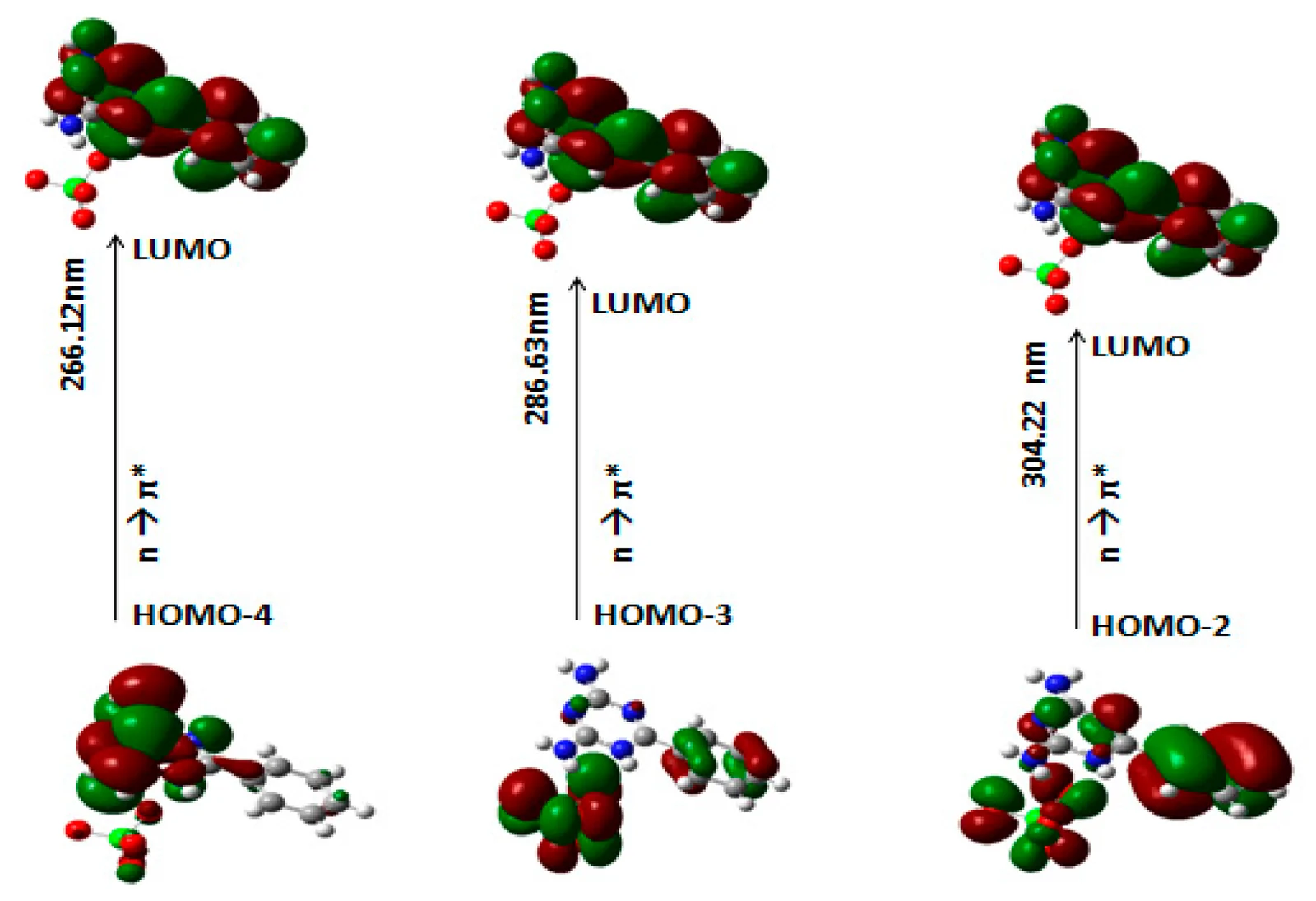
Key spectral features, including absorption maxima (nm), electronic transition characteristics (e.g., π→π, n→π), and molecular orbital geometry (HOMO-LUMO spatial distribution), were analyzed for the 2,4-diamino-6-phenyl-1,3,5-triazinium perchlorate derivative to correlate experimental UV-Vis data with computational electronic structure calculations.

**Figure 7:**
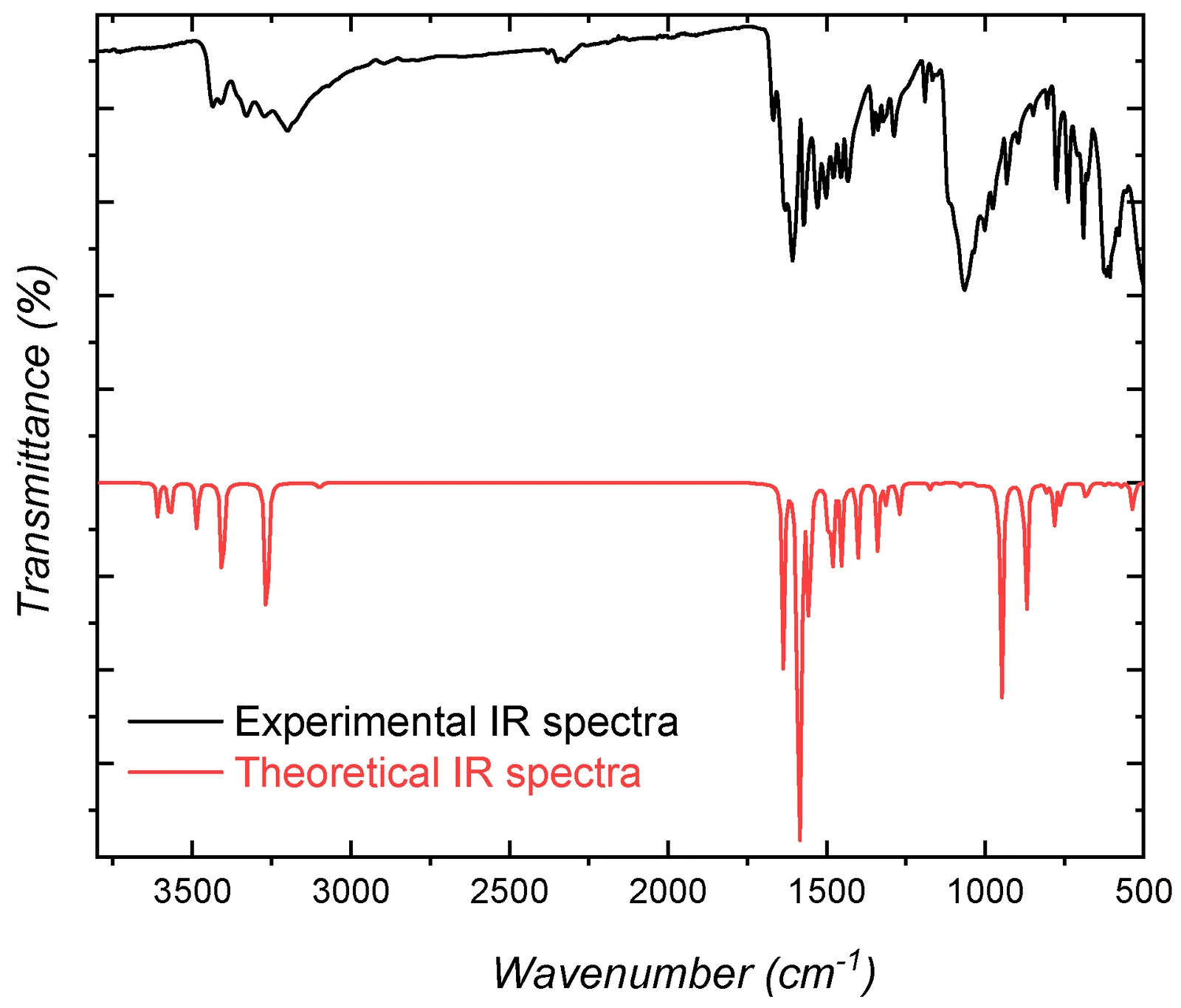
Comparative analysis of simulated and experimental FT-IR spectra.

**Figure 8:**
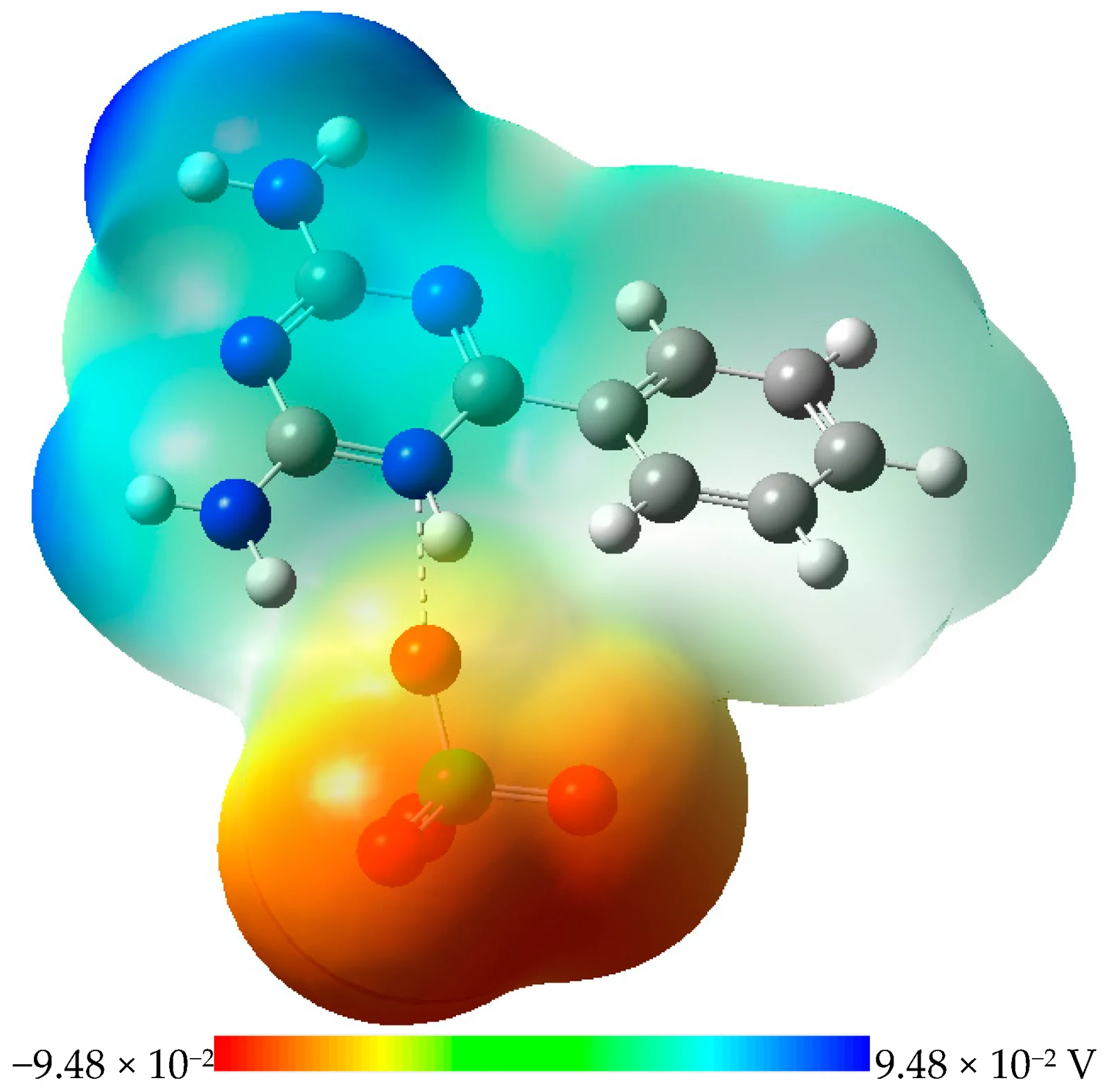
Total electron density surfaces mapped with electrostatic potential (MEP) of the asymmetric unit of title compound.

**Figure 9:**
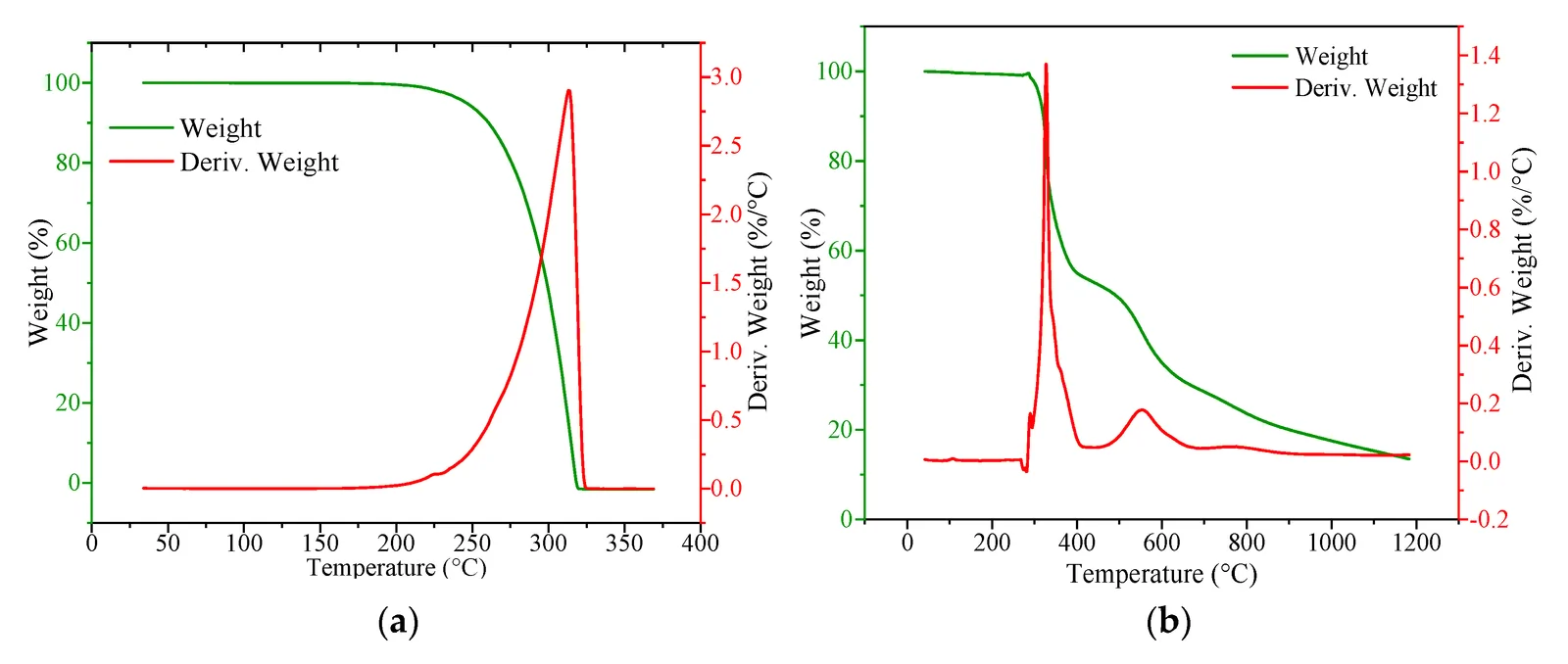
TGA/DTG curves of (**a**) benzoguanamine and (**b**) 2,4-diamino-6-phenyl-1,3,5-triazinium perchlorite.

**Figure 10:**
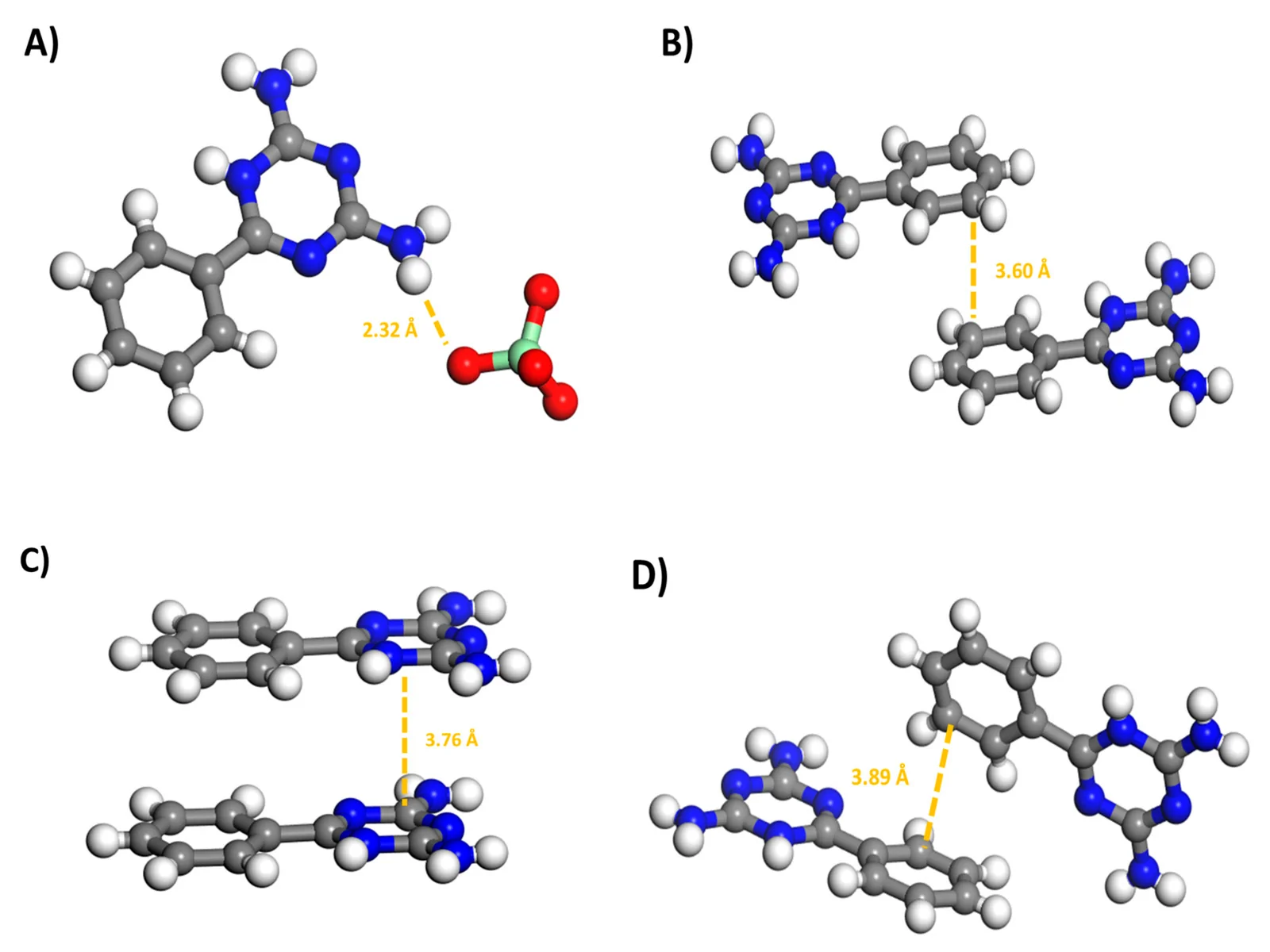
Selected intermolecular dimers used for the quantitative interaction-energy analysis of 2,4-diamino-6-phenyl-1,3,5-triazinium perchlorate: (**A**) cation–anion perchlorate O---H–N hydrogen-bonded pair; (**B**) parallel face-to-face π---π interaction between carbon-based aromatic rings; (**C**) face-to-face π---π interaction involving the N-C-N-C heteroaromatic ring; (**D**) perpendicular π-contact between carbon-based aromatic rings.

**Table 1:** Crystal data structure and the parameters of refinement.

Empirical Formula	C_9_H_10_N_5_^+^·ClO_4_^−^
Molecular weight (g/mol)	244.61
Crystal system	Monoclinic
Crystal color/habit	Colorless/prism
Space group	*P*2_1_/*c*
*a* (Å)	13.7975 (6)
*b* (Å)	5.0711 (2)
*c* (Å)	16.6471 (9)
β (°)	91.869 (2)
*V* (Å^3^)	1164.15 (9)
*Z*	4
Crystal size (mm^3^)	0.60 × 0.40 × 0.35
*D*_calc_ (Mg/m^3^)	1.641
λ _MoK*α*1_ (Å)	0.7107
T (K)	150
*μ* (mm^−1^)	0.35
*θ* Range (°)	2.5→27.3
*F*(000)	592
Δ ρ_min_ (eÅ^−3^)	−0.42
Δ ρ_max_ (eÅ^−3^)	0.36
H	−17→17
K	−6→+6
L	−21→21
Independent reflections	2681
Measured reflections	9047
Reflections with I > 2σ(I)	2094
No. of parameters	187
R_int_	0.027
*wR*(*F*^2^)	0.127
*R* [F^2^ *>* 2*σ*(F2)]	0.042
*S*	1.03

**Table 2:** Some quantitative measures of the Hirshfeld surface characteristics of the crystal under investigation.

Molecular Volume V_H_ (Å^3^)	Surface Area S_H_ (Å^2^)	Globularity (G)	Asphericity (Ω)
285.12	279.39	0.750	0.132

**Table 3:** UV-Vis absorption spectral analysis: experimental and theoretical electronic transitions.

Theoretical						Experimental
No.	Wavelength (nm)	Band Gap (eV)	Energy (cm^−1^)	Oscillatory Strength	Assignments (Major Contributions)	Wavelength Max (nm)
1	204.2	6.07	51,942	0.1138	HOMO-2→LUMO+2 (50.1%)	204
2	208.01	5.96	43,231	0.2628	HOMO→LUMO+2 (50.1%)	247
3	212.76	5.83	47,022	0.7433	HOMO-3→LUMO+2 (48.7%)	
4	266.12	4.66	37,585	0.1479	HOMO-4→LUMO (10.9%)	
5	286.63	4.33	34,924	0.1672	HOMO-3→LUMO (90.8%)	
6	304.22	4.08	32,907	0.5968	HOMO-2→LUMO (69.4%)	

**Table 4:** Comparative analysis of simulated and experimental IR spectra: vibrational mode assignment for 2,4-Diamino-6-phenyl-1,3,5-triazinium perchlorate.

Exp. (cm^−1^)	Cal. (cm^−1^)		Assignments
	Unscaled	Scaled	
3514	3592	3484.24	Symmetric N–H stretching (amine)
3327	3365	3264.05	Symmetric N–H and H–N stretching (amine)
1669	1686	1635.42	N–H bending (scissoring) and C–N stretching
1629	1637	1587.89	N–H bending (scissoring), C–C/C–N stretching, and aromatic ring deformation
1606	1602	1553.94	C–N and C–C stretching vibrations
1567 and 1528	1539	1492.83	C–N stretching, triazine-ring vibration, and N–H bending
1454 and 1429	1476	1431.72	Ring semi-circular stretching, exocyclic C–N contraction, and C–C stretching
1390	1380	1338.6	C-N stretching and bending of N-H
978	978	946.72	Asymmetric stretching of ClO_4_^−^ group
890	897	870.05	H-bond vibrations (ClO_4_^−^ and triazine N), radial in-phase vibration, and ClO_4_^−^ asymmetric stretching
806	805	780.5	Symmetric stretching of ClO_4_^−^ group
691	688	667.36	Out-of-plane N–H bending, symmetric triazine-ring vibration, and C–H bending
567	553	536.41	C–N twisting and torsional vibration of ClO_4_^−^

**Table 5:** Selected dominant intermolecular interaction motifs and corresponding BSSE-corrected interaction energies in the crystal structure of 2,4-diamino-6-phenyl-1,3,5-triazinium perchlorate.

Interaction Motif	Structural Interpretation	BSSE-Corrected Interaction Energy (kJ mol^−1^)
Cation–anion perchlorate O---H–N	Principal cohesive interaction	−248.8
Parallel face-to-face π---π interaction between carbon aromatic rings	Pairwise destabilizing packing contact	+148.9
Face-to-face π---π interaction involving the N-C-N-C ring	Pairwise destabilizing packing contact	+118.8
Perpendicular π-contact involving carbon aromatic rings	Pairwise destabilizing packing contact	+165.5

**Table 6:** Nonlinear optical parameters of C_9_H_10_N_5_^+^·ClO_4_^−^.

**Units**	**(a.u.)**	**(esu) × 10^−24^**
α_xx_	250.932	037.184
α_yy_	088.399	013.099
α_zz_	250.159	037.069
α_yx_	−004.111	−000.609
α_zx_	−006.546	−000.970
α_zy_	003.578	000.530
<α>	196.496	029.117
Δα	114.655	016.990
**Units**	**(a.u.)**	**(esu) × 10^−30^**
β_XXX_	−0913.078	−7.888
β_YYY_	−1433.052	−12.380
β_ZZZ_	−3803.180	−32.856
β_XYY_	−0514.260	−4.442
β_XXY_	−0322.388	−2.785
β_XXZ_	−0683.071	−5.901
β_XZZ_	−0622.712	−5.379
β_YZZ_	−1132.477	−9.783
β_YYZ_	−1335.069	−11.534
β_XYZ_	−0143.481	−1.239
β_tot_	4527.870	39.117
β_tot_ of urea	90.400	0.781

## Data Availability

The crystallographic data have been deposited with the Cambridge Crystallographic Data Centre (CCDC) under deposition number 2412879. The supporting crystallographic information file (CIF) is provided with the Supplementary Materials. Other data are contained within the article.

## Supplementary Materials

The following supporting information can be downloaded at: https://www.mdpi.com/article/10.3390/molecules31193369/s1, Table S1. Selected characteristic geometric parameters (Å, °); Table S2. Geometric properties of the hydrogen bonds (Å, °). crystallographic information file (CIF) for 2,4-diamino-6-phenyl-1,3,5-triazinium perchlorate (CCDC 2412879).

## Abbreviations

The following abbreviations are used in this manuscript:

SXRD	Single-crystal X-ray diffraction
UV-Vis	Ultraviolet–visible spectroscopy
FT-IR	Fourier Transform Infrared
TGA	Thermogravimetric analysis
DTG	Derivative thermogravimetry
CCDC	Cambridge Crystallographic Data Centre
3D	Three- dimensional
2D	Two-dimensional
XRD	X-ray diffraction
HS	Hirshfeld surface
d_norm_	Normalized contact-distance mapping is utilized to categorize areas of specific interest for intermolecular interactions
d_i_	Distance of any HS point to the inner surface of the closest nucleus to the surface
d_e_	Distance of any HS point to the outer surface of the closest nucleus to the surface
vdW	Van der Waals
$r_{i}^{vdW}$	Van der Waals radius of the appropriate atom (internal)
$r_{e}^{vdW}$	Van der Waals radius of the appropriate atom (external)
