# Supplementary material for: Synthesis, Crystal Structure, Hirshfeld Surface, Vibrational Thermal Analyses, and NLO Properties of a New Hybrid Material: 2,4-Diamino-6-Phenyl-1,3,5-Triazinium Perchlorate, Supported by DFT Calculations
Source: Molecules. 2026 Sep 22;31(19):3369. doi: 10.3390/molecules31193369 (PMC13665900; doi:10.3390/molecules31193369)
Supplement: Supplementary file 1 [file molecules-31-03369-s001.zip › File S1 Crystallographic Information File.pdf]

```
###########################################################################
#
# This file contains crystal structure data downloaded from the
# Cambridge Structural Database (CSD) hosted by the Cambridge
# Crystallographic Data Centre (CCDC).
#
# Full information about CCDC data access policies and citation
# guidelines are available at http://www.ccdc.cam.ac.uk/access/V1
#
# Audit and citation data items may have been added by the CCDC.
# Please retain this information to preserve the provenance of
# this file and to allow appropriate attribution of the data.
#
###########################################################################

data_MH1x3
_audit_block_doi                 10.5517/ccdc.csd.cc2lzst6
_database_code_depnum_ccdc_archive 'CCDC 2412879'
loop_
_audit_author_name
_audit_author_address
'Riadh Bourzami'
;Setif university
Algeria
;
_audit_update_record
;
2024-12-25 deposited with the CCDC.	2026-09-20 downloaded from the CCDC.
;

#----------------------------------------------------------------------------#
# Date of experiment: 07 juin 2024                                           #
#----------------------------------------------------------------------------#


#----------------------------------------------------------------------------#
#                          CHEMICAL INFORMATION                              #
#----------------------------------------------------------------------------#

_chemical_name_systematic
'2,4-diamino-6-phenyl-1,3,5-triazinium perchlorate'
_chemical_formula_moiety          'C9 H10 Cl N5 O4'
_chemical_formula_sum             'C9 H10 Cl N5 O4'
_chemical_formula_weight          287.67
_chemical_compound_source         'synthesis as described'

#----------------------------------------------------------------------------#
#                   SPACE GROUP AND UNIT CELL INFORMATION                    #
#----------------------------------------------------------------------------#
```

_space_group_crystal_system monoclinic
_space_group_name_H-M_alt 'P 21/c'
_space_group_name_Hall '-P 2ybc'
_space_group_IT_number 14
loop_
_space_group_symop_operation_xyz
'x, y, z'
'-x, y+1/2, -z+1/2'
'-x, -y, -z'
'x, -y-1/2, z-1/2'

_cell_length_a 13.7975(6)
_cell_length_b 5.0711(2)
_cell_length_c 16.6471(9)
_cell_angle_alpha 90
_cell_angle_beta 91.869(2)
_cell_angle_gamma 90
_cell_volume 1164.15(9)
_cell_formula_units_Z 4
_cell_measurement_temperature 150(2)
_cell_measurement_reflns_used 2778
_cell_measurement_theta_min 2.4482
_cell_measurement_theta_max 27.2733
_cell_measurement_wavelength 0.710730

#--#
ORIENTATION MATRIX
#--#

_diffrn_orient_matrix_UB_11 -0.0331759
_diffrn_orient_matrix_UB_21 -0.0427671
_diffrn_orient_matrix_UB_31 -0.0482579
_diffrn_orient_matrix_UB_12 -0.0232514
_diffrn_orient_matrix_UB_22 -0.1383428
_diffrn_orient_matrix_UB_32 0.1385868
_diffrn_orient_matrix_UB_13 -0.0538406
_diffrn_orient_matrix_UB_23 0.0228718
_diffrn_orient_matrix_UB_33 0.0137984

#--#
CRYSTAL INFORMATION
#--#

_exptl_crystal_description prism
_exptl_crystal_colour colourless
_exptl_crystal_size_max 0.600
_exptl_crystal_size_mid 0.400
_exptl_crystal_size_min 0.350

```
_exptl_crystal_density_meas      ?
_exptl_crystal_density_diffrn    1.641
_exptl_crystal_density_method    ?
_exptl_crystal_F_000             592
_exptl_special_details
;
 ?
;

#----------------------------------------------------------------------------#
#                        ABSORPTION CORRECTION                               #
#----------------------------------------------------------------------------#

_exptl_absorpt_coefficient_mu    0.349
_exptl_absorpt_correction_type   multi-scan
_exptl_absorpt_process_details
;
  [Sheldrick, G.M. (2014). SADABS Bruker AXS Inc., Madison, Wisconsin, USA]
;
_exptl_absorpt_correction_T_min  0.817
_exptl_absorpt_correction_T_max  0.885

#----------------------------------------------------------------------------#
#                        DATA COLLECTION                                     #
#----------------------------------------------------------------------------#

# Software                 : BIS 2014.5.0.0/23-May-2014 && APEX2_2014.11-0   #
# Number of scans          :        3                                        #
# Total number of frames   :      376                                        #
# Total length of scans    :   376.00 (deg.)                                 #
# Rotation speed           :    10.00 sec./deg.                              #
# Total exposition time    :       1 h      2 min.                           #
#                                                                            #
#    Scan  Time(s) Width DX(mm) Frames Range 2Theta  Omega    Phi    Chi  T(K) #
#............................................................................#
#                                                                            #
#  1 Omega   10.0  1.00    35.0   121  121.0   14.9  335.3   36.6   -3.3 296.1 #
#  2 Omega   10.0  1.00    35.0    97   97.0   28.3  330.0  288.8    3.4 296.1 #
#  3 Omega   10.0  1.00    35.0   158  158.0   49.4  331.6  188.0   -0.9 296.1 #
#                                                                            #
#............................................................................#

_diffrn_measurement_device_type  'APEXII Bruker-AXS'
_diffrn_measurement_method       'CCD rotation images, thin slices'
_diffrn_detector                 CCD-LDI-APEX2
_diffrn_detector_area_resol_mean 8.33
_diffrn_ambient_temperature      150(2)
_diffrn_radiation_wavelength     0.71073
_diffrn_radiation_type           MoK\a
_diffrn_radiation_probe          x-ray
```

```
_diffrn_radiation_source         'fine-focus sealed tube'
_diffrn_radiation_monochromator  graphite
_diffrn_source_current          30.00
_diffrn_source_voltage          50.00
_diffrn_source_power            50.0
_diffrn_reflns_av_uneti/neti     0.0274
_diffrn_reflns_av_r_equivalents  0.0268
_diffrn_reflns_number           9047
_diffrn_reflns_limit_h_min       -17
_diffrn_reflns_limit_h_max       17
_diffrn_reflns_limit_k_min       -6
_diffrn_reflns_limit_k_max       6
_diffrn_reflns_limit_l_min       -21
_diffrn_reflns_limit_l_max       21
_diffrn_reflns_theta_min         2.818
_diffrn_reflns_theta_max         27.509
_diffrn_reflns_theta_full        25.242
_diffrn_measured_fraction_theta_full 0.999
_diffrn_measured_fraction_theta_max 0.999
_reflns_number_total            2681
_reflns_number_gt               2094
_reflns_threshold_expression     >2sigma(I)

_reflns_special_details
;
Reflections were merged by SHELXL according to the crystal class for the
calculation of statistics and refinement.
;

#----------------------------------------------------------------------------#
#                         COMPUTER PROGRAMS USED                       #
#----------------------------------------------------------------------------#

_computing_data_collection       'Bruker APEX3 (Bruker, 2015)'
_computing_cell_refinement       'APEX3 (Bruker, 2015)'
_computing_data_reduction        'SAINT (Bruker, 2014)'
_computing_structure_solution    'SHELXT 2018_2 (Sheldrick, 2018)'
_computing_structure_refinement  'SHELXL-2018_3 (Sheldrick, 2018)'
_computing_molecular_graphics
;
  'SXGRAPH (L. Farrugia, 1999), Mercury (CSD, 2020)'
;
_computing_publication_material  'CRYSCALC (T. Roisnel, local program, 2020)'

#----------------------------------------------------------------------------#
#                         REFINEMENT INFORMATION                       #
#----------------------------------------------------------------------------#

_refine_special_details
;
```

Refinement of F^2^ against ALL reflections. The weighted R-factor wR and goodness of fit S are based on F^2^, conventional R-factors R are based on F, with F set to zero for negative F^2^. The threshold expression of F^2^ > 2sigma(F^2^) is used only for calculating R-factors(gt) etc. and is not relevant to the choice of reflections for refinement. R-factors based on F^2^ are statistically about twice as large as those based on F, and R-factors based on ALL data will be even larger.
;
_refine_ls_structure_factor_coef Fsqd
_refine_ls_matrix_type full
_refine_ls_weighting_scheme calc
_refine_ls_weighting_details
'w=1/[\s^2^(Fo^2^)+(0.0758P)^2^+0.2470P] where P=(Fo^2^+2Fc^2^)/3'
_atom_sites_solution_primary dual
_atom_sites_solution_secondary ?
_atom_sites_solution_hydrogens mixed
_refine_ls_hydrogen_treatment mixed
_refine_ls_extinction_method none
_refine_ls_extinction_coef .
_refine_ls_number_reflns 2681
_refine_ls_number_parameters 187
_refine_ls_number_restraints 0
_refine_ls_R_factor_all 0.0560
_refine_ls_R_factor_gt 0.0415
_refine_ls_wR_factor_ref 0.1268
_refine_ls_wR_factor_gt 0.1143
_refine_ls_goodness_of_fit_ref 1.033
_refine_ls_restrained_S_all 1.033
_refine_ls_shift/su_max 0.001
_refine_ls_shift/su_mean 0.000
_refine_diff_density_max 0.356
_refine_diff_density_min -0.419
_refine_diff_density_rms 0.059

#--#
ATOMIC TYPES, COORDINATES AND THERMAL PARAMETERS
#--#

loop_
_atom_type_symbol
_atom_type_description
_atom_type_scat_dispersion_real
_atom_type_scat_dispersion_imag
_atom_type_scat_source
C C 0.0033 0.0016 'International Tables Vol C Tables 4.2.6.8 and 6.1.1.4'
H H 0.0000 0.0000 'International Tables Vol C Tables 4.2.6.8 and 6.1.1.4'
N N 0.0061 0.0033 'International Tables Vol C Tables 4.2.6.8 and 6.1.1.4'
Cl Cl 0.1484 0.1585 'International Tables Vol C Tables 4.2.6.8 and 6.1.1.4'
O O 0.0106 0.0060 'International Tables Vol C Tables 4.2.6.8 and 6.1.1.4'

```
loop_
_atom_site_label
_atom_site_type_symbol
_atom_site_fract_x
_atom_site_fract_y
_atom_site_fract_z
_atom_site_U_iso_or_equiv
_atom_site_adp_type
_atom_site_occupancy
_atom_site_site_symmetry_order
_atom_site_calc_flag
_atom_site_refinement_flags_posn
_atom_site_refinement_flags_adp
_atom_site_refinement_flags_occupancy
_atom_site_disorder_assembly
_atom_site_disorder_group
C1 C 0.11028(13) 0.4550(4) 0.37151(12) 0.0300(4) Uani 1 1 d . . . . .
C2 C 0.15140(13) 0.1301(4) 0.28552(11) 0.0294(4) Uani 1 1 d . . . . .
C3 C 0.27399(13) 0.3614(4) 0.34644(11) 0.0284(4) Uani 1 1 d . . . . .
C4 C 0.37796(14) 0.4226(4) 0.36055(12) 0.0315(4) Uani 1 1 d . . . . .
C5 C 0.44548(15) 0.2597(5) 0.32595(14) 0.0464(5) Uani 1 1 d . . . . .
H5 H 0.424283 0.114855 0.293715 0.056 Uiso 1 1 calc R U . . .
C6 C 0.54339(16) 0.3065(6) 0.33801(16) 0.0544(7) Uani 1 1 d . . . . .
H6 H 0.589074 0.191361 0.314843 0.065 Uiso 1 1 calc R U . . .
C7 C 0.57525(16) 0.5159(5) 0.38279(16) 0.0512(6) Uani 1 1 d . . . . .
H7 H 0.642778 0.547623 0.390459 0.061 Uiso 1 1 calc R U . . .
C8 C 0.50934(18) 0.6803(6) 0.4167(2) 0.0641(8) Uani 1 1 d . . . . .
H8 H 0.531447 0.826503 0.447880 0.077 Uiso 1 1 calc R U . . .
C9 C 0.41072(17) 0.6357(5) 0.40585(18) 0.0547(7) Uani 1 1 d . . . . .
H9 H 0.365569 0.751259 0.429504 0.066 Uiso 1 1 calc R U . . .
N1 N 0.20671(12) 0.5042(3) 0.38502(10) 0.0303(4) Uani 1 1 d . . . . .
H1N H 0.2221(17) 0.613(5) 0.4133(15) 0.036 Uiso 1 1 d . U . . .
N2 N 0.08088(11) 0.2721(3) 0.31918(10) 0.0318(4) Uani 1 1 d . . . . .
N3 N 0.24864(11) 0.1715(3) 0.29741(10) 0.0318(4) Uani 1 1 d . . . . .
N4 N 0.04740(14) 0.5968(4) 0.40985(13) 0.0422(5) Uani 1 1 d . . . . .
H4N H -0.010(2) 0.559(5) 0.4045(16) 0.051 Uiso 1 1 d . U . . .
H4O H 0.0617(19) 0.717(5) 0.4417(17) 0.051 Uiso 1 1 d . U . . .
N5 N 0.12606(14) -0.0669(4) 0.23814(11) 0.0365(4) Uani 1 1 d . . . . .
H5N H 0.0701(18) -0.102(5) 0.2284(15) 0.044 Uiso 1 1 d . U . . .
H5O H 0.1644(19) -0.142(5) 0.2123(15) 0.044 Uiso 1 1 d . U . . .
Cl1 Cl 0.18248(3) 1.03762(9) 0.56233(3) 0.03257(17) Uani 1 1 d . . . . .
O1 O 0.08910(12) 0.9149(4) 0.56263(13) 0.0593(5) Uani 1 1 d . . . . .
O2 O 0.23750(14) 0.9500(4) 0.63044(13) 0.0694(6) Uani 1 1 d . . . . .
O3 O 0.22912(17) 0.9635(4) 0.49004(13) 0.0711(6) Uani 1 1 d . . . . .
O4 O 0.17284(12) 1.3175(3) 0.56400(10) 0.0488(4) Uani 1 1 d . . . . .

loop_
_atom_site_aniso_label
_atom_site_aniso_U_11
_atom_site_aniso_U_22
```

_atom_site_aniso_U_33
_atom_site_aniso_U_23
_atom_site_aniso_U_13
_atom_site_aniso_U_12
C1 0.0256(9) 0.0332(10) 0.0313(9) 0.0037(8) 0.0019(7) 0.0029(8)
C2 0.0263(9) 0.0350(10) 0.0268(9) 0.0043(8) -0.0003(7) -0.0008(8)
C3 0.0242(9) 0.0340(10) 0.0271(9) 0.0052(7) 0.0003(7) 0.0008(8)
C4 0.0244(9) 0.0361(10) 0.0338(10) 0.0050(8) -0.0001(7) -0.0001(8)
C5 0.0300(10) 0.0575(14) 0.0518(13) -0.0138(11) 0.0047(9) -0.0002(10)
C6 0.0271(11) 0.0723(18) 0.0643(16) -0.0087(13) 0.0093(10) 0.0042(11)
C7 0.0254(10) 0.0652(16) 0.0629(16) 0.0085(13) -0.0023(10) -0.0086(11)
C8 0.0364(13) 0.0601(17) 0.095(2) -0.0238(15) -0.0066(13) -0.0096(12)
C9 0.0324(11) 0.0533(14) 0.0781(18) -0.0217(13) -0.0023(11) 0.0005(11)
N1 0.0251(8) 0.0329(9) 0.0328(9) -0.0035(7) 0.0000(7) 0.0006(7)
N2 0.0225(8) 0.0393(9) 0.0334(8) 0.0002(7) 0.0000(6) 0.0023(7)
N3 0.0244(8) 0.0383(9) 0.0326(8) -0.0038(7) 0.0006(6) 0.0017(7)
N4 0.0256(9) 0.0504(11) 0.0509(11) -0.0130(9) 0.0037(8) 0.0044(8)
N5 0.0297(9) 0.0450(11) 0.0346(9) -0.0082(8) -0.0011(7) -0.0007(8)
Cl1 0.0289(3) 0.0310(3) 0.0379(3) -0.00199(18) 0.00269(19) 0.00235(18)
O1 0.0343(9) 0.0565(11) 0.0872(14) -0.0066(10) 0.0026(9) -0.0089(8)
O2 0.0600(12) 0.0671(13) 0.0792(14) 0.0211(10) -0.0267(11) -0.0041(10)
O3 0.0975(16) 0.0499(11) 0.0686(13) -0.0143(9) 0.0445(12) -0.0029(10)
O4 0.0561(10) 0.0309(8) 0.0597(11) -0.0038(7) 0.0072(8) 0.0055(7)

#--#
MOLECULAR GEOMETRY
#--#

_geom_special_details
;
All esds (except the esd in the dihedral angle between two l.s. planes)
are estimated using the full covariance matrix. The cell esds are taken
into account individually in the estimation of esds in distances, angles
and torsion angles; correlations between esds in cell parameters are only
used when they are defined by crystal symmetry. An approximate (isotropic)
treatment of cell esds is used for estimating esds involving l.s. planes.
;
loop_
_geom_bond_atom_site_label_1
_geom_bond_atom_site_label_2
_geom_bond_distance
_geom_bond_site_symmetry_2
_geom_bond_publ_flag
C1 N4 1.309(3) . ?
C1 N2 1.327(3) . ?
C1 N1 1.365(2) . ?
C2 N5 1.313(3) . ?
C2 N2 1.347(3) . ?
C2 N3 1.366(2) . ?

C3 N3 1.303(2) . ?
C3 N1 1.356(2) . ?
C3 C4 1.479(3) . ?
C4 C5 1.385(3) . ?
C4 C9 1.385(3) . ?
C5 C6 1.380(3) . ?
C5 H5 0.9500 . ?
C6 C7 1.362(4) . ?
C6 H6 0.9500 . ?
C7 C8 1.369(4) . ?
C7 H7 0.9500 . ?
C8 C9 1.385(3) . ?
C8 H8 0.9500 . ?
C9 H9 0.9500 . ?
N1 H1N 0.75(2) . ?
N4 H4N 0.82(3) . ?
N4 H4O 0.83(3) . ?
N5 H5N 0.80(2) . ?
N5 H5O 0.79(3) . ?
Cl1 O2 1.4154(18) . ?
Cl1 O4 1.4261(15) . ?
Cl1 O1 1.4309(17) . ?
Cl1 O3 1.4331(18) . ?

loop_
_geom_angle_atom_site_label_1
_geom_angle_atom_site_label_2
_geom_angle_atom_site_label_3
_geom_angle
_geom_angle_site_symmetry_1
_geom_angle_site_symmetry_3
_geom_angle_publ_flag
N4 C1 N2 120.73(18) . . ?
N4 C1 N1 118.49(19) . . ?
N2 C1 N1 120.76(17) . . ?
N5 C2 N2 118.35(17) . . ?
N5 C2 N3 116.42(18) . . ?
N2 C2 N3 125.23(17) . . ?
N3 C3 N1 121.19(17) . . ?
N3 C3 C4 119.59(17) . . ?
N1 C3 C4 119.22(17) . . ?
C5 C4 C9 118.7(2) . . ?
C5 C4 C3 118.11(19) . . ?
C9 C4 C3 123.21(19) . . ?
C6 C5 C4 120.4(2) . . ?
C6 C5 H5 119.8 . . ?
C4 C5 H5 119.8 . . ?
C7 C6 C5 120.7(2) . . ?
C7 C6 H6 119.6 . . ?
C5 C6 H6 119.6 . . ?

C6 C7 C8 119.6(2) . . ?
C6 C7 H7 120.2 . . ?
C8 C7 H7 120.2 . . ?
C7 C8 C9 120.7(2) . . ?
C7 C8 H8 119.7 . . ?
C9 C8 H8 119.7 . . ?
C8 C9 C4 120.0(2) . . ?
C8 C9 H9 120.0 . . ?
C4 C9 H9 120.0 . . ?
C3 N1 C1 120.19(17) . . ?
C3 N1 H1N 120.3(18) . . ?
C1 N1 H1N 119.5(18) . . ?
C1 N2 C2 115.93(15) . . ?
C3 N3 C2 116.54(16) . . ?
C1 N4 H4N 118.3(19) . . ?
C1 N4 H4O 124.7(19) . . ?
H4N N4 H4O 117(3) . . ?
C2 N5 H5N 121.7(18) . . ?
C2 N5 H5O 121.6(19) . . ?
H5N N5 H5O 116(3) . . ?
O2 Cl1 O4 110.18(11) . . ?
O2 Cl1 O1 108.66(12) . . ?
O4 Cl1 O1 110.37(11) . . ?
O2 Cl1 O3 110.27(14) . . ?
O4 Cl1 O3 108.80(11) . . ?
O1 Cl1 O3 108.54(13) . . ?

loop_
_geom_torsion_atom_site_label_1
_geom_torsion_atom_site_label_2
_geom_torsion_atom_site_label_3
_geom_torsion_atom_site_label_4
_geom_torsion
_geom_torsion_site_symmetry_1
_geom_torsion_site_symmetry_2
_geom_torsion_site_symmetry_3
_geom_torsion_site_symmetry_4
_geom_torsion_publ_flag
N3 C3 C4 C5 -5.4(3) . . . . ?
N1 C3 C4 C5 174.53(19) . . . . ?
N3 C3 C4 C9 173.9(2) . . . . ?
N1 C3 C4 C9 -6.1(3) . . . . ?
C9 C4 C5 C6 1.4(4) . . . . ?
C3 C4 C5 C6 -179.3(2) . . . . ?
C4 C5 C6 C7 -1.2(4) . . . . ?
C5 C6 C7 C8 0.5(4) . . . . ?
C6 C7 C8 C9 0.0(5) . . . . ?
C7 C8 C9 C4 0.2(5) . . . . ?
C5 C4 C9 C8 -0.8(4) . . . . ?
C3 C4 C9 C8 179.8(2) . . . . ?

```
N3 C3 N1 C1 -1.6(3) . . . . ?
C4 C3 N1 C1 178.54(17) . . . . ?
N4 C1 N1 C3 179.98(18) . . . . ?
N2 C1 N1 C3 -1.7(3) . . . . ?
N4 C1 N2 C2 -177.39(19) . . . . ?
N1 C1 N2 C2 4.3(3) . . . . ?
N5 C2 N2 C1 174.78(18) . . . . ?
N3 C2 N2 C1 -4.3(3) . . . . ?
N1 C3 N3 C2 1.8(3) . . . . ?
C4 C3 N3 C2 -178.33(16) . . . . ?
N5 C2 N3 C3 -177.84(18) . . . . ?
N2 C2 N3 C3 1.2(3) . . . . ?

loop_
_geom_hbond_atom_site_label_D
_geom_hbond_atom_site_label_H
_geom_hbond_atom_site_label_A
_geom_hbond_distance_DH
_geom_hbond_distance_HA
_geom_hbond_distance_DA
_geom_hbond_angle_DHA
_geom_hbond_site_symmetry_A
_geom_hbond_publ_flag
#
# Hydrogen bonding scheme
# ----------------------
#
# D       H       A       D-H        H...A       D...A       D-H...A   symm publ
# ----------------------------------------------------------------------------
N1 H1N O3 0.75(2) 2.19(2) 2.922(3) 165(2) . yes

#----------------------------------------------------------------------------#
#                          EMBEDDED FILES                                    #
#----------------------------------------------------------------------------#
#                                                      #

#..................................................................
# .RES SHELXL file: job.res
#..................................................................

_shelx_res_file
;
TITL MH1x3 [Space group= P2(1)/c]
   job.res
   created by SHELXL-2018/3 at 16:02:06 on 07-Jun-2024
CELL  0.71073  13.7975   5.0711  16.6471   90.000   91.869   90.000
ZERR     4.00   0.0006   0.0002   0.0009    0.000    0.002    0.000
LATT   1
SYMM  - X, 1/2 + Y, 1/2 - Z
SFAC  C    H    N    CL   O
```

```
UNIT  36   40   20   4    16
MERG   2
HTAB N1 O3
FMAP   2
PLAN   20
SIZE     0.35   0.40   0.60
ACTA
BOND   $H
OMIT 1 0 0
CONF
LIST   4
WPDB  -2
L.S.   10
TEMP  -123.00
WGHT    0.075800    0.247000
FVAR       0.63990
MOLE    1
C1    1    0.110275    0.454971    0.371510    11.00000    0.02556    0.03319 =
         0.03129    0.00375    0.00186    0.00290
C2    1    0.151397    0.130089    0.285522    11.00000    0.02633    0.03503 =
         0.02683    0.00428   -0.00032   -0.00080
C3    1    0.273987    0.361365    0.346441    11.00000    0.02416    0.03397 =
         0.02707    0.00522    0.00031    0.00083
C4    1    0.377956    0.422558    0.360547    11.00000    0.02443    0.03606 =
         0.03382    0.00497   -0.00013   -0.00008
C5    1    0.445485    0.259665    0.325947    11.00000    0.03002    0.05750 =
         0.05178   -0.01382    0.00466   -0.00024
AFIX  43
H5    2    0.424283    0.114855    0.293715    11.00000   -1.20000
AFIX   0
C6    1    0.543393    0.306451    0.338012    11.00000    0.02710    0.07227 =
         0.06433   -0.00872    0.00933    0.00422
AFIX  43
H6    2    0.589074    0.191361    0.314843    11.00000   -1.20000
AFIX   0
C7    1    0.575252    0.515882    0.382786    11.00000    0.02535    0.06519 =
         0.06288    0.00846   -0.00226   -0.00864
AFIX  43
H7    2    0.642778    0.547623    0.390459    11.00000   -1.20000
AFIX   0
C8    1    0.509339    0.680317    0.416653    11.00000    0.03642    0.06009 =
         0.09525   -0.02377   -0.00659   -0.00963
AFIX  43
H8    2    0.531447    0.826503    0.447880    11.00000   -1.20000
AFIX   0
C9    1    0.410721    0.635711    0.405851    11.00000    0.03242    0.05326 =
         0.07811   -0.02168   -0.00231    0.00048
AFIX  43
H9    2    0.365569    0.751259    0.429504    11.00000   -1.20000
AFIX   0
```

```
N1   3   0.206715   0.504222   0.385019   11.00000   0.02507   0.03288 =
      0.03282  -0.00345   0.00002   0.00063
AFIX  2
H1N  2   0.222136   0.612594   0.413292   11.00000  -1.20000
AFIX  0
N2   3   0.080879   0.272102   0.319184   11.00000   0.02249   0.03931 =
      0.03341   0.00015   0.00002   0.00227
N3   3   0.248640   0.171514   0.297411   11.00000   0.02445   0.03827 =
      0.03259  -0.00383   0.00064   0.00172
N4   3   0.047395   0.596824   0.409850   11.00000   0.02557   0.05036 =
      0.05090  -0.01298   0.00371   0.00441
AFIX  2
H4N  2  -0.010173   0.559073   0.404457   11.00000  -1.20000
H4O  2   0.061701   0.716789   0.441695   11.00000  -1.20000
AFIX  0
N5   3   0.126061  -0.066887   0.238142   11.00000   0.02975   0.04504 =
      0.03459  -0.00816  -0.00107  -0.00070
H5N  2   0.070071  -0.102211   0.228383   11.00000  -1.20000
H5O  2   0.164412  -0.141808   0.212252   11.00000  -1.20000
MOLE   2
CL1   4   0.182484   1.037622   0.562326   11.00000   0.02891   0.03098 =
      0.03793  -0.00199   0.00269   0.00235
O1   5   0.089104   0.914921   0.562633   11.00000   0.03434   0.05651 =
      0.08717  -0.00665   0.00259  -0.00888
O2   5   0.237501   0.949955   0.630440   11.00000   0.05998   0.06709 =
      0.07916   0.02113  -0.02670  -0.00409
O3   5   0.229121   0.963480   0.490043   11.00000   0.09749   0.04986 =
      0.06861  -0.01431   0.04451  -0.00288
O4   5   0.172839   1.317545   0.564005   11.00000   0.05609   0.03094 =
      0.05970  -0.00381   0.00720   0.00553
HKLF    4




REM  MH1x3 [Space group= P2(1)/c]
REM wR2 = 0.1268, GooF = S = 1.033, Restrained GooF = 1.033 for all data
REM R1 = 0.0415 for 2094 Fo > 4sig(Fo) and 0.0560 for all 2681 data
REM 187 parameters refined using 0 restraints

END

WGHT      0.0591      0.4274

REM Highest difference peak  0.356,  deepest hole -0.419,  1-sigma level  0.059
Q1    1   0.1609  0.9584  0.4784  11.00000  0.05    0.36
Q2    1   0.2840  0.9654  0.5871  11.00000  0.05    0.32
Q3    1   0.3261  0.3744  0.3615  11.00000  0.05    0.22
Q4    1   0.1125  0.9572  0.6140  11.00000  0.05    0.21
Q5    1   0.3582  0.6780  0.4649  11.00000  0.05    0.19
```

```
Q6   1   0.4001  0.5505  0.4330  11.00000  0.05    0.18
Q7   1   0.1106  0.1743  0.3161  11.00000  0.05    0.18
Q8   1   0.0740  0.8608  0.5608  11.00000  0.05    0.18
Q9   1   0.2422  0.8312  0.5169  11.00000  0.05    0.17
Q10  1   0.0373  0.2951  0.2962  11.00000  0.05    0.17
Q11  1   0.2811  0.0576  0.2893  11.00000  0.05    0.17
Q12  1  -0.0622  0.5910  0.3609  11.00000  0.05    0.16
Q13  1   0.4073  0.3302  0.3615  11.00000  0.05    0.16
Q14  1   0.3013  0.2035  0.4217  11.00000  0.05    0.16
Q15  1   0.3420  0.2234  0.4646  11.00000  0.05    0.15
Q16  1   0.0219  0.2158  0.3116  11.00000  0.05    0.15
Q17  1   0.4167  0.2008  0.2591  11.00000  0.05    0.15
Q18  1  -0.0239  0.7783  0.3569  11.00000  0.05    0.15
Q19  1   0.1573  0.5167  0.3631  11.00000  0.05    0.14
Q20  1   0.1501  1.2430  0.5205  11.00000  0.05    0.14
;

_shelx_res_checksum              99291

# ............... End of .RES file .......................................... #
#---------------------------------------------------------------------------#

#...........................................................................
# .HKL SHELXL file: job.hkl
#...........................................................................

_shelx_hkl_file
;
   1   0   0   -1.69     0.09
  -1   0   0   -1.61     0.10
   2   0   0 1152.05    16.30
  -2   0   0 1164.64    16.33
  -3   0   0 9257.04   132.46
  -3   0   0 9587.55   134.19
   3   0   0 9421.19   132.45
  -4   0   0    5.43     0.99
   4   0   0    3.87     0.84
  -4   0   0    3.83     0.89
   5   0   0  924.23    15.24
  -5   0   0  920.46    15.61
   5   0   0  917.97    15.06
  -5   0   0  928.12    15.35
  -6   0   0 1164.93    19.29
   6   0   0 1174.21    19.25
   6   0   0 1204.83    19.10
  -6   0   0 1166.39    19.72
   7   0   0  217.01     6.17
  -7   0   0  202.02     6.47
  -7   0   0  203.89     6.19
   7   0   0  209.47     6.18
```

8	0	0	205.15	6.41
-8	0	0	202.47	6.84
-8	0	0	201.88	6.51
8	0	0	212.24	6.56
-9	0	0	62.92	4.01
9	0	0	53.72	3.50
9	0	0	56.62	3.59
-10	0	0	1.06	1.21
10	0	0	1.37	1.21
10	0	0	1.53	1.30
11	0	0	266.96	8.71
11	0	0	274.79	8.77
-11	0	0	272.31	9.30
-12	0	0	80.41	5.38
12	0	0	81.60	5.01
13	0	0	56.22	4.59
-13	0	0	59.19	4.97
14	0	0	93.64	5.80
-14	0	0	93.32	6.27
15	0	0	13.93	3.13
-15	0	0	10.64	2.95
16	0	0	63.64	5.31
-16	0	0	64.77	6.65
17	0	0	1.88	2.35
-17	0	0	3.82	2.48
0	-1	0	-1.37	0.56
0	1	0	-2.02	0.52
-1	-1	0	1372.85	20.30
1	-1	0	1364.48	19.07
1	1	0	1398.99	20.27
-1	1	0	1379.64	19.06
2	-1	0	259.78	4.34
2	-1	0	258.65	4.27
-2	-1	0	256.83	5.99
2	1	0	260.52	5.76
-2	1	0	258.60	4.33
2	1	0	266.32	6.00
3	-1	0	241.13	4.14
-3	-1	0	243.87	6.15
3	-1	0	248.33	4.25
-3	1	0	239.56	4.23
3	1	0	235.57	6.04
4	-1	0	267.48	5.01
-4	1	0	262.48	4.99
4	1	0	263.38	6.70
4	1	0	264.55	6.86
-5	-1	0	74.31	3.73
5	-1	0	78.39	2.48
5	1	0	80.13	3.80
5	1	0	78.61	3.71

-5	1	0	71.79	2.59
-6	-1	0	58.64	3.60
6	-1	0	65.07	2.53
6	1	0	58.30	3.57
-6	1	0	61.99	2.72
6	1	0	63.17	3.65
-7	-1	0	46.32	3.42
7	-1	0	51.46	2.50
7	-1	0	45.07	2.33
7	1	0	51.51	3.58
-7	1	0	46.21	2.59
7	1	0	52.35	3.55
-8	-1	0	34.21	3.59
8	-1	0	43.45	2.52
-8	-1	0	31.89	3.60
8	-1	0	38.29	2.41
8	1	0	32.65	3.43
8	1	0	41.97	3.35
-8	1	0	41.98	2.63
-9	-1	0	0.49	1.31
-9	-1	0	-1.31	0.85
9	-1	0	0.20	0.96
9	-1	0	0.07	0.82
-9	1	0	-0.47	0.83
9	1	0	-1.02	1.06
10	-1	0	314.41	8.06
-10	-1	0	313.68	10.69
-10	-1	0	312.47	10.26
10	-1	0	323.68	8.27
10	1	0	345.12	10.47
-10	1	0	332.55	8.86
-11	-1	0	61.98	5.17
-11	-1	0	66.35	4.88
11	-1	0	56.52	3.68
11	-1	0	61.98	3.70
-11	1	0	58.36	3.94
11	1	0	57.44	4.64
12	-1	0	55.02	3.70
-12	-1	0	57.50	4.79
-12	-1	0	58.08	5.10
12	-1	0	49.92	3.69
-12	1	0	46.74	3.83
12	1	0	60.86	5.09
-13	-1	0	20.79	3.86
13	-1	0	22.14	3.08
13	-1	0	33.91	3.53
-13	-1	0	29.62	4.15
13	1	0	28.08	4.21
-13	1	0	25.67	3.47
14	-1	0	15.46	2.78

-14	-1	0	16.53	3.66
14	1	0	22.07	3.95
-14	1	0	23.09	3.53
-15	-1	0	-1.82	1.73
15	-1	0	0.72	1.67
15	1	0	1.94	2.20
-15	1	0	0.18	1.59
-16	-1	0	2.47	2.37
16	1	0	5.93	2.70
-17	-1	0	12.07	3.76
17	1	0	24.59	4.70
0	-2	0	1923.13	28.50
0	2	0	1928.75	28.00
0	2	0	1896.91	28.30
1	-2	0	911.20	14.83
-1	-2	0	911.23	15.92
-1	2	0	888.80	14.63
-1	2	0	916.10	14.32
1	2	0	902.94	15.72
1	2	0	900.14	15.37
2	-2	0	1738.54	25.03
-2	-2	0	1760.71	27.48
-2	2	0	1729.39	24.91
2	2	0	1693.76	27.13
2	2	0	1757.10	26.88
-3	-2	0	340.63	8.85
3	-2	0	329.82	6.33
3	-2	0	331.79	6.13
3	2	0	341.82	8.70
-3	2	0	338.78	6.34
4	-2	0	65.88	2.18
4	-2	0	69.02	2.12
-4	-2	0	73.11	4.06
4	2	0	67.58	3.85
4	2	0	60.64	3.71
-5	-2	0	1014.33	19.61
5	-2	0	1007.22	14.60
5	-2	0	988.01	14.61
5	2	0	1019.09	19.13
5	2	0	990.84	19.19
6	-2	0	2.84	0.65
6	-2	0	4.62	0.77
-6	-2	0	1.57	1.39
6	2	0	3.34	1.49
6	2	0	2.36	1.40
-7	-2	0	105.78	5.70
7	-2	0	104.74	2.93
7	-2	0	104.99	2.95
-7	2	0	106.39	3.17
7	2	0	113.44	5.75

-8	-2	0	12.41	2.55
8	-2	0	15.27	1.33
8	-2	0	16.15	1.39
8	2	0	12.72	2.66
-8	2	0	14.38	1.52
-9	-2	0	18.59	3.48
-9	-2	0	20.80	3.32
9	-2	0	19.15	1.63
-9	2	0	17.85	1.88
9	2	0	17.95	3.13
-10	-2	0	218.55	9.55
10	-2	0	208.48	5.13
10	-2	0	204.77	5.32
-10	-2	0	199.99	8.89
10	2	0	215.37	8.93
-10	2	0	203.72	5.81
11	-2	0	0.41	0.89
-11	-2	0	-0.90	1.73
-11	-2	0	-0.96	1.75
-11	2	0	0.47	0.99
11	2	0	-3.96	1.35
-12	-2	0	12.25	3.27
-12	-2	0	30.77	4.92
12	2	0	22.20	4.18
-12	2	0	26.16	2.82
-13	-2	0	169.97	9.04
-13	-2	0	158.26	9.13
13	2	0	160.86	8.83
-14	-2	0	38.06	5.69
-14	-2	0	46.39	5.79
14	2	0	40.07	5.56
-15	-2	0	2.43	2.54
-15	-2	0	-1.67	1.88
15	2	0	6.42	2.99
-16	-2	0	35.03	5.90
16	2	0	31.91	5.43
-17	-2	0	35.92	6.13
17	2	0	47.87	6.52
0	-3	0	-0.23	0.94
0	3	0	-0.81	0.91
0	3	0	-1.39	0.76
-1	-3	0	16.98	2.44
1	-3	0	15.05	2.06
1	3	0	16.78	2.35
-1	3	0	17.00	2.11
-1	3	0	18.91	2.16
1	3	0	16.62	2.31
-2	-3	0	171.12	6.63
2	-3	0	170.31	5.36
-2	3	0	165.84	5.20

2	3	0	163.67	6.36
2	3	0	164.29	6.15
-2	3	0	165.35	4.89
3	-3	0	124.81	4.24
-3	-3	0	125.45	5.89
-3	3	0	126.28	4.22
3	3	0	124.38	5.67
-3	3	0	119.86	3.79
3	3	0	117.13	5.56
-4	-3	0	258.15	8.88
4	-3	0	265.71	5.80
4	-3	0	245.80	5.99
-4	3	0	254.40	5.94
4	3	0	245.70	8.41
5	-3	0	529.80	8.95
5	-3	0	527.39	9.45
5	3	0	535.48	13.56
6	-3	0	6.39	0.84
6	-3	0	6.46	0.95
-6	-3	0	6.73	2.15
6	3	0	3.83	1.85
7	-3	0	134.37	3.09
-7	-3	0	143.87	7.38
7	3	0	132.39	7.00
-8	-3	0	81.59	5.86
8	3	0	84.92	6.01
-9	-3	0	6.61	2.54
9	3	0	5.59	2.48
-10	-3	0	133.23	8.19
-10	-3	0	112.17	7.34
10	3	0	120.97	7.40
-11	-3	0	49.87	5.89
-11	-3	0	39.74	5.61
11	3	0	46.99	5.85
-12	-3	0	66.25	7.29
-12	-3	0	80.20	6.77
12	3	0	73.29	7.39
-13	-3	0	3.70	2.60
-13	-3	0	2.35	2.45
13	3	0	2.85	2.70
-14	-3	0	3.01	2.86
-14	-3	0	5.00	2.91
14	3	0	4.08	3.12
-15	-3	0	24.83	5.39
-15	-3	0	33.59	5.77
15	3	0	36.55	6.16
0	-4	0	61.68	4.18
0	4	0	61.19	4.15
0	4	0	68.07	4.11
-1	-4	0	4.74	1.69

1	-4	0	8.73	1.91
-1	4	0	4.56	1.60
1	4	0	6.50	1.97
-1	4	0	3.61	1.55
2	-4	0	29.91	3.17
-2	-4	0	32.44	3.93
2	4	0	26.90	3.49
-2	4	0	25.60	2.80
-2	4	0	27.62	2.88
3	-4	0	175.31	6.10
-3	-4	0	168.28	7.69
-3	4	0	175.66	5.57
-3	4	0	175.03	5.94
3	4	0	169.06	7.43
-4	-4	0	0.39	1.81
4	-4	0	3.12	1.26
4	4	0	-0.42	1.75
-4	4	0	3.46	1.22
-5	-4	0	85.83	6.03
5	-4	0	96.72	3.92
5	4	0	90.21	6.13
6	-4	0	12.95	1.60
-6	-4	0	13.71	3.21
6	4	0	8.68	2.74
-7	-4	0	68.00	6.70
7	-4	0	57.95	2.28
7	4	0	63.23	6.45
-8	-4	0	126.21	8.00
8	4	0	129.47	7.89
-9	-4	0	47.40	6.23
-9	-4	0	52.82	6.12
9	4	0	61.34	6.71
-10	-4	0	33.55	5.19
-10	-4	0	47.01	6.63
10	4	0	39.43	5.62
-11	-4	0	56.48	7.45
-11	-4	0	39.92	5.83
11	4	0	40.64	5.83
-12	-4	0	-1.51	2.22
-12	-4	0	2.67	2.69
12	4	0	3.32	2.89
-13	-4	0	1.27	2.70
-13	-4	0	-0.87	2.29
13	4	0	4.30	3.31
-14	-4	0	-1.57	2.60
-14	-4	0	7.26	3.63
14	4	0	4.30	3.57
0	-5	0	1.41	1.66
0	5	0	0.85	1.48
-1	-5	0	1.22	1.73

1	-5	0	0.66	1.49
-1	5	0	-1.82	1.29
1	5	0	4.29	1.98
2	-5	0	0.76	1.44
-2	-5	0	3.02	1.99
-2	5	0	0.40	1.46
2	5	0	1.96	2.01
-3	-5	0	94.78	6.43
3	-5	0	88.78	4.96
3	5	0	86.43	5.91
4	-5	0	62.78	4.02
-4	-5	0	50.49	5.63
4	5	0	55.93	5.91
-5	-5	0	42.19	5.46
5	-5	0	28.26	2.87
5	5	0	48.47	5.66
-6	-5	0	24.60	4.44
6	5	0	23.40	4.38
-7	-5	0	53.06	6.29
7	5	0	63.52	6.95
-8	-5	0	-0.51	1.89
-8	-5	0	-0.37	2.15
8	5	0	-0.11	2.13
-9	-5	0	123.97	8.85
-9	-5	0	121.53	8.87
9	5	0	133.68	8.96
-10	-5	0	24.22	5.02
-10	-5	0	30.79	5.80
10	5	0	26.93	5.41
-11	-5	0	4.29	3.24
-11	-5	0	3.91	3.21
11	5	0	3.04	3.17
0	-6	0	3.63	2.22
0	6	0	7.36	2.63
1	-6	0	2.03	2.10
-1	-6	0	0.31	1.97
1	6	0	4.56	2.49
-2	-6	0	47.13	5.77
2	6	0	48.64	5.64
-3	-6	0	29.70	4.95
-3	-6	0	27.80	4.71
3	6	0	35.86	5.11
-4	-6	0	14.58	3.85
-4	-6	0	19.28	4.39
4	6	0	19.05	4.11
-5	-6	0	24.44	4.91
-5	-6	0	30.86	5.29
5	6	0	17.01	4.11
-6	-6	0	6.16	3.19
-6	-6	0	6.41	3.31

6	6	0	7.24	3.30
-7	-6	0	2.95	2.55
-7	-6	0	-0.09	2.31
7	6	0	2.04	2.82
17	0	-1	0.48	2.27
-17	0	1	0.93	2.37
16	0	-1	-1.88	1.77
-16	0	1	1.23	1.99
15	0	-1	0.34	1.90
-15	0	1	-0.35	1.82
14	0	-1	0.08	1.69
-14	0	1	1.10	1.76
13	0	-1	0.48	1.77
-13	0	1	1.30	1.77
-12	0	1	-2.49	1.21
12	0	-1	0.56	1.53
11	0	-1	-1.54	1.37
-11	0	1	-1.03	1.15
11	0	-1	-1.66	1.06
-11	0	1	-0.79	1.20
-10	0	1	0.96	1.17
10	0	-1	-0.91	1.20
10	0	-1	-1.68	0.87
-10	0	1	0.96	1.17
-9	0	1	-0.63	1.02
9	0	-1	0.09	0.94
-9	0	1	-0.95	0.89
9	0	-1	0.14	1.09
-8	0	1	-0.30	0.90
-8	0	1	-0.26	0.96
8	0	-1	0.56	0.91
8	0	-1	0.10	1.00
-7	0	1	-0.17	0.81
7	0	-1	-1.30	0.60
-7	0	1	-0.37	0.70
7	0	-1	0.12	0.84
6	0	-1	0.49	0.87
6	0	-1	0.11	0.73
-6	0	1	-1.59	0.61
-5	0	1	0.42	0.67
5	0	-1	0.19	0.67
5	0	-1	0.25	0.60
-4	0	1	-0.60	0.57
4	0	-1	1.79	0.78
-3	0	1	-0.05	0.49
3	0	-1	0.55	0.55
-2	0	1	1.89	0.52
2	0	-1	-1.16	0.32
-1	0	1	0.02	0.13
1	0	-1	0.07	0.13

0	0	1	-0.04	0.06
0	0	-1	-0.02	0.04
-1	0	-1	-1.84	0.12
1	0	1	-1.80	0.15
-2	0	-1	-1.73	0.64
3	0	1	-0.16	0.67
-3	0	-1	0.56	0.79
3	0	1	-1.64	0.56
4	0	1	-0.26	0.58
4	0	1	0.39	0.69
-4	0	-1	-0.97	0.55
4	0	1	-0.70	0.56
5	0	1	-1.73	0.63
-5	0	-1	-1.24	0.66
5	0	1	-1.30	0.63
6	0	1	-1.20	0.74
-6	0	-1	0.66	0.89
6	0	1	0.47	0.81
7	0	1	-1.83	0.80
7	0	1	0.47	1.03
-7	0	-1	0.74	1.08
8	0	1	1.18	1.00
-8	0	-1	0.84	1.11
8	0	1	-1.67	0.71
9	0	1	-0.19	1.06
-9	0	-1	-0.87	0.76
9	0	1	-0.99	0.80
10	0	1	1.13	1.20
10	0	1	-0.76	1.12
-10	0	-1	-0.27	1.20
11	0	1	-1.53	1.18
-11	0	-1	-0.01	1.25
-12	0	-1	-1.12	1.34
12	0	1	0.90	1.43
13	0	1	-0.12	1.64
-13	0	-1	1.37	2.00
-14	0	-1	-3.18	1.46
14	0	1	-2.00	1.55
15	0	1	-2.01	1.54
-15	0	-1	2.33	2.06
-16	0	-1	-3.15	1.40
16	0	1	-0.78	1.79
17	0	1	-1.62	2.09
-17	0	-1	0.55	2.03
-17	-1	1	7.84	3.23
17	1	-1	3.27	2.63
-16	-1	1	17.67	4.08
16	1	-1	21.56	4.20
-15	-1	1	45.85	5.93
15	-1	-1	34.01	3.91

-15	1	1	40.73	4.54
15	1	-1	36.18	4.97
-14	-1	1	110.88	7.26
14	-1	-1	107.33	5.34
-14	-1	1	105.08	6.75
-14	1	1	116.03	6.05
14	1	-1	110.49	6.91
13	-1	-1	39.43	3.40
-13	-1	1	41.98	4.83
-13	-1	1	39.13	5.09
13	-1	-1	42.17	3.49
13	1	-1	42.15	4.98
-13	1	1	36.27	4.02
12	-1	-1	384.26	9.76
-12	-1	1	363.64	12.53
12	-1	-1	390.59	9.81
-12	-1	1	377.52	12.12
12	1	-1	374.52	11.91
-12	1	1	386.36	10.51
-11	-1	1	543.31	15.13
11	-1	-1	543.03	11.82
-11	-1	1	555.68	14.76
11	-1	-1	558.25	11.90
-11	1	1	555.66	12.69
11	1	-1	565.70	14.59
10	-1	-1	80.62	3.76
-10	-1	1	87.39	5.23
10	-1	-1	79.65	3.78
-10	-1	1	87.36	5.63
-10	1	1	81.05	4.19
10	1	-1	86.27	5.13
9	-1	-1	199.01	5.71
-9	-1	1	199.32	7.64
9	-1	-1	216.42	5.85
9	1	-1	208.07	7.59
9	1	-1	203.15	7.50
-9	1	1	207.92	6.30
8	-1	-1	234.02	5.80
8	-1	-1	239.62	5.88
-8	-1	1	234.20	7.93
8	1	-1	236.25	7.78
-8	1	1	239.66	6.38
8	1	-1	231.03	7.74
-7	-1	1	197.66	6.90
7	-1	-1	194.82	4.79
7	-1	-1	195.94	4.86
7	1	-1	200.88	6.75
-7	1	1	207.25	5.38
7	1	-1	190.20	6.67
6	-1	-1	1160.33	17.00

-6	-1	1	1114.39	20.29
6	1	-1	1141.64	20.05
6	1	-1	1162.93	20.30
-6	1	1	1150.20	17.55
5	-1	-1	298.51	5.32
-5	-1	1	293.97	7.85
5	1	-1	307.09	7.86
-5	1	1	309.23	5.76
5	1	-1	289.42	7.56
-4	-1	1	392.63	8.81
4	-1	-1	389.74	5.87
4	1	-1	376.31	8.63
-4	1	1	391.57	6.20
4	1	-1	394.25	8.56
-3	-1	1	1729.43	26.28
3	1	-1	1727.53	26.19
3	1	-1	1767.98	26.00
-2	-1	1	1872.43	27.25
2	-1	-1	1902.02	25.53
2	1	-1	1884.10	27.20
2	1	-1	1797.97	26.76
1	-1	-1	2780.44	41.65
-1	-1	1	2930.94	39.91
-1	1	1	2912.60	40.18
1	1	-1	2965.33	39.88
0	-1	-1	15.06	1.21
0	-1	1	13.29	1.30
0	1	1	14.33	1.19
0	1	-1	13.68	1.28
1	-1	1	8.70	1.00
-1	-1	-1	10.92	1.29
1	1	1	9.77	1.22
-1	1	-1	10.04	1.03
2	-1	1	2824.34	38.98
2	-1	1	2953.16	39.06
-2	-1	-1	2898.62	40.73
2	1	1	2931.66	40.70
2	1	1	2931.35	40.39
-2	1	-1	2928.42	39.05
-3	-1	-1	280.42	6.85
3	-1	1	276.24	5.35
3	1	1	293.12	6.71
-3	1	-1	280.43	5.40
4	-1	1	70.77	2.53
-4	-1	-1	70.45	3.51
-4	-1	-1	72.27	3.48
4	1	1	76.01	3.49
4	1	1	74.20	3.37
-4	1	-1	72.67	2.50
5	-1	1	30.37	1.81

-5	-1	-1	25.11	2.63
-5	-1	-1	30.28	2.59
5	1	1	30.00	2.49
-5	1	-1	28.99	1.84
5	1	1	32.60	2.91
6	-1	1	518.95	9.64
6	-1	1	498.18	9.77
-6	-1	-1	491.58	11.85
6	-1	1	508.45	9.29
-6	-1	-1	528.36	11.93
-6	1	-1	514.73	9.90
6	1	1	509.16	11.42
6	1	1	529.51	11.80
7	-1	1	97.33	3.67
-7	-1	-1	96.28	4.85
7	-1	1	103.54	3.60
-7	-1	-1	99.44	5.06
7	1	1	97.21	4.80
7	1	1	96.81	4.72
-7	1	-1	99.76	3.91
-8	-1	-1	0.32	1.04
-8	-1	-1	2.17	1.32
8	-1	1	1.19	0.75
8	-1	1	0.35	0.78
8	1	1	-0.09	0.95
8	1	1	-0.31	0.97
-8	1	-1	1.28	0.86
-9	-1	-1	155.91	7.08
-9	-1	-1	148.37	6.68
9	-1	1	167.85	5.54
9	-1	1	154.14	5.19
-9	1	-1	159.18	5.74
9	1	1	150.28	6.60
-10	-1	-1	149.53	7.28
10	-1	1	149.41	5.50
10	-1	1	140.44	5.26
-10	-1	-1	130.89	6.61
10	1	1	151.02	6.87
-10	1	-1	140.71	5.74
11	-1	1	32.98	3.30
-11	-1	-1	33.32	4.16
11	-1	1	34.89	3.32
-11	-1	-1	32.31	4.41
11	1	1	31.13	4.14
-11	1	-1	36.09	3.74
12	-1	1	36.28	3.56
-12	-1	-1	46.27	5.42
12	-1	1	43.77	3.49
-12	1	-1	41.05	4.22
12	1	1	40.06	4.76

-13	-1	-1	-0.10	1.71
13	-1	1	1.28	1.45
-13	1	-1	-1.40	1.13
13	1	1	3.19	2.14
-14	-1	-1	89.61	6.84
14	-1	1	82.05	4.92
-14	1	-1	82.03	5.43
14	1	1	87.60	6.35
-15	-1	-1	1.99	2.30
15	-1	1	7.19	2.22
15	1	1	1.23	2.27
-15	1	-1	0.45	1.65
-16	-1	-1	38.76	5.86
16	-1	1	40.07	4.51
-16	1	-1	40.93	4.90
16	1	1	41.19	5.70
-17	-1	-1	61.20	7.35
17	1	1	50.57	6.48
-17	-2	1	-1.07	2.52
17	2	-1	1.03	2.51
-16	-2	1	12.58	3.95
-16	-2	1	15.31	4.01
16	2	-1	19.64	4.60
-15	-2	1	75.30	8.15
-15	-2	1	92.02	7.15
15	2	-1	74.08	7.43
-14	-2	1	25.52	4.69
-14	-2	1	27.76	4.68
14	2	-1	24.46	4.50
-13	-2	1	2.28	2.36
-13	-2	1	7.83	3.00
13	2	-1	2.64	2.42
-12	-2	1	118.00	7.64
-12	-2	1	107.65	7.11
12	2	-1	105.41	6.85
-12	2	1	109.85	4.39
-11	-2	1	160.49	8.50
11	-2	-1	161.66	4.36
-11	-2	1	162.78	8.23
11	2	-1	151.09	7.88
-11	2	1	159.69	5.00
-10	-2	1	11.03	2.72
10	-2	-1	12.93	1.31
-10	2	1	16.69	1.81
10	2	-1	12.56	2.95
9	-2	-1	36.31	1.66
-9	-2	1	40.06	4.43
-9	2	1	33.36	1.98
9	2	-1	36.50	4.34
-8	-2	1	121.60	6.36

8	-2	-1	130.53	2.83
8	2	-1	143.31	6.60
-8	2	1	136.63	3.44
-7	-2	1	197.36	7.79
7	2	-1	193.92	7.52
-7	2	1	206.80	3.91
-6	-2	1	22.09	2.98
6	2	-1	26.05	3.23
6	2	-1	30.91	3.51
-5	-2	1	111.54	5.32
5	2	-1	115.68	5.19
5	2	-1	114.33	5.28
-4	-2	1	4.82	1.53
4	-2	-1	6.76	0.67
4	2	-1	9.28	1.80
4	2	-1	6.60	1.59
3	-2	-1	0.90	0.51
-3	-2	1	0.22	0.86
3	-2	-1	0.92	0.42
3	2	-1	1.54	0.98
3	2	-1	0.94	0.99
-3	2	1	1.37	0.53
-2	-2	1	69.95	3.58
2	-2	-1	66.60	2.47
-2	2	1	71.18	2.47
2	2	-1	66.94	3.36
2	2	-1	71.00	3.57
1	-2	-1	72.82	2.86
-1	-2	1	77.92	3.55
1	2	-1	78.30	3.38
-1	2	1	75.58	2.84
1	2	-1	69.58	3.34
0	-2	1	72.56	3.22
0	-2	-1	71.44	3.11
0	2	-1	69.67	2.97
0	2	-1	73.10	3.15
0	2	1	66.97	2.99
0	2	1	67.81	2.87
1	-2	1	223.09	5.68
-1	-2	-1	237.89	6.36
1	2	1	235.07	6.03
-1	2	-1	222.09	5.63
-1	2	-1	225.00	5.33
1	2	1	224.05	6.12
-2	-2	-1	4.22	1.28
2	-2	1	2.09	0.86
-2	2	-1	5.12	1.03
2	2	1	4.43	1.29
2	2	1	1.71	1.05
3	-2	1	41.40	2.04

-3	-2	-1	40.69	2.98
3	-2	1	45.97	2.18
3	2	1	31.01	2.64
-3	2	-1	46.34	2.19
3	2	1	42.43	2.91
4	-2	1	463.16	8.55
-4	-2	-1	480.70	11.53
4	-2	1	472.41	8.46
4	2	1	476.26	11.06
4	2	1	482.17	11.33
5	-2	1	73.37	2.69
-5	-2	-1	83.11	4.61
5	-2	1	75.60	2.73
5	2	1	72.22	4.18
5	2	1	80.58	4.45
-6	-2	-1	164.84	6.86
6	-2	1	168.85	4.44
6	-2	1	175.41	4.38
6	2	1	165.70	6.58
-6	2	-1	168.50	4.43
6	2	1	155.72	6.53
7	-2	1	368.33	7.40
-7	-2	-1	359.55	11.19
-7	-2	-1	351.98	10.91
7	-2	1	365.98	7.59
-7	2	-1	378.54	7.73
7	2	1	378.22	10.79
8	-2	1	111.46	3.72
-8	-2	-1	109.31	6.28
8	-2	1	108.41	3.69
-8	-2	-1	109.62	6.10
-8	2	-1	109.77	3.92
8	2	1	120.78	6.24
-9	-2	-1	0.91	1.47
-9	-2	-1	-0.63	1.27
9	-2	1	0.32	0.86
9	-2	1	0.40	0.70
9	2	1	0.02	1.29
-9	2	-1	-0.45	0.71
10	-2	1	107.22	3.83
-10	-2	-1	99.51	6.57
10	-2	1	103.37	4.03
-10	-2	-1	98.65	6.30
10	2	1	112.78	6.55
-10	2	-1	102.53	4.32
11	-2	1	102.12	4.08
11	-2	1	93.08	4.06
-11	-2	-1	110.86	6.93
11	2	1	106.25	6.81
-11	2	-1	102.90	4.68

-12	-2	-1	106.76	7.12
-12	-2	-1	118.27	7.73
12	2	1	114.28	7.17
-12	2	-1	113.11	5.08
-13	-2	-1	16.36	4.00
-13	-2	-1	13.91	3.44
-13	2	-1	17.40	2.68
13	2	1	16.46	3.82
-14	-2	-1	58.71	6.89
14	2	1	62.53	6.79
-15	-2	-1	2.69	2.46
15	2	1	2.50	2.55
-16	-2	-1	-3.57	1.84
16	2	1	-0.77	2.46
-17	-2	-1	1.19	2.79
17	2	1	0.09	2.81
-15	-3	1	29.88	5.45
-15	-3	1	35.68	6.22
15	3	-1	32.92	5.80
-14	-3	1	10.60	3.95
-14	-3	1	14.84	4.01
14	3	-1	14.66	4.39
-13	-3	1	4.39	2.71
-13	-3	1	6.65	3.04
13	3	-1	3.27	2.66
-12	-3	1	28.19	4.98
-12	-3	1	33.17	5.10
12	3	-1	24.79	4.49
-11	-3	1	74.47	6.39
-11	-3	1	74.58	6.56
11	3	-1	74.60	6.21
-10	-3	1	6.61	2.53
10	3	-1	3.93	2.60
-9	-3	1	75.29	5.88
9	3	-1	91.35	6.37
-8	-3	1	205.11	9.26
8	3	-1	247.00	9.79
-7	-3	1	13.25	2.87
7	3	-1	16.33	3.11
-6	-3	1	278.45	9.96
-5	-3	1	32.71	3.84
5	-3	-1	33.99	1.64
5	3	-1	31.41	3.78
4	-3	-1	95.34	2.85
-4	-3	1	92.76	5.30
4	-3	-1	99.42	3.24
4	3	-1	91.24	5.06
-4	3	1	97.92	3.17
3	-3	-1	995.61	16.54
-3	-3	1	1027.97	20.31

-3	3	1	1024.55	15.85
-3	3	1	1016.95	16.39
3	3	-1	1019.14	19.57
3	3	-1	1022.83	20.00
2	-3	-1	13.80	1.78
-2	-3	1	17.47	2.52
-2	3	1	15.99	1.81
-2	3	1	17.73	1.94
2	3	-1	13.57	2.22
2	3	-1	19.24	2.61
-1	-3	1	477.37	11.54
1	-3	-1	476.81	10.41
-1	3	1	488.72	10.34
1	3	-1	484.99	11.39
-1	3	1	485.07	9.93
1	3	-1	470.23	10.95
0	-3	1	887.00	16.98
0	-3	-1	881.89	16.63
0	3	-1	911.74	16.42
0	3	1	907.44	16.53
0	3	-1	901.57	16.81
0	3	1	847.62	15.88
-1	-3	-1	211.71	7.02
1	-3	1	215.63	6.59
1	3	1	210.64	6.84
1	3	1	213.66	6.68
-1	3	-1	207.39	6.38
-1	3	-1	206.60	6.06
2	-3	1	704.88	13.67
-2	-3	-1	715.76	15.42
-2	3	-1	712.54	12.98
2	3	1	729.27	15.21
2	3	1	723.66	14.89
-2	3	-1	725.08	13.63
-3	-3	-1	38.77	3.87
3	-3	1	35.21	2.49
-3	3	-1	29.62	2.35
3	3	1	32.05	3.53
-3	3	-1	36.12	2.28
3	3	1	39.89	3.37
-4	-3	-1	486.88	13.02
4	-3	1	501.78	9.72
4	-3	1	502.23	9.96
4	3	1	482.67	12.47
-4	3	-1	502.55	9.22
-4	3	-1	509.07	9.92
-5	-3	-1	209.31	8.31
5	-3	1	207.55	5.40
5	-3	1	198.07	5.12
6	-3	1	36.94	2.14

-6	-3	-1	43.46	4.62
6	-3	1	40.69	2.23
6	3	1	43.42	4.63
7	-3	1	108.63	3.43
7	-3	1	106.48	3.44
-7	-3	-1	106.34	6.51
7	3	1	102.40	6.09
8	-3	1	29.71	1.88
8	-3	1	26.40	1.88
-8	-3	-1	22.93	3.90
-8	-3	-1	32.05	4.71
8	3	1	29.96	4.39
-9	-3	-1	336.34	12.43
9	-3	1	344.87	6.75
-9	-3	-1	345.85	12.90
9	-3	1	348.56	6.73
9	3	1	329.37	11.95
-10	-3	-1	17.84	3.70
-10	-3	-1	27.56	4.65
10	-3	1	22.62	1.67
10	-3	1	23.19	1.62
10	3	1	24.50	4.29
-11	-3	-1	77.04	6.40
-11	-3	-1	79.06	6.78
11	3	1	65.55	6.83
-12	-3	-1	43.86	6.07
-12	-3	-1	39.27	5.45
12	3	1	51.32	6.37
-13	-3	-1	27.32	5.26
-13	-3	-1	32.98	5.22
13	3	1	37.88	5.94
-14	-3	-1	88.22	7.50
14	3	1	90.97	7.60
-15	-3	-1	14.95	4.40
-15	-3	-1	25.60	5.05
15	3	1	18.16	4.51
-14	-4	1	-2.83	2.22
-14	-4	1	-0.75	2.52
14	4	-1	-0.98	2.64
-13	-4	1	32.68	5.65
-13	-4	1	63.14	8.24
13	4	-1	43.42	6.45
-12	-4	1	27.84	5.14
-12	-4	1	22.69	5.11
12	4	-1	25.11	4.92
-11	-4	1	-1.77	1.92
-11	-4	1	-2.35	1.76
11	4	-1	-1.70	1.98
-10	-4	1	139.56	8.94
10	4	-1	139.11	8.60

-9	-4	1	18.20	3.95
9	4	-1	19.10	4.19
-8	-4	1	7.70	2.99
8	4	-1	7.12	3.12
-7	-4	1	290.79	11.77
7	4	-1	288.65	11.31
6	-4	-1	0.14	0.69
-6	-4	1	0.62	1.83
6	4	-1	0.08	1.87
5	-4	-1	166.99	4.20
-5	-4	1	169.71	8.30
5	-4	-1	172.36	4.94
5	-4	-1	171.21	4.15
5	4	-1	180.18	8.36
4	-4	-1	16.84	2.03
-4	-4	1	18.76	3.38
-4	4	1	15.14	1.92
4	4	-1	19.28	3.45
3	-4	-1	19.77	2.44
-3	-4	1	17.79	3.17
-3	4	1	19.88	2.26
3	4	-1	13.82	2.86
-3	4	1	16.76	2.19
-2	-4	1	330.46	10.72
2	-4	-1	315.65	8.82
-2	4	1	330.78	8.31
2	4	-1	312.96	9.90
-2	4	1	327.12	8.70
1	-4	-1	3.03	1.41
-1	-4	1	0.25	1.28
-1	4	1	3.87	1.39
-1	4	1	5.27	1.64
1	4	-1	0.90	1.37
0	-4	-1	93.10	5.01
0	-4	1	87.27	4.92
0	4	1	89.62	4.85
0	4	1	83.87	4.51
0	4	-1	92.51	4.79
1	-4	1	56.14	3.90
-1	-4	-1	57.15	4.21
-1	4	-1	57.94	3.67
1	4	1	63.80	4.22
-1	4	-1	53.28	3.79
-2	-4	-1	9.09	2.41
2	-4	1	14.71	2.37
2	4	1	16.40	2.93
-2	4	-1	10.54	1.97
-2	4	-1	18.58	2.45
3	-4	1	156.63	5.93
-3	-4	-1	158.54	7.43

-3	4	-1	156.02	5.77
3	4	1	146.73	6.96
-3	4	-1	151.22	5.33
4	-4	1	352.43	8.86
-4	-4	-1	323.60	11.31
-4	4	-1	337.45	8.59
4	4	1	330.49	10.92
-4	4	-1	344.46	7.99
-5	-4	-1	48.33	5.21
5	-4	1	41.01	2.85
5	4	1	40.38	4.74
6	-4	1	149.48	4.93
-6	-4	-1	137.65	7.79
6	4	1	159.23	7.99
-7	-4	-1	399.17	14.07
-7	-4	-1	450.89	14.96
7	-4	1	420.31	8.59
7	4	1	430.60	14.04
-8	-4	-1	197.98	10.10
-8	-4	-1	204.48	10.16
8	-4	1	190.20	4.64
8	4	1	210.22	9.88
-9	-4	-1	102.40	7.58
-9	-4	-1	89.82	7.06
9	4	1	80.56	6.61
-10	-4	-1	37.33	5.52
-10	-4	-1	32.92	5.51
10	4	1	45.39	6.10
-11	-4	-1	-0.26	2.14
-11	-4	-1	2.51	2.45
11	4	1	-0.16	2.21
-12	-4	-1	25.58	5.33
-12	-4	-1	24.26	4.96
12	4	1	34.00	5.83
-13	-4	-1	-1.42	2.33
-13	-4	-1	2.15	2.84
13	4	1	-0.99	2.48
-14	-4	-1	94.42	9.66
-14	-4	-1	88.26	9.84
-11	-5	1	5.23	3.15
-11	-5	1	0.06	2.40
11	5	-1	1.79	2.93
-10	-5	1	0.30	2.29
-10	-5	1	-1.57	2.34
10	5	-1	1.49	2.83
-9	-5	1	28.35	5.24
9	5	-1	40.81	6.04
-8	-5	1	4.35	2.84
8	5	-1	6.65	3.18
-7	-5	1	10.49	3.36

7	5	-1	8.96	3.40
-6	-5	1	-0.30	1.84
6	5	-1	4.33	2.41
-5	-5	1	1.06	2.16
5	5	-1	1.33	2.20
4	-5	-1	0.34	1.26
-4	-5	1	2.49	2.37
4	5	-1	-1.62	1.74
-3	-5	1	7.71	2.60
3	-5	-1	13.27	2.33
3	-5	-1	9.63	2.17
3	5	-1	13.03	3.20
2	-5	-1	16.04	2.76
-2	-5	1	11.92	2.91
2	5	-1	13.68	2.97
1	-5	-1	-0.06	1.39
-1	-5	1	0.90	1.64
1	5	-1	4.75	2.07
-1	5	1	0.08	1.42
0	-5	1	0.91	1.49
0	-5	-1	3.14	1.89
0	5	1	-0.23	1.50
0	5	-1	-0.06	1.58
1	-5	1	-0.82	1.30
-1	-5	-1	-1.64	1.41
1	5	1	-1.53	1.41
-1	5	-1	0.57	1.47
2	-5	1	80.01	5.10
-2	-5	-1	81.37	5.81
2	5	1	69.05	5.14
-2	5	-1	88.78	4.82
-3	-5	-1	11.82	3.01
3	-5	1	13.35	2.57
3	5	1	17.46	3.49
4	-5	1	8.29	2.03
-4	-5	-1	4.63	2.35
4	5	1	7.96	2.80
5	-5	1	69.07	4.09
-5	-5	-1	56.61	6.04
-5	-5	-1	75.33	6.35
5	5	1	52.60	5.87
-6	-5	-1	0.97	2.13
-6	-5	-1	5.26	2.77
6	5	1	-1.12	1.85
-7	-5	-1	0.26	2.15
-7	-5	-1	5.31	2.74
7	5	1	1.36	2.28
-8	-5	-1	1.77	2.22
-8	-5	-1	3.97	2.58
8	5	1	0.49	2.29

-9	-5	-1	-0.41	2.15
-9	-5	-1	0.63	2.57
9	5	1	0.99	2.58
-10	-5	-1	9.25	3.78
-10	-5	-1	10.19	3.70
10	5	1	10.16	3.98
-11	-5	-1	0.64	2.59
-11	-5	-1	-2.81	2.24
11	5	1	0.77	2.68
-7	-6	1	37.56	6.21
-7	-6	1	41.02	6.22
7	6	-1	45.13	6.54
-6	-6	1	1.53	2.65
6	6	-1	2.39	2.67
-5	-6	1	15.80	3.95
5	6	-1	14.91	3.80
-4	-6	1	-0.83	2.17
4	6	-1	-0.63	2.18
-3	-6	1	2.82	2.68
3	6	-1	-0.71	2.11
-2	-6	1	-1.74	1.70
2	6	-1	0.07	2.20
1	-6	-1	0.21	1.66
1	-6	-1	2.19	2.06
-1	-6	1	1.39	2.08
1	6	-1	-1.84	1.77
0	-6	1	24.50	4.13
0	-6	-1	27.92	4.25
0	-6	-1	28.80	4.26
0	6	-1	29.21	4.18
1	-6	1	49.28	5.36
-1	-6	-1	53.25	5.84
-1	-6	-1	34.29	4.85
1	6	1	50.88	5.54
2	-6	1	15.61	3.28
-2	-6	-1	17.24	3.70
-2	-6	-1	24.60	4.48
2	6	1	22.60	4.08
-3	-6	-1	72.13	7.18
-3	-6	-1	66.31	6.97
3	6	1	73.49	6.10
-4	-6	-1	92.37	7.42
-4	-6	-1	107.97	7.73
4	6	1	106.27	7.39
-5	-6	-1	8.66	3.26
-5	-6	-1	8.06	3.20
5	6	1	6.49	3.11
-6	-6	-1	15.37	4.15
-6	-6	-1	16.60	4.33
6	6	1	17.34	4.15

-7	-6	-1	49.41	6.74
-7	-6	-1	49.21	6.83
7	6	1	47.27	6.57
17	0	-2	209.37	9.36
-17	0	2	186.42	9.61
16	0	-2	35.03	4.71
-16	0	2	35.44	4.90
15	0	-2	73.01	5.29
-15	0	2	71.17	5.60
14	0	-2	29.72	3.98
-14	0	2	46.81	5.20
13	0	-2	24.34	3.65
-13	0	2	33.13	4.23
-12	0	2	221.72	8.07
12	0	-2	224.55	8.09
-12	0	2	211.95	8.37
-11	0	2	-0.20	1.28
11	0	-2	-1.30	1.11
-11	0	2	2.85	1.46
11	0	-2	0.22	1.55
-10	0	2	78.85	4.41
10	0	-2	74.36	4.30
-10	0	2	76.71	4.63
10	0	-2	82.68	4.45
9	0	-2	477.91	10.90
-9	0	2	447.76	10.83
9	0	-2	463.67	10.77
-9	0	2	446.89	11.26
8	0	-2	881.51	16.19
-8	0	2	873.39	16.32
8	0	-2	879.01	16.17
7	0	-2	1.45	0.96
-7	0	2	3.57	1.08
7	0	-2	1.25	1.04
-6	0	2	143.42	4.58
6	0	-2	132.32	4.39
6	0	-2	137.65	4.46
-5	0	2	303.73	6.78
5	0	-2	302.79	6.70
5	0	-2	307.09	6.70
4	0	-2	959.71	14.96
-4	0	2	958.53	15.02
-3	0	2	1532.32	21.68
3	0	-2	1505.06	21.61
2	0	-2	-0.42	0.50
-2	0	2	-0.57	0.50
-1	0	2	22.20	1.12
1	0	-2	19.75	1.08
1	0	2	444.45	7.08
-1	0	-2	336.55	6.72

2	0	2	1205.02	17.83
-2	0	-2	1184.26	17.85
-3	0	-2	-0.63	0.59
3	0	2	0.31	0.67
-4	0	-2	3307.79	47.18
4	0	2	3361.56	46.89
4	0	2	3425.45	46.63
4	0	2	3383.94	47.09
-5	0	-2	2538.21	37.43
5	0	2	2527.49	36.56
5	0	2	2549.55	36.97
6	0	2	1014.29	17.55
6	0	2	1016.19	17.23
-6	0	-2	944.11	17.73
7	0	2	3170.60	46.46
7	0	2	3212.47	46.95
-7	0	-2	3236.53	47.72
-8	0	-2	382.15	10.37
8	0	2	403.85	10.07
8	0	2	390.25	9.75
-9	0	-2	36.44	3.84
9	0	2	32.39	3.34
9	0	2	28.15	3.15
10	0	2	719.48	15.74
10	0	2	720.92	15.88
-10	0	-2	755.10	16.98
11	0	2	230.75	8.30
-11	0	-2	232.39	8.93
-12	0	-2	1.56	1.52
12	0	2	1.02	1.51
-13	0	-2	571.54	15.89
13	0	2	565.95	14.85
-14	0	-2	100.47	6.67
14	0	2	107.20	6.33
15	0	2	1.51	1.83
-15	0	-2	2.19	2.16
16	0	2	76.56	5.84
-16	0	-2	88.35	6.78
17	0	2	-1.26	2.10
-17	0	-2	0.81	2.16
-17	-1	2	38.49	5.92
17	1	-2	45.49	5.84
-16	-1	2	17.62	3.99
16	1	-2	20.96	4.12
-15	-1	2	4.42	2.74
-15	-1	2	2.03	2.08
15	1	-2	4.11	2.65
-15	1	2	0.78	1.76
14	-1	-2	8.62	2.29
-14	-1	2	19.19	3.85

-14	-1	2	16.39	3.45
14	1	-2	12.93	3.26
-14	1	2	11.34	2.54
13	-1	-2	102.68	4.97
-13	-1	2	103.15	6.34
-13	-1	2	104.92	6.71
13	-1	-2	87.72	4.60
-13	1	2	95.15	5.27
13	1	-2	97.27	6.19
12	-1	-2	-0.93	1.13
12	-1	-2	-3.05	1.20
-12	-1	2	-0.58	1.66
-12	-1	2	-0.51	1.55
-12	1	2	-2.18	1.12
12	1	-2	1.06	1.91
-11	-1	2	453.81	13.45
-11	-1	2	431.41	12.75
11	-1	-2	444.99	10.05
11	-1	-2	441.11	10.08
11	1	-2	426.36	12.40
-11	1	2	437.38	10.81
-10	-1	2	116.26	6.03
10	-1	-2	98.26	4.11
10	-1	-2	106.91	4.16
10	1	-2	102.51	5.59
-10	1	2	100.97	4.49
9	-1	-2	163.07	4.96
9	-1	-2	159.04	4.84
-9	-1	2	158.37	6.73
9	1	-2	173.47	6.86
-9	1	2	164.36	5.34
9	1	-2	161.08	6.63
8	-1	-2	110.62	3.69
8	-1	-2	110.64	3.61
-8	-1	2	108.74	5.29
8	1	-2	109.21	5.22
8	1	-2	102.90	5.17
-8	1	2	103.45	3.89
-7	-1	2	598.20	13.32
7	-1	-2	612.64	10.23
7	1	-2	586.39	13.11
-7	1	2	596.75	10.77
7	1	-2	618.13	13.26
6	-1	-2	1107.79	16.08
-6	-1	2	1098.56	19.85
6	1	-2	1104.79	19.48
-6	1	2	1101.34	16.71
6	1	-2	1100.23	19.66
-5	-1	2	2162.58	33.29
5	-1	-2	2197.69	29.12

-5	1	2	2188.09	29.71
5	1	-2	2162.92	33.10
5	1	-2	2163.48	32.93
-4	-1	2	391.80	8.95
4	1	-2	389.27	8.62
-4	1	2	404.30	6.12
4	1	-2	392.04	8.84
-3	-1	2	0.53	0.73
3	1	-2	0.08	0.68
3	1	-2	1.52	0.81
2	-1	-2	5.98	0.49
-2	-1	2	5.33	1.10
-2	1	2	7.14	0.55
2	1	-2	4.35	1.01
-2	1	2	5.40	0.45
2	1	-2	8.18	1.23
-1	-1	2	327.90	6.92
1	-1	-2	327.99	5.55
1	1	-2	336.62	6.91
-1	1	2	341.98	5.71
-1	1	2	333.52	5.57
1	1	-2	339.35	6.70
0	-1	2	585.40	10.06
0	-1	-2	577.19	9.46
0	-1	-2	582.53	9.54
0	1	-2	574.39	9.95
0	1	2	581.97	9.55
0	1	2	576.69	9.44
1	-1	2	237.22	5.15
-1	-1	-2	230.26	5.37
1	-1	2	242.11	5.19
-1	-1	-2	238.74	5.45
1	1	2	235.42	5.40
1	1	2	240.55	5.41
-1	1	-2	235.39	5.15
-2	-1	-2	52.55	2.67
2	-1	2	56.42	2.25
2	-1	2	56.02	2.23
-2	-1	-2	53.75	2.66
2	1	2	52.00	2.48
-2	1	-2	57.99	2.30
2	1	2	54.90	2.63
-3	-1	-2	164.69	5.13
3	-1	2	168.16	4.26
3	1	2	159.86	4.82
-3	1	-2	160.29	4.19
-4	-1	-2	460.19	10.32
4	-1	2	466.40	8.76
-4	-1	-2	463.15	10.19
4	1	2	460.97	10.11

4	1	2	452.90	9.79
-4	1	-2	455.65	8.78
-5	-1	-2	3.74	1.27
5	-1	2	7.91	1.29
-5	-1	-2	8.22	1.66
5	-1	2	6.64	1.13
5	1	2	7.46	1.56
-5	1	-2	5.61	1.17
5	1	2	4.59	1.37
6	-1	2	956.65	15.61
-6	-1	-2	938.62	18.47
-6	-1	-2	957.27	18.25
-6	1	-2	934.55	16.29
6	1	2	944.71	17.74
6	1	2	965.19	18.12
7	-1	2	190.52	5.62
-7	-1	-2	190.87	7.22
7	-1	2	198.66	5.47
-7	-1	-2	195.46	7.02
7	1	2	195.85	6.95
7	1	2	175.36	6.55
-7	1	-2	180.71	5.80
8	-1	2	15.84	2.03
-8	-1	-2	15.02	2.67
8	-1	2	17.71	2.13
-8	-1	-2	14.83	2.48
8	1	2	17.22	2.60
8	1	2	19.29	2.78
-8	1	-2	18.66	2.36
9	-1	2	239.23	7.12
9	-1	2	237.14	6.87
-9	-1	-2	232.21	8.81
-9	-1	-2	240.92	8.59
-9	1	-2	231.23	7.36
9	1	2	239.99	8.49
10	-1	2	44.72	3.16
-10	-1	-2	42.96	4.73
10	-1	2	42.52	3.31
10	1	2	50.57	4.19
-10	1	-2	38.35	3.33
-11	-1	-2	8.69	2.72
11	-1	2	10.97	2.20
11	-1	2	12.58	2.21
-11	1	-2	10.33	2.24
11	1	2	3.06	2.11
12	-1	2	30.52	3.42
12	-1	2	22.69	3.05
-12	-1	-2	23.02	4.12
-12	1	-2	41.41	4.35
12	1	2	25.61	4.01

-13	-1	-2	82.22	6.27
13	-1	2	84.30	4.87
-13	1	-2	83.84	5.45
13	1	2	80.89	5.84
14	-1	2	27.30	3.60
-14	-1	-2	26.29	4.67
-14	1	-2	24.78	3.74
14	1	2	23.60	4.14
15	-1	2	22.18	3.50
-15	-1	-2	18.70	4.10
15	1	2	22.02	4.29
-15	1	-2	25.70	3.94
16	-1	2	5.62	2.47
-16	-1	-2	6.04	3.17
-16	1	-2	4.02	2.33
16	1	2	9.78	3.32
-17	-1	-2	43.37	6.40
17	1	2	44.55	6.17
-17	-2	2	61.35	7.15
-17	-2	2	65.65	7.89
17	2	-2	62.34	7.42
-16	-2	2	1.80	2.73
-16	-2	2	2.21	2.63
16	2	-2	2.04	2.65
-15	-2	2	10.83	3.62
-15	-2	2	4.45	2.86
15	2	-2	3.77	2.62
-14	-2	2	4.93	2.56
-14	-2	2	13.11	3.54
14	2	-2	9.67	3.31
-13	-2	2	54.60	6.46
-13	-2	2	53.19	5.86
13	2	-2	53.66	6.02
-12	-2	2	164.11	8.94
-12	-2	2	184.59	9.08
12	2	-2	167.45	8.63
-11	-2	2	6.74	2.51
11	2	-2	12.05	3.04
-11	2	2	9.92	1.48
-10	-2	2	25.94	3.81
10	2	-2	20.28	3.44
-10	2	2	25.55	1.78
-9	-2	2	121.98	6.60
-9	2	2	123.97	3.24
9	2	-2	118.90	6.37
-8	-2	2	124.81	6.37
8	2	-2	130.60	6.34
-7	-2	2	113.63	5.89
7	2	-2	110.18	5.61
-6	-2	2	124.73	5.88

6	2	-2	125.84	5.81
-5	-2	2	631.02	14.22
5	2	-2	607.45	13.60
5	2	-2	626.75	13.95
-4	-2	2	83.86	4.38
4	2	-2	85.48	4.36
-3	-2	2	82.27	4.15
3	-2	-2	87.74	2.27
3	-2	-2	84.72	1.89
-3	2	2	84.28	2.21
3	2	-2	85.50	4.13
-2	-2	2	125.94	4.89
2	-2	-2	122.32	3.16
2	-2	-2	126.51	3.41
-2	2	2	130.27	3.37
2	2	-2	128.00	4.66
2	2	-2	129.21	4.89
-1	-2	2	562.16	11.58
1	-2	-2	567.64	9.96
1	-2	-2	573.77	10.26
1	2	-2	577.06	11.56
-1	2	2	577.46	10.10
1	2	-2	554.97	11.06
0	-2	-2	1357.92	21.28
0	-2	2	1377.05	22.09
0	-2	-2	1378.84	21.54
0	2	2	1383.58	21.36
0	2	-2	1364.97	21.92
0	2	-2	1388.77	21.48
0	2	2	1394.02	21.04
-1	-2	-2	60.47	3.14
-1	-2	-2	64.91	3.19
1	-2	2	72.29	3.20
1	2	2	60.57	3.13
-1	2	-2	64.92	2.84
1	2	2	61.65	3.04
-1	2	-2	59.38	2.94
2	-2	2	252.71	6.17
-2	-2	-2	253.32	7.15
-2	-2	-2	256.58	7.21
2	-2	2	252.43	6.24
2	2	2	266.90	7.19
-2	2	-2	252.24	6.19
-2	2	-2	248.46	5.80
2	2	2	257.49	6.85
-3	-2	-2	151.57	5.77
3	-2	2	153.54	4.55
3	-2	2	161.18	4.60
-3	-2	-2	166.48	5.95
3	2	2	159.83	5.72

-3	2	-2	162.80	4.61
-3	2	-2	166.90	4.31
3	2	2	160.64	5.57
4	-2	2	287.45	6.50
-4	-2	-2	276.25	8.45
4	-2	2	290.09	6.59
-4	-2	-2	280.16	8.46
-4	2	-2	284.60	6.07
4	2	2	301.80	8.51
4	2	2	290.22	8.20
5	-2	2	0.50	0.65
-5	-2	-2	0.37	1.13
5	-2	2	1.12	0.79
-5	-2	-2	4.25	1.55
5	2	2	1.07	1.23
-5	2	-2	-0.04	0.63
5	2	2	0.99	1.04
6	-2	2	67.25	2.98
6	-2	2	62.84	2.88
-6	-2	-2	60.44	4.36
-6	-2	-2	61.22	4.24
6	2	2	64.19	4.25
-6	2	-2	61.25	2.88
6	2	2	66.69	4.25
7	-2	2	77.78	3.32
-7	-2	-2	78.57	5.04
-7	-2	-2	92.46	5.38
7	2	2	69.08	4.64
-7	2	-2	83.26	3.54
8	-2	2	373.25	7.84
8	-2	2	367.61	8.25
-8	-2	-2	360.55	11.84
8	2	2	362.56	11.15
-8	2	-2	360.01	8.49
-9	-2	-2	99.16	6.33
9	-2	2	95.04	3.98
-9	-2	-2	99.85	6.13
9	-2	2	98.77	3.82
-9	2	-2	93.80	4.17
9	2	2	99.07	5.97
10	-2	2	35.79	2.64
-10	-2	-2	41.71	4.80
-10	-2	-2	34.91	4.78
10	-2	2	32.12	2.70
10	2	2	42.36	5.03
-10	2	-2	32.53	3.17
11	-2	2	715.18	13.74
-11	-2	-2	730.45	19.72
11	-2	2	721.94	14.30
-11	-2	-2	728.92	19.14

11	2	2	713.70	18.63
-11	2	-2	738.03	15.08
12	-2	2	2.18	1.34
-12	-2	-2	1.84	1.98
-12	-2	-2	1.98	2.00
12	2	2	2.40	2.29
-12	2	-2	1.14	1.31
-13	-2	-2	60.18	6.96
13	2	2	53.51	6.32
-13	2	-2	44.33	3.70
-14	-2	-2	47.96	6.48
14	2	2	54.94	6.61
-15	-2	-2	1.80	2.35
15	2	2	1.85	2.53
-16	-2	-2	12.88	4.01
16	2	2	20.77	4.61
-15	-3	2	66.29	7.82
-15	-3	2	48.42	6.83
15	3	-2	60.51	7.33
-14	-3	2	1.40	2.56
-14	-3	2	-0.04	2.17
14	3	-2	3.30	2.79
-13	-3	2	17.60	4.26
-13	-3	2	12.04	3.54
13	3	-2	17.96	4.35
-12	-3	2	24.83	4.58
12	3	-2	25.53	4.62
-11	-3	2	-3.66	1.31
11	3	-2	0.34	2.20
-10	-3	2	-2.21	1.41
10	3	-2	1.61	2.21
-9	-3	2	8.37	2.77
9	3	-2	11.50	2.99
-8	-3	2	397.09	13.07
8	3	-2	384.06	12.60
-7	-3	2	22.40	3.55
7	3	-2	24.64	3.60
-6	-3	2	44.47	4.60
6	3	-2	54.29	4.49
-5	-3	2	310.19	10.27
4	-3	-2	162.51	4.04
4	-3	-2	156.39	3.37
-4	-3	2	175.02	7.27
4	-3	-2	162.71	3.38
4	3	-2	176.01	7.02
-4	3	2	163.99	3.97
-3	-3	2	41.96	4.08
3	-3	-2	42.50	2.33
3	-3	-2	41.54	2.14
3	3	-2	40.86	3.96

-3	3	2	35.81	2.19
3	3	-2	48.67	4.37
2	-3	-2	14.64	1.81
2	-3	-2	18.10	1.86
-2	-3	2	17.07	2.69
-2	3	2	17.33	1.85
-2	3	2	14.51	1.66
2	3	-2	13.09	2.21
2	3	-2	14.87	2.37
-1	-3	2	1.85	1.29
1	-3	-2	2.16	1.14
1	3	-2	3.77	1.40
1	3	-2	5.72	1.61
-1	3	2	2.74	1.17
-1	3	2	5.03	1.34
0	-3	-2	2.65	1.31
0	-3	2	3.54	1.37
0	3	2	3.62	1.30
0	3	-2	5.58	1.48
0	3	-2	2.43	1.25
0	3	2	3.84	1.38
-1	-3	-2	2.51	1.30
1	-3	2	6.48	1.63
-1	3	-2	5.99	1.47
1	3	2	4.13	1.39
-1	3	-2	3.44	1.33
1	3	2	6.37	1.63
-2	-3	-2	267.47	8.36
2	-3	2	255.67	7.23
2	3	2	255.24	8.05
2	3	2	243.95	7.70
-2	3	-2	246.20	7.08
-2	3	-2	250.24	6.70
3	-3	2	9.25	1.64
-3	-3	-2	10.64	2.23
-3	3	-2	8.75	1.55
3	3	2	6.81	1.81
3	3	2	11.25	2.19
-3	3	-2	10.01	1.57
-4	-3	-2	-1.86	1.03
4	-3	2	-0.50	0.77
-4	-3	-2	0.85	1.35
-4	3	-2	-0.32	0.76
-4	3	-2	-0.07	0.78
4	3	2	-0.69	1.24
5	-3	2	33.43	2.41
-5	-3	-2	30.16	3.91
-5	-3	-2	24.88	3.53
5	3	2	30.84	3.78
-5	3	-2	34.48	2.25

-6	-3	-2	10.92	2.65
6	-3	2	11.08	1.60
-6	-3	-2	14.14	2.76
-6	3	-2	11.53	1.48
6	3	2	13.17	2.82
-7	-3	-2	42.31	5.12
7	-3	2	44.75	2.62
-7	-3	-2	33.49	4.31
7	3	2	36.83	4.51
-8	-3	-2	475.64	14.73
8	-3	2	487.42	9.67
-8	-3	-2	460.16	14.74
8	3	2	470.54	14.14
9	-3	2	38.87	2.55
-9	-3	-2	30.41	4.44
-9	-3	-2	37.21	5.26
9	3	2	43.36	5.30
-10	-3	-2	21.21	4.27
10	-3	2	21.27	2.12
10	-3	2	17.72	1.98
-10	-3	-2	21.58	3.95
10	3	2	11.62	3.40
-11	-3	-2	17.27	4.02
11	-3	2	16.33	1.89
-11	-3	-2	6.98	3.28
11	3	2	11.79	3.44
-12	-3	-2	48.71	6.59
-12	-3	-2	38.97	5.53
12	3	2	42.92	5.84
-13	-3	-2	13.37	3.70
-13	-3	-2	10.11	3.67
13	3	2	9.16	3.64
-14	-3	-2	20.44	4.83
14	3	2	37.60	6.00
-15	-3	-2	10.26	3.92
15	3	2	8.30	3.54
-14	-4	2	26.33	5.70
-14	-4	2	15.51	4.48
14	4	-2	25.72	5.49
-13	-4	2	2.87	2.78
-13	-4	2	3.07	2.97
13	4	-2	1.47	2.62
-12	-4	2	6.95	3.28
-12	-4	2	8.03	3.26
12	4	-2	3.06	2.69
-11	-4	2	4.93	2.75
11	4	-2	7.73	3.27
-10	-4	2	17.64	4.13
10	4	-2	22.11	4.42
-9	-4	2	10.74	3.43

9	4	-2	3.19	2.50
-8	-4	2	215.87	10.41
8	4	-2	203.40	9.78
-7	-4	2	29.39	4.61
7	4	-2	24.43	4.27
-6	-4	2	90.76	6.40
6	4	-2	100.40	6.50
-5	-4	2	32.34	4.37
-5	4	2	30.80	1.99
5	4	-2	39.09	4.83
4	-4	-2	13.93	1.64
4	-4	-2	11.21	1.69
-4	-4	2	13.49	3.09
4	-4	-2	13.65	1.57
-4	4	2	13.28	1.74
4	4	-2	12.46	3.08
3	-4	-2	111.34	4.31
3	-4	-2	111.27	4.57
-3	-4	2	113.86	6.40
3	4	-2	111.37	6.09
-3	4	2	113.63	4.50
-3	4	2	115.65	4.20
2	-4	-2	311.83	8.60
2	-4	-2	316.98	8.22
-2	-4	2	313.13	10.53
2	4	-2	292.72	9.64
-2	4	2	305.36	8.27
-2	4	2	310.51	7.95
1	-4	-2	95.76	4.71
-1	-4	2	107.39	5.84
1	-4	-2	109.15	5.18
-1	4	2	112.13	5.01
1	4	-2	95.46	5.27
-1	4	2	102.37	4.66
0	-4	-2	62.51	4.12
0	-4	-2	67.18	4.31
0	-4	2	65.58	4.48
0	4	2	69.34	4.08
0	4	-2	64.84	4.25
0	4	2	70.42	4.25
-1	-4	-2	16.82	2.75
1	-4	2	14.00	2.50
-1	4	-2	14.68	2.44
1	4	2	8.58	2.15
-1	4	-2	15.57	2.51
2	-4	2	74.40	4.39
-2	-4	-2	66.81	4.67
-2	-4	-2	70.26	4.72
-2	4	-2	67.70	3.94
-2	4	-2	63.62	4.05

2	4	2	69.95	4.68
-3	-4	-2	29.03	3.88
-3	-4	-2	43.43	4.85
3	-4	2	36.17	3.48
3	4	2	33.21	4.12
-3	4	-2	33.87	3.28
-3	4	-2	33.96	2.83
-4	-4	-2	31.61	4.33
-4	-4	-2	27.33	4.00
4	-4	2	25.72	2.84
-4	4	-2	29.01	3.01
4	4	2	31.28	4.08
-4	4	-2	32.78	2.96
5	-4	2	56.71	3.54
-5	-4	-2	67.83	5.42
-5	-4	-2	66.98	5.39
5	4	2	68.29	5.39
-6	-4	-2	29.54	4.35
6	-4	2	27.47	2.69
-6	-4	-2	24.85	4.23
6	4	2	18.21	3.59
-7	-4	-2	0.48	1.93
7	-4	2	0.42	1.00
-7	-4	-2	-2.04	1.47
7	4	2	-0.51	1.87
8	-4	2	179.22	5.29
-8	-4	-2	187.50	9.85
-8	-4	-2	185.08	9.76
8	4	2	176.26	9.32
-9	-4	-2	23.24	4.78
-9	-4	-2	20.32	4.22
9	4	2	26.96	4.86
-10	-4	-2	4.49	2.91
-10	-4	-2	3.08	2.58
10	4	2	-0.16	2.19
-11	-4	-2	31.65	5.67
-11	-4	-2	15.95	4.15
11	4	2	18.94	4.27
-12	-4	-2	0.39	2.28
-12	-4	-2	0.29	2.46
12	4	2	1.74	2.67
-13	-4	-2	17.52	4.29
-13	-4	-2	15.90	4.56
13	4	2	20.07	4.86
-14	-4	-2	3.05	3.22
-14	-4	-2	5.70	3.34
14	4	2	-0.89	2.76
-11	-5	2	5.25	3.36
-11	-5	2	11.03	3.92
11	5	-2	14.56	4.38

-10	-5	2	5.01	3.07
10	5	-2	4.37	3.19
-9	-5	2	11.89	3.83
9	5	-2	15.51	4.42
-8	-5	2	122.28	8.57
8	5	-2	111.64	8.03
-7	-5	2	2.10	2.27
7	5	-2	2.12	2.26
-6	-5	2	56.77	6.31
6	5	-2	64.79	6.77
-5	-5	2	141.89	8.54
5	5	-2	144.67	8.23
-4	-5	2	118.56	7.45
4	-5	-2	119.46	5.00
4	5	-2	126.82	7.44
-3	-5	2	-0.82	1.67
3	-5	-2	1.86	1.37
3	-5	-2	2.93	1.51
3	5	-2	0.58	1.97
2	-5	-2	191.38	7.40
2	-5	-2	199.26	7.23
-2	-5	2	198.25	9.12
2	5	-2	199.34	8.68
-1	-5	2	20.94	3.62
1	-5	-2	23.77	3.32
1	-5	-2	21.82	3.26
1	5	-2	15.49	3.02
-1	5	2	24.44	3.17
0	-5	2	0.34	1.68
0	-5	-2	-1.51	1.22
0	-5	-2	0.93	1.57
0	5	2	-1.16	1.29
0	5	-2	-0.29	1.59
1	-5	2	75.46	5.23
-1	-5	-2	86.91	5.63
-1	-5	-2	81.39	5.57
1	5	2	86.86	5.37
-1	5	-2	73.03	4.78
-2	-5	-2	11.58	2.90
-2	-5	-2	7.62	2.62
2	-5	2	19.29	3.14
-2	5	-2	12.95	2.61
2	5	2	6.82	2.37
-3	-5	-2	50.13	5.53
3	-5	2	48.37	4.07
-3	-5	-2	55.21	6.11
3	5	2	51.04	5.46
4	-5	2	1.38	1.49
-4	-5	-2	3.69	2.19
-4	-5	-2	2.74	2.00

4	5	2	2.01	2.10
-5	-5	-2	-0.40	1.77
-5	-5	-2	0.64	2.00
5	-5	2	-0.51	1.21
5	5	2	-0.13	1.92
-6	-5	-2	57.55	6.36
-6	-5	-2	54.68	6.52
6	5	2	49.35	5.88
-7	-5	-2	24.67	4.75
-7	-5	-2	22.53	4.41
7	5	2	17.29	4.01
-8	-5	-2	4.00	2.90
-8	-5	-2	-0.37	1.84
8	5	2	-0.62	2.30
-9	-5	-2	23.80	4.98
-9	-5	-2	16.41	4.03
9	5	2	31.51	5.62
-10	-5	-2	3.23	3.01
-10	-5	-2	4.45	3.28
10	5	2	3.40	3.11
-11	-5	-2	13.28	4.04
-11	-5	-2	8.24	3.97
11	5	2	7.50	3.61
-7	-6	2	14.70	4.03
7	6	-2	10.08	3.73
-6	-6	2	9.72	3.58
6	6	-2	6.40	3.36
-5	-6	2	-0.26	2.39
5	6	-2	3.24	2.60
-4	-6	2	9.91	3.40
4	6	-2	11.75	3.55
-3	-6	2	3.36	2.62
3	6	-2	1.56	2.33
-2	-6	2	1.15	2.23
2	6	-2	1.23	2.21
1	-6	-2	0.27	1.93
-1	-6	2	0.43	2.16
1	6	-2	1.16	1.95
0	-6	2	4.86	2.52
0	-6	-2	3.83	2.29
0	-6	-2	7.27	2.60
0	6	-2	6.57	2.52
-1	-6	-2	0.93	2.08
-1	-6	-2	-1.10	1.62
1	-6	2	0.83	1.91
1	6	2	1.41	1.93
-2	-6	-2	3.27	2.43
2	-6	2	1.89	1.82
-2	-6	-2	-0.22	2.01
2	6	2	3.22	2.16

-3	-6	-2	68.91	7.10
-3	-6	-2	76.62	6.51
3	6	2	66.72	6.62
-4	-6	-2	0.94	2.38
-4	-6	-2	0.87	2.30
4	6	2	-1.33	1.97
-5	-6	-2	11.36	3.69
-5	-6	-2	1.67	2.65
5	6	2	0.52	2.39
-6	-6	-2	0.83	2.38
-6	-6	-2	3.54	2.67
6	6	2	0.84	2.57
-7	-6	-2	10.49	3.92
-7	-6	-2	12.08	3.76
7	6	2	5.94	3.24
-17	0	3	-2.21	1.88
17	0	-3	1.17	2.35
-16	0	3	-0.73	1.66
16	0	-3	2.77	2.25
-15	0	3	1.00	1.64
15	0	-3	0.41	1.76
-14	0	3	-1.63	1.41
14	0	-3	-1.69	1.54
13	0	-3	-2.14	1.75
-13	0	3	-2.77	1.42
-13	0	3	-1.17	1.49
-12	0	3	-0.78	1.59
12	0	-3	-2.64	1.50
-12	0	3	-2.40	1.28
-11	0	3	2.08	1.48
11	0	-3	-0.39	1.59
11	0	-3	-2.77	1.09
-11	0	3	0.15	1.36
10	0	-3	-0.57	1.16
10	0	-3	-0.82	1.27
-10	0	3	-1.32	1.25
9	0	-3	0.34	1.14
-9	0	3	-1.55	0.93
9	0	-3	-0.66	0.92
8	0	-3	0.25	1.02
-8	0	3	0.75	1.03
8	0	-3	1.10	0.93
7	0	-3	-0.61	0.81
-7	0	3	-0.30	0.72
7	0	-3	-0.94	0.66
-6	0	3	-0.54	0.64
6	0	-3	-0.36	0.66
6	0	-3	-0.82	0.73
-5	0	3	-1.60	0.59
5	0	-3	-1.30	0.67

5	0	-3	-1.46	0.61
-4	0	3	-0.72	0.53
4	0	-3	0.39	0.63
4	0	-3	0.00	0.53
-3	0	3	0.54	0.60
3	0	-3	-0.15	0.52
2	0	-3	-0.18	0.52
-2	0	3	0.24	0.55
-1	0	3	-0.33	0.51
-1	0	3	-0.24	0.56
1	0	-3	-0.49	0.52
1	0	-3	-0.61	0.54
0	0	-3	-1.19	0.48
0	0	3	-1.03	0.48
-1	0	-3	-1.90	0.49
1	0	3	-0.18	0.63
2	0	3	-1.62	0.55
-2	0	-3	-1.30	0.57
2	0	3	-1.52	0.54
3	0	3	-2.35	0.62
3	0	3	7.59	1.44
-4	0	-3	-0.44	0.58
4	0	3	0.59	0.72
5	0	3	-0.56	0.74
-5	0	-3	-0.57	0.68
5	0	3	-0.01	0.70
6	0	3	-0.88	0.80
6	0	3	0.34	0.90
-6	0	-3	-1.15	0.77
7	0	3	0.58	1.03
7	0	3	-0.76	0.85
-7	0	-3	-0.23	0.96
8	0	3	0.40	1.00
-8	0	-3	0.15	1.05
8	0	3	0.00	0.97
9	0	3	0.90	1.19
-9	0	-3	-0.16	1.07
9	0	3	-0.18	0.90
-10	0	-3	1.61	1.48
10	0	3	1.32	1.39
11	0	3	0.88	1.44
-11	0	-3	0.34	1.32
-12	0	-3	-0.50	1.40
12	0	3	1.13	1.65
-13	0	-3	1.40	1.89
13	0	3	1.41	1.86
14	0	3	3.73	2.12
-14	0	-3	3.02	2.07
-15	0	-3	-1.38	1.61
15	0	3	-0.73	1.92

-16	0	-3	0.98	1.92
16	0	3	-2.31	1.54
17	0	3	0.31	2.18
-17	0	-3	0.12	2.00
-17	-1	3	-1.56	2.13
17	1	-3	1.30	2.51
-16	-1	3	0.93	2.08
-16	-1	3	-0.37	2.02
16	1	-3	1.88	2.25
-15	-1	3	111.03	7.53
-15	-1	3	103.83	6.92
15	1	-3	107.08	6.99
14	-1	-3	57.30	3.94
-14	-1	3	66.31	5.51
-14	-1	3	56.84	6.12
14	1	-3	54.19	5.69
-14	1	3	63.82	4.50
-13	-1	3	2.55	2.09
13	-1	-3	-0.38	1.38
-13	-1	3	-0.29	1.74
13	1	-3	8.41	2.68
-13	1	3	2.80	1.64
12	-1	-3	1.24	1.34
12	-1	-3	2.45	1.60
-12	-1	3	3.97	2.12
-12	1	3	3.22	1.66
12	1	-3	2.63	2.08
11	-1	-3	289.11	7.56
11	-1	-3	288.57	7.71
-11	-1	3	280.86	9.96
-11	1	3	282.91	8.15
11	1	-3	270.85	9.69
10	-1	-3	12.69	1.74
-10	-1	3	11.94	2.51
10	-1	-3	13.62	1.99
10	1	-3	13.00	2.67
-10	1	3	13.00	2.06
-9	-1	3	47.33	3.74
9	-1	-3	36.35	2.47
9	-1	-3	45.67	2.51
-9	1	3	41.71	2.71
9	1	-3	45.22	3.67
9	1	-3	40.51	4.01
-8	-1	3	76.22	4.47
8	-1	-3	73.37	3.01
8	-1	-3	72.97	2.89
8	1	-3	80.28	4.49
-8	1	3	76.91	2.97
8	1	-3	82.34	4.50
-8	1	3	75.43	3.25

7	-1	-3	45.91	2.06
-7	-1	3	44.92	3.32
-7	1	3	45.62	2.07
7	1	-3	43.68	3.37
7	1	-3	46.10	3.36
-6	-1	3	88.26	4.35
6	-1	-3	91.04	2.61
6	1	-3	95.83	4.40
6	1	-3	100.21	4.62
-6	1	3	89.93	2.63
5	-1	-3	18.85	1.08
-5	-1	3	18.99	2.32
5	1	-3	16.35	2.13
-5	1	3	19.11	1.09
5	1	-3	20.65	2.35
-4	-1	3	509.94	10.79
-4	1	3	517.70	7.59
4	1	-3	514.59	10.72
4	1	-3	504.33	10.45
-3	-1	3	16.09	1.83
-3	1	3	20.06	0.96
-3	1	3	17.10	0.87
3	1	-3	15.21	1.77
3	1	-3	18.73	1.94
2	-1	-3	3026.73	41.28
-2	-1	3	3069.49	43.08
2	1	-3	3058.10	42.60
-2	1	3	3035.62	43.32
2	1	-3	3026.49	42.90
-1	-1	3	6106.95	80.71
1	-1	-3	5909.92	86.57
1	1	-3	6012.24	80.17
-1	1	3	5977.72	85.73
1	1	-3	6075.21	80.55
0	-1	3	3372.24	46.65
0	-1	-3	3285.91	45.80
0	1	3	3363.67	45.85
0	1	-3	3435.85	46.22
0	1	-3	3421.86	46.59
1	-1	3	5188.64	68.36
1	-1	3	5115.88	68.26
-1	-1	-3	4826.63	68.27
1	1	3	5095.02	68.04
1	1	3	5069.65	71.70
1	1	3	5065.99	68.33
-1	1	-3	5186.43	68.29
2	-1	3	3756.27	51.76
-2	-1	-3	3789.19	52.77
2	-1	3	3742.05	51.83
2	1	3	3832.05	52.73

2	1	3	3851.25	52.71
-2	1	-3	3784.81	51.91
2	1	3	3789.19	52.25
3	-1	3	371.15	7.38
3	-1	3	374.91	7.76
-3	1	-3	368.96	7.83
3	1	3	373.61	8.30
3	1	3	375.49	8.61
4	-1	3	-1.39	0.57
-4	-1	-3	-0.49	0.83
4	-1	3	-0.35	0.65
4	1	3	0.10	0.82
4	1	3	-0.18	0.86
-4	1	-3	0.01	0.72
5	-1	3	111.16	3.82
-5	-1	-3	113.94	5.00
5	1	3	114.20	4.89
5	1	3	120.23	4.82
-5	1	-3	110.81	4.18
6	-1	3	445.19	9.30
-6	-1	-3	460.01	11.52
6	1	3	455.45	10.89
-6	1	-3	435.65	9.85
7	-1	3	32.34	2.40
-7	-1	-3	30.05	3.55
-7	1	-3	36.27	2.81
7	1	3	34.14	3.60
7	1	3	25.55	3.03
-8	-1	-3	51.73	4.12
8	-1	3	53.21	3.27
8	-1	3	55.78	3.27
8	1	3	50.67	3.86
-8	1	-3	55.29	3.53
9	-1	3	73.05	4.04
9	-1	3	76.77	3.97
-9	-1	-3	74.10	5.26
-9	1	-3	72.03	4.21
9	1	3	73.94	4.88
10	-1	3	363.88	9.63
-10	-1	-3	385.56	12.30
10	-1	3	391.79	10.23
10	1	3	383.54	11.60
-10	1	-3	345.00	10.17
11	-1	3	23.31	3.05
11	-1	3	19.91	2.86
-11	-1	-3	21.89	3.79
-11	1	-3	16.30	2.81
11	1	3	19.27	3.37
12	-1	3	164.43	6.66
-12	-1	-3	169.08	8.63

12	1	3	148.58	7.65
-12	1	-3	154.31	7.06
13	-1	3	129.89	6.24
-13	-1	-3	123.92	7.79
-13	1	-3	136.67	6.91
13	1	3	120.38	7.30
-14	-1	-3	7.77	2.85
14	-1	3	8.65	2.47
14	1	3	6.32	2.70
-14	1	-3	9.88	2.71
-15	-1	-3	105.14	7.69
15	-1	3	113.16	6.24
15	1	3	120.94	7.69
-15	1	-3	124.90	7.17
16	-1	3	11.30	2.94
-16	-1	-3	6.66	3.05
16	1	3	8.51	3.25
-16	1	-3	15.79	3.41
-17	-1	-3	4.13	3.01
17	-1	3	-0.66	2.26
17	1	3	2.39	2.63
-16	-2	3	16.40	4.44
-16	-2	3	15.30	4.05
16	2	-3	11.42	3.69
-15	-2	3	-0.84	2.08
-15	-2	3	5.27	2.97
15	2	-3	0.70	2.38
-14	-2	3	2.53	2.33
-14	-2	3	0.29	2.14
14	2	-3	-1.63	1.92
-13	-2	3	5.09	2.78
13	2	-3	8.60	3.01
-12	-2	3	57.53	5.98
12	2	-3	50.51	5.68
-11	-2	3	15.91	3.38
-11	2	3	13.71	1.55
11	2	-3	11.97	3.12
-10	-2	3	23.99	3.73
10	2	-3	15.56	3.13
-10	2	3	21.72	1.52
-9	-2	3	10.45	2.49
9	2	-3	13.91	2.88
-9	2	3	12.60	0.98
-8	-2	3	1.85	1.44
8	2	-3	1.49	1.44
-7	-2	3	83.05	5.06
7	2	-3	89.30	5.20
-6	-2	3	61.42	4.22
6	2	-3	60.48	4.09
6	2	-3	65.69	4.29

-5	-2	3	150.95	6.28
5	2	-3	151.99	6.22
-4	-2	3	42.90	3.30
4	2	-3	40.82	3.18
3	-2	-3	0.17	0.31
-3	-2	3	-1.21	0.73
-3	2	3	0.31	0.41
3	2	-3	0.77	0.94
3	2	-3	-0.14	0.83
-2	-2	3	200.22	6.44
2	-2	-3	194.86	4.31
2	2	-3	199.65	6.10
-2	2	3	192.80	4.46
2	2	-3	196.29	6.29
-1	-2	3	79.75	3.92
1	-2	-3	76.89	3.01
1	-2	-3	83.07	3.02
1	2	-3	87.60	3.83
-1	2	3	80.47	2.91
-1	2	3	78.08	2.99
1	2	-3	76.94	3.75
0	-2	-3	605.74	11.38
0	-2	3	619.13	12.34
0	-2	-3	624.40	11.63
0	2	3	613.11	11.48
0	2	-3	614.34	11.76
0	2	3	611.52	11.15
0	2	-3	618.84	12.14
-1	-2	-3	80.00	3.67
1	-2	3	79.88	3.61
-1	-2	-3	80.43	3.65
1	2	3	83.27	3.66
-1	2	-3	83.39	3.66
1	2	3	79.52	3.49
-1	2	-3	78.99	3.37
-2	-2	-3	1225.03	21.12
-2	-2	-3	1210.18	21.09
2	-2	3	1252.86	20.21
-2	2	-3	1241.24	20.03
-2	2	-3	1213.80	19.37
2	2	3	1239.20	20.66
2	2	3	1255.83	21.04
3	-2	3	4.01	1.16
-3	-2	-3	5.64	1.70
-3	-2	-3	2.37	1.20
-3	2	-3	5.09	1.17
3	2	3	1.29	1.06
3	2	3	5.19	1.42
4	-2	3	112.67	4.10
-4	-2	-3	109.83	5.21

-4	-2	-3	108.58	5.13
4	2	3	112.38	4.99
4	2	3	107.99	5.07
-5	-2	-3	49.99	3.72
-5	-2	-3	54.19	3.99
5	-2	3	46.46	2.74
5	2	3	47.67	3.56
-5	2	-3	48.42	2.80
5	2	3	47.38	3.58
-6	-2	-3	202.93	7.79
-6	-2	-3	211.43	8.11
6	-2	3	198.97	5.79
-6	2	-3	200.70	5.90
6	2	3	201.93	7.53
-7	-2	-3	3.66	1.74
7	-2	3	1.40	0.87
-7	-2	-3	1.89	1.50
7	2	3	-1.14	0.96
-7	2	-3	0.55	0.92
-8	-2	-3	1.67	1.70
8	-2	3	3.57	1.25
-8	-2	-3	3.28	1.81
8	-2	3	2.63	1.13
-8	2	-3	4.95	1.44
8	2	3	3.47	1.77
9	-2	3	36.96	2.71
-9	-2	-3	37.80	4.75
9	2	3	35.78	4.39
-9	2	-3	34.92	2.88
10	-2	3	63.07	3.71
-10	-2	-3	67.92	5.66
-10	-2	-3	68.84	5.36
10	-2	3	50.51	3.26
10	2	3	64.03	6.16
-10	2	-3	63.34	3.94
11	-2	3	255.90	7.83
-11	-2	-3	264.41	11.21
11	-2	3	263.78	7.52
11	2	3	250.00	10.48
-11	2	-3	254.60	8.24
12	-2	3	31.49	3.22
-12	-2	-3	33.51	5.07
12	2	3	24.91	4.40
-12	2	-3	33.86	3.71
-13	-2	-3	-0.69	1.77
-13	2	-3	-0.64	1.31
13	2	3	2.90	2.29
-14	-2	-3	33.59	5.56
14	2	3	30.80	5.26
-14	2	-3	33.73	4.03

-15	-2	-3	-1.94	1.63
15	2	3	0.31	2.39
-16	-2	-3	-1.22	2.44
16	2	3	6.39	3.30
-15	-3	3	81.43	9.15
-15	-3	3	89.41	7.82
15	3	-3	83.76	8.85
-14	-3	3	12.14	3.95
-14	-3	3	17.23	4.27
14	3	-3	16.12	4.19
-13	-3	3	1.16	2.39
13	3	-3	-1.80	2.32
-12	-3	3	130.74	8.39
12	3	-3	136.69	8.49
-11	-3	3	31.21	4.71
11	3	-3	31.72	5.04
-10	-3	3	84.29	6.36
10	3	-3	70.12	5.94
-9	-3	3	64.56	5.44
9	3	-3	60.57	6.09
-8	-3	3	34.42	4.54
8	3	-3	25.33	3.93
-7	-3	3	179.54	8.33
-6	-3	3	160.31	7.60
6	3	-3	175.08	7.56
-5	-3	3	1.40	1.42
5	3	-3	0.85	1.50
-4	-3	3	387.68	11.44
4	3	-3	373.06	10.81
-4	3	3	391.92	6.86
3	-3	-3	461.84	8.41
-3	-3	3	463.25	12.44
3	3	-3	488.27	12.03
-3	3	3	463.65	8.78
2	-3	-3	60.98	2.86
-2	-3	3	64.43	4.19
2	-3	-3	57.50	2.96
-2	3	3	60.03	2.91
2	3	-3	62.50	4.09
-2	3	3	61.22	2.78
2	3	-3	61.33	4.04
-1	-3	3	628.06	14.11
1	-3	-3	605.84	12.01
1	-3	-3	619.98	12.40
-1	3	3	613.67	12.09
1	3	-3	613.77	13.33
-1	3	3	600.60	11.70
1	3	-3	623.21	13.84
0	-3	-3	232.96	7.09
0	-3	3	238.29	7.61

0	-3	-3	245.48	7.09
0	3	3	225.32	6.78
0	3	-3	233.58	7.42
0	3	3	237.97	6.77
0	3	-3	225.84	7.00
1	-3	3	49.79	3.41
-1	-3	-3	51.41	3.52
-1	-3	-3	50.39	3.37
1	3	3	51.96	3.34
-1	3	-3	51.30	3.23
1	3	3	53.28	3.43
-1	3	-3	44.01	3.26
-2	-3	-3	1358.20	24.49
-2	-3	-3	1386.94	24.50
2	-3	3	1351.43	23.21
-2	3	-3	1359.89	23.05
-2	3	-3	1374.85	22.44
2	3	3	1346.49	23.71
2	3	3	1349.43	24.05
-3	-3	-3	40.61	4.21
3	-3	3	33.65	2.71
-3	-3	-3	31.36	3.45
3	3	3	36.70	3.71
3	3	3	33.65	3.50
-3	3	-3	35.79	2.82
-3	3	-3	33.43	2.51
-4	-3	-3	158.85	7.01
4	-3	3	147.79	5.37
-4	-3	-3	149.39	6.81
-4	3	-3	151.37	5.00
4	3	3	151.88	6.54
-5	-3	-3	672.69	16.63
-5	-3	-3	688.77	16.88
5	-3	3	683.77	13.42
-5	3	-3	672.81	12.52
5	3	3	680.33	16.10
-6	-3	-3	5.85	2.27
6	-3	3	6.63	1.51
-6	-3	-3	4.07	1.98
-6	3	-3	5.89	1.32
6	3	3	4.69	2.06
-7	-3	-3	356.49	12.35
-7	-3	-3	352.86	12.03
7	-3	3	348.62	8.46
7	3	3	326.32	11.29
-7	3	-3	342.64	7.65
8	-3	3	30.69	2.78
-8	-3	-3	29.37	4.21
-8	-3	-3	27.42	4.35
8	3	3	23.28	3.90

-9	-3	-3	67.43	5.83
-9	-3	-3	85.99	6.69
9	-3	3	75.06	3.77
9	3	3	81.84	6.17
-10	-3	-3	169.97	9.38
10	-3	3	158.92	5.46
-10	-3	-3	154.46	8.71
10	3	3	175.60	9.00
11	-3	3	96.84	4.35
-11	-3	-3	93.87	7.13
-11	-3	-3	105.33	7.81
11	3	3	108.08	7.52
-12	-3	-3	20.21	4.59
12	3	3	17.26	4.07
-13	-3	-3	41.90	6.33
13	3	3	37.07	6.04
-14	-3	-3	-2.21	2.07
14	3	3	-2.88	2.01
-15	-3	-3	5.47	3.49
15	3	3	13.67	4.28
-14	-4	3	22.37	5.41
-14	-4	3	21.92	5.41
14	4	-3	38.07	6.42
13	4	-3	41.61	6.49
-12	-4	3	2.20	2.51
12	4	-3	2.41	2.98
-11	-4	3	65.19	7.30
11	4	-3	69.92	7.57
-10	-4	3	113.16	8.12
10	4	-3	106.12	7.68
-9	-4	3	111.49	7.81
9	4	-3	114.08	7.73
-8	-4	3	110.43	7.47
8	4	-3	120.90	7.69
-7	-4	3	26.55	4.26
7	4	-3	22.23	4.09
-6	-4	3	188.94	9.22
6	4	-3	194.05	9.07
-5	-4	3	217.08	9.49
5	4	-3	211.80	9.08
-5	4	3	207.99	4.60
-4	-4	3	11.24	3.05
4	-4	-3	25.60	1.79
-4	4	3	24.89	2.10
4	4	-3	30.89	4.36
3	-4	-3	179.06	5.38
3	-4	-3	183.18	5.80
-3	-4	3	185.60	8.28
-3	4	3	183.52	5.31
-3	4	3	180.02	5.67

3	4	-3	189.08	8.06
2	-4	-3	35.20	3.26
2	-4	-3	33.79	2.83
-2	-4	3	35.47	4.23
2	4	-3	37.94	4.25
-2	4	3	35.33	2.96
-2	4	3	32.07	2.73
1	-4	-3	90.32	4.71
-1	-4	3	91.04	5.49
1	-4	-3	97.34	4.69
-1	4	3	99.56	4.54
-1	4	3	98.60	4.75
1	4	-3	98.52	5.38
0	-4	-3	60.12	4.11
0	-4	3	64.99	4.53
0	-4	-3	61.39	4.05
0	4	-3	67.43	4.31
0	4	3	62.02	3.90
0	4	3	60.33	4.02
1	-4	3	42.87	3.70
-1	-4	-3	44.47	3.76
-1	-4	-3	38.19	4.06
1	4	3	43.17	3.59
-1	4	-3	48.14	3.58
-2	-4	-3	31.56	3.80
-2	-4	-3	31.38	3.95
2	-4	3	32.38	3.52
-2	4	-3	31.47	3.44
2	4	3	34.22	3.85
-2	4	-3	31.83	3.33
-3	-4	-3	210.28	8.67
3	-4	3	217.21	7.58
-3	-4	-3	216.58	8.77
-3	4	-3	221.22	6.99
3	4	3	217.72	8.43
-3	4	-3	220.69	7.40
-4	-4	-3	158.41	7.84
4	-4	3	153.36	6.15
-4	-4	-3	163.23	7.93
-4	4	-3	159.94	6.17
4	4	3	169.52	7.76
-4	4	-3	161.08	5.69
-5	-4	-3	43.97	5.03
5	-4	3	56.53	3.69
-5	-4	-3	62.97	6.38
5	4	3	53.32	5.41
-5	4	-3	58.99	3.39
-6	-4	-3	303.47	11.96
-6	-4	-3	325.85	12.15
6	-4	3	332.45	8.80

6	4	3	317.80	11.66
-7	-4	-3	0.63	1.88
-7	-4	-3	-0.60	1.76
7	-4	3	0.64	1.09
7	4	3	-0.99	1.92
-8	-4	-3	60.62	6.32
8	-4	3	54.74	3.33
-8	-4	-3	61.80	6.85
8	4	3	48.96	5.92
-9	-4	-3	53.62	6.64
-9	-4	-3	48.93	6.08
9	4	3	52.86	6.23
-10	-4	-3	30.50	5.41
-10	-4	-3	27.26	4.88
10	4	3	34.53	5.43
-11	-4	-3	95.55	7.91
-11	-4	-3	90.18	7.58
11	4	3	79.38	8.24
-12	-4	-3	39.58	6.19
-12	-4	-3	31.86	5.56
12	4	3	26.80	5.25
-13	-4	-3	0.01	2.52
13	4	3	-1.41	2.46
-11	-5	3	-0.96	2.27
11	5	-3	-1.74	2.35
-10	-5	3	-0.29	2.25
10	5	-3	-2.24	2.14
-9	-5	3	-1.70	1.86
9	5	-3	-0.80	2.44
-8	-5	3	51.48	6.60
8	5	-3	58.41	6.96
-7	-5	3	-2.75	1.67
7	5	-3	2.67	2.57
-6	-5	3	16.05	3.72
6	5	-3	12.53	3.49
-5	-5	3	-0.51	1.89
5	5	-3	2.60	2.31
-4	-5	3	15.29	3.53
4	5	-3	16.62	3.71
-3	-5	3	202.14	9.58
3	-5	-3	192.62	6.77
3	-5	-3	192.21	6.32
3	5	-3	188.52	8.79
2	-5	-3	0.57	1.26
-2	-5	3	0.80	1.68
2	-5	-3	-0.82	1.28
2	5	-3	-0.66	1.79
1	-5	-3	-1.04	1.34
-1	-5	3	-0.79	1.58
1	-5	-3	-2.67	1.04

1	5	-3	2.37	1.96
-1	5	3	-0.18	1.19
0	-5	-3	11.59	2.63
0	-5	-3	10.02	2.53
0	-5	3	7.32	2.44
0	5	3	9.32	2.29
0	5	-3	8.73	2.49
-1	-5	-3	0.93	1.71
1	-5	3	2.71	1.89
-1	-5	-3	2.98	1.96
1	5	3	2.89	1.84
-1	5	-3	-1.85	1.37
2	-5	3	0.69	1.67
-2	-5	-3	1.45	1.70
-2	-5	-3	3.05	1.97
-2	5	-3	1.31	1.67
2	5	3	1.55	1.94
-3	-5	-3	7.77	2.81
3	-5	3	17.47	2.90
-3	-5	-3	9.69	2.78
3	5	3	10.12	2.92
-3	5	-3	14.50	2.65
-4	-5	-3	17.49	3.80
4	-5	3	15.72	2.76
-4	-5	-3	12.28	3.13
4	5	3	13.70	3.29
-5	-5	-3	9.59	2.96
5	-5	3	7.49	2.07
-5	-5	-3	5.06	2.77
5	5	3	0.66	2.29
6	-5	3	3.21	1.64
-6	-5	-3	2.20	2.21
-6	-5	-3	3.43	2.54
6	5	3	5.18	2.90
-7	-5	-3	6.25	2.91
-7	-5	-3	0.22	2.06
7	5	3	4.43	2.65
-8	-5	-3	3.91	3.05
-8	-5	-3	1.11	2.27
8	5	3	0.82	2.37
-9	-5	-3	23.10	5.18
-9	-5	-3	23.02	4.77
9	5	3	22.87	4.93
-10	-5	-3	-3.21	1.99
-10	-5	-3	4.41	2.87
10	5	3	1.80	2.93
-11	-5	-3	3.88	2.96
-11	-5	-3	7.16	3.41
11	5	3	3.06	3.32
-7	-6	3	11.82	4.04

7	6	-3	14.35	4.28
-6	-6	3	1.11	2.68
6	6	-3	-1.20	2.23
-5	-6	3	75.49	8.10
5	6	-3	77.40	7.97
-4	-6	3	3.14	2.60
4	6	-3	2.27	2.59
-3	-6	3	39.82	5.48
3	6	-3	48.53	5.91
-2	-6	3	52.52	6.11
2	6	-3	34.83	4.99
-1	-6	3	3.41	2.26
1	6	-3	0.90	2.06
0	-6	-3	23.40	3.91
0	-6	3	20.03	3.88
0	-6	-3	23.12	3.81
0	6	-3	23.14	3.98
-1	-6	-3	16.78	3.55
-1	-6	-3	23.12	4.11
1	-6	3	22.59	4.00
1	6	3	16.81	3.37
-1	6	-3	18.51	3.53
-2	-6	-3	-1.73	1.86
-2	-6	-3	0.59	2.20
2	-6	3	0.05	1.86
2	6	3	4.40	2.48
-3	-6	-3	-0.12	2.09
-3	-6	-3	1.02	2.39
3	6	3	0.26	2.09
-4	-6	-3	7.45	3.08
-4	-6	-3	10.73	3.52
4	6	3	4.42	2.85
-5	-6	-3	0.42	2.40
-5	-6	-3	1.80	2.54
5	6	3	9.03	3.35
-6	-6	-3	8.95	3.48
-6	-6	-3	12.63	3.83
6	6	3	3.36	3.03
-17	0	4	127.91	7.94
17	0	-4	132.29	7.41
-16	0	4	2.47	2.04
16	0	-4	4.59	2.38
-15	0	4	6.66	2.55
15	0	-4	5.33	2.45
-14	0	4	38.62	4.65
-14	0	4	50.49	4.32
14	0	-4	44.76	4.69
-13	0	4	585.43	15.31
-13	0	4	582.67	14.50
13	0	-4	559.62	14.32

-12	0	4	544.71	13.67
-12	0	4	581.74	14.66
12	0	-4	551.23	13.63
-11	0	4	293.07	9.06
11	0	-4	290.41	8.90
11	0	-4	280.76	8.90
10	0	-4	1718.46	28.60
-10	0	4	1724.27	28.73
10	0	-4	1695.42	28.40
9	0	-4	301.07	8.26
-9	0	4	299.61	8.37
9	0	-4	285.33	8.16
8	0	-4	110.01	4.55
-8	0	4	105.55	4.52
8	0	-4	101.42	4.41
7	0	-4	384.82	8.78
-7	0	4	358.23	8.61
7	0	-4	356.03	8.49
-6	0	4	167.39	5.21
6	0	-4	167.16	5.14
6	0	-4	175.22	5.23
5	0	-4	764.85	13.35
-5	0	4	765.90	13.43
5	0	-4	765.08	13.30
-4	0	4	1056.15	16.82
4	0	-4	1045.36	16.68
4	0	-4	1039.07	16.69
3	0	-4	121.10	3.70
-3	0	4	125.67	3.78
3	0	-4	118.48	3.62
-2	0	4	1007.42	15.95
-2	0	4	1013.07	15.82
2	0	-4	1019.36	15.70
2	0	-4	1005.42	15.73
-1	0	4	9999.00	136.42
1	0	-4	9702.70	138.16
-1	0	4	9671.91	132.42
0	0	4	4385.20	58.69
0	0	-4	4360.65	59.11
0	0	4	4341.51	58.97
1	0	4	1561.77	22.66
-1	0	-4	1487.36	22.95
2	0	4	4653.08	63.63
2	0	4	4733.15	63.28
-2	0	-4	4625.05	63.77
3	0	4	55.95	2.81
-3	0	-4	49.57	2.83
4	0	4	204.23	5.82
-4	0	-4	198.00	6.10
5	0	4	17.34	2.13

-5	0	-4	15.06	2.16
6	0	4	703.36	14.02
6	0	4	710.50	14.36
-6	0	-4	711.94	14.80
-7	0	-4	0.97	1.06
7	0	4	1.49	1.10
7	0	4	1.74	1.19
8	0	4	58.25	3.99
8	0	4	54.29	3.74
-8	0	-4	57.27	4.13
9	0	4	269.68	8.62
9	0	4	269.40	8.84
-9	0	-4	255.14	9.00
10	0	4	526.63	13.34
-10	0	-4	546.68	14.42
11	0	4	43.69	4.50
-11	0	-4	48.18	5.17
-12	0	-4	166.77	8.16
12	0	4	170.35	7.68
-13	0	-4	106.83	6.79
13	0	4	104.45	6.38
14	0	4	42.22	5.07
-14	0	-4	32.19	4.71
15	0	4	37.71	4.93
-15	0	-4	37.53	5.48
-16	0	-4	0.31	2.19
16	0	4	2.70	2.19
17	0	4	50.63	6.15
-17	0	-4	55.57	6.86
-17	-1	4	2.66	2.46
-17	-1	4	-1.09	2.36
17	1	-4	-0.42	2.17
-16	-1	4	16.64	3.71
-16	-1	4	20.54	4.38
16	1	-4	10.54	3.29
-15	-1	4	2.08	2.12
-15	-1	4	1.43	2.25
15	1	-4	-0.01	1.91
-14	-1	4	7.48	2.80
14	-1	-4	9.48	2.28
-14	-1	4	9.12	2.80
-14	1	4	8.73	2.27
14	1	-4	9.24	2.75
-13	-1	4	0.72	1.89
13	-1	-4	1.88	1.51
-13	1	4	0.91	1.34
13	1	-4	4.84	2.34
-12	-1	4	86.92	5.73
12	-1	-4	84.67	4.20
12	-1	-4	98.26	4.54

12	1	-4	77.61	5.66
-12	1	4	96.64	4.82
11	-1	-4	140.86	5.29
-11	-1	4	151.16	7.27
11	-1	-4	142.42	5.09
11	1	-4	135.06	6.94
-11	1	4	145.72	5.70
-10	-1	4	157.08	7.00
10	-1	-4	147.58	5.07
10	-1	-4	149.04	4.86
10	1	-4	160.09	6.92
-10	1	4	157.26	5.51
-10	1	4	148.43	4.87
9	-1	-4	159.52	4.71
-9	-1	4	159.90	6.78
9	-1	-4	160.52	4.94
-9	1	4	160.77	5.21
-9	1	4	154.57	4.73
9	1	-4	156.33	6.55
8	-1	-4	162.64	4.41
8	-1	-4	156.72	4.61
-8	-1	4	156.61	6.46
-8	1	4	161.29	4.48
8	1	-4	161.52	6.47
8	1	-4	163.06	6.50
-7	-1	4	6.45	1.74
7	-1	-4	6.07	1.03
7	-1	-4	7.20	1.21
7	1	-4	3.60	1.43
7	1	-4	6.75	1.74
-7	1	4	5.41	1.03
6	-1	-4	43.69	1.91
-6	-1	4	42.99	3.15
6	1	-4	43.36	3.16
6	1	-4	43.64	3.12
-6	1	4	46.28	1.94
-5	-1	4	-0.59	0.83
5	-1	-4	-0.03	0.46
-5	1	4	-0.03	0.46
5	1	-4	0.34	0.91
5	1	-4	-0.32	0.78
-4	-1	4	244.42	6.95
4	1	-4	235.66	6.74
-4	1	4	240.61	4.86
-4	1	4	238.95	4.60
4	1	-4	247.14	6.72
-3	-1	4	858.64	15.58
3	1	-4	863.06	15.46
-3	1	4	859.25	12.89
3	1	-4	881.92	15.33

-3	1	4	867.22	13.05
-2	-1	4	23.44	2.05
2	-1	-4	20.40	1.35
2	1	-4	24.05	2.19
2	1	-4	20.29	2.07
-2	1	4	22.12	1.37
1	-1	-4	15.67	1.35
-1	-1	4	11.48	1.63
-1	1	4	16.46	1.40
1	1	-4	14.67	1.68
1	1	-4	14.67	1.78
0	-1	4	8764.93	110.70
0	-1	-4	8119.38	121.80
0	1	-4	8533.40	110.47
0	1	4	8084.64	116.68
0	1	4	8128.69	118.41
0	1	-4	8267.81	109.96
1	-1	4	79.31	3.30
-1	-1	-4	72.43	3.14
-1	1	-4	70.20	3.16
1	1	4	74.82	3.16
-1	1	-4	70.58	3.22
1	1	4	77.44	3.07
2	-1	4	-1.09	0.57
-2	-1	-4	-0.44	0.65
-2	1	-4	-0.24	0.56
2	1	4	-0.91	0.50
2	1	4	-0.30	0.61
3	-1	4	1137.31	18.23
-3	-1	-4	1157.17	19.86
-3	1	-4	1152.85	18.98
3	1	4	1112.75	19.08
3	1	4	1149.07	19.56
-4	-1	-4	1.27	1.13
4	-1	4	0.91	0.85
4	1	4	0.73	1.00
4	1	4	2.37	1.07
-4	1	-4	0.31	0.76
-5	-1	-4	218.57	7.47
5	-1	4	224.88	6.13
5	1	4	236.50	7.21
-5	1	-4	226.48	6.62
5	1	4	229.72	7.34
6	-1	4	25.63	2.48
-6	-1	-4	27.15	3.35
6	1	4	28.36	3.11
-6	1	-4	23.23	2.60
6	1	4	26.46	2.96
7	-1	4	47.37	3.08
-7	-1	-4	48.95	3.93

7	1	4	51.30	3.89
-7	1	-4	54.78	3.55
7	1	4	54.47	3.84
-8	-1	-4	34.32	4.15
8	-1	4	32.63	3.13
8	1	4	32.34	3.67
-8	1	-4	33.46	3.44
9	-1	4	96.43	4.83
9	-1	4	95.92	4.65
-9	-1	-4	91.11	5.83
-9	1	-4	86.73	4.91
9	1	4	90.23	5.37
-10	-1	-4	26.54	4.06
10	-1	4	22.26	2.99
10	-1	4	26.12	3.17
10	1	4	16.41	3.08
-10	1	-4	26.96	3.48
-11	-1	-4	0.76	1.76
11	-1	4	2.38	1.58
11	1	4	1.95	1.84
-11	1	-4	1.48	1.46
-12	-1	-4	2.42	2.10
12	-1	4	1.02	1.60
-12	1	-4	0.34	1.50
12	1	4	1.38	2.03
-13	-1	-4	9.83	3.08
13	-1	4	8.98	2.45
13	1	4	3.70	2.29
-13	1	-4	10.55	2.76
14	-1	4	1.98	1.81
-14	-1	-4	0.95	2.19
-14	1	-4	2.06	1.97
14	1	4	3.71	2.54
-15	-1	-4	0.46	2.13
15	-1	4	0.35	1.72
-15	1	-4	-2.12	1.58
15	1	4	-3.95	1.57
16	-1	4	9.69	2.75
-16	-1	-4	12.98	3.84
16	1	4	3.04	2.80
-16	1	-4	10.89	3.25
-17	-1	-4	4.66	2.93
17	-1	4	2.34	2.44
-17	1	-4	9.37	3.09
17	1	4	2.08	2.86
-16	-2	4	19.11	4.54
-16	-2	4	5.56	3.07
16	2	-4	11.19	3.85
-15	-2	4	19.87	4.33
-15	-2	4	19.64	4.25

15	2	-4	26.67	4.87
-14	-2	4	11.53	3.36
14	2	-4	7.82	3.11
-13	-2	4	96.51	6.99
13	2	-4	104.20	7.15
-12	-2	4	119.22	7.36
12	2	-4	125.73	7.48
-11	-2	4	32.08	4.38
11	2	-4	34.36	4.69
-10	-2	4	198.90	8.77
10	2	-4	214.04	9.00
-10	2	4	203.49	4.20
-9	-2	4	156.75	7.53
9	2	-4	152.53	7.24
-8	-2	4	34.52	4.06
8	2	-4	33.15	3.98
-7	-2	4	265.71	9.27
7	2	-4	268.72	9.06
-6	-2	4	194.44	7.54
6	2	-4	170.90	6.84
-5	-2	4	836.94	17.60
5	2	-4	854.07	17.54
-4	-2	4	92.28	4.78
4	2	-4	103.03	4.81
4	2	-4	100.25	4.87
-3	-2	4	27.30	2.97
3	-2	-4	30.79	1.37
3	2	-4	31.36	3.11
-3	2	4	28.85	1.44
3	2	-4	24.87	2.69
-2	-2	4	69.42	3.88
2	-2	-4	71.65	2.56
-2	2	4	75.73	2.74
2	2	-4	78.94	3.89
2	2	-4	75.59	3.96
1	-2	-4	7.02	1.19
-1	-2	4	5.27	1.50
1	2	-4	6.29	1.49
-1	2	4	11.87	1.45
1	2	-4	7.53	1.62
-1	2	4	6.96	1.19
0	-2	-4	153.49	4.88
0	-2	4	154.84	5.46
0	2	-4	161.17	5.28
0	2	4	144.51	4.73
0	2	4	150.21	4.65
0	2	-4	152.27	5.37
-1	-2	-4	3.82	1.22
1	-2	4	5.46	1.46
1	2	4	11.90	1.74

-1	2	-4	7.44	1.48
-1	2	-4	4.15	1.22
1	2	4	3.13	1.12
2	-2	4	89.97	4.01
-2	-2	-4	83.75	4.24
-2	2	-4	82.57	3.76
2	2	4	81.79	3.87
-2	2	-4	87.23	3.67
2	2	4	85.06	4.17
-3	-2	-4	7.66	1.74
3	-2	4	4.45	1.25
3	2	4	6.55	1.57
3	2	4	6.24	1.59
4	-2	4	186.10	5.81
-4	-2	-4	193.79	7.09
-4	-2	-4	195.05	7.03
4	2	4	196.04	6.77
-4	2	-4	188.54	5.94
4	2	4	189.04	6.88
-5	-2	-4	13.69	2.42
-5	-2	-4	16.39	2.62
5	-2	4	14.61	1.98
5	2	4	11.77	2.25
-5	2	-4	18.71	2.27
5	2	4	21.16	2.89
-6	-2	-4	-0.73	1.08
6	-2	4	-0.47	0.73
-6	2	-4	-1.55	0.67
6	2	4	0.20	1.20
7	-2	4	58.21	3.36
-7	-2	-4	65.78	4.83
-7	2	-4	55.08	3.47
7	2	4	65.27	4.54
8	-2	4	18.45	2.29
8	-2	4	17.35	2.34
-8	-2	-4	24.25	3.80
-8	2	-4	20.76	2.67
8	2	4	17.76	3.09
9	-2	4	53.28	3.38
-9	-2	-4	48.33	5.40
9	2	4	60.22	4.94
-9	2	-4	54.80	3.79
10	-2	4	30.48	3.20
10	-2	4	27.61	3.06
-10	-2	-4	24.32	4.25
-10	2	-4	34.05	3.67
10	2	4	35.70	4.75
-11	-2	-4	13.05	3.63
11	-2	4	14.43	2.47
11	-2	4	11.26	2.21

11	2	4	13.18	3.48
-11	2	-4	12.40	2.55
12	-2	4	72.46	4.33
-12	-2	-4	71.89	7.56
-12	2	-4	66.62	4.63
12	2	4	71.96	6.07
13	-2	4	9.71	2.29
-13	-2	-4	5.56	2.91
-13	2	-4	6.92	2.22
13	2	4	4.95	2.67
-14	-2	-4	13.95	4.11
14	2	4	8.64	3.43
-14	2	-4	7.06	2.29
-15	-2	-4	69.97	7.95
15	2	4	71.18	7.72
-16	-2	-4	12.65	4.30
16	2	4	13.69	4.06
-15	-3	4	4.86	3.16
-15	-3	4	1.22	2.76
15	3	-4	5.45	3.16
-14	-3	4	36.27	5.76
14	3	-4	33.14	5.55
-13	-3	4	108.44	7.98
13	3	-4	115.49	8.15
-12	-3	4	1.65	2.22
12	3	-4	-1.25	2.23
-11	-3	4	12.29	3.29
11	3	-4	11.89	3.62
-10	-3	4	1.49	1.96
10	3	-4	0.81	2.18
-9	-3	4	41.07	5.08
9	3	-4	46.60	5.35
-8	-3	4	32.93	4.39
8	3	-4	21.17	3.63
-7	-3	4	24.94	3.76
7	3	-4	20.55	3.52
-6	-3	4	72.65	5.24
6	3	-4	72.76	5.25
-5	-3	4	161.71	7.40
5	3	-4	134.45	6.56
-4	-3	4	2.26	1.57
-4	3	4	0.05	0.57
4	3	-4	0.18	1.33
-3	-3	4	-0.36	1.18
3	-3	-4	-0.26	0.62
-3	3	4	-0.57	0.66
3	3	-4	-1.47	1.06
-2	-3	4	237.27	8.27
2	-3	-4	219.72	5.76
-2	3	4	222.01	5.66

-2	3	4	227.32	5.96
2	3	-4	216.24	7.79
2	3	-4	216.05	7.58
1	-3	-4	83.48	3.79
-1	-3	4	88.79	4.86
1	3	-4	90.57	4.80
-1	3	4	81.21	3.76
-1	3	4	84.54	3.69
1	3	-4	82.84	4.45
0	-3	-4	54.28	3.39
0	-3	-4	53.98	3.28
0	-3	4	51.59	3.67
0	3	4	55.31	3.28
0	3	-4	52.53	3.54
0	3	-4	59.70	3.86
0	3	4	51.67	3.26
-1	-3	-4	12.94	2.23
1	-3	4	10.11	2.02
-1	-3	-4	11.52	2.04
-1	3	-4	13.07	2.07
1	3	4	14.99	2.21
1	3	4	13.02	2.11
-1	3	-4	9.76	1.91
2	-3	4	183.08	6.55
-2	-3	-4	190.77	7.09
-2	-3	-4	187.71	7.09
2	3	4	182.63	6.90
2	3	4	184.71	6.75
-2	3	-4	196.35	6.30
-2	3	-4	195.58	6.65
3	-3	4	103.84	4.78
-3	-3	-4	102.73	5.43
-3	-3	-4	101.91	5.38
-3	3	-4	98.56	4.34
3	3	4	102.60	5.26
-3	3	-4	101.26	4.68
3	3	4	95.13	5.13
4	-3	4	2.37	1.28
-4	-3	-4	-1.02	1.19
-4	-3	-4	2.00	1.64
4	3	4	0.34	1.27
-4	3	-4	0.74	0.93
5	-3	4	694.38	14.02
-5	-3	-4	647.99	16.55
-5	-3	-4	654.21	16.45
5	3	4	676.79	16.12
-5	3	-4	659.33	12.92
-6	-3	-4	405.46	12.82
6	-3	4	397.44	9.78
-6	3	-4	388.15	8.96

6	3	4	385.79	12.00
-7	-3	-4	147.72	7.75
7	-3	4	160.45	5.83
-7	-3	-4	145.37	7.83
-7	3	-4	146.10	5.08
7	3	4	153.56	7.80
8	-3	4	0.30	1.16
-8	-3	-4	0.76	1.83
-8	-3	-4	-2.33	1.62
-8	3	-4	0.18	1.20
8	3	4	3.33	2.30
-9	-3	-4	144.15	8.49
9	-3	4	148.02	5.67
9	3	4	160.46	8.50
10	-3	4	4.06	1.64
-10	-3	-4	-0.83	1.91
10	3	4	4.32	2.66
11	-3	4	72.51	4.20
-11	-3	-4	77.72	6.84
-11	3	-4	80.73	4.52
11	3	4	69.99	7.20
-12	-3	-4	42.95	6.38
12	3	4	59.28	7.03
-13	-3	-4	52.98	7.24
13	3	4	42.02	6.15
-14	-3	-4	18.56	4.96
14	3	4	19.90	4.75
-15	-3	-4	25.74	5.55
15	3	4	13.22	4.48
-13	-4	4	-0.94	2.30
13	4	-4	1.05	2.75
-12	-4	4	8.56	3.38
12	4	-4	9.83	3.71
-11	-4	4	0.14	2.10
11	4	-4	1.76	2.62
-10	-4	4	16.40	3.89
10	4	-4	18.76	4.16
-9	-4	4	164.78	9.39
9	4	-4	168.21	9.26
-8	-4	4	10.46	3.26
8	4	-4	14.77	3.58
-7	-4	4	79.63	6.41
7	4	-4	83.00	6.42
-6	-4	4	1.88	2.09
6	4	-4	6.77	2.66
-5	-4	4	0.47	1.82
5	4	-4	1.30	1.97
4	-4	-4	5.95	0.99
-4	-4	4	4.10	2.17
4	4	-4	5.13	2.53

-4	4	4	5.63	1.28
3	-4	-4	241.92	6.22
-3	-4	4	233.34	9.57
3	4	-4	228.74	9.11
-3	4	4	234.84	6.58
-2	-4	4	13.13	2.91
2	-4	-4	10.14	1.94
-2	4	4	15.09	2.15
2	4	-4	9.54	2.38
-2	4	4	12.44	2.06
1	-4	-4	-1.28	1.03
1	-4	-4	-0.41	0.97
-1	-4	4	-0.49	1.29
-1	4	4	-0.14	1.05
-1	4	4	-1.03	0.97
1	4	-4	1.79	1.59
0	-4	-4	13.58	2.39
0	-4	-4	16.01	2.56
0	-4	4	13.33	2.60
0	4	4	17.30	2.68
0	4	4	17.69	2.60
0	4	-4	15.23	2.73
-1	-4	-4	13.51	2.60
-1	-4	-4	12.86	2.54
1	-4	4	12.68	2.54
-1	4	-4	10.71	2.30
1	4	4	11.43	2.34
-2	-4	-4	7.13	2.25
-2	-4	-4	5.08	1.90
2	-4	4	4.10	1.82
2	4	4	5.25	2.00
-2	4	-4	6.68	1.92
-3	-4	-4	1.91	1.64
3	-4	4	4.31	1.73
-3	-4	-4	4.62	2.10
-3	4	-4	-0.45	1.27
3	4	4	-2.37	1.16
-3	4	-4	1.81	1.38
-4	-4	-4	62.68	5.13
-4	-4	-4	57.08	4.88
4	-4	4	67.62	4.31
4	4	4	60.91	4.97
-4	4	-4	65.98	4.28
-4	4	-4	72.13	4.06
-5	-4	-4	6.11	2.52
-5	-4	-4	3.36	2.10
5	-4	4	1.55	1.38
-5	4	-4	1.90	1.33
5	4	4	2.52	2.07
-6	-4	-4	46.83	5.35

6	-4	4	51.67	3.74
-6	-4	-4	40.33	5.44
6	4	4	53.50	5.69
-7	-4	-4	76.32	6.25
7	-4	4	66.30	4.10
-7	-4	-4	69.45	5.87
7	4	4	63.42	6.49
8	-4	4	1.05	1.23
-8	-4	-4	-1.07	1.89
-8	-4	-4	-0.52	1.93
8	4	4	-2.22	1.88
-9	-4	-4	11.23	3.26
-9	-4	-4	5.49	2.98
9	-4	4	6.50	1.73
9	4	4	3.34	2.68
-10	-4	-4	5.39	2.78
-10	-4	-4	1.38	2.17
10	4	4	6.19	3.02
-11	-4	-4	7.87	3.45
11	4	4	10.38	3.73
-12	-4	-4	10.75	3.87
12	4	4	13.23	4.20
-13	-4	-4	4.13	3.19
13	4	4	2.20	3.07
-11	-5	4	37.63	6.30
11	5	-4	35.67	6.29
-10	-5	4	56.85	7.23
10	5	-4	51.63	7.02
-9	-5	4	-1.53	2.11
9	5	-4	-0.04	2.61
-8	-5	4	85.46	7.41
8	5	-4	91.71	7.46
-7	-5	4	42.39	5.82
7	5	-4	55.48	6.60
-6	-5	4	10.12	3.30
6	5	-4	16.82	3.92
-5	-5	4	18.19	3.94
5	5	-4	20.43	4.03
-4	-5	4	39.54	5.28
4	5	-4	19.82	3.86
-3	-5	4	1.03	2.06
3	5	-4	2.03	2.27
-2	-5	4	0.97	1.79
2	-5	-4	-0.83	1.33
2	5	-4	-2.56	1.41
1	-5	-4	73.24	4.69
-1	-5	4	74.86	5.70
1	5	-4	71.43	5.34
-1	5	4	70.07	4.46
0	-5	4	33.85	4.36

0	-5	-4	39.09	4.29
0	-5	-4	32.90	3.98
0	5	4	39.04	4.13
0	5	-4	40.89	4.66
-1	-5	-4	0.76	1.62
1	-5	4	2.79	1.77
-1	-5	-4	-0.80	1.31
1	5	4	-0.86	1.38
-1	5	-4	-0.74	1.70
-2	-5	-4	17.46	3.37
-2	-5	-4	17.28	3.37
2	-5	4	20.59	3.44
2	5	4	18.58	3.42
-2	5	-4	16.99	3.05
-3	-5	-4	190.20	8.96
3	-5	4	198.15	8.18
-3	-5	-4	180.91	8.89
-3	5	-4	177.01	7.17
3	5	4	176.47	8.42
4	-5	4	-0.44	1.34
-4	-5	-4	1.30	1.87
-4	-5	-4	4.94	2.40
4	5	4	2.64	2.33
5	-5	4	89.43	5.29
-5	-5	-4	94.66	6.95
-5	-5	-4	94.18	6.99
5	5	4	83.17	6.46
-6	-5	-4	34.54	5.21
-6	-5	-4	33.64	5.01
6	-5	4	39.57	3.96
6	5	4	42.35	5.67
-7	-5	-4	-0.21	1.94
-7	-5	-4	0.93	1.98
7	5	4	-0.04	2.12
-8	-5	-4	73.07	7.79
-8	-5	-4	65.31	7.40
8	5	4	58.88	6.93
-9	-5	-4	0.09	2.46
-9	-5	-4	0.16	1.97
9	5	4	1.50	2.71
-10	-5	-4	1.76	2.60
10	5	4	-3.25	2.13
-11	-5	-4	28.05	5.67
11	5	4	23.32	5.20
-6	-6	4	-2.52	2.05
6	6	-4	-0.51	2.50
-5	-6	4	1.38	2.59
5	6	-4	4.37	3.01
-4	-6	4	-0.03	2.36
4	6	-4	4.99	2.89

-3	-6	4	2.53	2.47
3	6	-4	-1.77	1.98
-2	-6	4	7.82	3.23
2	6	-4	10.85	3.35
-1	-6	4	4.52	2.67
1	6	-4	3.51	2.40
0	-6	4	0.47	2.08
0	-6	-4	3.46	2.16
0	6	-4	0.95	1.98
-1	-6	-4	7.18	2.62
1	-6	4	9.43	3.07
-1	6	-4	8.52	2.86
1	6	4	8.22	2.77
2	-6	4	-0.29	1.90
-2	-6	-4	-0.33	2.12
-2	-6	-4	1.65	2.31
2	6	4	0.73	2.27
-3	-6	-4	39.17	5.50
-3	-6	-4	40.49	5.49
3	6	4	36.35	5.05
-4	-6	-4	1.68	2.45
-4	-6	-4	5.03	2.75
4	6	4	7.68	3.26
-5	-6	-4	1.08	2.59
-5	-6	-4	-2.05	2.18
5	6	4	1.27	2.45
-6	-6	-4	0.18	2.18
-6	-6	-4	0.96	2.57
6	6	4	-2.56	1.99
17	0	-5	0.82	2.03
-17	0	5	1.48	2.21
-16	0	5	-1.62	1.78
16	0	-5	3.99	2.51
-15	0	5	-1.45	1.58
-15	0	5	-0.98	1.50
15	0	-5	-1.16	1.72
14	0	-5	1.76	1.92
-14	0	5	-0.55	1.49
-14	0	5	0.11	1.61
-13	0	5	-4.40	1.45
13	0	-5	-3.45	1.73
-13	0	5	0.44	1.87
12	0	-5	-3.68	1.64
-12	0	5	-2.85	1.41
11	0	-5	0.25	1.63
11	0	-5	-1.75	1.25
-11	0	5	-1.67	1.31
-10	0	5	-0.18	1.47
10	0	-5	1.56	1.57
10	0	-5	-1.66	1.53

9	0	-5	0.41	1.24
9	0	-5	-0.91	0.97
-9	0	5	0.53	1.06
8	0	-5	-1.24	0.88
8	0	-5	0.16	0.88
-8	0	5	-0.97	0.80
7	0	-5	-0.63	0.78
-7	0	5	-2.70	0.64
7	0	-5	0.95	1.05
6	0	-5	-0.68	0.83
-6	0	5	-1.12	0.69
6	0	-5	-0.67	0.74
-5	0	5	0.19	0.76
5	0	-5	0.99	0.85
5	0	-5	0.10	0.78
-4	0	5	-0.39	0.66
4	0	-5	-1.07	0.58
4	0	-5	0.43	0.72
3	0	-5	0.01	0.67
3	0	-5	0.15	0.69
-3	0	5	-0.02	0.74
-2	0	5	-0.03	0.86
-2	0	5	-1.33	0.73
2	0	-5	-2.33	0.68
-1	0	5	-0.13	0.67
1	0	-5	-0.44	0.59
-1	0	5	-1.05	0.53
0	0	5	-0.11	0.65
0	0	-5	-0.45	0.58
0	0	5	-0.30	0.63
1	0	5	-0.44	0.70
-1	0	-5	-0.88	0.66
-2	0	-5	0.70	0.72
2	0	5	0.08	0.63
-3	0	-5	-0.38	0.60
3	0	5	-0.48	0.68
-4	0	-5	-0.90	0.60
4	0	5	-0.63	0.70
5	0	5	-0.15	0.92
-5	0	-5	-1.25	0.82
-6	0	-5	-0.10	1.00
6	0	5	0.47	1.04
7	0	5	-0.88	0.92
-7	0	-5	-0.05	1.03
7	0	5	1.09	1.08
8	0	5	0.99	1.19
-8	0	-5	0.48	1.18
8	0	5	-1.51	0.95
9	0	5	-0.40	1.27
-9	0	-5	0.36	1.34

10	0	5	-0.44	1.36
-10	0	-5	1.51	1.64
11	0	5	0.20	1.62
-11	0	-5	-0.33	1.50
-12	0	-5	0.34	1.71
12	0	5	-0.94	1.59
-13	0	-5	3.11	2.30
13	0	5	1.37	1.86
-14	0	-5	-1.53	1.62
14	0	5	5.28	2.38
-15	0	-5	1.53	2.32
15	0	5	-2.38	1.54
-16	0	-5	0.82	2.19
16	0	5	-2.06	1.66
17	0	5	2.02	2.42
-17	0	-5	-1.32	1.90
-17	-1	5	-0.06	2.43
-17	-1	5	0.84	2.27
17	1	-5	4.36	2.89
-16	-1	5	39.60	5.65
-16	-1	5	35.40	4.99
16	1	-5	39.09	5.38
-15	-1	5	41.69	5.25
-15	-1	5	37.96	5.37
15	1	-5	40.53	5.19
-14	-1	5	6.02	2.53
-14	1	5	12.02	2.52
14	1	-5	5.17	2.64
-13	-1	5	74.92	5.60
13	-1	-5	81.44	4.41
-13	1	5	74.52	4.58
13	1	-5	78.28	5.67
-12	-1	5	75.66	5.51
12	-1	-5	66.78	3.96
12	-1	-5	70.23	3.85
-12	1	5	69.32	4.21
-12	1	5	67.84	3.80
12	1	-5	62.76	5.27
11	-1	-5	448.47	10.18
-11	-1	5	438.81	12.87
11	-1	-5	448.49	9.83
-11	1	5	453.86	9.99
-11	1	5	431.07	10.58
11	1	-5	461.69	13.01
10	-1	-5	266.74	7.12
-10	-1	5	244.79	9.01
10	-1	-5	264.19	6.78
-10	1	5	276.30	6.94
10	1	-5	264.12	9.16
-9	-1	5	334.76	10.29

9	-1	-5	334.73	7.54
9	-1	-5	340.98	7.91
9	1	-5	348.72	10.32
-9	1	5	347.51	7.70
8	-1	-5	52.76	2.58
-8	-1	5	57.26	4.05
8	-1	-5	56.31	2.83
8	1	-5	59.27	4.01
8	1	-5	63.80	4.15
-8	1	5	59.36	2.71
7	-1	-5	652.91	11.57
7	-1	-5	675.16	11.38
-7	-1	5	660.92	14.67
7	1	-5	663.15	14.46
7	1	-5	651.04	14.26
-7	1	5	683.61	11.46
6	-1	-5	10.65	1.37
6	-1	-5	10.99	1.22
-6	-1	5	9.71	1.93
-6	1	5	11.20	1.30
6	1	-5	10.71	2.09
6	1	-5	8.80	1.83
-5	-1	5	366.91	9.43
5	-1	-5	355.25	7.00
5	1	-5	373.54	9.37
-5	1	5	360.73	7.17
5	1	-5	363.77	9.16
-5	1	5	360.26	6.86
-4	-1	5	3.92	1.30
4	1	-5	4.16	1.39
-4	1	5	4.56	0.96
4	1	-5	2.90	1.18
-4	1	5	2.60	0.74
-3	-1	5	120.82	4.75
3	1	-5	133.10	4.86
-3	1	5	127.23	3.62
3	1	-5	118.00	4.55
-3	1	5	122.13	3.44
-2	-1	5	6.91	1.50
2	-1	-5	5.71	1.04
-2	1	5	4.33	0.93
2	1	-5	5.19	1.31
2	1	-5	5.66	1.27
-2	1	5	8.41	1.21
1	-1	-5	475.15	9.07
-1	-1	5	469.81	10.10
1	1	-5	461.36	9.66
-1	1	5	473.38	9.02
-1	1	5	471.68	8.88
1	1	-5	462.77	9.97

0	-1	5	1684.71	26.63
0	-1	-5	1701.33	25.92
0	1	-5	1750.35	26.69
0	1	5	1742.90	25.94
0	1	5	1720.99	25.59
1	-1	5	81.82	3.63
-1	-1	-5	80.25	3.56
1	1	5	92.59	3.58
-1	1	-5	80.86	3.68
-2	-1	-5	7.93	1.55
2	-1	5	9.04	1.52
-2	1	-5	9.30	1.58
2	1	5	10.10	1.59
-3	-1	-5	139.41	5.28
3	-1	5	147.78	4.80
-3	1	-5	141.25	5.09
3	1	5	145.29	5.13
-4	-1	-5	250.30	7.81
4	-1	5	253.57	6.84
-4	1	-5	260.37	7.36
4	1	5	256.72	7.70
4	1	5	273.87	7.65
5	-1	5	229.28	6.54
-5	1	-5	219.36	6.93
5	1	5	229.61	7.54
5	1	5	222.47	7.22
-6	-1	-5	375.37	10.90
6	-1	5	404.45	9.50
6	1	5	394.89	10.51
6	1	5	391.30	10.75
-6	1	-5	387.83	10.04
-7	-1	-5	802.99	18.02
7	-1	5	805.23	15.63
-7	1	-5	808.16	16.52
7	1	5	794.68	17.12
8	-1	5	-0.64	1.01
-8	-1	-5	-1.02	1.05
8	1	5	1.63	1.38
-8	1	-5	1.06	1.16
9	-1	5	589.53	13.37
9	-1	5	581.78	13.65
-9	-1	-5	596.16	15.98
-9	1	-5	567.74	14.06
9	1	5	570.64	14.81
-10	-1	-5	135.74	7.49
10	-1	5	146.26	6.25
10	1	5	148.92	7.30
-10	1	-5	135.55	6.53
11	-1	5	35.76	3.97
-11	-1	-5	39.88	5.21

11	1	5	40.06	4.75
-11	1	-5	43.54	4.76
-12	-1	-5	157.18	8.69
12	-1	5	165.79	7.22
-12	1	-5	166.79	7.86
12	1	5	165.45	8.45
-13	-1	-5	128.97	8.19
13	-1	5	138.41	6.85
-13	1	-5	142.58	7.57
13	1	5	135.33	7.90
-14	-1	-5	31.96	5.27
14	-1	5	35.59	4.46
-14	1	-5	36.18	4.91
14	1	5	35.93	5.12
15	-1	5	4.66	2.39
-15	-1	-5	6.41	2.82
-15	1	-5	6.05	2.55
15	1	5	11.10	3.19
-16	-1	-5	-0.39	2.13
16	-1	5	3.08	2.30
-16	1	-5	0.29	2.04
16	1	5	-0.63	2.04
-17	-1	-5	27.33	5.55
17	-1	5	37.77	5.17
17	1	5	32.00	5.44
-17	1	-5	23.74	4.54
-16	-2	5	23.40	5.17
-16	-2	5	17.22	4.31
16	2	-5	17.08	4.31
-15	-2	5	1.23	2.34
15	2	-5	3.12	2.50
-14	-2	5	46.09	5.67
14	2	-5	59.45	6.61
-13	-2	5	47.05	5.72
13	2	-5	43.15	5.62
-12	-2	5	1.04	1.88
12	2	-5	2.63	2.33
-11	-2	5	2.73	1.93
11	2	-5	5.25	2.36
-10	-2	5	7.52	2.44
10	2	-5	2.48	2.07
-9	-2	5	21.26	3.41
9	2	-5	20.36	3.45
-8	-2	5	-0.54	1.14
8	2	-5	0.56	1.46
-7	-2	5	43.59	4.48
7	2	-5	42.28	4.29
-6	-2	5	509.46	13.21
6	2	-5	495.18	12.65
-5	-2	5	424.31	11.55

5	2	-5	410.61	11.18
5	2	-5	422.77	11.15
-4	-2	5	60.15	4.03
4	2	-5	65.50	4.10
-4	2	5	65.24	1.95
4	2	-5	63.87	3.97
-4	2	5	63.70	2.00
-4	2	5	60.96	1.97
3	-2	-5	6.40	0.94
-3	-2	5	3.56	1.47
3	2	-5	5.76	1.62
-3	2	5	6.55	0.98
3	2	-5	6.11	1.61
-3	2	5	6.58	0.97
-2	-2	5	284.38	8.51
2	-2	-5	302.06	6.44
-2	2	5	298.23	6.38
-2	2	5	306.01	6.61
-2	2	5	294.86	6.40
2	2	-5	288.26	8.15
2	2	-5	292.94	8.40
1	-2	-5	52.01	2.70
-1	-2	5	48.23	3.34
1	2	-5	56.18	3.52
-1	2	5	49.23	2.60
-1	2	5	50.92	2.71
-1	2	5	55.29	2.66
1	2	-5	51.82	3.40
0	-2	-5	432.38	9.46
0	-2	5	428.89	10.46
0	2	-5	427.71	10.25
0	2	5	433.03	9.20
0	2	5	422.39	9.43
0	2	-5	446.41	10.01
1	-2	5	211.44	6.80
-1	-2	-5	208.39	6.53
1	2	5	226.94	6.45
-1	2	-5	228.79	6.92
1	2	5	215.39	6.59
-1	2	-5	225.20	6.49
2	-2	5	7.63	1.63
-2	-2	-5	4.16	1.43
2	2	5	5.45	1.48
-2	2	-5	5.51	1.44
-2	2	-5	5.81	1.46
2	2	5	4.42	1.38
-3	-2	-5	142.79	5.97
3	-2	5	145.84	5.41
3	2	5	139.27	5.55
3	2	5	135.19	5.66

-4	-2	-5	1.74	1.27
4	-2	5	-0.29	0.83
4	2	5	-1.45	0.79
-4	2	-5	-0.19	0.84
4	2	5	-0.40	0.97
-5	-2	-5	25.54	3.48
5	-2	5	26.38	2.71
5	2	5	26.59	3.23
-5	2	-5	29.45	2.57
5	2	5	29.80	3.51
6	-2	5	21.34	2.53
-6	-2	-5	18.33	3.13
6	2	5	20.79	3.06
-6	2	-5	17.33	2.47
-7	-2	-5	73.14	5.07
7	-2	5	53.70	3.50
7	2	5	56.69	4.43
-7	2	-5	58.17	3.81
8	-2	5	69.10	4.05
-8	-2	-5	74.73	5.60
-8	2	-5	72.36	4.32
8	2	5	70.95	5.15
-9	-2	-5	277.99	11.13
9	-2	5	284.76	8.25
9	2	5	271.76	10.45
-9	2	-5	289.09	9.04
-10	-2	-5	-0.42	1.76
10	-2	5	-1.07	1.25
10	2	5	0.32	2.01
-10	2	-5	0.76	1.32
11	-2	5	55.69	3.99
-11	-2	-5	53.24	6.29
-11	2	-5	57.51	4.35
11	2	5	59.33	6.39
-12	-2	-5	2.62	2.42
12	-2	5	2.38	1.71
12	2	5	5.65	2.84
-12	2	-5	1.21	1.69
13	-2	5	5.78	2.00
-13	-2	-5	2.39	2.45
-13	2	-5	2.43	1.88
13	2	5	2.63	2.64
14	-2	5	5.57	2.14
-14	-2	-5	14.41	4.03
-14	2	-5	6.25	2.48
14	2	5	3.37	2.78
-15	-2	-5	1.33	2.49
15	2	5	3.58	2.94
-16	-2	-5	38.88	6.64
16	2	5	49.30	6.88

-15	-3	5	-1.36	2.50
15	3	-5	-2.84	2.25
-14	-3	5	12.03	3.73
14	3	-5	4.89	3.22
-13	-3	5	63.37	7.04
13	3	-5	65.30	7.20
-12	-3	5	-1.77	1.83
12	3	-5	0.80	2.41
-11	-3	5	17.12	3.80
11	3	-5	15.96	3.90
-10	-3	5	97.41	6.93
10	3	-5	99.43	6.86
-9	-3	5	7.24	2.65
9	3	-5	11.86	3.25
-8	-3	5	105.70	6.78
8	3	-5	116.67	6.92
-7	-3	5	84.75	5.93
7	3	-5	79.79	5.64
-6	-3	5	24.94	3.76
6	3	-5	19.30	3.35
-5	-3	5	114.62	6.39
5	3	-5	110.66	6.13
4	-3	-5	113.63	2.64
-4	-3	5	120.95	6.30
4	3	-5	116.26	5.94
-4	3	5	112.34	3.16
3	-3	-5	3.01	0.88
-3	-3	5	3.95	1.75
3	3	-5	3.62	1.69
-3	3	5	4.26	1.03
-3	3	5	3.46	0.98
-2	-3	5	143.61	6.55
2	-3	-5	149.93	4.73
-2	3	5	157.26	4.94
2	3	-5	151.57	6.35
-2	3	5	152.66	4.69
-1	-3	5	145.92	6.54
1	-3	-5	153.43	5.23
-1	3	5	151.63	5.08
-1	3	5	160.74	5.43
1	3	-5	159.92	6.42
0	-3	-5	43.76	3.17
0	-3	5	43.50	3.53
0	3	5	45.29	3.07
0	3	5	43.14	3.08
0	3	-5	44.84	3.40
0	3	-5	41.42	3.45
-1	-3	-5	179.73	6.60
1	-3	5	174.70	6.77
-1	3	-5	184.71	6.46

1	3	5	172.72	6.22
1	3	5	184.34	6.59
-1	3	-5	163.04	6.47
-2	-3	-5	29.78	3.43
2	-3	5	27.21	3.07
-2	3	-5	27.42	2.96
2	3	5	27.73	3.28
2	3	5	27.62	3.27
-2	3	-5	32.71	3.37
-3	-3	-5	90.44	5.21
3	-3	5	90.55	4.65
-3	3	-5	88.99	4.45
-3	3	-5	92.41	4.73
3	3	5	92.17	5.00
-4	-3	-5	95.31	5.59
4	-3	5	85.19	4.55
-4	3	-5	81.55	4.17
4	3	5	78.07	4.90
-5	-3	-5	53.63	4.62
5	-3	5	54.06	3.69
5	3	5	54.90	4.45
-5	3	-5	54.60	3.42
6	-3	5	114.63	5.24
-6	-3	-5	109.80	6.63
-6	3	-5	110.99	4.71
6	3	5	103.96	6.20
7	-3	5	306.07	8.89
-7	-3	-5	305.27	11.60
-7	3	-5	288.73	8.02
7	3	5	285.15	10.76
-8	-3	-5	258.90	11.03
8	-3	5	248.58	7.96
8	3	5	261.00	10.53
9	-3	5	376.12	10.20
-9	-3	-5	375.37	14.01
9	3	5	391.94	13.56
-10	-3	-5	326.58	13.39
10	-3	5	322.08	9.41
-10	3	-5	323.17	9.70
10	3	5	325.00	12.73
-11	-3	-5	40.72	5.84
11	-3	5	40.81	3.55
-11	3	-5	43.36	4.33
11	3	5	43.11	5.90
-12	-3	-5	205.78	11.38
12	-3	5	207.67	7.56
12	3	5	207.31	10.88
-13	-3	-5	37.57	6.26
13	3	5	33.28	5.66
-14	-3	-5	55.58	7.74

14	3	5	53.83	7.05
-15	-3	-5	0.75	2.85
15	3	5	2.73	3.53
-13	-4	5	1.72	2.86
13	4	-5	-0.04	2.69
-12	-4	5	6.87	3.26
12	4	-5	6.96	3.49
-11	-4	5	12.25	3.69
11	4	-5	8.11	3.38
-10	-4	5	5.46	2.83
10	4	-5	9.01	3.25
-9	-4	5	-2.14	1.80
9	4	-5	1.66	2.42
-8	-4	5	50.03	6.10
8	4	-5	48.94	5.99
-7	-4	5	88.07	6.71
7	4	-5	96.96	6.96
-6	-4	5	277.17	11.39
6	4	-5	248.82	10.49
-5	-4	5	111.77	7.07
5	4	-5	114.84	6.99
4	-4	-5	8.08	1.08
-4	-4	5	10.95	2.84
4	4	-5	7.73	2.58
-4	4	5	8.36	1.43
-3	-4	5	787.01	19.54
3	-4	-5	818.37	14.23
-3	4	5	824.09	14.81
3	4	-5	808.56	18.94
2	-4	-5	68.90	3.77
-2	-4	5	74.21	5.37
-2	4	5	65.91	3.79
2	4	-5	65.31	5.03
-2	4	5	75.53	3.78
1	-4	-5	105.62	4.96
-1	-4	5	94.51	5.85
1	4	-5	96.46	5.61
-1	4	5	101.37	4.71
-1	4	5	93.32	4.74
0	-4	-5	290.17	8.98
0	-4	5	292.96	10.16
0	4	5	305.42	8.87
0	4	5	288.00	8.90
0	4	-5	277.82	9.38
1	-4	5	3.87	1.84
-1	-4	-5	4.55	1.76
1	4	5	3.69	1.63
-1	4	-5	2.20	1.67
2	-4	5	244.84	8.82
-2	-4	-5	232.90	8.86

2	4	5	236.45	8.56
-2	4	-5	240.98	8.17
3	-4	5	70.42	4.70
-3	-4	-5	65.22	4.91
-3	-4	-5	72.48	5.29
3	4	5	77.77	5.11
-3	4	-5	68.52	4.36
-4	-4	-5	127.00	7.00
-4	-4	-5	118.56	6.86
4	-4	5	130.11	6.12
4	4	5	122.22	6.80
-4	4	-5	127.38	5.62
-5	-4	-5	60.75	5.40
5	-4	5	54.83	4.14
-5	-4	-5	50.22	5.34
-5	4	-5	51.19	3.68
5	4	5	57.20	5.77
-6	-4	-5	18.35	3.60
6	-4	5	22.79	3.06
-6	-4	-5	28.45	4.57
6	4	5	24.19	4.18
-6	4	-5	27.91	3.12
-7	-4	-5	30.08	4.83
7	-4	5	36.29	3.69
7	4	5	37.43	5.01
8	-4	5	117.45	5.57
-8	-4	-5	122.92	8.25
8	4	5	111.38	7.63
9	-4	5	37.32	3.67
-9	-4	-5	40.57	6.11
9	4	5	35.70	5.42
-10	-4	-5	3.21	2.79
10	4	5	7.86	3.41
-11	-4	-5	35.35	5.87
11	4	5	42.53	6.29
-12	-4	-5	7.40	3.41
12	4	5	10.36	3.85
-13	-4	-5	44.54	7.19
13	4	5	50.53	7.27
-11	-5	5	4.40	3.31
11	5	-5	-0.56	2.68
-10	-5	5	-0.11	2.42
10	5	-5	0.57	2.69
-9	-5	5	23.35	4.95
9	5	-5	35.49	5.96
-8	-5	5	38.02	5.98
8	5	-5	43.68	6.41
-7	-5	5	-1.77	2.12
7	5	-5	0.24	2.34
-6	-5	5	13.14	3.63

6	5	-5	8.86	3.10
-5	-5	5	7.65	3.07
5	5	-5	9.79	2.97
-4	-5	5	-1.21	2.02
4	5	-5	-1.84	1.72
-3	-5	5	9.25	2.97
3	5	-5	3.94	2.44
2	-5	-5	53.68	3.80
-2	-5	5	59.14	6.21
2	5	-5	45.81	5.43
-1	-5	5	-0.64	1.67
1	-5	-5	-1.22	1.35
1	5	-5	0.52	1.62
-1	5	5	1.19	1.61
0	-5	5	0.38	1.62
0	-5	-5	0.81	1.72
0	5	-5	0.73	1.71
0	5	5	2.17	1.90
1	-5	5	8.27	2.54
-1	-5	-5	5.98	2.25
-1	5	-5	11.94	2.82
1	5	5	11.04	2.67
-2	-5	-5	3.72	2.13
2	-5	5	-0.99	1.44
2	5	5	2.11	1.91
-2	5	-5	0.72	1.74
-3	-5	-5	7.87	2.77
3	-5	5	5.55	2.13
3	5	5	8.18	2.80
-3	5	-5	9.49	2.43
-4	-5	-5	39.47	5.10
4	-5	5	34.01	4.14
-4	5	-5	46.26	4.44
4	5	5	40.96	5.28
-5	-5	-5	13.26	3.47
5	-5	5	9.69	2.51
5	5	5	13.16	3.44
-6	-5	-5	-1.42	1.72
6	-5	5	1.50	1.58
6	5	5	-0.91	1.87
-7	-5	-5	12.36	3.50
7	5	5	7.79	3.16
-8	-5	-5	-0.45	1.85
8	5	5	2.15	2.72
-9	-5	-5	1.71	2.50
9	5	5	-1.04	2.36
-10	-5	-5	19.08	4.79
10	5	5	19.21	4.94
-6	-6	5	38.41	6.11
6	6	-5	49.43	6.77

-5	-6	5	13.86	4.03
5	6	-5	14.49	4.23
-4	-6	5	1.18	2.46
4	6	-5	2.77	2.56
-3	-6	5	63.07	7.18
3	6	-5	66.72	7.10
-2	-6	5	18.06	4.12
2	6	-5	8.70	3.22
-1	-6	5	19.13	4.07
1	6	-5	23.14	4.23
0	-6	5	65.69	6.86
0	6	-5	49.10	5.70
1	-6	5	1.56	2.13
-1	-6	-5	1.30	2.13
-1	6	-5	0.02	1.74
2	-6	5	120.78	7.46
-2	-6	-5	105.35	7.14
2	6	5	104.53	6.81
-3	-6	-5	11.37	3.39
3	-6	5	9.04	2.97
3	6	5	12.46	3.48
-4	-6	-5	14.03	3.78
4	6	5	20.49	4.27
-5	-6	-5	55.00	6.96
5	6	5	51.84	6.46
-6	-6	-5	3.33	2.69
17	0	-6	89.34	6.21
-16	0	6	48.99	5.57
16	0	-6	42.77	5.00
-15	0	6	56.82	5.13
15	0	-6	49.25	5.22
-15	0	6	62.51	4.87
14	0	-6	5.75	2.38
-14	0	6	3.58	1.94
-14	0	6	5.26	2.34
-13	0	6	360.10	11.06
13	0	-6	369.91	11.27
12	0	-6	80.85	5.23
-12	0	6	80.97	5.19
11	0	-6	674.50	15.29
-11	0	6	685.91	15.34
11	0	-6	695.54	15.21
10	0	-6	935.99	18.16
10	0	-6	921.83	18.24
-10	0	6	917.70	18.29
9	0	-6	-1.80	1.13
-9	0	6	-0.67	1.10
9	0	-6	2.48	1.31
8	0	-6	215.85	6.89
8	0	-6	228.51	6.96

-8	0	6	229.33	7.09
-7	0	6	348.15	8.83
7	0	-6	357.78	8.84
7	0	-6	356.70	8.77
6	0	-6	394.32	9.30
6	0	-6	424.40	9.48
-6	0	6	415.83	9.51
5	0	-6	2383.65	35.29
-5	0	6	2390.33	35.50
5	0	-6	2386.45	35.30
4	0	-6	280.88	7.05
4	0	-6	275.19	6.95
-4	0	6	274.18	7.07
3	0	-6	2076.13	30.65
3	0	-6	2038.61	30.66
-3	0	6	2061.28	30.82
-2	0	6	605.56	11.63
2	0	-6	605.43	11.60
1	0	-6	222.09	6.24
-1	0	6	234.29	6.20
0	0	6	957.11	16.22
0	0	-6	941.91	16.67
1	0	6	2619.03	38.33
-1	0	-6	2650.07	39.00
-2	0	-6	1312.97	21.92
2	0	6	1270.94	21.16
-3	0	-6	138.93	5.32
3	0	6	132.06	4.88
-4	0	-6	984.63	18.66
-5	0	-6	288.78	8.82
5	0	6	308.45	8.53
6	0	6	1802.32	29.71
-6	0	-6	1795.91	30.66
-7	0	-6	144.22	6.50
7	0	6	149.45	6.17
-8	0	-6	135.99	6.57
8	0	6	130.62	6.12
-9	0	-6	1301.15	25.70
9	0	6	1276.53	24.52
-10	0	-6	12.61	2.91
10	0	6	11.37	2.76
11	0	6	209.35	8.68
-11	0	-6	202.27	9.06
-12	0	-6	409.52	13.59
12	0	6	398.60	12.76
-13	0	-6	170.11	8.97
13	0	6	158.54	8.16
-14	0	-6	161.16	8.98
14	0	6	143.43	8.01
15	0	6	76.71	6.18

-15	0	-6	80.06	6.60
-16	0	-6	6.61	3.19
16	0	6	3.59	2.66
17	0	6	25.25	4.58
-17	0	-6	36.94	5.93
-17	-1	6	7.82	3.27
17	1	-6	12.89	3.73
-16	-1	6	4.00	2.59
-16	-1	6	4.23	2.68
16	1	-6	2.80	2.59
-15	-1	6	11.07	3.32
15	1	-6	9.92	3.11
-14	-1	6	9.87	2.90
14	1	-6	5.99	2.82
13	-1	-6	0.99	1.59
-13	-1	6	-1.76	1.66
13	1	-6	-0.83	1.92
-13	1	6	0.35	1.32
12	-1	-6	42.20	3.27
-12	-1	6	49.45	5.11
12	-1	-6	38.98	3.32
-12	1	6	38.72	3.11
12	1	-6	45.10	5.00
-12	1	6	40.74	3.40
11	-1	-6	64.60	3.70
-11	-1	6	64.06	5.15
11	-1	-6	63.75	3.86
-11	1	6	68.02	3.72
11	1	-6	71.76	5.17
-10	-1	6	11.87	2.56
10	-1	-6	8.18	1.61
10	-1	-6	8.95	1.90
-10	1	6	10.03	1.69
10	1	-6	7.62	2.42
9	-1	-6	2.46	1.26
9	-1	-6	1.68	0.97
-9	-1	6	-0.66	1.22
9	1	-6	-1.23	1.27
-9	1	6	0.70	0.88
8	-1	-6	13.85	1.67
-8	-1	6	17.00	2.67
8	-1	-6	16.11	1.97
-8	1	6	15.06	1.73
8	1	-6	16.38	2.73
8	1	-6	15.69	2.61
7	-1	-6	244.48	6.09
7	-1	-6	243.36	5.86
-7	-1	6	258.66	8.33
7	1	-6	230.84	7.87
-7	1	6	255.61	5.98

7	1	-6	231.61	7.90
6	-1	-6	266.75	6.12
6	-1	-6	266.37	6.30
-6	-1	6	267.99	8.31
6	1	-6	260.52	8.01
6	1	-6	281.69	8.34
-6	1	6	276.59	6.56
-6	1	6	270.91	6.13
5	-1	-6	59.91	2.67
-5	-1	6	50.91	3.49
-5	1	6	60.87	2.79
-5	1	6	57.63	2.53
5	1	-6	48.58	3.55
5	1	-6	50.29	3.48
4	-1	-6	657.75	11.50
-4	-1	6	639.39	13.76
-4	1	6	669.46	11.49
4	1	-6	673.71	13.63
-4	1	6	665.89	11.72
4	1	-6	647.07	13.62
-3	-1	6	633.74	13.16
-3	1	6	627.87	11.26
3	1	-6	616.20	12.89
3	1	-6	627.01	12.72
-3	1	6	601.02	10.93
2	-1	-6	74.56	3.17
-2	-1	6	76.02	3.86
2	1	-6	76.97	3.77
-2	1	6	82.15	3.27
2	1	-6	85.18	3.99
-1	-1	6	226.83	6.81
1	-1	-6	229.19	6.07
1	1	-6	233.53	6.78
1	1	-6	229.57	6.56
-1	1	6	233.51	5.90
0	-1	6	75.13	3.73
0	-1	-6	77.60	3.50
0	1	6	81.70	3.42
0	1	-6	74.03	3.68
1	-1	6	7.98	1.62
-1	-1	-6	8.47	1.58
1	1	6	9.45	1.56
-1	1	-6	7.15	1.50
-2	-1	-6	98.75	4.41
2	-1	6	84.05	3.99
2	1	6	88.86	3.99
-2	1	-6	90.97	4.24
3	-1	6	324.59	8.18
-3	-1	-6	332.88	9.10
3	-1	6	324.68	8.50

-3	1	-6	326.49	8.75
3	1	6	333.13	8.64
-4	-1	-6	669.35	14.75
4	-1	6	676.52	13.45
4	1	6	666.61	14.06
-4	1	-6	666.08	14.07
-5	-1	-6	557.68	13.56
5	-1	6	563.96	12.08
-5	1	-6	558.18	12.77
5	1	6	577.37	13.40
5	1	6	550.19	12.90
6	-1	6	142.64	5.55
-6	1	-6	141.95	5.99
6	1	6	144.58	6.20
-7	-1	-6	370.70	11.42
7	-1	6	372.31	9.79
7	1	6	357.28	10.58
-7	1	-6	344.95	10.18
-8	-1	-6	6.41	2.23
8	-1	6	6.46	1.79
-8	1	-6	5.26	1.82
8	1	6	6.93	2.07
9	-1	6	46.20	3.76
-9	-1	-6	48.45	5.32
9	1	6	43.37	4.64
-9	1	-6	55.66	4.24
-10	-1	-6	55.28	5.89
10	-1	6	48.67	3.97
-10	1	-6	43.57	4.62
10	1	6	46.10	5.05
11	-1	6	6.69	2.25
-11	-1	-6	2.21	2.07
-11	1	-6	4.69	2.02
11	1	6	5.74	2.49
-12	-1	-6	20.16	4.04
12	-1	6	18.52	3.28
12	1	6	16.27	3.57
-12	1	-6	12.91	2.96
-13	-1	-6	51.47	6.35
13	-1	6	58.91	5.59
-13	1	-6	53.38	5.72
13	1	6	59.63	6.24
-14	-1	-6	8.00	3.12
14	-1	6	6.81	2.50
-14	1	-6	12.44	3.15
14	1	6	8.39	3.09
-15	-1	-6	2.27	2.31
15	-1	6	1.36	2.14
-15	1	-6	0.84	2.07
15	1	6	2.37	2.34

16	-1	6	36.13	4.95
-16	-1	-6	17.35	4.40
16	1	6	24.87	4.93
-16	1	-6	26.66	4.68
-16	-2	6	0.75	2.42
-16	-2	6	-1.88	2.31
16	2	-6	1.88	2.80
-15	-2	6	113.53	8.10
15	2	-6	105.17	7.74
-14	-2	6	1.47	2.30
14	2	-6	7.48	2.86
-13	-2	6	41.91	5.46
13	2	-6	40.71	5.44
-12	-2	6	93.04	6.52
12	2	-6	94.92	6.71
-11	-2	6	34.58	4.71
11	2	-6	30.37	4.35
-10	-2	6	120.24	6.93
10	2	-6	112.78	6.79
-9	-2	6	53.62	4.68
9	2	-6	63.15	4.93
-9	2	6	56.61	1.77
-8	-2	6	118.24	6.48
-7	-2	6	0.80	1.28
7	2	-6	2.57	1.66
-6	-2	6	35.52	3.98
6	2	-6	47.15	3.91
-5	-2	6	46.28	3.83
-5	2	6	53.01	1.97
5	2	-6	58.05	4.04
-5	2	6	54.20	1.86
-5	2	6	52.81	1.87
-4	-2	6	27.98	3.22
-4	2	6	31.95	1.78
4	2	-6	28.99	3.24
-4	2	6	32.35	1.72
-4	2	6	31.88	1.73
4	2	-6	29.29	3.29
3	-2	-6	276.28	6.11
-3	-2	6	266.49	8.64
-3	2	6	278.87	6.11
-3	2	6	278.09	6.19
3	2	-6	277.30	8.56
-3	2	6	276.83	6.14
3	2	-6	268.82	8.35
-2	-2	6	207.52	7.25
2	-2	-6	193.31	5.24
-2	2	6	188.86	5.12
-2	2	6	180.06	5.08
2	2	-6	187.16	6.78

-2	2	6	189.60	5.26
2	2	-6	180.24	6.51
1	-2	-6	70.58	3.33
-1	-2	6	75.32	4.24
-1	2	6	62.26	3.09
-1	2	6	70.30	3.37
1	2	-6	68.97	3.91
1	2	-6	80.09	4.31
0	-2	-6	644.41	12.87
0	-2	6	654.71	14.15
0	2	6	666.58	12.72
0	2	-6	620.82	13.29
0	2	-6	643.22	13.84
0	2	6	655.86	12.98
1	-2	6	850.14	16.97
-1	-2	-6	887.98	16.68
-1	2	-6	878.61	17.01
1	2	6	881.82	16.63
-1	2	-6	862.87	16.36
1	2	6	857.38	16.09
2	-2	6	970.77	18.41
-2	-2	-6	962.79	18.56
2	2	6	974.60	18.05
-2	2	-6	950.90	17.69
2	2	6	1002.84	18.65
-3	-2	-6	18.00	2.65
3	-2	6	17.66	2.43
3	2	6	17.12	2.43
3	2	6	22.33	2.88
4	-2	6	88.51	4.49
-4	-2	-6	89.34	5.12
4	2	6	80.76	4.59
-4	2	-6	81.52	4.45
4	2	6	89.45	4.95
5	-2	6	58.82	3.72
-5	-2	-6	51.52	4.20
-5	2	-6	48.95	3.56
5	2	6	49.26	3.88
6	-2	6	9.76	1.91
-6	-2	-6	6.55	2.10
6	2	6	6.85	2.09
-6	2	-6	7.98	1.89
-7	-2	-6	54.16	4.73
7	-2	6	46.29	3.54
-7	2	-6	46.44	3.62
7	2	6	55.11	4.48
-8	-2	-6	585.21	16.52
8	-2	6	588.51	13.66
-8	2	-6	564.60	13.89
8	2	6	570.59	15.58

-9	-2	-6	41.18	5.42
9	-2	6	35.91	3.64
9	2	6	29.81	4.30
-9	2	-6	31.14	3.80
10	-2	6	100.84	5.23
-10	-2	-6	104.57	7.27
10	2	6	93.33	6.69
-10	2	-6	89.48	5.42
-11	-2	-6	386.70	14.40
11	-2	6	357.44	10.48
11	2	6	385.39	13.68
-11	2	-6	357.54	11.38
-12	-2	-6	47.82	6.46
12	-2	6	40.20	4.35
12	2	6	34.89	5.19
-12	2	-6	45.86	4.95
-13	-2	-6	0.35	2.21
13	-2	6	1.98	1.89
-13	2	-6	1.39	1.75
13	2	6	0.02	2.17
14	-2	6	15.30	3.11
-14	-2	-6	21.51	4.72
-14	2	-6	20.22	3.73
14	2	6	20.39	4.63
-15	-2	-6	16.83	4.59
15	2	6	11.60	3.68
-16	-2	-6	26.82	5.53
16	2	6	20.08	5.01
-15	-3	6	7.57	3.38
15	3	-6	1.39	2.99
-14	-3	6	2.21	2.68
14	3	-6	9.07	3.67
-13	-3	6	1.83	2.35
13	3	-6	-0.20	2.49
-12	-3	6	18.23	3.94
12	3	-6	6.72	3.16
-11	-3	6	3.53	2.53
11	3	-6	0.36	2.21
-10	-3	6	3.74	2.47
10	3	-6	-2.63	1.64
-9	-3	6	77.71	6.13
9	3	-6	80.01	6.19
-8	-3	6	20.51	3.74
8	3	-6	22.84	3.95
-7	-3	6	1.66	1.95
7	3	-6	0.96	1.87
-6	-3	6	15.52	3.15
6	3	-6	17.35	3.40
-5	-3	6	39.70	4.48
5	3	-6	47.05	4.83

-4	-3	6	78.37	5.31
4	-3	-6	81.36	2.57
4	3	-6	79.87	5.18
-4	3	6	76.10	2.76
3	-3	-6	171.33	4.71
-3	-3	6	174.55	7.68
-3	3	6	166.22	4.92
-3	3	6	180.21	4.90
3	3	-6	164.18	7.06
-2	-3	6	84.73	5.19
2	-3	-6	80.42	3.52
-2	3	6	77.57	3.60
-2	3	6	75.14	3.46
2	3	-6	80.39	4.92
-1	-3	6	0.82	1.23
1	-3	-6	-0.09	0.84
1	3	-6	2.00	1.41
-1	3	6	-0.67	0.81
-1	3	6	-0.37	0.81
0	-3	-6	130.51	5.33
0	-3	6	129.54	6.13
0	3	6	130.46	5.16
0	3	-6	116.65	5.50
0	3	6	130.00	5.31
1	-3	6	9.45	2.12
-1	-3	-6	8.79	1.98
-1	3	-6	9.22	2.03
1	3	6	11.56	2.12
1	3	6	13.46	2.29
-1	3	-6	14.96	2.46
2	-3	6	142.35	6.25
-2	-3	-6	143.09	6.39
2	3	6	126.35	5.83
-2	3	-6	141.34	5.85
-2	3	-6	141.84	6.09
2	3	6	123.34	5.76
-3	-3	-6	41.61	4.46
3	-3	6	38.33	3.32
-3	3	-6	39.56	3.72
3	3	6	38.54	4.00
-4	-3	-6	220.83	8.88
4	-3	6	244.77	8.16
4	3	6	237.64	8.75
-4	3	-6	234.70	7.44
5	-3	6	47.43	3.69
-5	-3	-6	49.59	5.34
-5	3	-6	41.62	3.29
5	3	6	42.95	4.66
-6	-3	-6	17.78	3.52
6	-3	6	17.57	2.73

-6	3	-6	22.18	2.89
6	3	6	22.27	3.63
7	-3	6	192.17	7.21
-7	-3	-6	198.60	9.42
7	3	6	192.83	8.88
8	-3	6	18.48	2.79
-8	-3	-6	20.04	4.02
8	3	6	16.52	3.56
9	-3	6	21.09	3.03
-9	-3	-6	15.68	3.84
9	3	6	14.19	3.61
-9	3	-6	23.12	3.34
-10	-3	-6	61.93	7.27
10	-3	6	68.35	4.60
-10	3	-6	66.40	4.66
10	3	6	68.69	7.08
11	-3	6	118.24	5.95
-11	-3	-6	141.42	9.40
11	3	6	133.07	8.57
-11	3	-6	133.87	6.60
12	-3	6	11.87	2.51
-12	-3	-6	9.81	3.63
12	3	6	2.00	2.77
-13	-3	-6	34.20	6.12
13	3	6	27.94	5.42
-14	-3	-6	80.72	9.44
14	3	6	72.93	8.26
-15	-3	-6	-2.31	2.41
-13	-4	6	35.70	5.92
13	4	-6	26.61	5.45
-12	-4	6	-1.57	2.57
12	4	-6	1.08	2.59
-11	-4	6	20.39	4.53
11	4	-6	13.13	4.06
-10	-4	6	21.94	4.45
10	4	-6	19.79	4.35
-9	-4	6	31.25	5.12
9	4	-6	19.33	4.25
-8	-4	6	2.23	2.23
8	4	-6	-1.21	2.02
-7	-4	6	15.80	3.64
7	4	-6	13.59	3.64
-6	-4	6	29.79	4.59
6	4	-6	27.74	4.38
-5	-4	6	168.15	8.76
5	4	-6	173.69	8.64
4	-4	-6	93.66	2.96
-4	-4	6	92.26	6.35
4	4	-6	90.50	6.11
3	-4	-6	16.40	2.10

-3	-4	6	12.06	3.01
3	4	-6	22.93	3.86
-3	4	6	17.89	2.24
-2	-4	6	378.03	12.62
2	-4	-6	392.06	9.41
2	4	-6	361.99	11.78
-2	4	6	376.52	9.23
-2	4	6	379.48	9.51
-1	-4	6	34.63	4.33
1	-4	-6	35.21	3.52
-1	4	6	39.89	3.67
-1	4	6	36.65	3.08
1	4	-6	39.20	4.46
0	-4	6	6.67	2.28
0	-4	-6	10.05	2.36
0	4	6	9.11	2.10
0	4	6	9.74	2.16
0	4	-6	10.26	2.53
-1	-4	-6	14.53	2.76
1	-4	6	13.16	2.86
-1	4	-6	18.04	2.96
1	4	6	17.22	2.87
2	-4	6	2.85	1.81
-2	-4	-6	-2.17	1.28
-2	4	-6	0.13	1.47
2	4	6	1.02	1.68
3	-4	6	60.78	4.58
-3	-4	-6	66.40	5.19
-3	4	-6	71.64	4.62
3	4	6	57.91	4.63
4	-4	6	44.72	4.44
-4	-4	-6	57.60	5.99
-4	4	-6	40.87	4.07
4	4	6	39.96	4.72
5	-4	6	16.14	2.87
-5	-4	-6	15.52	3.53
5	4	6	9.43	3.00
-5	4	-6	21.99	3.03
-6	-4	-6	99.08	6.92
6	-4	6	102.27	5.59
6	4	6	97.74	6.66
-6	4	-6	104.29	5.20
7	-4	6	47.67	3.93
-7	-4	-6	55.63	6.41
7	4	6	53.26	6.07
-8	-4	-6	-1.68	1.75
8	-4	6	-1.22	1.24
8	4	6	-1.67	1.73
9	-4	6	16.83	2.79
-9	-4	-6	16.14	4.12

9	4	6	20.64	4.48
-10	-4	-6	8.01	3.32
10	4	6	6.21	3.09
-11	-4	-6	32.36	5.93
11	4	6	42.93	6.24
-12	-4	-6	7.01	3.34
12	4	6	0.99	2.58
-13	-4	-6	3.33	3.07
13	4	6	6.09	3.41
-10	-5	6	4.02	3.03
10	5	-6	0.50	2.72
-9	-5	6	28.39	5.44
9	5	-6	28.33	5.33
-8	-5	6	3.88	2.76
8	5	-6	10.97	3.75
-7	-5	6	2.83	2.77
7	5	-6	-0.09	2.07
-6	-5	6	56.08	6.64
6	5	-6	58.54	6.72
-5	-5	6	40.51	5.74
5	5	-6	34.47	5.10
-4	-5	6	67.43	7.02
4	5	-6	54.28	6.04
-3	-5	6	233.88	10.84
3	5	-6	231.29	10.27
2	-5	-6	35.66	3.56
-2	-5	6	34.01	4.98
2	5	-6	40.44	5.12
-1	-5	6	81.27	6.27
1	-5	-6	70.90	4.60
1	5	-6	82.92	5.92
0	-5	-6	114.72	6.16
0	-5	6	106.64	6.94
0	5	6	104.99	5.72
0	5	-6	99.90	6.19
-1	-5	-6	12.81	2.85
1	-5	6	9.73	2.72
-1	5	-6	8.39	2.58
1	5	6	10.98	2.72
2	-5	6	62.60	5.23
-2	-5	-6	55.35	4.98
2	5	6	60.98	5.02
-2	5	-6	53.05	5.21
3	-5	6	3.08	2.01
-3	-5	-6	4.41	2.39
3	5	6	2.23	2.02
-3	5	-6	3.49	1.85
4	-5	6	19.69	3.37
-4	-5	-6	19.86	3.92
4	5	6	18.98	3.90

-4	5	-6	18.46	3.16
-5	-5	-6	31.26	4.98
5	-5	6	40.22	4.54
5	5	6	40.13	5.41
-6	-5	-6	-1.70	1.92
6	-5	6	4.53	1.97
6	5	6	4.33	2.75
-7	-5	-6	9.09	3.48
7	5	6	12.39	3.62
-8	-5	-6	14.48	4.14
8	5	6	8.36	3.56
-9	-5	-6	2.32	2.61
9	5	6	3.57	2.88
-10	-5	-6	4.58	3.15
10	5	6	11.10	4.02
-5	-6	6	11.24	3.76
5	6	-6	11.15	3.84
-4	-6	6	2.49	2.86
4	6	-6	6.60	3.07
-3	-6	6	0.03	2.40
3	6	-6	3.73	2.64
-2	-6	6	2.41	2.58
2	6	-6	-0.28	2.02
-1	-6	6	5.57	2.79
1	6	-6	4.87	2.79
0	-6	6	5.71	2.74
0	6	-6	3.41	2.50
-1	-6	-6	1.78	2.14
1	-6	6	5.37	2.84
-1	6	-6	3.77	2.57
2	-6	6	5.32	2.69
-2	-6	-6	0.32	2.33
-2	6	-6	0.69	2.13
2	6	6	4.44	2.47
3	-6	6	11.06	3.25
-3	-6	-6	19.44	4.22
3	6	6	15.61	3.87
-4	-6	-6	8.80	3.41
4	6	6	7.69	3.16
-5	-6	-6	2.16	2.70
5	6	6	6.26	3.21
17	0	-7	0.32	2.28
-16	0	7	0.62	2.01
16	0	-7	-0.82	2.01
15	0	-7	2.00	2.05
-15	0	7	-0.02	1.62
14	0	-7	-2.24	1.62
-14	0	7	-1.52	1.53
13	0	-7	-1.19	1.72
-13	0	7	-1.08	1.51

12	0	-7	-2.49	1.78
-12	0	7	-1.77	1.59
11	0	-7	5.32	2.04
-11	0	7	1.54	1.78
10	0	-7	-1.42	1.39
10	0	-7	-0.77	1.22
-10	0	7	0.69	1.45
-9	0	7	-0.65	1.22
9	0	-7	-1.16	1.13
9	0	-7	-0.04	1.33
8	0	-7	0.66	1.13
8	0	-7	0.10	1.14
-8	0	7	0.08	1.02
-7	0	7	0.79	1.06
7	0	-7	-0.58	0.86
7	0	-7	0.38	0.92
6	0	-7	0.52	0.81
-6	0	7	0.32	0.87
6	0	-7	0.07	0.92
5	0	-7	-0.74	0.82
-5	0	7	0.09	0.91
5	0	-7	0.00	0.93
4	0	-7	0.46	0.87
4	0	-7	-0.28	0.79
-4	0	7	-0.11	0.82
-3	0	7	0.92	0.96
3	0	-7	-1.01	0.70
3	0	-7	-0.50	0.82
-2	0	7	0.32	0.92
1	0	-7	-0.39	0.78
-1	0	7	0.77	1.02
0	0	7	-0.64	0.82
0	0	-7	-0.67	0.80
1	0	7	0.77	0.95
-1	0	-7	0.58	0.97
-2	0	-7	1.51	1.09
2	0	7	0.17	0.88
-3	0	-7	-0.30	0.96
3	0	7	-0.73	0.86
4	0	7	0.06	0.97
-4	0	-7	0.21	1.00
-5	0	-7	0.53	1.16
5	0	7	-1.82	0.85
-6	0	-7	-0.67	1.05
6	0	7	0.81	1.25
-7	0	-7	2.35	1.37
7	0	7	-1.37	1.12
8	0	7	1.01	1.54
-8	0	-7	-1.23	1.09
9	0	7	-0.66	1.38

-9	0	-7	2.38	1.75
-10	0	-7	1.31	1.71
10	0	7	2.79	1.75
-11	0	-7	0.35	1.82
11	0	7	1.22	1.67
12	0	7	-0.74	1.62
-12	0	-7	1.52	2.15
13	0	7	1.88	2.19
-13	0	-7	1.01	2.01
14	0	7	3.11	2.33
-14	0	-7	-0.10	1.86
15	0	7	1.63	2.24
-15	0	-7	-0.81	1.97
-16	0	-7	-1.38	1.90
16	0	7	1.11	2.34
-16	-1	7	25.50	4.45
-16	-1	7	29.59	5.11
16	1	-7	29.36	4.85
-15	-1	7	-1.64	1.98
15	1	-7	-2.55	1.83
-14	-1	7	27.46	4.26
14	1	-7	30.73	4.66
13	-1	-7	75.94	4.41
-13	-1	7	72.02	5.68
13	1	-7	72.53	5.64
-13	1	7	77.55	4.67
-12	-1	7	10.20	2.72
12	-1	-7	11.19	2.33
12	1	-7	10.79	2.90
-12	1	7	11.02	2.08
11	-1	-7	691.46	13.63
-11	-1	7	680.82	17.10
11	-1	-7	704.50	14.18
11	1	-7	712.05	17.24
-11	1	7	705.35	13.79
-10	-1	7	283.80	9.91
10	-1	-7	266.42	7.18
10	-1	-7	273.71	7.54
10	1	-7	263.53	9.42
-10	1	7	273.10	7.22
9	-1	-7	20.89	2.45
-9	-1	7	21.38	3.17
9	-1	-7	20.11	2.18
-9	1	7	22.47	2.25
9	1	-7	23.67	3.43
-8	-1	7	349.92	10.50
8	-1	-7	348.64	8.21
8	-1	-7	355.92	8.02
8	1	-7	359.93	10.48
-8	1	7	349.64	8.01

-7	-1	7	1.82	1.40
7	-1	-7	0.26	0.92
7	-1	-7	-0.42	0.72
7	1	-7	-0.51	1.19
7	1	-7	-1.49	0.94
-7	1	7	0.13	0.74
6	-1	-7	578.57	10.98
-6	-1	7	573.82	13.47
6	1	-7	552.58	13.05
-6	1	7	550.03	10.67
6	1	-7	569.53	13.16
-5	-1	7	332.56	9.39
5	-1	-7	307.71	7.21
5	1	-7	319.01	9.05
-5	1	7	328.73	7.34
5	1	-7	307.75	8.90
-4	-1	7	560.14	12.76
-4	1	7	557.43	10.63
4	1	-7	543.09	12.44
4	1	-7	538.67	12.28
-3	-1	7	850.78	16.65
3	-1	-7	804.30	14.65
3	1	-7	823.43	16.04
3	1	-7	865.55	16.50
-3	1	7	827.27	14.49
-2	-1	7	79.38	4.19
2	-1	-7	84.19	3.64
2	1	-7	91.22	4.33
2	1	-7	90.28	4.21
-1	-1	7	136.44	5.36
1	-1	-7	127.98	4.73
1	1	-7	135.95	5.16
1	1	-7	119.26	5.00
-1	1	7	126.07	4.51
0	-1	-7	593.54	12.33
0	1	7	602.04	12.03
-1	-1	-7	2.58	1.17
1	-1	7	4.05	1.43
1	1	7	2.87	1.22
-1	1	-7	3.48	1.34
2	-1	7	50.89	3.41
-2	-1	-7	47.89	3.43
2	1	7	50.96	3.35
-2	1	-7	62.10	3.72
-3	-1	-7	-0.39	0.93
3	-1	7	0.59	1.04
-3	1	-7	-0.22	0.99
3	1	7	0.47	0.94
4	-1	7	416.14	10.23
-4	-1	-7	420.26	11.24

4	1	7	415.55	10.70
-4	1	-7	406.98	10.80
5	-1	7	57.17	3.66
-5	-1	-7	60.84	4.31
-5	1	-7	59.85	4.02
5	1	7	49.99	3.75
5	1	7	61.63	4.14
-6	-1	-7	14.47	2.76
6	-1	7	16.93	2.50
6	1	7	16.17	2.72
-6	1	-7	17.54	2.79
-7	-1	-7	56.47	4.59
7	-1	7	41.92	3.45
7	1	7	49.86	4.11
-7	1	-7	46.02	3.82
-8	-1	-7	23.40	3.70
8	-1	7	29.26	3.46
-8	1	-7	29.33	3.79
8	1	7	33.13	4.12
-9	-1	-7	183.36	8.75
9	-1	7	176.81	7.33
9	1	7	202.85	8.62
-9	1	-7	204.70	8.36
-10	-1	-7	20.74	3.97
10	-1	7	13.41	2.75
10	1	7	15.07	3.31
-10	1	-7	17.84	3.27
11	-1	7	323.49	10.60
-11	-1	-7	312.99	12.40
-11	1	-7	317.26	11.27
11	1	7	306.63	11.59
-12	-1	-7	62.92	6.83
12	-1	7	65.42	5.02
-12	1	-7	60.85	5.34
12	1	7	69.17	6.82
-13	-1	-7	4.52	2.75
13	-1	7	7.87	2.66
-13	1	-7	3.15	2.24
13	1	7	4.12	2.77
14	-1	7	45.17	5.15
-14	-1	-7	33.36	5.44
-14	1	-7	53.11	6.17
14	1	7	46.93	6.00
-15	-1	-7	-0.65	1.92
15	-1	7	1.49	2.17
-15	1	-7	-0.57	1.92
15	1	7	-1.07	1.91
-16	-1	-7	9.95	3.60
16	-1	7	6.66	2.86
16	1	7	4.79	3.26

-16	1	-7	5.81	3.07
-16	-2	7	18.12	4.58
16	2	-7	8.12	3.50
-15	-2	7	13.97	3.77
15	2	-7	10.30	3.46
-14	-2	7	11.90	3.69
14	2	-7	10.53	3.60
-13	-2	7	6.00	2.80
13	2	-7	1.52	2.33
-12	-2	7	-1.99	1.88
12	2	-7	2.91	2.44
-11	-2	7	-1.81	1.58
11	2	-7	1.00	1.88
-10	-2	7	2.37	1.85
10	2	-7	-2.03	1.58
-9	-2	7	0.95	1.62
9	2	-7	-2.48	1.29
-8	-2	7	0.46	1.51
8	2	-7	0.96	1.53
-8	2	7	1.31	0.65
-7	-2	7	53.93	4.39
-7	2	7	52.73	1.64
7	2	-7	50.66	4.29
-7	2	7	52.23	2.07
-6	-2	7	28.39	3.56
6	2	-7	36.13	4.12
-6	2	7	31.29	1.77
-6	2	7	33.88	1.61
-5	-2	7	94.39	5.39
-5	2	7	88.20	2.92
-5	2	7	94.67	3.11
5	2	-7	85.75	5.00
-4	-2	7	14.66	2.57
4	2	-7	15.79	2.63
-4	2	7	17.92	1.71
-4	2	7	18.81	1.83
-3	-2	7	59.83	4.17
3	-2	-7	54.49	2.73
-3	2	7	55.32	2.88
-3	2	7	57.90	2.84
3	2	-7	51.11	3.77
3	2	-7	53.86	3.87
2	-2	-7	61.06	3.09
-2	-2	7	59.28	4.10
-2	2	7	58.23	3.05
2	2	-7	68.80	4.28
2	2	-7	64.01	4.09
-1	-2	7	478.07	12.22
1	-2	-7	484.98	10.50
-1	2	7	493.56	10.39

1	2	-7	492.07	11.79
-1	2	7	494.21	10.62
1	2	-7	490.62	12.11
0	-2	7	133.48	5.96
0	-2	-7	145.36	5.37
0	2	7	137.97	5.28
0	2	7	127.67	4.99
0	2	-7	134.31	5.56
0	2	-7	136.67	5.85
1	-2	7	1345.32	23.99
-1	-2	-7	1320.85	23.18
-1	2	-7	1322.06	23.23
-1	2	-7	1335.08	23.74
1	2	7	1306.01	22.70
1	2	7	1334.87	23.19
2	-2	7	0.87	1.14
-2	-2	-7	-0.94	0.89
2	2	7	3.95	1.49
2	2	7	0.22	1.02
3	-2	7	112.51	5.25
-3	-2	-7	99.74	5.28
3	2	7	106.24	5.36
3	2	7	114.64	5.39
4	-2	7	262.15	8.34
-4	-2	-7	276.50	9.34
-4	2	-7	264.04	8.51
4	2	7	262.85	8.67
-5	-2	-7	25.58	3.48
5	-2	7	31.48	3.33
5	2	7	30.95	3.65
-5	2	-7	32.60	3.61
-6	-2	-7	58.19	4.77
6	-2	7	67.81	4.29
6	2	7	71.01	4.90
-6	2	-7	59.16	4.09
7	-2	7	25.35	3.20
-7	-2	-7	20.66	3.63
-7	2	-7	15.76	2.76
7	2	7	12.13	2.78
8	-2	7	372.51	10.89
-8	-2	-7	397.79	13.38
8	2	7	372.60	12.44
-8	2	-7	394.85	11.60
9	-2	7	131.50	6.02
-9	-2	-7	135.66	7.97
9	-2	7	131.75	6.43
-9	2	-7	129.87	6.58
9	2	7	122.19	7.35
-10	-2	-7	-2.48	1.57
10	-2	7	-1.59	1.23

-10	2	-7	1.19	1.69
10	2	7	1.48	2.07
-11	-2	-7	6.43	2.92
11	-2	7	5.55	2.19
11	2	7	5.82	2.82
-11	2	-7	6.64	2.37
-12	-2	-7	-1.00	1.83
12	-2	7	-0.45	1.65
12	2	7	-2.15	1.85
-12	2	-7	4.93	2.17
-13	-2	-7	4.65	2.83
13	-2	7	3.46	2.04
13	2	7	2.60	2.57
-13	2	-7	0.91	1.82
14	-2	7	11.55	2.89
-14	-2	-7	12.86	4.08
14	2	7	12.20	3.80
-14	2	-7	8.79	2.90
-15	-2	-7	33.59	6.05
15	-2	7	43.73	5.02
-15	2	-7	36.63	5.13
15	2	7	25.70	5.15
-15	-3	7	4.67	3.48
15	3	-7	7.17	3.37
-14	-3	7	13.48	4.07
14	3	-7	16.08	4.35
-13	-3	7	162.10	9.85
13	3	-7	133.07	8.85
-12	-3	7	-0.37	2.13
12	3	-7	2.51	2.55
-11	-3	7	141.55	8.73
11	3	-7	143.19	8.58
-10	-3	7	132.50	8.19
10	3	-7	142.79	8.30
-9	-3	7	56.09	6.13
8	3	-7	538.10	15.84
-7	-3	7	76.15	5.86
7	3	-7	84.23	6.01
-6	-3	7	288.95	10.94
6	3	-7	272.07	10.35
-5	-3	7	162.42	7.99
-5	3	7	164.46	3.76
-5	3	7	173.08	3.83
5	3	-7	175.20	7.97
-4	-3	7	10.66	2.67
4	-3	-7	16.85	1.62
4	3	-7	12.81	2.97
-4	3	7	19.63	1.90
-4	3	7	14.45	1.60
3	-3	-7	392.62	8.37

-3	-3	7	401.05	12.31
3	3	-7	386.08	11.58
-3	3	7	380.75	8.52
-3	3	7	387.33	8.46
-2	-3	7	53.97	4.36
2	-3	-7	44.43	2.83
-2	3	7	42.17	2.80
-2	3	7	41.60	2.81
2	3	-7	50.99	4.31
1	-3	-7	8.58	1.76
-1	-3	7	9.85	2.35
-1	3	7	8.12	1.75
1	3	-7	16.01	2.72
-1	3	7	13.80	2.09
0	-3	7	48.80	4.01
0	-3	-7	53.27	3.62
0	3	-7	49.36	3.82
0	3	7	61.79	3.85
0	3	7	53.59	3.51
-1	-3	-7	20.64	2.87
1	-3	7	24.03	3.29
1	3	7	18.07	2.63
1	3	7	20.11	2.71
-1	3	-7	23.26	3.07
-2	-3	-7	2.01	1.45
2	-3	7	-0.50	1.22
2	3	7	-0.44	1.23
-2	3	-7	1.05	1.28
3	-3	7	71.59	4.73
-3	-3	-7	71.45	4.86
3	3	7	63.42	4.42
-3	3	-7	64.35	4.19
-4	-3	-7	282.15	10.22
4	-3	7	286.37	9.34
4	3	7	312.53	10.25
-4	3	-7	277.22	8.67
5	-3	7	73.89	4.75
-5	-3	-7	73.08	5.44
-5	3	-7	75.49	4.55
5	3	7	76.00	5.44
-6	-3	-7	211.73	9.54
6	-3	7	205.06	7.87
6	3	7	210.88	9.07
-6	3	-7	219.55	7.58
7	-3	7	0.33	1.39
-7	-3	-7	3.41	2.48
7	3	7	-1.41	1.67
-8	-3	-7	49.35	6.29
8	-3	7	47.36	4.09
8	3	7	48.85	5.74

9	-3	7	46.39	4.49
-9	-3	-7	57.60	6.81
-9	3	-7	55.19	4.48
9	3	7	48.48	6.01
10	-3	7	108.97	5.95
-10	-3	-7	115.03	8.28
-10	3	-7	120.61	6.60
10	3	7	128.37	8.34
-11	-3	-7	2.14	2.51
11	-3	7	2.11	1.87
11	3	7	0.25	2.28
-11	3	-7	-0.43	1.46
-12	-3	-7	9.75	3.72
12	-3	7	8.46	2.49
-12	3	-7	15.30	3.14
12	3	7	2.77	2.80
-13	-3	-7	8.93	3.93
13	3	7	2.67	2.91
-14	-3	-7	11.28	4.27
14	3	7	21.41	5.20
-13	-4	7	0.35	2.86
13	4	-7	3.41	3.27
-12	-4	7	38.84	6.20
12	4	-7	41.40	6.34
-11	-4	7	15.18	4.20
11	4	-7	15.79	4.26
-10	-4	7	31.93	5.25
10	4	-7	46.59	6.28
-9	-4	7	40.15	5.62
9	4	-7	32.36	5.26
-8	-4	7	14.23	3.69
8	4	-7	8.24	3.22
-7	-4	7	48.59	5.96
7	4	-7	49.24	5.98
-6	-4	7	107.84	7.39
6	4	-7	118.02	7.61
-5	-4	7	10.38	3.00
5	4	-7	12.10	3.29
-4	-4	7	71.14	5.78
4	-4	-7	63.42	2.63
4	4	-7	48.35	5.33
-3	-4	7	-1.57	1.61
3	-4	-7	-0.21	0.93
3	4	-7	3.30	2.11
-2	-4	7	1.07	1.81
2	-4	-7	-0.77	1.05
-2	4	7	-1.00	1.11
-2	4	7	-1.09	1.12
2	4	-7	-0.82	1.53
1	-4	-7	300.86	8.84

-1	-4	7	293.62	10.97
1	4	-7	305.49	10.54
-1	4	7	290.65	8.62
-1	4	7	309.65	9.04
0	-4	-7	0.27	1.34
0	-4	7	1.98	1.75
0	4	-7	-0.90	1.48
0	4	7	0.81	1.35
1	-4	7	167.71	7.97
-1	-4	-7	188.07	7.64
1	4	7	187.90	7.39
-1	4	-7	169.29	7.51
2	-4	7	330.89	11.14
-2	-4	-7	326.03	10.90
2	4	7	338.00	10.68
-2	4	-7	324.01	10.50
3	-4	7	7.11	2.45
-3	-4	-7	8.39	2.71
3	4	7	9.85	2.64
-3	4	-7	9.35	2.45
4	-4	7	350.92	11.25
-4	-4	-7	353.65	12.37
-4	4	-7	348.07	10.51
4	4	7	341.77	11.83
5	-4	7	284.07	9.96
-5	-4	-7	283.02	11.54
5	4	7	297.08	11.30
-5	4	-7	298.58	9.53
-6	-4	-7	2.38	2.22
6	-4	7	7.66	2.31
-6	4	-7	5.89	2.20
6	4	7	5.00	2.50
7	-4	7	99.64	5.72
-7	-4	-7	86.49	6.94
7	4	7	92.46	6.84
-7	4	-7	93.28	5.15
8	-4	7	27.17	3.66
-8	-4	-7	33.83	5.48
8	4	7	24.51	4.60
-9	-4	-7	0.57	2.07
9	-4	7	-1.65	1.29
9	4	7	-0.60	2.16
-10	-4	-7	5.55	2.98
10	4	7	6.36	3.31
-11	-4	-7	11.84	4.15
11	4	7	15.90	4.38
-12	-4	-7	3.68	3.07
12	4	7	4.73	3.29
-10	-5	7	1.99	2.75
10	5	-7	1.28	3.13

-9	-5	7	8.59	3.54
9	5	-7	1.63	2.70
-8	-5	7	30.94	5.54
8	5	-7	31.75	5.55
-7	-5	7	0.31	2.35
7	5	-7	-2.99	2.07
-6	-5	7	2.79	2.58
6	5	-7	1.42	2.63
-5	-5	7	1.51	2.15
5	5	-7	-1.28	2.07
-4	-5	7	3.00	2.45
4	5	-7	-0.28	1.77
-3	-5	7	46.52	5.79
3	5	-7	22.87	4.32
-2	-5	7	-0.99	1.78
2	-5	-7	-0.69	1.23
2	5	-7	-4.27	1.67
1	-5	-7	8.81	2.35
-1	-5	7	7.76	2.86
1	5	-7	7.80	2.80
0	-5	7	-0.34	1.80
0	-5	-7	-0.42	1.33
0	5	7	-1.12	1.49
0	5	-7	-0.62	1.73
-1	-5	-7	1.02	1.74
1	-5	7	3.73	2.27
-1	5	-7	1.76	2.01
1	5	7	0.44	1.68
-2	-5	-7	8.08	2.77
2	-5	7	2.59	2.12
2	5	7	7.01	2.59
-2	5	-7	7.55	2.46
-3	-5	-7	10.60	3.28
3	-5	7	7.59	2.64
3	5	7	6.07	2.70
-3	5	-7	2.63	2.03
-4	-5	-7	21.86	4.40
4	-5	7	17.29	3.34
-4	5	-7	21.23	3.51
4	5	7	23.08	4.11
-5	-5	-7	23.41	4.46
5	-5	7	21.20	3.66
5	5	7	23.81	4.36
-6	-5	-7	-0.38	1.91
6	-5	7	0.64	1.67
6	5	7	-0.43	2.18
7	-5	7	2.12	1.87
-7	-5	-7	2.00	2.43
7	5	7	6.43	3.07
-8	-5	-7	0.38	2.17

8	5	7	1.74	2.75
-9	-5	-7	-0.98	2.32
9	5	7	0.50	2.62
-4	-6	7	2.89	2.85
4	6	-7	6.58	3.27
-3	-6	7	25.34	4.97
3	6	-7	32.81	5.35
-2	-6	7	4.01	2.87
2	6	-7	8.35	3.14
-1	-6	7	27.53	5.02
1	6	-7	15.38	3.85
0	-6	7	8.34	3.10
0	6	-7	4.85	2.75
1	-6	7	23.11	4.47
-1	6	-7	25.02	4.20
2	-6	7	33.64	5.08
-2	-6	-7	46.26	5.80
2	6	7	33.03	4.80
-2	6	-7	29.26	4.44
-3	-6	-7	1.80	2.41
3	-6	7	3.69	2.65
3	6	7	2.91	2.64
-4	-6	-7	21.71	4.68
4	6	7	27.99	4.96
16	0	-8	19.98	3.72
15	0	-8	-0.83	1.87
14	0	-8	91.73	5.91
-14	0	8	89.82	5.68
-13	0	8	1.27	1.83
13	0	-8	1.18	1.86
12	0	-8	587.80	14.66
-12	0	8	593.14	14.64
-11	0	8	936.14	19.11
11	0	-8	885.65	18.94
10	0	-8	24.97	3.19
-10	0	8	23.07	3.07
10	0	-8	28.69	3.51
9	0	-8	647.77	14.45
-9	0	8	673.52	14.72
9	0	-8	660.66	14.62
8	0	-8	1272.39	22.85
-8	0	8	1277.93	22.97
8	0	-8	1306.04	22.88
7	0	-8	170.55	6.21
7	0	-8	173.75	6.33
-7	0	8	170.09	6.29
6	0	-8	104.12	4.72
-6	0	8	113.72	4.96
6	0	-8	113.63	4.95
5	0	-8	40.36	2.92

5	0	-8	34.47	2.76
-5	0	8	40.66	3.07
4	0	-8	24.41	2.62
4	0	-8	23.67	2.62
-4	0	8	22.71	2.56
-3	0	8	459.53	10.61
-2	0	8	18.98	2.33
1	0	-8	211.90	6.87
0	0	-8	434.10	10.62
0	0	8	414.52	10.17
-1	0	-8	289.55	8.54
1	0	8	284.14	8.04
-2	0	-8	180.85	6.64
2	0	8	190.30	6.47
-3	0	-8	590.50	13.73
3	0	8	570.88	12.92
-4	0	-8	123.05	5.84
4	0	8	120.99	5.44
-5	0	-8	72.78	4.65
5	0	8	67.26	4.23
-6	0	-8	874.73	19.21
6	0	8	883.41	18.47
-7	0	-8	5.67	2.06
7	0	8	4.85	1.84
-8	0	-8	699.96	17.68
8	0	8	679.97	16.37
9	0	8	317.61	10.77
-9	0	-8	326.23	11.71
-10	0	-8	59.34	5.30
10	0	8	54.12	5.55
11	0	8	281.79	10.74
-11	0	-8	295.76	11.79
-12	0	-8	69.76	6.17
12	0	8	57.42	6.01
13	0	8	5.50	2.70
-13	0	-8	9.12	3.14
-14	0	-8	64.60	7.22
14	0	8	60.41	6.63
15	0	8	5.55	2.93
-15	0	-8	7.70	3.31
-16	0	-8	5.89	3.47
16	0	8	5.07	2.85
-16	-1	8	-0.68	2.15
16	1	-8	-2.53	1.80
-15	-1	8	7.95	2.86
15	1	-8	13.26	3.52
-14	-1	8	-1.66	1.83
14	1	-8	3.07	2.38
-13	-1	8	34.28	4.53
13	-1	-8	36.87	3.83

13	1	-8	32.26	4.61
12	-1	-8	37.62	3.68
-12	-1	8	29.93	4.12
12	1	-8	35.93	4.65
-12	1	8	33.34	3.14
-11	-1	8	143.80	7.38
11	-1	-8	147.75	5.79
11	-1	-8	134.57	5.33
-11	1	8	135.47	5.33
11	1	-8	146.62	7.45
10	-1	-8	35.61	3.13
10	-1	-8	33.77	3.32
-10	-1	8	31.91	4.07
-10	1	8	37.45	2.86
10	1	-8	40.14	4.53
9	-1	-8	1.28	1.32
-9	-1	8	1.03	1.44
9	-1	-8	0.77	1.06
-9	1	8	0.71	0.98
9	1	-8	-0.11	1.40
-8	-1	8	55.33	4.34
8	-1	-8	54.09	3.21
8	-1	-8	59.61	3.35
-8	1	8	54.73	3.13
8	1	-8	57.21	4.24
-7	-1	8	58.58	4.19
7	-1	-8	55.34	3.22
-7	1	8	55.91	3.02
7	1	-8	51.37	3.95
6	-1	-8	39.97	2.77
-6	-1	8	44.66	3.65
6	1	-8	48.56	4.44
6	1	-8	52.06	3.85
-6	1	8	44.29	2.71
-5	-1	8	37.17	3.70
5	-1	-8	43.53	2.81
-5	1	8	35.80	2.52
5	1	-8	38.99	3.78
5	1	-8	38.33	3.76
-4	-1	8	404.61	10.79
4	1	-8	393.31	10.34
4	1	-8	397.84	10.58
-4	1	8	400.73	8.99
3	-1	-8	101.13	4.26
-3	-1	8	103.76	5.08
3	1	-8	90.29	4.69
3	1	-8	105.56	4.90
-3	1	8	94.47	4.00
2	-1	-8	3.92	1.28
-2	-1	8	1.24	1.31

2	1	-8	3.65	1.39
-2	1	8	3.18	1.24
2	1	-8	5.14	1.55
1	-1	-8	1447.39	24.18
-1	-1	8	1411.30	24.98
-1	1	8	1432.26	23.64
1	1	-8	1487.96	25.18
0	-1	8	315.59	9.08
0	-1	-8	326.18	8.79
0	1	8	334.86	8.57
-1	-1	-8	52.18	3.66
1	-1	8	43.82	3.34
-1	1	-8	59.99	3.93
1	1	8	59.96	3.56
-2	-1	-8	1184.95	21.53
2	-1	8	1117.98	21.01
2	1	8	1130.61	20.71
-2	1	-8	1137.90	21.44
3	-1	8	6.19	1.82
-3	-1	-8	5.76	1.74
-3	1	-8	5.37	1.70
3	1	8	2.96	1.42
4	-1	8	53.90	3.70
-4	-1	-8	61.13	4.36
-4	1	-8	60.26	4.19
4	1	8	64.85	4.16
5	-1	8	1.51	1.33
-5	-1	-8	1.78	1.33
5	1	8	2.03	1.28
-5	1	-8	1.34	1.21
6	-1	8	19.24	2.85
-6	-1	-8	19.44	3.17
6	1	8	22.62	3.26
-6	1	-8	20.29	3.12
-7	-1	-8	10.71	2.63
7	-1	8	14.76	2.67
7	1	8	12.47	2.77
-7	1	-8	8.33	2.36
-8	-1	-8	27.10	4.24
8	-1	8	36.99	4.11
-8	1	-8	28.98	3.94
8	1	8	30.87	4.20
-9	-1	-8	9.92	2.93
9	-1	8	14.10	2.89
9	1	8	17.04	3.42
-9	1	-8	17.09	3.35
10	-1	8	2.81	1.83
-10	-1	-8	1.32	1.95
10	1	8	0.71	2.12
-10	1	-8	1.30	1.77

11	-1	8	7.82	2.61
-11	-1	-8	3.90	2.44
-11	1	-8	8.02	2.83
11	1	8	10.48	3.06
12	-1	8	3.11	2.16
-12	-1	-8	4.17	2.79
12	1	8	7.64	3.04
-12	1	-8	6.89	2.70
13	-1	8	-0.08	1.69
-13	-1	-8	0.27	2.18
13	1	8	-0.06	2.14
-13	1	-8	0.79	1.97
14	-1	8	2.31	2.27
-14	-1	-8	0.06	2.29
14	1	8	-0.05	2.04
-14	1	-8	2.59	2.24
-15	-1	-8	1.03	2.54
15	-1	8	2.96	2.37
15	1	8	0.40	2.32
-15	1	-8	3.66	2.60
16	-1	8	1.80	2.47
-16	-1	-8	3.05	3.04
16	1	8	-1.36	2.36
-16	1	-8	2.07	2.54
-15	-2	8	3.09	2.80
15	2	-8	0.07	2.59
-14	-2	8	29.87	4.84
14	2	-8	41.75	5.66
-13	-2	8	17.60	3.83
13	2	-8	21.53	4.35
-12	-2	8	1.62	2.02
12	2	-8	8.41	2.94
-11	-2	8	75.80	5.97
11	2	-8	87.30	6.43
-10	-2	8	54.58	5.57
10	2	-8	53.41	5.75
-9	-2	8	100.29	6.42
9	2	-8	108.15	6.50
-8	-2	8	169.42	8.05
8	-2	-8	173.76	4.62
8	2	-8	175.83	7.98
-8	2	8	160.60	3.72
-7	-2	8	12.89	2.78
7	-2	-8	19.11	1.92
7	2	-8	13.80	2.84
-7	2	8	14.11	1.59
-7	2	8	18.46	1.42
6	-2	-8	323.51	7.08
-6	-2	8	326.91	10.91
6	2	-8	341.78	10.80

-6	2	8	329.63	6.77
-6	2	8	326.52	7.06
-5	-2	8	10.27	2.48
-5	2	8	10.87	1.43
-5	2	8	16.99	1.84
5	2	-8	12.74	2.60
4	-2	-8	37.08	2.39
-4	-2	8	32.81	3.85
4	2	-8	34.15	3.82
-4	2	8	33.00	2.22
-3	-2	8	4.80	1.73
3	-2	-8	3.46	1.09
-3	2	8	4.08	1.24
3	2	-8	3.55	1.60
2	-2	-8	13.68	1.99
-2	-2	8	12.71	2.43
2	2	-8	7.49	2.00
-2	2	8	8.40	1.61
-1	-2	8	39.89	3.97
1	-2	-8	34.72	2.88
1	2	-8	44.33	4.11
-1	2	8	40.04	2.94
0	-2	-8	702.04	14.50
0	-2	8	665.28	15.46
0	2	-8	691.65	14.99
0	2	8	683.78	14.07
0	2	-8	693.37	15.51
-1	-2	-8	1.69	1.26
1	-2	8	2.01	1.41
1	2	8	-0.82	0.96
-1	2	-8	2.57	1.42
1	2	8	-0.69	1.00
-1	2	-8	2.45	1.55
2	-2	8	15.49	2.65
-2	-2	-8	14.99	2.54
2	2	8	16.05	2.52
2	2	8	13.16	2.31
-3	-2	-8	0.36	1.11
3	-2	8	-0.76	1.05
3	2	8	1.37	1.32
4	-2	8	1.21	1.30
-4	-2	-8	-1.22	1.00
-4	2	-8	-0.79	0.97
4	2	8	0.37	1.14
5	-2	8	100.75	5.26
-5	-2	-8	86.93	5.59
-5	2	-8	87.52	5.15
5	2	8	89.52	5.51
6	-2	8	585.82	14.36
-6	-2	-8	596.72	16.23

6	2	8	603.04	15.60
-6	2	-8	620.33	15.04
7	-2	8	42.21	4.21
-7	-2	-8	40.66	5.15
7	2	8	35.44	4.50
-7	2	-8	38.84	4.31
8	-2	8	204.85	8.06
-8	-2	-8	208.99	9.78
-8	2	-8	211.80	8.69
8	2	8	213.62	9.44
9	-2	8	311.48	10.48
9	2	8	340.34	12.33
-9	2	-8	335.32	11.29
10	-2	8	7.08	2.32
-10	-2	-8	3.86	2.40
-10	2	-8	7.05	2.46
10	2	8	8.96	2.93
11	-2	8	59.60	4.71
-11	-2	-8	68.36	7.43
-11	2	-8	74.83	5.69
11	2	8	58.31	6.49
-12	-2	-8	8.14	3.15
12	-2	8	15.84	3.26
-12	2	-8	8.49	2.72
12	2	8	14.15	3.90
-13	-2	-8	20.41	4.64
13	-2	8	34.72	4.45
-13	2	-8	31.81	4.60
13	2	8	25.42	4.91
14	-2	8	55.81	5.65
-14	-2	-8	48.98	7.12
-14	2	-8	53.82	6.10
14	2	8	48.65	6.71
15	-2	8	15.08	3.48
-15	-2	-8	10.61	4.00
15	2	8	8.26	3.64
-15	2	-8	12.59	3.51
-14	-3	8	23.00	4.97
14	3	-8	11.18	3.85
-13	-3	8	25.61	4.93
13	3	-8	28.28	5.14
-12	-3	8	17.01	4.03
12	3	-8	7.65	3.32
-11	-3	8	7.14	2.91
11	3	-8	4.34	2.88
-10	-3	8	1.37	2.01
10	3	-8	-1.61	1.80
-9	-3	8	23.78	4.23
9	3	-8	36.81	5.28
-8	-3	8	9.85	2.97

8	3	-8	11.75	3.22
-7	-3	8	102.63	6.85
-6	-3	8	107.65	6.83
6	3	-8	120.32	7.04
-5	-3	8	82.77	5.96
-5	3	8	92.00	3.08
-5	3	8	86.12	3.03
5	3	-8	96.10	6.16
-4	-3	8	309.47	11.16
4	-3	-8	291.62	6.75
-4	3	8	292.05	6.96
4	3	-8	296.44	10.51
-3	-3	8	880.05	20.29
3	-3	-8	856.71	15.39
-3	3	8	870.13	15.52
-3	3	8	875.08	15.72
3	3	-8	852.35	19.45
2	-3	-8	40.23	2.92
-2	-3	8	38.10	4.42
2	3	-8	43.84	4.69
-2	3	8	40.52	2.98
-2	3	8	39.58	2.93
-1	-3	8	59.53	4.76
1	-3	-8	52.97	3.53
-1	3	8	65.97	3.79
-1	3	8	55.04	3.65
1	3	-8	59.92	4.51
0	-3	8	-0.12	1.35
0	-3	-8	0.35	1.07
0	3	8	0.62	1.08
0	3	8	0.46	1.17
0	3	-8	-1.29	1.21
1	-3	8	41.58	4.35
-1	-3	-8	40.50	3.62
1	3	8	39.12	3.87
1	3	8	41.24	3.95
-1	3	-8	39.35	4.09
2	-3	8	34.32	3.95
-2	-3	-8	39.10	4.26
2	3	8	30.29	3.60
-2	3	-8	35.51	3.85
3	-3	8	45.27	4.56
-3	-3	-8	43.15	4.66
3	3	8	48.43	4.06
-3	3	-8	45.06	3.68
4	-3	8	162.04	7.20
-4	-3	-8	148.85	7.49
-4	3	-8	153.64	6.61
4	3	8	154.61	7.21
5	-3	8	3.56	1.84

-5	-3	-8	3.38	2.00
-5	3	-8	2.38	1.67
5	3	8	2.11	1.73
6	-3	8	-0.04	1.36
-6	-3	-8	3.00	2.16
6	3	8	1.34	1.78
7	-3	8	35.63	4.06
-7	-3	-8	43.85	5.58
7	3	8	39.53	5.05
8	-3	8	23.47	3.38
-8	-3	-8	20.60	4.14
8	3	8	18.93	3.91
-9	-3	-8	14.99	3.84
9	-3	8	2.94	1.83
9	3	8	7.43	2.92
-9	3	-8	6.16	2.27
10	-3	8	6.79	2.27
-10	-3	-8	16.06	4.17
10	3	8	5.89	2.97
-10	3	-8	6.98	2.46
11	-3	8	-0.95	1.41
-11	-3	-8	1.94	2.63
11	3	8	1.38	2.42
-11	3	-8	1.69	1.85
-12	-3	-8	0.92	2.42
12	-3	8	0.58	1.74
-12	3	-8	1.00	1.91
12	3	8	-2.37	1.97
-13	-3	-8	3.93	3.17
13	3	8	-0.18	2.57
-14	-3	-8	46.83	7.44
14	3	8	55.40	7.50
-12	-4	8	2.35	2.86
12	4	-8	0.59	2.76
-11	-4	8	8.48	3.69
11	4	-8	5.35	3.15
-10	-4	8	8.52	3.45
10	4	-8	12.79	4.03
-9	-4	8	22.62	4.42
9	4	-8	27.95	5.01
-8	-4	8	14.35	3.77
8	4	-8	16.49	3.94
-7	-4	8	8.81	3.17
7	4	-8	13.48	3.66
-6	-4	8	30.44	4.83
6	4	-8	37.64	5.21
-5	-4	8	4.47	2.57
5	4	-8	-1.51	2.06
4	-4	-8	35.49	2.30
-4	-4	8	35.86	5.01

4	4	-8	35.30	4.98
3	-4	-8	28.18	2.77
-3	-4	8	27.08	4.28
3	4	-8	29.52	4.45
-2	-4	8	9.25	2.86
2	-4	-8	15.40	2.40
-2	4	8	11.26	2.16
2	4	-8	8.78	2.71
1	-4	-8	10.94	2.28
-1	-4	8	10.17	2.89
-1	4	8	10.33	2.23
-1	4	8	7.60	2.00
1	4	-8	9.97	2.84
0	-4	-8	-1.35	1.18
0	-4	8	-2.96	1.02
0	4	-8	2.74	1.96
0	4	8	-0.63	1.36
1	-4	8	-1.43	1.36
-1	-4	-8	1.03	1.56
1	4	8	1.43	1.67
-1	4	-8	2.35	1.81
2	-4	8	3.03	2.06
-2	-4	-8	5.55	2.18
2	4	8	3.15	2.06
-2	4	-8	3.87	2.03
3	-4	8	66.04	5.08
-3	-4	-8	61.44	5.05
-3	4	-8	57.16	4.61
3	4	8	63.52	5.07
-4	-4	-8	3.06	2.17
4	-4	8	-0.55	1.59
4	4	8	-0.54	1.76
-4	4	-8	1.15	1.63
-5	-4	-8	2.91	2.18
5	-4	8	3.02	1.98
5	4	8	-0.84	1.79
-5	4	-8	0.05	1.59
6	-4	8	20.70	3.49
-6	-4	-8	18.56	3.92
6	4	8	18.72	3.83
-6	4	-8	22.96	3.40
7	-4	8	0.80	1.68
-7	-4	-8	1.96	2.28
7	4	8	2.59	2.36
-7	4	-8	0.31	1.50
-8	-4	-8	76.11	6.82
8	-4	8	80.81	5.57
8	4	8	77.57	7.88
-9	-4	-8	22.33	4.79
9	-4	8	24.82	3.69

9	4	8	31.21	5.37
10	-4	8	13.83	2.97
-10	-4	-8	16.57	4.35
10	4	8	14.72	4.21
-11	-4	-8	115.87	9.19
11	4	8	125.36	9.15
-12	-4	-8	14.19	4.42
12	4	8	12.11	4.29
-9	-5	8	40.17	6.44
9	5	-8	33.34	5.91
-8	-5	8	1.36	2.67
8	5	-8	-3.23	2.37
-7	-5	8	75.91	8.14
7	5	-8	84.51	8.69
-6	-5	8	5.00	2.87
6	5	-8	6.17	3.22
-5	-5	8	3.12	2.49
5	5	-8	2.91	2.44
-4	-5	8	36.21	5.45
4	5	-8	31.97	4.92
-3	-5	8	13.24	3.67
3	5	-8	8.37	3.04
-2	-5	8	16.55	3.91
2	5	-8	25.84	4.40
-1	-5	8	31.23	4.88
1	-5	-8	40.82	4.20
1	5	-8	32.36	4.62
0	-5	8	-0.48	1.91
0	-5	-8	0.57	1.51
0	5	-8	-1.86	1.49
0	5	8	0.40	1.64
1	-5	8	66.50	6.60
-1	-5	-8	65.12	5.07
-1	5	-8	56.59	5.85
1	5	8	55.45	4.68
2	-5	8	53.85	5.83
-2	-5	-8	43.98	5.17
2	5	8	47.63	5.25
-2	5	-8	45.41	5.15
-3	-5	-8	25.20	4.22
3	-5	8	26.65	4.22
3	5	8	31.09	4.48
-3	5	-8	35.57	4.47
4	-5	8	66.92	5.62
-4	-5	-8	79.91	6.37
4	5	8	62.02	6.49
-4	5	-8	65.63	5.17
-5	-5	-8	2.35	2.34
5	-5	8	1.62	2.06
5	5	8	0.11	2.15

-5	5	-8	-0.89	1.59
-6	-5	-8	13.80	3.81
6	-5	8	17.16	3.43
6	5	8	16.67	3.97
7	-5	8	24.36	3.90
-7	-5	-8	26.45	4.99
7	5	8	27.58	5.07
-8	-5	-8	-0.03	2.21
8	5	8	-1.56	2.36
-9	-5	-8	11.37	3.82
9	5	8	4.38	3.50
-3	-6	8	11.14	3.80
3	6	-8	6.52	3.23
-2	-6	8	1.21	2.56
2	6	-8	7.96	3.35
-1	-6	8	0.73	2.46
1	6	-8	1.58	2.24
0	-6	8	2.01	2.64
0	6	-8	1.72	2.43
1	-6	8	1.33	2.47
-1	6	-8	3.78	2.49
-2	-6	-8	8.90	3.09
2	-6	8	10.92	3.46
2	6	8	4.77	2.75
-2	6	-8	6.35	2.82
3	-6	8	0.20	2.40
-3	-6	-8	-2.58	1.79
3	6	8	-1.39	2.30
16	0	-9	1.91	2.22
15	0	-9	-2.60	1.69
-14	0	9	-0.43	1.68
14	0	-9	-0.03	1.96
13	0	-9	-0.12	1.74
-13	0	9	-0.32	1.56
12	0	-9	4.33	2.17
-12	0	9	2.18	1.89
-11	0	9	0.15	1.71
11	0	-9	-3.68	1.59
10	0	-9	2.52	1.80
-10	0	9	3.07	1.68
9	0	-9	1.64	1.54
9	0	-9	3.81	1.60
-9	0	9	2.22	1.54
8	0	-9	1.54	1.45
-8	0	9	-2.59	1.13
8	0	-9	1.05	1.37
7	0	-9	-0.18	1.09
7	0	-9	-0.81	1.13
-7	0	9	0.52	1.07
6	0	-9	0.42	1.12

-6	0	9	0.41	1.12
6	0	-9	1.33	1.13
-5	0	9	-0.49	1.06
5	0	-9	0.03	1.08
5	0	-9	0.69	1.10
-4	0	9	-0.08	1.09
4	0	-9	-1.76	0.87
4	0	-9	-0.66	0.88
-3	0	9	-0.28	0.89
-2	0	9	-1.38	0.94
-1	0	9	-1.60	0.68
1	0	-9	0.99	1.12
0	0	-9	0.56	1.10
0	0	9	1.86	1.19
-1	0	-9	0.68	1.12
1	0	9	0.11	1.03
-2	0	-9	0.16	1.08
2	0	9	0.69	1.06
-3	0	-9	-0.07	0.93
3	0	9	0.50	1.06
4	0	9	-0.85	1.15
-4	0	-9	0.68	1.22
-5	0	-9	-0.38	1.02
5	0	9	-0.77	1.12
-6	0	-9	1.38	1.48
6	0	9	0.22	1.24
-7	0	-9	-1.83	1.18
7	0	9	0.12	1.45
-8	0	-9	0.48	1.60
8	0	9	0.29	1.52
9	0	9	-0.12	1.72
-9	0	-9	-1.69	1.22
10	0	9	3.51	2.35
-10	0	-9	1.59	1.83
11	0	9	-1.57	1.54
-11	0	-9	0.41	1.90
-12	0	-9	1.98	2.08
12	0	9	2.34	2.13
-13	0	-9	-0.93	1.87
13	0	9	-0.47	1.69
14	0	9	1.87	2.46
-14	0	-9	-0.71	1.94
15	0	9	0.22	2.16
-15	0	-9	-1.40	2.07
-16	0	-9	0.50	2.35
16	0	9	-2.04	2.22
16	1	-9	11.77	3.71
-15	-1	9	0.55	2.25
15	1	-9	3.70	2.55
-14	-1	9	67.74	5.81

14	1	-9	78.14	6.09
-13	-1	9	162.54	8.33
13	1	-9	143.34	7.78
12	-1	-9	2.09	1.76
-12	-1	9	-2.28	1.67
12	1	-9	0.66	2.03
11	-1	-9	42.50	3.58
-11	-1	9	39.22	4.63
-11	1	9	46.57	3.37
11	1	-9	39.88	4.81
10	-1	-9	287.92	8.33
10	-1	-9	291.89	8.14
-10	-1	9	291.17	10.44
10	1	-9	290.25	10.28
-10	1	9	298.78	8.11
-9	-1	9	83.66	5.40
9	-1	-9	96.26	4.54
-9	1	9	93.40	4.29
9	1	-9	94.11	5.69
-8	-1	9	235.57	8.81
8	-1	-9	221.42	6.85
8	1	-9	222.40	8.41
-8	1	9	222.87	6.67
7	-1	-9	55.83	3.39
-7	-1	9	51.77	4.14
-7	1	9	50.03	3.11
7	1	-9	45.91	3.85
-6	-1	9	134.98	6.40
6	-1	-9	136.55	5.13
6	1	-9	143.53	6.37
-6	1	9	125.49	4.88
-5	-1	9	173.29	7.07
5	-1	-9	163.16	5.61
5	1	-9	166.03	6.77
-5	1	9	175.47	5.74
-4	-1	9	416.08	11.34
4	1	-9	395.55	10.82
4	1	-9	397.98	10.87
-4	1	9	403.23	9.52
3	-1	-9	31.29	2.75
-3	-1	9	32.19	3.47
3	1	-9	32.27	3.45
-3	1	9	32.79	2.70
3	1	-9	27.83	3.21
2	-1	-9	172.07	6.12
-2	-1	9	171.85	6.78
2	1	-9	170.09	6.62
2	1	-9	171.13	6.57
-2	1	9	179.04	6.06
1	-1	-9	116.22	5.15

-1	-1	9	111.99	5.47
-1	1	9	111.69	4.85
0	-1	-9	527.41	12.45
0	-1	9	533.79	12.91
0	1	9	528.57	12.03
-1	-1	-9	269.72	8.55
1	-1	9	265.45	8.52
-1	1	-9	260.10	8.72
1	1	9	280.48	8.31
-2	-1	-9	51.03	3.84
2	-1	9	49.98	3.77
2	1	9	45.84	3.51
-2	1	-9	43.67	3.73
-3	-1	-9	763.41	16.90
3	-1	9	758.89	16.46
-3	1	-9	738.41	16.78
3	1	9	736.09	16.16
-4	-1	-9	163.52	7.25
4	-1	9	153.17	6.50
-4	1	-9	152.87	6.97
-5	-1	-9	174.85	7.81
5	-1	9	172.05	6.94
5	1	9	175.01	7.38
-5	1	-9	166.57	7.37
6	-1	9	18.15	2.94
-6	-1	-9	15.76	3.01
6	1	9	14.80	2.94
-6	1	-9	20.43	3.29
7	-1	9	299.09	9.88
-7	-1	-9	303.02	11.15
7	1	9	300.61	10.57
-7	1	-9	327.85	10.97
-8	-1	-9	454.95	14.53
8	-1	9	481.63	13.17
8	1	9	464.16	13.93
-8	1	-9	475.53	14.03
-9	-1	-9	96.21	6.86
9	-1	9	96.27	5.97
-9	1	-9	104.22	6.56
9	1	9	97.29	6.59
10	-1	9	159.98	7.70
-10	-1	-9	173.19	9.21
-10	1	-9	170.35	8.53
10	1	9	165.53	8.60
-11	-1	-9	70.78	7.45
11	-1	9	69.50	5.43
-11	1	-9	67.94	5.77
11	1	9	75.50	6.17
-12	-1	-9	5.26	2.85
12	-1	9	11.32	3.14

12	1	9	13.03	3.63
-12	1	-9	8.90	3.00
13	-1	9	118.00	7.25
-13	-1	-9	117.84	8.45
13	1	9	126.78	8.39
-13	1	-9	115.10	7.68
14	-1	9	2.98	2.39
-14	-1	-9	0.47	2.24
-14	1	-9	3.04	2.33
14	1	9	-1.37	2.21
-15	-1	-9	-1.08	2.32
15	-1	9	1.60	2.25
15	1	9	-1.94	2.32
-15	1	-9	2.42	2.49
-15	-2	9	44.54	6.29
15	2	-9	38.62	5.83
-14	-2	9	-1.30	2.09
14	2	-9	2.64	2.50
-13	-2	9	-0.97	2.04
13	2	-9	1.59	2.29
-12	-2	9	2.41	2.51
12	2	-9	4.83	2.70
-11	-2	9	-1.39	1.81
11	2	-9	-1.53	1.81
-10	-2	9	-2.31	1.61
10	2	-9	2.98	2.30
-9	-2	9	55.57	5.66
-9	2	9	52.65	2.25
9	2	-9	56.60	5.79
8	-2	-9	1.19	1.04
-8	-2	9	2.51	1.89
-8	2	9	1.50	0.83
8	2	-9	1.84	1.86
7	-2	-9	75.62	3.41
-7	-2	9	82.94	5.71
7	2	-9	73.10	5.33
-7	2	9	78.20	3.09
6	-2	-9	0.14	1.04
-6	-2	9	-0.60	1.36
-6	2	9	0.61	0.82
6	2	-9	1.25	1.60
-5	-2	9	2.58	1.75
5	2	-9	9.48	2.46
-5	2	9	5.31	1.27
-4	-2	9	144.21	7.00
4	-2	-9	152.96	5.20
4	2	-9	154.55	6.97
-4	2	9	149.44	5.00
3	-2	-9	65.49	3.57
-3	-2	9	61.32	4.58

3	2	-9	68.25	4.62
-2	-2	9	18.91	2.96
2	-2	-9	26.05	2.80
-2	2	9	22.87	2.63
2	2	-9	22.00	3.12
-1	-2	9	20.09	3.11
1	-2	-9	20.55	2.65
-1	2	9	21.26	2.67
1	2	-9	20.27	3.05
0	-2	9	667.66	15.80
0	-2	-9	644.84	14.45
0	2	-9	694.13	15.41
0	2	9	665.78	14.25
1	-2	9	55.59	4.22
-1	-2	-9	60.91	4.09
-1	2	-9	55.20	4.05
1	2	9	55.89	3.80
2	-2	9	71.26	4.65
-2	-2	-9	72.70	4.65
2	2	9	57.94	4.08
-3	-2	-9	65.04	4.67
3	-2	9	62.67	4.41
3	2	9	61.72	4.34
-4	-2	-9	28.55	3.82
4	-2	9	27.78	3.63
4	2	9	28.91	3.87
-4	2	-9	22.25	3.36
5	-2	9	23.52	3.33
-5	-2	-9	20.97	3.50
-5	2	-9	25.15	3.58
5	2	9	24.84	3.65
6	-2	9	117.53	6.14
-6	-2	-9	111.42	6.82
-6	2	-9	115.43	6.41
6	2	9	113.96	6.56
7	-2	9	8.57	2.37
-7	-2	-9	13.71	3.38
-7	2	-9	12.62	2.81
7	2	9	10.53	2.93
8	-2	9	229.58	8.90
-8	2	-9	227.02	9.36
8	2	9	216.97	9.75
9	-2	9	6.69	2.37
-9	-2	-9	5.24	2.63
-9	2	-9	6.81	2.63
9	2	9	5.20	2.61
10	-2	9	5.75	2.26
-10	-2	-9	3.61	2.61
-10	2	-9	1.49	1.92
10	2	9	2.13	2.53

11	-2	9	39.93	4.62
-11	-2	-9	38.46	6.00
11	2	9	44.81	6.01
-11	2	-9	37.53	4.95
-12	-2	-9	31.34	5.61
12	-2	9	31.17	4.32
12	2	9	26.76	4.99
-12	2	-9	22.46	4.01
-13	-2	-9	5.81	3.23
13	-2	9	5.34	2.60
13	2	9	5.78	3.35
-13	2	-9	16.92	3.83
14	-2	9	0.19	2.12
-14	-2	-9	0.83	2.63
14	2	9	-1.79	2.41
-14	2	-9	0.16	2.08
15	-2	9	39.29	5.16
-15	-2	-9	35.62	6.39
15	2	9	42.61	6.73
-15	2	-9	24.47	4.66
-14	-3	9	19.88	4.65
14	3	-9	15.00	4.41
-13	-3	9	0.91	2.59
13	3	-9	0.63	2.73
-12	-3	9	16.03	3.95
12	3	-9	8.07	3.46
-11	-3	9	65.57	7.27
11	3	-9	63.85	7.06
-10	-3	9	4.38	2.66
10	3	-9	3.64	2.76
-9	-3	9	6.54	2.83
9	3	-9	9.70	3.20
-8	-3	9	31.66	4.66
8	3	-9	22.17	4.09
-7	-3	9	38.45	4.97
7	3	-9	37.55	4.95
-6	-3	9	75.52	5.93
-6	3	9	80.80	2.63
6	3	-9	64.42	5.49
-5	-3	9	38.21	4.84
-5	3	9	48.20	2.57
5	3	-9	38.40	4.77
-5	3	9	43.12	2.49
-4	-3	9	87.77	6.16
4	-3	-9	85.03	3.79
-4	3	9	87.92	3.83
4	3	-9	98.58	6.39
3	-3	-9	783.07	14.93
-3	-3	9	768.86	19.21
3	3	-9	757.46	18.63

-3	3	9	792.62	15.15
-3	3	9	815.29	15.15
-2	-3	9	61.40	5.08
2	-3	-9	58.59	3.70
2	3	-9	55.70	5.50
-2	3	9	63.01	3.79
-2	3	9	57.47	3.69
-1	-3	9	224.52	9.24
1	-3	-9	222.67	7.41
-1	3	9	218.02	7.47
-1	3	9	217.02	7.25
1	3	-9	229.05	8.94
0	-3	9	658.84	16.93
0	-3	-9	666.96	15.30
0	3	9	698.74	15.52
0	3	9	708.88	15.23
0	3	-9	691.55	16.56
1	-3	9	3.96	2.01
-1	-3	-9	2.51	1.55
-1	3	-9	1.86	1.62
1	3	9	0.74	1.44
-2	-3	-9	208.76	8.31
2	-3	9	208.90	8.56
-2	3	-9	204.07	8.05
2	3	9	204.75	7.95
3	-3	9	96.56	5.78
-3	-3	-9	92.30	5.87
-3	3	-9	85.85	5.25
3	3	9	81.50	5.37
-4	-3	-9	12.18	3.00
4	-3	9	11.63	2.63
4	3	9	12.35	2.96
-4	3	-9	9.24	2.33
-5	-3	-9	98.67	6.54
5	-3	9	92.43	5.85
-5	3	-9	101.68	5.70
5	3	9	95.54	6.17
6	-3	9	69.37	5.00
-6	-3	-9	71.98	5.91
6	3	9	61.18	6.13
-7	-3	-9	8.91	3.07
7	-3	9	10.37	2.69
7	3	9	5.04	2.50
-8	-3	-9	170.66	9.54
8	-3	9	169.07	7.85
8	3	9	172.20	9.13
-9	-3	-9	36.48	5.67
9	-3	9	41.21	4.62
-9	3	-9	32.25	4.49
9	3	9	40.19	5.72

10	-3	9	49.02	5.13
-10	-3	-9	42.15	6.16
-10	3	-9	44.15	5.26
10	3	9	42.42	6.07
-11	-3	-9	116.93	8.82
11	-3	9	113.98	6.77
-11	3	-9	113.69	7.17
11	3	9	115.70	8.46
12	-3	9	1.60	1.92
-12	-3	-9	-0.64	2.23
12	3	9	-2.09	2.51
-12	3	-9	-1.00	1.88
-13	-3	-9	55.13	7.96
13	3	9	39.41	6.46
-12	-4	9	-0.17	2.79
12	4	-9	-0.26	2.37
-11	-4	9	9.33	3.78
11	4	-9	7.58	3.45
-10	-4	9	20.45	4.63
10	4	-9	10.97	4.13
-9	-4	9	3.32	2.82
9	4	-9	5.13	3.14
-8	-4	9	33.09	5.16
8	4	-9	36.35	5.52
-7	-4	9	14.70	3.64
7	4	-9	18.80	4.28
-6	-4	9	6.24	2.75
6	4	-9	3.60	2.60
-5	-4	9	46.37	5.91
5	4	-9	38.73	5.32
-4	-4	9	397.74	14.15
4	-4	-9	380.60	8.40
4	4	-9	390.10	13.51
-3	-4	9	1.01	2.19
3	-4	-9	1.29	1.34
3	4	-9	3.59	2.59
2	-4	-9	219.12	7.29
-2	-4	9	195.57	9.54
-2	4	9	215.33	7.18
2	4	-9	213.52	9.50
1	-4	-9	295.04	9.22
-1	-4	9	285.14	11.35
-1	4	9	291.79	9.02
1	4	-9	288.67	10.91
0	-4	-9	1.09	1.52
0	-4	9	-0.45	1.70
0	4	-9	-1.21	1.70
0	4	9	0.87	1.46
1	-4	9	113.35	6.91
-1	-4	-9	120.39	6.38

1	4	9	113.67	6.12
-1	4	-9	112.49	6.54
2	-4	9	-0.12	1.59
-2	-4	-9	0.08	1.50
-2	4	-9	-1.82	1.62
2	4	9	-0.46	1.45
-3	-4	-9	155.01	7.97
3	-4	9	138.86	7.48
-3	4	-9	138.83	7.09
3	4	9	158.20	7.83
-4	-4	-9	0.77	1.92
4	-4	9	2.01	1.83
-4	4	-9	1.68	1.80
4	4	9	2.15	2.11
-5	-4	-9	2.83	2.29
5	-4	9	0.87	1.91
-5	4	-9	4.07	2.04
5	4	9	1.66	2.27
6	-4	9	82.36	5.89
-6	-4	-9	90.66	6.92
-6	4	-9	93.57	5.65
6	4	9	97.42	6.83
7	-4	9	20.89	3.58
-7	-4	-9	27.41	5.12
7	4	9	15.34	3.82
-7	4	-9	17.08	3.19
8	-4	9	4.79	2.17
-8	-4	-9	1.01	2.07
-8	4	-9	0.87	1.80
8	4	9	-0.01	2.08
-9	-4	-9	63.24	7.69
9	-4	9	74.31	5.60
9	4	9	67.41	7.53
-10	-4	-9	39.89	6.47
10	-4	9	44.73	4.98
10	4	9	40.47	6.30
-11	-4	-9	0.15	2.41
11	4	9	3.46	3.10
-9	-5	9	6.20	3.71
9	5	-9	4.93	3.41
-8	-5	9	8.49	3.62
8	5	-9	-2.02	2.52
-7	-5	9	2.05	2.63
7	5	-9	2.10	2.84
-6	-5	9	14.77	4.08
6	5	-9	14.57	4.06
-5	-5	9	10.96	3.66
5	5	-9	8.98	3.26
-4	-5	9	2.42	2.34
4	5	-9	3.38	2.48

-3	-5	9	10.43	3.53
3	5	-9	13.24	3.60
-2	-5	9	46.27	5.94
2	5	-9	31.50	4.90
-1	-5	9	-0.01	1.89
1	-5	-9	-0.54	1.36
1	5	-9	0.59	2.16
0	-5	-9	0.42	1.63
0	-5	9	5.67	2.59
0	5	-9	1.74	2.24
0	5	9	4.88	2.30
-1	-5	-9	0.63	1.87
1	-5	9	1.50	2.11
1	5	9	0.63	1.78
-1	5	-9	1.24	1.83
-2	-5	-9	1.77	2.00
2	-5	9	2.74	2.37
2	5	9	1.19	1.93
-2	5	-9	2.26	2.14
-3	-5	-9	4.75	2.42
3	-5	9	7.31	2.73
3	5	9	4.33	2.51
-3	5	-9	5.84	2.60
4	-5	9	1.67	2.03
-4	-5	-9	1.01	2.25
4	5	9	2.78	2.43
-4	5	-9	2.10	2.07
5	-5	9	37.28	4.92
-5	-5	-9	46.55	6.20
-5	5	-9	46.29	5.11
5	5	9	41.78	5.77
-6	-5	-9	5.90	2.83
6	-5	9	8.62	2.76
6	5	9	9.47	3.41
-7	-5	-9	2.35	2.33
7	-5	9	-0.52	1.88
7	5	9	-0.83	1.98
-8	-5	-9	-1.09	2.11
8	5	9	2.59	2.61
16	0	-10	-2.22	2.22
15	0	-10	17.20	3.65
14	0	-10	39.24	4.82
-13	0	10	18.95	3.23
13	0	-10	8.56	2.75
12	0	-10	-0.13	1.85
-12	0	10	2.59	1.87
11	0	-10	849.35	18.85
-11	0	10	890.12	19.13
10	0	-10	24.41	3.49
-10	0	10	27.72	3.49

9	0	-10	130.93	6.20
-9	0	10	134.12	6.26
-8	0	10	101.02	5.33
8	0	-10	108.02	5.55
7	0	-10	286.38	8.94
-7	0	10	283.25	8.94
7	0	-10	284.32	8.94
-6	0	10	622.94	14.33
6	0	-10	647.36	14.31
6	0	-10	624.01	14.22
5	0	-10	620.42	13.98
-5	0	10	604.15	13.97
5	0	-10	610.08	13.79
-4	0	10	1569.50	26.99
4	0	-10	1533.13	26.63
-3	0	10	588.83	13.60
-2	0	10	185.52	6.97
1	0	-10	554.92	13.51
-1	0	10	569.16	13.28
0	0	10	52.23	3.78
0	0	-10	54.69	3.99
1	0	10	199.52	7.24
-1	0	-10	191.97	7.55
2	0	10	683.39	15.26
-2	0	-10	667.77	15.90
-3	0	-10	3.65	1.61
3	0	10	1.50	1.36
-4	0	-10	49.99	4.22
4	0	10	48.47	3.89
5	0	10	413.09	12.03
-5	0	-10	444.29	13.06
-6	0	-10	33.70	4.32
6	0	10	24.68	3.51
-7	0	-10	127.60	7.15
7	0	10	120.97	6.62
8	0	10	12.76	3.02
-8	0	-10	16.68	3.48
9	0	10	-0.57	1.84
-9	0	-10	-1.90	1.28
10	0	10	354.31	12.48
-10	0	-10	333.84	12.83
11	0	10	66.70	6.62
-11	0	-10	72.96	6.22
-12	0	-10	99.56	7.39
12	0	10	111.93	7.34
13	0	10	68.24	7.04
-13	0	-10	79.86	6.90
-14	0	-10	37.23	5.86
14	0	10	28.85	5.00
-15	0	-10	23.91	5.10

15	0	10	26.60	5.10
-15	-1	10	0.09	2.29
15	1	-10	-0.03	2.24
-14	-1	10	-1.07	1.85
14	1	-10	1.92	2.09
-13	-1	10	11.00	3.09
13	1	-10	10.65	3.21
12	-1	-10	6.15	2.06
-12	-1	10	4.48	2.40
12	1	-10	7.62	2.79
-11	-1	10	89.88	6.04
11	-1	-10	89.94	4.96
-11	1	10	89.19	4.61
11	1	-10	95.55	6.25
10	-1	-10	19.02	2.74
-10	-1	10	8.11	2.57
-10	1	10	16.09	2.44
10	1	-10	17.33	3.38
-9	-1	10	5.99	2.22
9	-1	-10	11.27	2.22
-9	1	10	13.14	2.16
9	1	-10	10.95	2.91
-8	-1	10	6.25	2.16
8	-1	-10	5.97	1.82
8	1	-10	8.59	2.53
-8	1	10	10.02	1.95
7	-1	-10	20.08	2.60
-7	-1	10	16.53	3.07
7	1	-10	13.51	2.84
-7	1	10	21.77	2.54
6	-1	-10	28.55	3.00
-6	-1	10	28.13	3.57
6	1	-10	27.30	3.67
-6	1	10	24.63	2.70
-5	-1	10	171.15	7.33
5	-1	-10	153.79	5.88
5	1	-10	155.67	6.81
-5	1	10	171.26	6.09
-4	-1	10	1.40	1.53
-4	1	10	1.47	1.15
4	1	-10	0.32	1.31
3	-1	-10	24.55	2.98
-3	-1	10	30.72	3.66
-3	1	10	18.39	2.48
3	1	-10	26.11	3.32
2	-1	-10	52.99	3.66
-2	-1	10	62.60	4.36
-2	1	10	60.62	3.81
2	1	-10	56.96	4.05
-1	-1	10	2.41	1.51

1	-1	-10	2.01	1.26
-1	1	10	1.58	1.31
0	-1	10	39.49	4.01
0	-1	-10	43.77	3.56
0	1	10	43.82	3.53
1	-1	10	70.65	4.54
-1	-1	-10	57.75	4.10
1	1	10	60.75	4.08
-1	1	-10	68.00	4.61
-2	-1	-10	223.76	8.40
2	-1	10	230.72	8.33
2	1	10	228.34	8.05
-2	1	-10	233.84	8.62
3	-1	10	127.67	6.17
-3	-1	-10	119.81	6.22
-3	1	-10	109.54	6.00
3	1	10	116.04	5.89
-4	-1	-10	22.06	3.31
4	-1	10	35.76	4.02
-4	1	-10	25.12	3.49
4	1	10	26.05	3.54
-5	-1	-10	15.38	3.08
5	-1	10	14.84	2.76
5	1	10	23.18	3.46
-5	1	-10	16.18	3.02
6	-1	10	397.91	11.79
-6	-1	-10	395.33	13.04
-6	1	-10	407.94	12.71
6	1	10	387.95	12.18
7	-1	10	24.36	3.64
-7	-1	-10	32.20	4.57
-7	1	-10	37.28	4.67
7	1	10	26.28	4.10
-8	-1	-10	215.59	10.02
8	-1	10	228.87	9.08
8	1	10	235.44	10.00
-8	1	-10	218.48	9.54
9	-1	10	181.53	8.37
-9	-1	-10	196.81	9.82
-9	1	-10	191.14	9.09
9	1	10	183.27	9.05
10	-1	10	6.53	2.61
-10	-1	-10	11.53	3.41
10	1	10	9.44	3.21
-10	1	-10	8.43	2.91
11	-1	10	11.56	3.04
-11	-1	-10	4.29	2.64
-11	1	-10	6.93	2.77
11	1	10	6.52	2.94
12	-1	10	16.98	3.65

-12	-1	-10	20.14	4.42
-12	1	-10	16.90	3.83
12	1	10	21.71	4.39
-13	-1	-10	12.16	3.55
13	-1	10	18.22	3.93
13	1	10	24.14	4.82
-13	1	-10	30.45	5.11
14	-1	10	46.76	5.81
-14	-1	-10	42.90	6.53
-14	1	-10	41.74	6.06
14	1	10	37.28	5.88
15	-1	10	9.98	3.44
-15	-1	-10	6.57	3.40
15	1	10	4.77	3.31
-15	1	-10	1.75	2.61
-15	-2	10	15.12	4.21
15	2	-10	18.59	4.49
-14	-2	10	12.55	3.82
14	2	-10	8.77	3.46
-13	-2	10	0.94	2.10
13	2	-10	4.47	2.78
-12	-2	10	145.34	8.60
12	2	-10	147.71	8.73
-11	-2	10	85.80	6.57
11	2	-10	89.74	6.64
-10	-2	10	17.29	3.50
10	2	-10	15.43	3.46
-9	-2	10	279.45	11.18
9	2	-10	273.39	10.89
8	-2	-10	57.55	3.29
-8	-2	10	69.28	5.53
8	2	-10	53.62	5.58
-8	2	10	66.04	3.01
7	-2	-10	5.18	1.51
-7	-2	10	2.62	2.11
7	2	-10	2.68	2.05
-7	2	10	3.71	1.16
-6	-2	10	132.63	7.21
6	-2	-10	137.22	4.96
6	2	-10	131.77	7.03
-6	2	10	133.58	4.71
-5	-2	10	4.27	2.09
5	2	-10	7.47	2.19
-5	2	10	4.90	1.39
4	-2	-10	165.69	5.86
-4	-2	10	160.80	7.65
-4	2	10	172.83	5.80
4	2	-10	161.23	7.41
-3	-2	10	28.53	3.76
3	-2	-10	32.67	2.83

3	2	-10	36.60	4.16
-2	-2	10	77.84	5.18
2	-2	-10	69.24	4.16
-2	2	10	72.14	4.07
2	2	-10	69.22	4.80
1	-2	-10	362.96	10.05
-1	-2	10	360.66	11.34
-1	2	10	378.25	9.96
1	2	-10	358.63	10.95
0	-2	10	93.15	5.57
0	-2	-10	102.15	5.26
0	2	10	98.88	5.11
0	2	-10	95.35	5.44
-1	-2	-10	43.84	3.71
1	-2	10	39.06	4.12
1	2	10	46.90	3.65
-1	2	-10	43.79	4.43
-2	-2	-10	253.25	9.14
2	-2	10	258.62	9.32
2	2	10	256.45	8.80
-3	-2	-10	70.19	5.03
3	-2	10	72.27	4.90
3	2	10	62.85	4.59
-4	-2	-10	3.15	1.76
4	-2	10	-0.12	1.34
-4	2	-10	1.89	1.84
4	2	10	1.78	1.76
5	-2	10	13.64	2.71
-5	-2	-10	9.59	2.67
-5	2	-10	12.12	2.79
5	2	10	15.55	3.13
-6	-2	-10	12.91	3.12
6	-2	10	11.65	2.63
6	2	10	11.19	2.86
-6	2	-10	17.47	3.38
7	-2	10	60.85	4.84
-7	-2	-10	67.69	6.89
7	2	10	44.17	5.24
-7	2	-10	74.82	5.50
8	-2	10	49.93	5.10
-8	-2	-10	50.66	6.19
8	2	10	35.08	5.08
-8	2	-10	55.74	5.00
9	-2	10	31.18	4.22
-9	-2	-10	31.43	5.02
9	2	10	32.62	4.99
-9	2	-10	27.79	4.29
-10	-2	-10	145.93	9.16
10	-2	10	133.02	7.37
10	2	10	142.30	8.76

-10	2	-10	145.77	8.23
-11	-2	-10	-0.87	2.09
11	-2	10	-0.09	1.62
11	2	10	5.55	3.01
-11	2	-10	5.11	2.32
12	-2	10	4.70	2.47
12	-2	10	4.00	2.46
-12	-2	-10	4.06	2.90
12	2	10	3.69	2.78
-12	2	-10	8.31	3.04
13	-2	10	4.55	2.61
-13	-2	-10	6.82	3.35
13	-2	10	4.97	2.62
13	2	10	0.80	2.44
-13	2	-10	2.75	2.35
-14	-2	-10	33.53	6.24
14	-2	10	45.16	5.60
14	2	10	43.10	6.79
-14	2	-10	45.94	6.16
-13	-3	10	7.15	3.40
13	3	-10	6.64	3.28
-12	-3	10	19.89	4.54
12	3	-10	31.86	5.48
-11	-3	10	3.61	2.73
11	3	-10	-1.96	1.96
-10	-3	10	32.17	4.95
10	3	-10	32.23	5.40
-9	-3	10	0.20	1.92
9	3	-10	2.96	2.49
-8	-3	10	17.38	3.80
8	3	-10	16.93	3.81
-7	-3	10	14.35	3.48
7	3	-10	16.93	3.71
-6	-3	10	66.62	5.75
-6	3	10	72.80	2.97
6	3	-10	68.94	5.72
-5	-3	10	0.18	1.76
5	3	-10	2.42	2.07
-5	3	10	1.10	1.00
4	-3	-10	3.74	1.38
-4	-3	10	4.86	2.28
4	3	-10	5.75	2.60
-4	3	10	3.20	1.29
-3	-3	10	13.23	3.09
3	-3	-10	14.08	2.29
-3	3	10	16.07	2.37
-3	3	10	17.16	2.43
3	3	-10	10.88	2.87
-2	-3	10	2.20	1.91
2	-3	-10	3.50	1.59

-2	3	10	0.49	1.33
2	3	-10	-1.91	1.57
-2	3	10	2.94	1.56
-1	-3	10	15.59	3.15
1	-3	-10	14.23	2.48
1	3	-10	15.99	3.19
-1	3	10	10.29	2.22
-1	3	10	15.84	2.63
0	-3	10	372.94	12.31
0	-3	-10	359.57	10.61
0	3	-10	365.60	11.64
0	3	10	351.27	10.36
1	-3	10	165.65	7.99
-1	-3	-10	177.85	7.65
1	3	10	170.59	7.25
-1	3	-10	168.64	7.83
-2	-3	-10	0.92	1.52
2	-3	10	3.35	1.97
-2	3	-10	0.11	1.64
2	3	10	1.40	1.63
-3	-3	-10	88.94	5.85
3	-3	10	96.14	5.97
3	3	10	89.02	5.76
-3	3	-10	86.14	5.55
4	-3	10	67.28	5.08
-4	-3	-10	73.73	5.58
4	3	10	60.79	5.12
-4	3	-10	62.26	4.92
5	-3	10	26.60	3.89
-5	-3	-10	30.85	4.74
-5	3	-10	27.17	3.89
5	3	10	27.15	4.15
6	-3	10	63.05	5.13
-6	-3	-10	49.87	5.92
6	3	10	73.67	5.83
-7	-3	-10	22.54	4.35
7	-3	10	20.79	3.57
7	3	10	23.09	4.22
-8	-3	-10	1.27	2.16
8	-3	10	0.05	1.76
8	3	10	-0.15	2.15
9	-3	10	14.25	3.05
-9	-3	-10	18.50	4.42
-9	3	-10	14.53	3.48
9	3	10	9.66	3.34
-10	-3	-10	80.59	7.24
10	-3	10	67.69	5.54
-10	3	-10	67.54	5.71
10	3	10	56.51	6.94
-11	-3	-10	23.09	5.09

11	-3	10	21.00	3.85
-11	3	-10	15.46	3.73
11	3	10	18.91	4.47
12	-3	10	6.12	2.58
-12	-3	-10	4.34	3.23
12	3	10	4.02	3.21
-12	3	-10	2.64	2.42
-13	-3	-10	4.40	3.05
-13	3	-10	1.87	2.54
13	3	10	5.54	3.55
-11	-4	10	17.38	4.58
11	4	-10	9.22	4.04
-10	-4	10	6.70	3.47
10	4	-10	10.89	3.92
-9	-4	10	11.25	3.72
9	4	-10	21.41	4.72
-8	-4	10	10.09	3.57
8	4	-10	10.69	3.59
-7	-4	10	15.22	3.88
7	4	-10	20.18	4.57
-6	-4	10	104.39	7.67
6	4	-10	106.49	7.58
-5	-4	10	10.41	3.29
5	4	-10	6.62	2.97
-4	-4	10	36.69	5.34
4	4	-10	30.13	4.79
3	-4	-10	137.25	5.50
-3	-4	10	135.14	8.26
3	4	-10	126.30	7.93
2	-4	-10	17.34	2.77
-2	-4	10	13.73	3.37
-2	4	10	14.88	2.60
2	4	-10	17.87	3.70
1	-4	-10	44.09	3.86
-1	-4	10	43.43	5.43
-1	4	10	41.76	4.16
1	4	-10	31.46	4.59
0	-4	-10	5.47	2.05
0	-4	10	5.15	2.43
0	4	10	6.97	2.23
0	4	-10	1.50	2.04
1	-4	10	1.47	1.84
-1	-4	-10	0.78	1.60
-1	4	-10	3.91	2.12
1	4	10	5.35	2.13
-2	-4	-10	10.16	2.77
2	-4	10	5.53	2.29
2	4	10	9.55	2.62
-2	4	-10	14.10	3.33
3	-4	10	12.18	3.04

-3	-4	-10	13.18	3.18
3	4	10	18.22	3.50
-3	4	-10	13.74	3.24
-4	-4	-10	8.60	2.90
4	-4	10	5.10	2.43
-4	4	-10	5.29	2.47
4	4	10	9.83	2.98
-5	-4	-10	39.29	5.38
5	-4	10	43.12	5.07
-5	4	-10	47.40	5.15
5	4	10	47.34	5.72
-6	-4	-10	2.67	2.21
6	-4	10	3.95	2.08
6	4	10	5.59	2.63
-6	4	-10	2.99	2.08
7	-4	10	3.30	2.21
-7	-4	-10	2.65	2.48
-7	4	-10	1.42	1.87
7	4	10	3.00	2.53
-8	-4	-10	14.02	3.96
8	-4	10	8.74	2.83
8	4	10	11.46	3.81
-8	4	-10	12.87	2.94
-9	-4	-10	-1.28	2.20
9	-4	10	2.70	2.11
9	4	10	-1.65	2.33
10	-4	10	8.54	3.04
-10	-4	-10	19.11	5.04
10	4	10	3.35	2.93
-11	-4	-10	22.28	5.32
11	4	10	41.23	6.91
-8	-5	10	6.61	3.36
8	5	-10	2.06	2.86
-7	-5	10	10.11	3.66
7	5	-10	11.97	4.17
-6	-5	10	5.59	3.30
6	5	-10	2.21	2.70
-5	-5	10	18.73	4.36
5	5	-10	21.59	4.49
-4	-5	10	10.25	3.51
4	5	-10	16.38	3.92
-3	-5	10	-1.04	1.89
3	5	-10	0.79	2.11
-2	-5	10	57.12	6.82
2	5	-10	50.01	6.25
1	-5	-10	0.90	1.67
-1	-5	10	3.87	2.66
1	5	-10	2.38	2.35
0	-5	-10	14.86	3.11
0	-5	10	14.75	3.82

0	5	-10	16.20	3.77
0	5	10	13.11	2.99
-1	-5	-10	51.48	5.55
1	-5	10	60.43	6.63
1	5	10	38.37	4.80
-1	5	-10	45.27	5.64
-2	-5	-10	-1.06	1.69
2	-5	10	-0.12	1.95
-2	5	-10	1.30	2.16
2	5	10	0.11	1.92
-3	-5	-10	32.67	4.98
3	-5	10	30.62	4.81
-3	5	-10	29.71	4.62
3	5	10	26.47	4.46
4	-5	10	58.67	6.29
-4	-5	-10	55.04	6.59
4	5	10	57.55	6.50
-4	5	-10	53.33	5.89
-5	-5	-10	0.66	2.15
5	-5	10	0.22	1.90
5	5	10	0.09	2.16
-5	5	-10	0.65	1.91
6	-5	10	34.71	4.96
-6	-5	-10	43.31	6.22
-6	5	-10	39.35	4.85
6	5	10	31.16	5.25
7	-5	10	10.50	3.14
-7	-5	-10	7.06	3.05
7	5	10	12.82	4.25
15	0	-11	0.61	2.19
14	0	-11	3.10	2.35
-13	0	11	0.62	1.71
13	0	-11	2.15	2.21
-12	0	11	2.88	1.85
12	0	-11	2.66	1.89
11	0	-11	0.66	1.77
-11	0	11	1.23	1.85
-10	0	11	1.10	1.58
10	0	-11	-1.54	1.52
9	0	-11	-0.10	1.49
-9	0	11	-1.16	1.37
-8	0	11	0.44	1.36
8	0	-11	-1.04	1.45
-7	0	11	-1.20	1.25
7	0	-11	3.52	1.79
6	0	-11	-0.75	1.33
-6	0	11	0.61	1.35
5	0	-11	-0.28	1.27
-5	0	11	0.26	1.30
-4	0	11	2.30	1.48

4	0	-11	-1.62	1.11
-3	0	11	0.96	1.26
-2	0	11	-0.15	1.22
1	0	-11	4.01	1.73
-1	0	11	4.66	1.74
0	0	-11	-0.95	1.06
0	0	11	0.34	1.22
-1	0	-11	1.11	1.48
1	0	11	2.80	1.59
2	0	11	0.43	1.32
-2	0	-11	2.40	1.45
3	0	11	0.22	1.40
-3	0	-11	1.94	1.63
4	0	11	0.43	1.34
-4	0	-11	-1.04	1.19
5	0	11	-0.24	1.44
-5	0	-11	-2.25	1.08
6	0	11	-1.58	1.25
-6	0	-11	-1.02	1.30
7	0	11	0.53	1.73
-7	0	-11	-2.70	1.27
8	0	11	-2.63	1.61
-8	0	-11	2.07	2.04
-9	0	-11	0.68	1.95
9	0	11	-1.78	1.65
-10	0	-11	-1.76	1.72
10	0	11	2.35	2.23
11	0	11	1.19	2.06
-11	0	-11	0.97	2.18
12	0	11	5.95	2.89
-12	0	-11	-1.33	1.77
13	0	11	0.03	2.21
-13	0	-11	4.33	2.86
-14	0	-11	3.75	2.72
14	0	11	-0.31	2.18
-15	0	-11	1.81	2.67
15	0	11	-1.09	2.11
-15	-1	11	4.92	2.79
15	1	-11	7.35	3.17
-14	-1	11	-0.12	2.23
14	1	-11	2.26	2.42
-13	-1	11	43.19	5.27
13	1	-11	41.57	5.38
-12	-1	11	138.11	7.77
12	-1	-11	124.92	6.03
12	1	-11	132.64	7.58
-11	-1	11	40.55	4.95
11	-1	-11	43.72	4.20
-11	1	11	39.94	3.38
11	1	-11	42.98	5.16

10	-1	-11	175.58	6.77
-10	-1	11	164.64	8.13
10	1	-11	159.34	7.93
-10	1	11	156.87	6.21
-9	-1	11	212.29	9.05
9	-1	-11	201.45	7.24
-9	1	11	204.04	7.06
9	1	-11	224.78	9.20
-8	-1	11	20.99	3.48
8	-1	-11	24.03	3.02
-8	1	11	23.56	2.80
8	1	-11	24.54	3.82
-7	-1	11	26.19	3.74
7	-1	-11	31.54	3.37
7	1	-11	22.72	3.62
-7	1	11	31.06	3.19
6	-1	-11	158.62	6.13
-6	-1	11	151.14	7.22
6	1	-11	160.21	7.41
-6	1	11	155.51	6.07
5	-1	-11	35.87	3.64
-5	-1	11	32.58	3.91
5	1	-11	34.86	4.13
-5	1	11	41.64	3.36
-4	-1	11	4.07	1.94
4	1	-11	9.72	2.41
-4	1	11	7.33	1.78
-3	-1	11	64.13	4.71
3	-1	-11	60.86	4.15
-3	1	11	53.35	3.81
3	1	-11	53.94	4.29
2	-1	-11	47.78	3.71
-2	-1	11	46.44	4.00
2	1	-11	48.96	4.69
-2	1	11	49.39	3.66
-1	-1	11	366.07	11.15
1	-1	-11	364.74	10.63
0	-1	-11	10.72	2.43
0	-1	11	8.75	2.32
0	1	11	9.25	2.13
1	-1	11	118.62	6.10
-1	-1	-11	108.74	5.85
-1	1	-11	125.45	6.49
1	1	11	127.35	6.01
-2	-1	-11	2.86	1.77
2	-1	11	5.33	1.85
-2	1	-11	4.00	1.80
2	1	11	6.52	1.96
-3	-1	-11	0.47	1.36
3	-1	11	0.36	1.33

3	1	11	0.50	1.43
-3	1	-11	0.26	1.42
-4	-1	-11	261.20	10.02
4	-1	11	269.35	9.76
-4	1	-11	276.72	10.24
4	1	11	282.29	9.89
5	-1	11	62.43	4.75
-5	-1	-11	63.17	5.16
5	1	11	65.21	5.15
-5	1	-11	71.59	5.40
6	-1	11	2.21	1.72
-6	-1	-11	-1.83	1.39
-6	1	-11	-1.08	1.40
6	1	11	5.35	2.46
-7	-1	-11	114.70	7.30
7	-1	11	110.67	6.47
-7	1	-11	118.36	7.13
7	1	11	105.68	6.74
-8	-1	-11	368.69	13.69
8	-1	11	349.12	11.98
8	1	11	391.79	13.23
-8	1	-11	349.49	12.72
9	-1	11	0.99	2.00
-9	-1	-11	9.02	3.18
-9	1	-11	5.96	2.67
9	1	11	6.95	2.83
-10	-1	-11	61.83	6.99
10	-1	11	78.24	6.00
10	1	11	80.10	6.67
-10	1	-11	67.41	6.06
-11	-1	-11	41.69	6.15
11	-1	11	34.87	4.92
-11	1	-11	43.51	5.92
11	1	11	36.88	5.43
12	-1	11	-2.19	1.78
-12	-1	-11	1.48	2.39
12	1	11	-0.38	2.14
-12	1	-11	0.89	2.08
-13	-1	-11	43.95	6.73
13	-1	11	36.24	5.22
13	1	11	30.53	5.45
-13	1	-11	44.97	6.31
14	-1	11	-0.95	2.04
-14	-1	-11	4.91	3.22
-14	1	-11	5.56	3.00
14	1	11	2.53	2.75
-14	-2	11	0.37	2.50
14	2	-11	6.68	3.31
-13	-2	11	9.63	3.32
13	2	-11	10.03	3.43

-12	-2	11	1.63	2.34
12	2	-11	-0.26	2.25
-11	-2	11	20.91	4.09
11	2	-11	24.89	4.62
-10	-2	11	24.33	4.16
10	2	-11	29.73	4.76
-9	-2	11	3.75	2.44
9	2	-11	6.14	2.87
8	-2	-11	11.45	2.05
-8	-2	11	9.52	2.89
-8	2	11	11.41	1.75
8	2	-11	8.62	3.05
-7	-2	11	4.56	2.25
7	-2	-11	6.73	1.71
-7	2	11	4.64	1.38
7	2	-11	2.62	2.07
6	-2	-11	19.28	2.56
-6	-2	11	20.66	3.61
-6	2	11	17.43	2.24
6	2	-11	10.04	2.90
-5	-2	11	96.92	6.30
-5	2	11	99.01	4.60
5	2	-11	101.10	6.28
-4	-2	11	90.56	6.02
-4	2	11	103.02	4.76
4	2	-11	95.80	6.06
-3	-2	11	-0.28	1.48
3	-2	-11	0.08	1.22
-3	2	11	1.09	1.25
3	2	-11	-1.87	1.16
2	-2	-11	297.95	9.18
-2	-2	11	306.15	10.79
2	2	-11	308.88	10.49
-2	2	11	302.94	9.02
-1	-2	11	0.03	1.42
1	-2	-11	0.63	1.26
-1	2	11	-0.70	1.22
1	2	-11	0.08	1.61
0	-2	-11	213.62	8.07
0	-2	11	204.28	8.55
0	2	-11	202.81	8.41
0	2	11	202.03	7.57
1	-2	11	6.82	2.27
-1	-2	-11	8.81	2.27
1	2	11	11.00	2.39
-1	2	-11	6.82	2.35
-2	-2	-11	12.61	2.75
2	-2	11	12.48	2.64
2	2	11	11.53	2.55
3	-2	11	65.06	4.87

-3	-2	-11	48.65	5.21
3	2	11	51.29	4.32
-4	-2	-11	-0.75	1.31
4	-2	11	3.93	2.02
4	2	11	3.41	1.96
-4	2	-11	3.31	1.97
-5	-2	-11	4.18	2.15
5	-2	11	1.58	1.64
5	2	11	3.15	2.28
-5	2	-11	1.77	1.89
-6	-2	-11	32.15	4.75
6	-2	11	25.73	3.89
-6	2	-11	26.61	4.27
6	2	11	32.36	4.57
-7	-2	-11	134.49	8.14
7	-2	11	140.72	7.40
7	2	11	146.92	8.27
-7	2	-11	128.70	7.44
-8	-2	-11	-1.90	1.49
8	-2	11	0.39	1.69
8	2	11	0.05	2.01
-8	2	-11	-0.33	1.79
-9	-2	-11	22.37	4.58
9	-2	11	11.67	3.09
-9	2	-11	13.98	3.55
9	2	11	10.29	3.41
10	-2	11	31.99	4.51
-10	-2	-11	26.37	5.12
-10	2	-11	23.67	4.25
10	2	11	24.12	4.57
-11	-2	-11	-0.22	2.23
11	-2	11	-1.17	1.61
-11	2	-11	0.47	2.04
11	2	11	-1.56	2.15
-12	-2	-11	0.51	2.44
12	-2	11	-1.86	1.91
12	2	11	-0.85	2.35
-12	2	-11	-3.25	1.44
-13	-2	-11	-1.81	2.11
13	-2	11	2.38	2.41
-13	2	-11	-0.71	2.20
13	2	11	2.01	2.80
-14	-2	-11	4.83	3.35
14	-2	11	3.59	2.54
14	2	11	-1.04	2.73
-14	2	-11	1.82	2.60
-13	-3	11	7.06	3.55
13	3	-11	10.11	3.82
-12	-3	11	15.65	4.22
12	3	-11	12.06	3.80

-11	-3	11	5.31	2.99
11	3	-11	12.92	3.93
-10	-3	11	20.17	4.27
10	3	-11	25.96	5.01
-9	-3	11	112.19	7.89
9	3	-11	119.13	8.22
-8	-3	11	-1.06	2.01
8	3	-11	3.25	2.63
-7	-3	11	161.39	9.07
7	3	-11	173.53	9.21
-6	-3	11	95.65	7.00
6	3	-11	93.88	6.91
-5	-3	11	-0.67	1.85
-5	3	11	-0.27	1.02
5	3	-11	-2.97	1.54
4	-3	-11	87.06	4.43
-4	-3	11	81.19	6.18
-4	3	11	88.11	4.36
4	3	-11	83.68	6.19
-3	-3	11	21.71	3.77
3	-3	-11	26.65	3.11
-3	3	11	20.08	2.86
3	3	-11	24.69	4.06
-3	3	11	20.82	2.77
2	-3	-11	23.11	3.12
-2	-3	11	20.74	3.74
2	3	-11	22.96	3.85
-2	3	11	21.79	2.97
-2	3	11	29.12	3.39
-1	-3	11	11.20	2.98
1	-3	-11	11.52	2.38
1	3	-11	11.15	2.93
-1	3	11	15.45	2.75
0	-3	-11	12.76	2.65
0	-3	11	7.73	2.49
0	3	-11	17.39	3.41
0	3	11	20.38	3.21
1	-3	11	5.50	2.28
-1	-3	-11	-0.58	1.50
-1	3	-11	2.36	1.98
1	3	11	0.73	1.60
2	-3	11	86.40	5.98
-2	-3	-11	84.24	5.69
2	3	11	87.68	5.64
-2	3	-11	85.86	5.78
-3	-3	-11	11.24	2.86
3	-3	11	23.43	3.79
-3	3	-11	21.88	3.67
3	3	11	15.71	3.30
4	-3	11	199.58	9.05

-4	-3	-11	207.87	9.48
4	3	11	218.77	9.48
-4	3	-11	226.32	9.11
5	-3	11	82.12	5.92
-5	-3	-11	71.88	5.92
-5	3	-11	67.83	5.20
5	3	11	84.73	6.14
-6	-3	-11	0.03	1.92
6	-3	11	2.60	1.89
6	3	11	0.80	2.05
-7	-3	-11	156.78	9.18
7	-3	11	174.81	8.51
7	3	11	167.37	9.23
8	-3	11	28.85	4.34
-8	-3	-11	12.82	3.74
8	3	11	20.88	4.30
9	-3	11	14.79	3.21
-9	-3	-11	24.85	5.15
-9	3	-11	23.61	4.23
9	3	11	24.77	4.85
-10	-3	-11	11.39	3.91
10	-3	11	5.56	2.53
-10	3	-11	11.41	3.30
10	3	11	8.67	3.61
-11	-3	-11	31.46	5.90
11	-3	11	19.69	3.87
11	3	11	26.30	5.26
-11	3	-11	20.16	4.14
12	-3	11	2.35	2.40
-12	-3	-11	8.33	3.60
12	3	11	4.53	3.49
-12	3	-11	4.82	2.60
-11	-4	11	18.15	4.89
11	4	-11	18.94	4.89
-10	-4	11	2.67	2.88
10	4	-11	4.27	3.30
-9	-4	11	10.22	3.73
9	4	-11	5.66	3.41
-8	-4	11	87.99	8.74
8	4	-11	88.37	8.79
-7	-4	11	15.56	4.06
7	4	-11	22.23	4.67
-6	-4	11	118.73	8.36
6	4	-11	118.02	8.14
-5	-4	11	109.01	7.88
5	4	-11	123.69	8.12
-4	-4	11	4.22	2.64
4	4	-11	10.02	3.56
3	-4	-11	9.23	2.08
-3	-4	11	3.92	2.53

3	4	-11	7.51	2.88
2	-4	-11	56.76	4.16
-2	-4	11	58.07	6.44
-2	4	11	45.16	3.78
2	4	-11	53.04	5.97
1	-4	-11	0.47	1.51
-1	-4	11	0.20	1.96
-1	4	11	5.58	2.05
1	4	-11	1.83	2.29
0	-4	11	24.40	4.23
0	-4	-11	24.49	3.62
0	4	11	28.85	3.90
0	4	-11	28.40	4.36
-1	-4	-11	16.22	3.26
1	-4	11	17.34	3.49
-1	4	-11	15.77	3.59
1	4	11	22.21	3.54
-2	-4	-11	1.14	1.87
2	-4	11	1.51	2.08
2	4	11	3.63	2.16
-2	4	-11	1.75	2.36
-3	-4	-11	192.19	9.15
3	-4	11	192.56	9.41
-3	4	-11	200.69	9.07
3	4	11	194.84	9.13
4	-4	11	88.99	6.47
-4	-4	-11	91.86	6.65
4	4	11	88.01	6.57
-4	4	-11	80.01	5.98
-5	-4	-11	21.46	4.26
5	-4	11	11.60	3.11
5	4	11	16.77	3.84
-5	4	-11	16.45	3.42
-6	-4	-11	86.58	7.15
6	-4	11	70.43	5.91
6	4	11	74.00	6.32
-6	4	-11	88.17	5.98
-7	-4	-11	10.91	3.52
7	-4	11	15.54	3.47
-7	4	-11	8.67	2.70
7	4	11	5.75	3.16
8	-4	11	8.45	2.88
-8	-4	-11	11.99	3.77
8	4	11	14.86	4.18
-8	4	-11	8.94	2.79
9	-4	11	39.87	5.16
-9	-4	-11	30.58	5.56
9	4	11	34.40	6.09
-9	4	-11	31.11	4.27
10	-4	11	11.62	3.29

-10	-4	-11	16.87	4.47
10	4	11	12.69	4.23
-7	-5	11	1.08	2.76
7	5	-11	0.24	2.62
-6	-5	11	2.41	2.88
6	5	-11	0.82	2.74
-5	-5	11	2.09	2.54
5	5	-11	-1.38	2.43
-4	-5	11	-2.34	1.98
4	5	-11	1.06	2.40
-3	-5	11	8.31	3.22
3	5	-11	-0.37	2.16
-2	-5	11	2.36	2.44
2	5	-11	1.32	2.13
-1	-5	11	2.70	2.34
1	5	-11	2.18	2.36
0	-5	11	1.20	2.09
0	-5	-11	4.65	2.26
0	5	-11	1.24	2.12
0	5	11	4.86	2.38
-1	-5	-11	4.32	2.42
1	-5	11	4.67	2.46
-1	5	-11	6.71	2.75
1	5	11	6.14	2.70
-2	-5	-11	0.17	1.90
2	-5	11	0.27	2.10
2	5	11	1.42	2.09
-2	5	-11	1.21	2.18
-3	-5	-11	3.82	2.46
3	-5	11	1.51	2.23
-3	5	-11	-1.27	1.87
3	5	11	1.10	2.23
-4	-5	-11	0.41	2.18
4	-5	11	-0.78	1.95
4	5	11	-1.72	2.05
-4	5	-11	0.35	1.94
5	-5	11	-1.06	2.02
-5	-5	-11	-0.70	1.84
-5	5	-11	4.20	2.57
5	5	11	2.16	2.56
6	-5	11	-0.95	1.98
-6	-5	-11	1.31	2.24
-6	5	-11	3.68	2.33
6	5	11	1.87	2.76
7	-5	11	-1.28	1.94
-7	-5	-11	1.19	2.60
15	0	-12	76.51	6.00
14	0	-12	0.60	2.26
13	0	-12	51.38	5.43
-12	0	12	90.29	5.83

12	0	-12	97.01	6.04
11	0	-12	13.54	3.00
-11	0	12	9.62	2.54
10	0	-12	11.24	2.84
-10	0	12	9.96	2.56
-9	0	12	148.64	6.99
9	0	-12	147.66	7.09
-8	0	12	0.44	1.46
8	0	-12	-1.04	1.47
-7	0	12	205.87	8.11
7	0	-12	215.24	8.23
-6	0	12	149.72	6.89
6	0	-12	147.44	6.65
5	0	-12	55.82	4.36
-5	0	12	56.76	4.41
-4	0	12	649.94	15.53
4	0	-12	623.49	15.10
-3	0	12	14.42	2.76
-2	0	12	228.35	8.48
-1	0	12	1043.90	21.34
1	0	-12	1041.12	21.87
0	0	-12	2.75	1.73
0	0	12	0.03	1.39
-1	0	-12	532.54	14.58
2	0	12	2079.69	35.93
-2	0	-12	2077.48	37.03
3	0	12	12.59	2.90
-3	0	-12	18.59	3.42
4	0	12	180.15	8.09
-4	0	-12	193.53	8.80
-5	0	-12	46.65	5.23
5	0	12	58.49	4.90
6	0	12	137.49	7.47
-6	0	-12	144.40	8.00
7	0	12	193.17	9.10
-7	0	-12	193.81	9.57
-8	0	-12	189.73	9.72
8	0	12	175.94	8.88
-9	0	-12	269.94	11.97
9	0	12	265.04	11.18
-10	0	-12	31.97	5.22
10	0	12	26.27	4.55
11	0	12	4.83	2.73
-11	0	-12	-0.62	1.92
12	0	12	4.75	2.70
-12	0	-12	-0.61	2.05
13	0	12	20.34	4.36
-13	0	-12	23.18	4.84
-14	0	-12	1.58	2.52
14	0	12	1.90	2.72

-14	-1	12	3.95	2.63
14	1	-12	1.16	2.52
-13	-1	12	0.87	2.24
13	1	-12	-1.01	2.00
12	-1	-12	62.36	4.55
-12	-1	12	67.69	5.81
12	1	-12	65.01	6.52
11	-1	-12	22.19	3.36
-11	-1	12	20.29	3.84
11	1	-12	32.26	4.71
10	-1	-12	10.38	2.55
-10	-1	12	11.29	2.98
-10	1	12	7.54	2.16
10	1	-12	6.33	2.63
-9	-1	12	47.06	5.10
9	-1	-12	45.10	4.32
9	1	-12	36.75	4.81
-9	1	12	45.43	3.66
-8	-1	12	82.60	5.77
8	-1	-12	92.80	5.11
-8	1	12	90.81	4.89
8	1	-12	95.67	6.29
7	-1	-12	-1.19	1.25
-7	-1	12	1.39	1.78
7	1	-12	2.07	1.86
-7	1	12	2.63	1.56
-6	-1	12	10.49	2.57
6	-1	-12	10.32	2.34
6	1	-12	6.03	2.29
-6	1	12	6.77	1.99
-5	-1	12	24.20	3.67
5	-1	-12	24.85	3.30
-5	1	12	25.61	3.20
5	1	-12	22.94	3.63
-4	-1	12	18.09	3.19
4	1	-12	9.43	2.54
-4	1	12	14.25	2.45
3	-1	-12	67.73	4.53
-3	-1	12	63.76	4.89
3	1	-12	71.83	4.99
-3	1	12	69.64	4.53
-2	-1	12	27.68	3.67
2	-1	-12	34.85	4.02
-2	1	12	34.11	3.80
2	1	-12	31.52	3.99
-1	-1	12	0.02	1.39
1	-1	-12	5.19	1.84
-1	1	12	2.24	1.67
0	-1	-12	-1.66	1.10
0	-1	12	1.90	1.83

0	1	12	3.42	1.76
-1	-1	-12	93.24	5.69
1	-1	12	92.08	5.71
1	1	12	91.18	5.42
-1	1	-12	83.47	5.66
2	-1	12	2.03	1.58
-2	-1	-12	0.35	1.43
2	1	12	1.90	1.63
-2	1	-12	-0.93	1.15
3	-1	12	35.53	4.32
-3	-1	-12	35.67	4.67
-3	1	-12	37.67	4.69
3	1	12	29.45	3.98
-4	-1	-12	2.65	1.81
4	-1	12	-1.72	1.22
-4	1	-12	-0.18	1.64
4	1	12	-1.75	1.34
5	-1	12	1.73	1.73
-5	-1	-12	-0.03	1.56
5	1	12	0.48	1.79
-5	1	-12	3.19	2.13
-6	-1	-12	11.87	3.08
6	-1	12	12.11	2.98
-6	1	-12	7.61	2.62
6	1	12	11.58	3.07
7	-1	12	38.13	4.86
-7	-1	-12	36.03	5.29
-7	1	-12	34.77	4.95
7	1	12	36.83	4.92
-8	-1	-12	18.80	3.97
8	-1	12	10.70	3.11
-8	1	-12	9.89	3.10
8	1	12	12.21	3.30
9	-1	12	25.73	4.36
-9	-1	-12	21.01	4.40
9	1	12	19.77	4.00
-9	1	-12	23.59	4.47
10	-1	12	11.73	3.36
-10	-1	-12	15.80	3.97
10	1	12	9.77	3.40
-10	1	-12	12.61	3.53
11	-1	12	12.41	3.53
-11	-1	-12	9.72	3.33
11	1	12	11.97	3.65
-11	1	-12	7.07	2.97
12	-1	12	7.72	3.07
-12	-1	-12	5.97	3.26
12	1	12	6.42	3.09
-12	1	-12	5.75	2.91
13	-1	12	4.64	2.80

-13	-1	-12	3.82	3.12
13	1	12	-0.16	2.51
-13	1	-12	2.69	2.58
-14	-1	-12	5.03	3.39
14	-1	12	8.06	3.13
-14	1	-12	5.19	3.01
14	1	12	6.29	3.58
-14	-2	12	8.95	3.56
14	2	-12	13.58	4.06
-13	-2	12	4.89	2.97
13	2	-12	-2.84	2.11
-12	-2	12	0.70	2.32
12	2	-12	8.73	3.35
-11	-2	12	75.83	6.53
11	2	-12	77.91	6.57
-10	-2	12	1.27	2.18
10	2	-12	5.08	2.74
-9	-2	12	1.80	2.06
9	2	-12	2.99	2.42
-8	-2	12	21.86	3.93
8	-2	-12	32.36	3.24
-8	2	12	21.72	2.51
8	2	-12	17.87	3.78
7	-2	-12	58.63	3.78
-7	-2	12	51.40	5.60
7	2	-12	72.04	5.98
-7	2	12	57.83	3.58
6	-2	-12	46.49	3.62
-6	-2	12	43.40	5.14
6	2	-12	44.93	5.38
-6	2	12	43.57	3.31
-5	-2	12	35.72	4.63
-5	2	12	34.04	3.31
5	2	-12	32.18	4.67
-4	-2	12	197.28	9.00
-4	2	12	198.99	7.03
4	2	-12	181.05	8.70
3	-2	-12	297.32	9.40
-3	-2	12	313.62	11.43
-3	2	12	327.13	9.52
3	2	-12	318.17	11.27
-2	-2	12	295.42	10.98
2	-2	-12	287.00	9.51
2	2	-12	312.12	10.85
-1	-2	12	48.56	5.14
1	-2	-12	56.73	4.44
-1	2	12	51.55	4.02
1	2	-12	62.33	4.98
0	-2	12	111.78	6.64
0	-2	-12	120.44	6.32

0	2	12	107.20	5.74
0	2	-12	117.26	6.71
1	-2	12	262.33	10.08
-1	-2	-12	247.42	9.39
1	2	12	238.77	8.98
-2	-2	-12	70.85	5.26
2	-2	12	69.14	5.26
2	2	12	66.65	5.02
-3	-2	-12	34.91	4.49
3	-2	12	34.16	4.28
3	2	12	35.30	4.39
4	-2	12	85.95	5.89
-4	-2	-12	99.96	6.55
4	2	12	79.89	5.74
-4	2	-12	90.71	6.28
5	-2	12	26.42	4.00
-5	-2	-12	30.10	4.72
5	2	12	32.38	4.50
-5	2	-12	23.49	3.94
-6	-2	-12	118.46	7.62
6	-2	12	113.34	6.92
-6	2	-12	99.68	6.93
6	2	12	107.21	6.99
7	-2	12	106.31	6.88
-7	-2	-12	115.85	7.81
-7	2	-12	115.95	7.32
7	2	12	105.95	7.25
-8	-2	-12	8.37	3.21
8	-2	12	8.77	2.78
-8	2	-12	11.60	3.13
8	2	12	10.15	3.17
9	-2	12	64.16	6.43
-9	-2	-12	62.81	7.38
-9	2	-12	54.31	6.22
9	2	12	66.00	7.18
-10	-2	-12	30.28	5.29
10	-2	12	27.09	4.51
10	2	12	30.22	5.25
-10	2	-12	30.06	5.23
11	-2	12	0.24	1.86
-11	-2	-12	-0.10	2.20
-11	2	-12	-0.18	2.07
11	2	12	1.61	2.57
-12	-2	-12	13.48	4.27
12	-2	12	18.71	3.88
-12	2	-12	20.65	4.50
12	2	12	19.36	4.54
-13	-2	-12	-2.55	2.12
13	-2	12	2.32	2.43
-13	2	-12	6.31	3.17

13	2	12	2.24	2.90
-12	-3	12	1.86	3.03
12	3	-12	10.72	4.05
-11	-3	12	4.32	3.06
11	3	-12	4.39	2.95
-10	-3	12	3.89	2.88
10	3	-12	1.61	2.73
-9	-3	12	9.01	3.29
9	3	-12	5.61	2.91
-8	-3	12	40.81	5.71
8	3	-12	49.44	6.39
-7	-3	12	-1.72	1.89
7	3	-12	0.71	2.33
-6	-3	12	101.75	7.37
6	3	-12	114.59	7.64
-5	-3	12	77.68	6.34
-5	3	12	74.55	4.04
5	3	-12	59.24	6.48
-4	-3	12	26.81	4.38
4	-3	-12	28.35	3.28
4	3	-12	23.21	4.23
-4	3	12	29.79	3.19
3	-3	-12	230.07	8.03
-3	-3	12	243.97	10.74
-2	-3	12	120.15	7.40
2	-3	-12	99.17	5.45
2	3	-12	118.17	7.36
-2	3	12	109.89	5.64
1	-3	-12	66.73	4.72
-1	-3	12	74.20	5.88
1	3	-12	73.44	5.81
-1	3	12	62.94	4.55
0	-3	-12	59.26	4.69
0	-3	12	58.90	6.10
0	3	-12	56.26	5.83
0	3	12	64.20	4.82
-1	-3	-12	8.73	2.56
1	-3	12	9.68	2.78
-1	3	-12	15.98	3.41
1	3	12	10.51	2.70
2	-3	12	0.98	1.88
-2	-3	-12	-0.57	1.44
-2	3	-12	0.66	1.80
2	3	12	1.08	1.76
-3	-3	-12	10.53	2.90
3	-3	12	11.05	2.96
3	3	12	8.16	2.70
-3	3	-12	10.93	2.89
-4	-3	-12	29.45	4.58
4	-3	12	24.04	4.03

4	3	12	23.60	4.02
-4	3	-12	28.73	4.38
5	-3	12	22.21	3.75
-5	-3	-12	28.03	4.58
-5	3	-12	30.18	4.24
5	3	12	29.69	4.68
6	-3	12	83.23	6.14
-6	-3	-12	67.81	6.09
6	3	12	61.16	6.52
7	-3	12	1.80	1.95
-7	-3	-12	-0.78	1.72
7	3	12	1.11	2.28
-8	-3	-12	22.10	4.59
8	-3	12	18.41	3.62
8	3	12	19.55	4.36
-9	-3	-12	8.14	3.12
9	-3	12	4.39	2.46
-9	3	-12	5.07	2.70
9	3	12	4.83	3.33
-10	-3	-12	5.48	3.07
10	-3	12	13.77	3.41
10	3	12	12.30	4.02
-10	3	-12	16.33	3.87
-11	-3	-12	8.68	3.70
11	-3	12	6.24	2.76
11	3	12	9.46	3.90
-11	3	-12	1.51	2.60
12	-3	12	3.96	2.64
-12	-3	-12	3.66	3.38
12	3	12	0.83	3.17
-12	3	-12	5.24	3.21
-10	-4	12	2.86	2.97
10	4	-12	2.75	3.10
-9	-4	12	-0.03	2.42
9	4	-12	3.15	2.87
-8	-4	12	-1.65	2.29
8	4	-12	0.59	2.72
-7	-4	12	10.06	3.58
7	4	-12	9.72	3.61
-6	-4	12	0.87	2.50
6	4	-12	2.92	2.62
-5	-4	12	1.80	2.20
5	4	-12	-1.44	2.09
-4	-4	12	32.74	5.24
4	4	-12	21.70	4.42
3	-4	-12	8.37	2.12
-3	-4	12	6.61	2.79
3	4	-12	8.73	3.39
-2	-4	12	20.31	4.03
2	-4	-12	21.50	3.25

-2	4	12	23.43	3.36
2	4	-12	25.59	4.62
1	-4	-12	79.20	5.29
-1	-4	12	94.13	7.22
1	4	-12	90.20	6.77
-1	4	12	99.16	5.79
0	-4	-12	2.36	1.83
0	-4	12	-0.14	1.92
0	4	-12	0.84	1.98
0	4	12	0.57	1.75
-1	-4	-12	84.04	5.77
1	-4	12	77.75	6.49
1	4	12	79.85	5.71
-1	4	-12	78.85	6.39
-2	-4	-12	26.05	4.17
2	-4	12	23.46	4.29
-2	4	-12	17.74	3.73
2	4	12	25.45	4.10
3	-4	12	39.33	5.16
-3	-4	-12	41.78	5.36
3	4	12	35.49	5.01
-3	4	-12	38.99	5.20
4	-4	12	39.36	5.20
-4	-4	-12	38.11	5.23
4	4	12	38.91	5.39
-4	4	-12	31.01	4.68
5	-4	12	8.36	2.96
-5	-4	-12	5.42	2.72
-5	4	-12	2.33	2.11
5	4	12	5.09	2.71
-6	-4	-12	20.78	4.44
6	-4	12	22.58	4.24
6	4	12	21.11	4.42
-6	4	-12	21.42	3.82
7	-4	12	18.79	3.84
-7	-4	-12	11.17	3.63
7	4	12	22.87	4.85
-7	4	-12	16.51	3.63
8	-4	12	0.01	1.98
-8	-4	-12	-0.37	1.88
8	4	12	0.64	2.61
-8	4	-12	2.42	2.26
-9	-4	-12	6.66	3.39
9	-4	12	6.74	2.86
-9	4	-12	8.52	2.90
9	4	12	16.00	4.55
-6	-5	12	7.96	3.59
6	5	-12	10.28	3.92
-5	-5	12	15.10	4.13
5	5	-12	21.57	4.84

-4	-5	12	4.53	3.25
4	5	-12	0.59	2.70
-3	-5	12	15.20	4.27
3	5	-12	14.32	4.19
-2	-5	12	8.97	3.45
2	5	-12	7.92	3.31
-1	-5	12	7.55	3.27
1	5	-12	3.85	2.98
0	-5	12	28.09	5.13
0	-5	-12	18.83	3.53
0	5	-12	17.53	4.05
-1	-5	-12	2.15	2.27
1	-5	12	4.74	2.93
1	5	12	5.57	2.73
-1	5	-12	4.49	2.80
2	-5	12	5.71	2.89
-2	-5	-12	4.79	2.50
-2	5	-12	4.84	2.83
2	5	12	2.24	2.56
3	-5	12	8.44	3.18
-3	-5	-12	4.25	2.71
-3	5	-12	0.67	2.23
3	5	12	3.01	2.66
-4	-5	-12	1.48	2.35
4	-5	12	1.04	2.27
-4	5	-12	0.88	2.31
4	5	12	3.26	2.88
5	-5	12	4.56	2.72
-5	-5	-12	0.81	2.38
-5	5	-12	2.22	2.42
5	5	12	0.94	2.54
14	0	-13	-0.59	2.04
13	0	-13	0.47	2.08
-12	0	13	-0.87	1.74
12	0	-13	1.37	2.05
11	0	-13	1.18	2.04
-11	0	13	0.11	1.70
-10	0	13	-0.05	1.66
10	0	-13	-0.16	1.54
-9	0	13	2.09	1.76
9	0	-13	0.48	1.82
-8	0	13	1.29	1.69
8	0	-13	0.97	1.69
7	0	-13	1.56	1.75
-7	0	13	0.03	1.58
-6	0	13	0.30	1.65
6	0	-13	-2.59	1.20
-5	0	13	-1.15	1.22
5	0	-13	-1.29	1.51
-4	0	13	1.07	1.58

4	0	-13	-0.21	1.51
-3	0	13	-0.09	1.34
-2	0	13	0.64	1.50
0	0	13	-0.93	1.39
0	0	-13	-2.03	1.32
1	0	13	3.96	2.07
-1	0	-13	4.76	2.07
-3	0	-13	0.77	1.81
3	0	13	-2.29	1.46
-4	0	-13	-1.88	1.45
4	0	13	0.82	1.97
5	0	13	-1.00	1.73
-5	0	-13	-2.17	1.28
6	0	13	-0.29	1.82
-6	0	-13	-1.41	1.61
-7	0	-13	-0.24	1.79
7	0	13	-5.55	1.28
-8	0	-13	1.42	2.17
8	0	13	2.52	2.27
-9	0	-13	2.64	2.47
9	0	13	5.50	2.80
-10	0	-13	2.15	2.51
10	0	13	0.23	2.21
-11	0	-13	0.17	2.13
11	0	13	1.77	2.23
12	0	13	-1.59	2.28
-12	0	-13	-2.20	1.85
-13	0	-13	0.60	2.72
13	0	13	-3.44	2.22
-14	-1	13	-0.77	2.31
14	1	-13	-1.75	2.11
-13	-1	13	32.59	4.91
13	1	-13	26.57	4.58
12	-1	-13	36.49	4.24
-12	-1	13	34.60	4.92
12	1	-13	29.33	4.68
11	-1	-13	23.89	3.48
-11	-1	13	16.82	3.63
11	1	-13	15.57	3.66
10	-1	-13	133.04	6.38
-10	-1	13	126.01	7.45
-10	1	13	140.37	6.31
10	1	-13	132.24	7.67
9	-1	-13	103.32	5.77
-9	-1	13	103.61	6.68
9	1	-13	109.67	6.80
-9	1	13	107.82	5.58
-8	-1	13	0.00	1.70
8	-1	-13	2.61	1.80
-8	1	13	2.82	1.63

8	1	-13	3.53	2.34
-7	-1	13	34.71	4.57
7	-1	-13	36.44	4.01
-7	1	13	29.40	3.55
7	1	-13	32.47	4.51
-6	-1	13	6.95	2.49
6	-1	-13	12.91	2.67
-6	1	13	7.28	2.08
6	1	-13	11.13	2.94
-5	-1	13	238.08	9.73
5	-1	-13	245.60	8.61
-5	1	13	246.47	8.68
5	1	-13	264.42	10.03
-4	-1	13	84.25	5.78
-4	1	13	93.62	5.36
4	1	-13	88.11	5.80
-3	-1	13	32.25	4.23
-3	1	13	42.41	4.35
3	1	-13	40.31	4.74
-2	-1	13	213.94	9.03
2	-1	-13	207.91	8.39
-2	1	13	224.80	8.38
2	1	-13	200.24	8.58
1	-1	-13	197.88	8.28
-1	-1	13	181.11	8.31
-1	1	13	187.50	7.84
0	-1	-13	17.64	3.24
0	-1	13	17.59	3.28
1	-1	13	332.82	11.45
-1	-1	-13	321.04	11.26
-1	1	-13	339.67	11.94
1	1	13	320.49	10.77
2	-1	13	37.55	4.55
-2	-1	-13	34.59	4.55
-2	1	-13	20.66	3.58
2	1	13	33.12	4.31
3	-1	13	106.89	6.51
-3	-1	-13	112.86	6.80
-3	1	-13	95.18	6.47
3	1	13	109.22	6.53
-4	-1	-13	103.99	6.84
4	-1	13	107.25	6.63
4	1	13	117.16	6.86
-4	1	-13	107.74	6.95
5	-1	13	8.65	2.68
-5	-1	-13	3.61	2.30
-5	1	-13	2.00	2.08
5	1	13	8.61	2.93
-6	-1	-13	33.65	4.95
6	-1	13	49.18	5.48

6	1	13	38.59	5.14
-6	1	-13	51.20	5.97
-7	-1	-13	15.49	3.73
7	-1	13	4.60	2.59
-7	1	-13	11.53	3.30
7	1	13	10.12	3.05
8	-1	13	219.15	10.01
-8	-1	-13	233.01	11.24
-8	1	-13	215.51	10.65
8	1	13	215.90	10.42
9	-1	13	1.95	2.20
-9	-1	-13	-0.22	2.17
9	1	13	1.45	2.45
-9	1	-13	0.10	2.01
-10	-1	-13	-3.50	1.45
10	-1	13	1.08	2.06
-10	1	-13	1.31	2.15
10	1	13	-0.70	2.03
-11	-1	-13	52.83	7.07
11	-1	13	72.48	7.31
11	1	13	63.45	7.42
-11	1	-13	53.11	6.71
12	-1	13	93.00	7.35
-12	-1	-13	62.94	7.79
12	1	13	87.81	8.87
-12	1	-13	81.88	7.23
-13	-1	-13	8.58	3.73
13	-1	13	6.36	3.23
13	1	13	11.33	4.14
-13	1	-13	3.30	2.88
-13	-2	13	48.16	6.54
13	2	-13	51.19	6.73
-12	-2	13	19.30	4.42
12	2	-13	12.16	3.69
-11	-2	13	5.08	2.93
11	2	-13	5.40	3.04
-10	-2	13	83.42	6.95
10	2	-13	81.77	6.87
-9	-2	13	36.83	5.30
9	2	-13	26.90	4.66
8	-2	-13	20.41	2.95
-8	-2	13	26.57	4.45
8	2	-13	24.82	4.44
-7	-2	13	1.65	2.21
7	-2	-13	3.70	1.90
7	2	-13	0.84	2.08
-7	2	13	2.50	1.51
-6	-2	13	3.83	2.33
6	-2	-13	2.20	1.75
-6	2	13	3.68	1.67

6	2	-13	2.77	2.28
-5	-2	13	13.64	3.29
5	2	-13	10.29	3.15
-5	2	13	7.07	1.93
-4	-2	13	15.17	3.45
4	2	-13	13.72	3.28
-4	2	13	13.47	2.59
3	-2	-13	152.79	6.87
-3	-2	13	174.29	8.69
-3	2	13	144.58	6.53
3	2	-13	156.75	8.04
2	-2	-13	58.47	4.47
-2	-2	13	63.62	5.43
2	2	-13	47.93	5.31
-1	-2	13	23.02	3.87
1	-2	-13	22.95	3.45
-1	2	13	19.41	3.22
1	2	-13	25.36	4.19
0	-2	-13	-0.19	1.52
0	-2	13	3.94	2.17
0	2	13	-0.15	1.47
0	2	-13	-1.15	1.68
-1	-2	-13	5.22	2.16
1	-2	13	1.47	1.99
1	2	13	0.10	1.54
2	-2	13	0.55	1.63
-2	-2	-13	1.62	1.87
2	2	13	2.02	1.82
3	-2	13	13.11	3.07
-3	-2	-13	4.19	2.30
3	2	13	9.27	2.81
-4	-2	-13	4.15	2.34
4	-2	13	-0.73	1.79
4	2	13	2.70	1.90
-4	2	-13	-0.31	1.59
-5	-2	-13	85.98	6.51
5	-2	13	97.45	6.63
-5	2	-13	95.44	6.79
5	2	13	75.21	5.98
-6	-2	-13	2.85	2.28
6	-2	13	4.47	2.41
6	2	13	6.17	2.79
-6	2	-13	6.23	2.78
7	-2	13	39.43	5.12
-7	-2	-13	28.64	4.95
7	2	13	42.21	5.65
-7	2	-13	42.42	5.82
-8	-2	-13	11.73	3.56
8	-2	13	11.19	3.10
8	2	13	10.61	3.39

-8	2	-13	14.65	3.65
9	-2	13	6.01	2.54
-9	-2	-13	3.40	2.60
9	2	13	2.05	2.77
-9	2	-13	4.11	2.44
10	-2	13	1.23	2.08
-10	-2	-13	2.82	2.89
10	2	13	1.67	2.46
-10	2	-13	0.61	2.31
11	-2	13	5.09	2.57
-11	-2	-13	1.92	2.77
11	2	13	2.23	2.78
-11	2	-13	2.89	2.65
-12	-2	-13	3.91	3.28
12	-2	13	6.66	3.00
-12	2	-13	3.92	2.77
12	2	13	7.74	3.80
-12	-3	13	0.29	2.65
12	3	-13	0.23	2.69
-11	-3	13	33.24	5.77
11	3	-13	33.95	5.82
-10	-3	13	21.03	4.38
10	3	-13	29.57	5.69
-9	-3	13	-2.63	1.82
9	3	-13	4.06	3.02
-8	-3	13	23.52	4.84
8	3	-13	21.52	4.37
-7	-3	13	18.92	4.08
7	3	-13	17.90	4.10
-6	-3	13	29.85	4.98
6	3	-13	25.05	4.67
-5	-3	13	58.48	6.49
5	3	-13	68.84	7.20
-5	3	13	68.46	4.20
4	-3	-13	242.05	8.19
-4	3	13	224.45	7.84
4	3	-13	243.59	10.87
-3	-3	13	7.30	2.96
3	-3	-13	10.91	2.58
3	3	-13	11.69	3.30
-2	-3	13	380.11	13.78
2	-3	-13	373.60	11.33
2	3	-13	387.54	13.59
-2	3	13	359.33	11.04
-1	-3	13	284.96	11.76
1	-3	-13	304.23	10.36
1	3	-13	305.29	11.76
-1	3	13	289.04	10.07
0	-3	13	143.87	8.29
0	-3	-13	153.74	7.48

0	3	13	161.57	7.56
0	3	-13	161.30	8.49
1	-3	13	227.90	10.29
-1	-3	-13	204.21	9.02
-1	3	-13	217.68	9.72
1	3	13	220.76	9.23
2	-3	13	9.68	2.86
-2	-3	-13	11.15	3.01
-2	3	-13	8.60	2.90
2	3	13	5.87	2.50
-3	-3	-13	69.45	5.79
3	-3	13	68.15	5.84
-3	3	-13	56.70	5.98
3	3	13	71.15	5.63
-4	-3	-13	13.98	3.45
4	-3	13	13.13	3.23
4	3	13	13.00	3.39
-4	3	-13	11.59	3.21
5	-3	13	1.30	1.92
-5	-3	-13	1.46	2.03
5	3	13	4.15	2.42
-5	3	-13	2.48	2.07
6	-3	13	15.86	3.57
-6	-3	-13	15.09	3.78
6	3	13	13.72	3.67
7	-3	13	-0.72	1.84
-7	-3	-13	2.55	2.30
7	3	13	1.89	2.63
-8	-3	-13	3.51	2.66
8	-3	13	1.77	2.08
8	3	13	6.19	3.19
9	-3	13	5.53	2.74
-9	-3	-13	14.15	4.13
9	3	13	8.26	3.47
10	-3	13	-2.36	1.95
-10	-3	-13	0.70	2.37
-10	3	-13	0.06	2.25
10	3	13	7.71	3.57
-11	-3	-13	0.27	2.41
11	-3	13	0.37	2.15
-11	3	-13	-1.00	2.10
11	3	13	0.35	2.71
-9	-4	13	17.06	4.91
9	4	-13	17.59	4.65
-8	-4	13	-0.34	2.29
8	4	-13	0.18	2.80
-7	-4	13	1.64	2.86
7	4	-13	-1.62	2.21
-6	-4	13	16.12	4.34
6	4	-13	13.01	4.04

-5	-4	13	16.58	4.17
5	4	-13	10.33	3.89
-4	-4	13	13.37	3.84
4	4	-13	17.05	4.00
3	-4	-13	39.80	4.18
-3	-4	13	33.56	5.33
3	4	-13	34.23	5.40
2	-4	-13	0.45	1.61
-2	-4	13	2.95	2.42
2	4	-13	0.75	2.25
-2	4	13	-0.36	1.72
-1	-4	13	67.42	7.30
1	-4	-13	70.59	5.08
-1	4	13	63.34	4.86
0	-4	-13	108.83	6.52
0	-4	13	105.20	7.64
0	4	13	93.81	6.05
0	4	-13	115.91	7.78
1	-4	13	5.13	2.66
-1	-4	-13	4.50	2.41
1	4	13	0.75	2.01
-1	4	-13	5.97	2.85
2	-4	13	67.49	7.07
-2	-4	-13	63.98	5.59
-2	4	-13	47.03	5.75
2	4	13	61.27	6.22
3	-4	13	22.86	4.32
-3	-4	-13	11.53	3.20
-3	4	-13	22.03	4.15
3	4	13	17.41	3.79
4	-4	13	-1.08	1.82
-4	-4	-13	4.00	2.47
4	4	13	3.11	2.50
-4	4	-13	2.31	2.06
5	-4	13	38.71	5.37
-5	-4	-13	29.38	4.96
-5	4	-13	34.12	4.92
5	4	13	46.51	6.13
6	-4	13	17.82	3.90
-6	-4	-13	19.24	4.39
6	4	13	28.50	5.05
-6	4	-13	23.16	4.23
-7	-4	-13	-1.62	1.99
7	-4	13	1.60	2.32
7	4	13	1.18	2.41
-7	4	-13	0.29	2.12
-8	-4	-13	55.54	7.38
8	-4	13	42.04	5.68
-8	4	-13	38.66	5.15
8	4	13	24.19	5.07

-4	-5	13	0.61	2.33
4	5	-13	1.88	2.68
-3	-5	13	0.59	2.58
3	5	-13	-1.39	2.50
-2	-5	13	6.51	3.14
2	5	-13	3.18	2.72
-1	-5	13	-0.33	2.33
1	5	-13	0.95	2.59
0	-5	13	14.87	4.11
0	5	-13	13.88	3.95
1	-5	13	3.19	2.60
-1	-5	-13	-1.98	1.79
-1	5	-13	1.89	2.49
1	5	13	0.46	2.11
-2	-5	-13	0.14	2.09
2	-5	13	-0.41	2.28
2	5	13	-1.31	2.03
-2	5	-13	-0.55	2.21
3	-5	13	1.56	2.48
-3	-5	-13	1.18	2.23
3	5	13	1.98	2.49
-3	5	-13	0.74	2.38
4	-5	13	8.07	3.23
-4	-5	-13	8.42	3.27
4	5	13	2.98	2.85
-4	5	-13	2.54	2.47
14	0	-14	33.87	4.93
13	0	-14	13.25	3.48
-12	0	14	-1.33	1.83
12	0	-14	-1.09	2.01
-11	0	14	98.23	6.34
11	0	-14	114.87	6.88
10	0	-14	45.12	5.10
-10	0	14	34.90	4.48
-9	0	14	1.86	1.88
9	0	-14	1.11	2.09
-8	0	14	47.41	4.92
8	0	-14	31.03	4.17
7	0	-14	131.25	6.98
-7	0	14	132.46	7.05
-6	0	14	-2.95	1.19
6	0	-14	-0.19	1.72
5	0	-14	39.08	4.59
-5	0	14	34.84	4.35
-4	0	14	71.94	5.23
4	0	-14	63.79	5.07
-3	0	14	12.49	2.81
-2	0	14	360.04	11.85
-1	0	14	19.94	3.49
1	0	-14	18.50	3.50

0	0	-14	235.91	9.80
0	0	14	228.30	9.38
1	0	14	190.21	8.64
-1	0	-14	179.69	8.68
2	0	14	177.06	8.52
-2	0	-14	179.69	8.84
-3	0	-14	160.49	8.53
3	0	14	161.36	8.11
4	0	14	68.97	5.58
-4	0	-14	70.85	6.05
-5	0	-14	77.31	6.35
5	0	14	81.67	6.18
-6	0	-14	366.01	13.80
6	0	14	352.30	13.01
7	0	14	106.64	7.48
-7	0	-14	112.34	7.87
-8	0	-14	168.51	9.73
8	0	14	161.00	9.06
9	0	14	386.38	14.47
-9	0	-14	398.06	15.44
-10	0	-14	57.77	7.23
10	0	14	62.57	7.17
11	0	14	-2.67	2.20
-11	0	-14	0.63	2.44
-12	0	-14	41.19	6.37
12	0	14	49.82	6.77
-13	0	-14	5.01	3.36
13	0	14	6.08	3.38
-13	-1	14	-1.12	2.06
13	1	-14	0.65	2.40
12	-1	-14	5.15	2.37
-12	-1	14	6.33	2.88
12	1	-14	8.63	3.23
11	-1	-14	48.85	4.94
-11	-1	14	44.57	5.48
11	1	-14	47.68	5.80
10	-1	-14	5.22	2.26
-10	-1	14	9.09	2.98
10	1	-14	7.69	2.68
-9	-1	14	13.30	3.33
9	-1	-14	17.02	3.26
-9	1	14	18.32	3.05
9	1	-14	20.51	4.01
-8	-1	14	5.34	2.44
8	-1	-14	6.68	2.31
8	1	-14	6.12	2.76
-8	1	14	4.78	1.95
-7	-1	14	38.02	4.82
7	-1	-14	32.66	4.04
-7	1	14	37.73	4.17

7	1	-14	50.88	5.77
6	-1	-14	50.84	4.89
-6	-1	14	43.66	5.16
-6	1	14	37.64	4.11
6	1	-14	42.98	5.12
-5	-1	14	26.06	4.04
5	-1	-14	33.47	4.15
5	1	-14	32.99	4.59
-5	1	14	30.58	3.83
-4	-1	14	56.43	5.79
-4	1	14	39.30	4.34
4	1	-14	57.53	5.81
-3	-1	14	100.83	6.54
-3	1	14	109.71	6.23
3	1	-14	103.87	6.50
-2	-1	14	0.14	1.84
2	-1	-14	1.70	1.86
-2	1	14	0.42	1.55
2	1	-14	1.63	2.01
-1	-1	14	26.52	3.98
1	-1	-14	30.52	4.23
-1	1	14	29.98	3.99
0	-1	-14	-1.32	1.32
0	-1	14	1.58	1.96
0	1	14	-1.72	1.50
1	-1	14	87.34	6.13
-1	-1	-14	70.92	5.56
-1	1	-14	79.34	5.99
1	1	14	82.28	5.78
2	-1	14	13.91	3.19
-2	-1	-14	12.40	3.17
2	1	14	7.75	2.50
-2	1	-14	11.29	3.14
3	-1	14	-1.39	1.49
-3	-1	-14	-0.19	1.68
3	1	14	5.30	2.36
-3	1	-14	3.15	2.24
-4	-1	-14	12.58	3.28
4	-1	14	14.13	3.21
-4	1	-14	13.06	3.36
4	1	14	14.21	3.41
5	-1	14	47.23	5.45
-5	-1	-14	41.28	5.41
-5	1	-14	42.81	5.66
5	1	14	50.16	5.80
6	-1	14	16.36	3.65
-6	-1	-14	18.63	3.91
-6	1	-14	26.57	4.78
6	1	14	26.10	4.60
-7	-1	-14	13.95	3.95

7	-1	14	16.30	3.58
-7	1	-14	20.37	4.38
7	1	14	13.67	3.65
8	-1	14	139.34	8.29
8	1	14	149.87	8.98
-8	1	-14	133.65	8.70
9	-1	14	24.22	4.49
-9	-1	-14	17.45	4.41
9	1	14	22.59	4.74
-9	1	-14	18.32	4.27
10	-1	14	2.11	2.35
-10	-1	-14	-0.16	2.15
-10	1	-14	3.17	2.86
10	1	14	2.13	2.59
11	-1	14	10.98	3.48
-11	-1	-14	11.91	3.85
11	1	14	12.01	3.88
-11	1	-14	13.17	3.93
12	-1	14	6.41	3.36
-12	-1	-14	9.42	3.82
-12	1	-14	10.08	3.76
12	1	14	17.70	4.67
-13	-1	-14	1.66	2.85
-13	1	-14	4.16	3.09
-12	-2	14	3.61	2.99
12	2	-14	2.35	2.82
-11	-2	14	105.59	7.87
11	2	-14	121.73	8.30
-10	-2	14	30.61	4.93
10	2	-14	30.04	5.04
-9	-2	14	-1.35	1.81
9	2	-14	-0.27	2.18
8	-2	-14	31.75	3.69
-8	-2	14	38.00	5.34
8	2	-14	30.25	5.02
-7	-2	14	90.67	6.91
7	-2	-14	99.25	5.33
-7	2	14	88.79	4.96
7	2	-14	99.47	7.14
-6	-2	14	47.67	5.83
6	-2	-14	41.16	4.26
-6	2	14	40.58	4.03
6	2	-14	35.35	5.07
-5	-2	14	0.92	2.08
5	2	-14	7.18	2.90
-5	2	14	1.48	1.63
-4	-2	14	11.45	3.17
-4	2	14	13.48	2.71
4	2	-14	10.70	3.19
-3	-2	14	0.19	2.09

3	-2	-14	-0.19	1.44
3	2	-14	0.85	2.04
-3	2	14	0.69	1.47
-2	-2	14	2.44	2.20
2	-2	-14	-0.05	1.49
2	2	-14	2.97	2.17
-2	2	14	1.43	1.79
-1	-2	14	13.53	3.30
1	-2	-14	12.26	3.00
-1	2	14	8.95	2.54
1	2	-14	10.98	3.05
0	-2	-14	94.81	6.13
0	-2	14	77.28	6.08
0	2	14	94.33	6.01
0	2	-14	86.96	6.32
-1	-2	-14	74.91	5.65
1	-2	14	67.45	5.61
1	2	14	73.48	5.42
-1	2	-14	78.82	6.07
2	-2	14	6.02	2.37
-2	-2	-14	5.82	2.55
2	2	14	4.69	2.39
3	-2	14	38.33	4.84
-3	-2	-14	37.41	5.16
3	2	14	41.53	5.20
4	-2	14	69.79	5.76
-4	-2	-14	67.18	5.93
-4	2	-14	56.27	6.45
4	2	14	60.23	6.29
5	-2	14	27.83	4.50
-5	-2	-14	29.81	4.87
-5	2	-14	27.46	4.62
5	2	14	24.16	4.35
-6	-2	-14	23.16	4.47
6	-2	14	22.47	4.11
-6	2	-14	25.97	4.71
6	2	14	18.67	4.15
7	-2	14	2.94	2.21
-7	-2	-14	7.21	2.98
-7	2	-14	7.27	2.96
7	2	14	4.58	2.77
8	-2	14	14.53	3.53
-8	-2	-14	7.91	3.29
8	2	14	6.14	3.06
-8	2	-14	14.61	3.81
9	-2	14	24.55	4.41
-9	-2	-14	22.76	4.74
9	2	14	29.50	5.52
-9	2	-14	23.46	4.67
-10	-2	-14	18.80	4.90

10	-2	14	9.32	3.00
10	2	14	14.00	4.27
-10	2	-14	14.60	4.09
-11	-2	-14	3.59	2.92
11	-2	14	0.43	2.13
-11	2	-14	1.63	2.69
11	2	14	1.19	2.89
12	-2	14	7.14	3.28
-12	-2	-14	7.05	3.53
12	2	14	18.41	5.08
-12	2	-14	4.66	3.03
-11	-3	14	13.01	4.20
11	3	-14	17.40	4.66
-10	-3	14	5.53	3.26
10	3	-14	9.32	3.53
-9	-3	14	27.27	5.14
9	3	-14	21.49	4.57
-8	-3	14	1.21	2.30
8	3	-14	-2.96	2.08
-7	-3	14	18.07	4.21
7	3	-14	15.68	4.11
-6	-3	14	25.77	4.74
6	3	-14	32.49	5.38
-5	-3	14	6.86	2.90
5	3	-14	5.53	3.02
-4	-3	14	11.52	3.49
4	-3	-14	14.44	2.77
4	3	-14	13.42	3.62
-4	3	14	15.89	2.76
3	-3	-14	-0.97	1.38
-3	-3	14	2.70	2.42
-3	3	14	0.85	1.55
3	3	-14	2.37	2.55
2	-3	-14	2.20	1.78
-2	-3	14	5.39	2.61
2	3	-14	1.02	2.31
-2	3	14	3.41	1.95
-1	-3	14	70.12	6.10
1	-3	-14	62.55	5.11
-1	3	14	64.02	4.99
1	3	-14	55.55	6.20
0	-3	-14	1.97	2.01
0	-3	14	2.06	2.05
0	3	-14	0.30	2.13
0	3	14	0.85	1.97
-1	-3	-14	39.80	4.99
1	-3	14	33.18	5.09
1	3	14	40.63	4.93
-1	3	-14	38.94	5.32
2	-3	14	71.00	6.08

-2	-3	-14	70.75	5.75
2	3	14	64.52	6.42
3	-3	14	81.38	6.54
-3	-3	-14	76.59	6.20
-3	3	-14	87.49	6.59
3	3	14	89.47	6.43
-4	-3	-14	1.14	2.01
4	-3	14	6.56	2.69
-4	3	-14	3.24	2.40
4	3	14	2.43	2.19
5	-3	14	21.48	4.09
-5	-3	-14	22.95	4.39
-5	3	-14	24.70	4.33
5	3	14	24.75	4.48
6	-3	14	50.49	6.12
-6	-3	-14	32.63	5.40
6	3	14	52.93	6.68
-7	-3	-14	0.48	2.15
7	-3	14	0.13	1.87
7	3	14	1.65	2.52
-8	-3	-14	5.22	3.04
8	-3	14	13.64	3.54
8	3	14	6.82	3.30
9	-3	14	5.82	2.77
-9	-3	-14	6.88	3.45
9	3	14	4.91	3.17
10	-3	14	47.08	6.08
-10	-3	-14	33.37	6.29
10	3	14	38.79	6.46
-8	-4	14	-0.75	2.63
8	4	-14	-4.54	2.30
-7	-4	14	1.24	2.57
7	4	-14	6.84	3.39
-6	-4	14	3.17	3.08
6	4	-14	4.05	3.16
-5	-4	14	19.62	4.47
5	4	-14	20.58	4.63
-4	-4	14	25.64	4.92
4	4	-14	33.36	5.60
-3	-4	14	4.98	2.85
3	4	-14	1.22	2.69
-2	-4	14	6.46	3.06
2	-4	-14	2.34	1.88
-2	4	14	1.51	1.98
2	4	-14	1.56	2.66
-1	-4	14	0.90	2.17
1	-4	-14	0.32	1.74
1	4	-14	3.29	2.58
-1	4	14	-1.16	1.80
0	-4	-14	11.82	2.92

0	-4	14	15.45	3.89
0	4	-14	16.10	4.11
0	4	14	12.14	3.14
1	-4	14	0.05	2.13
-1	-4	-14	0.73	1.83
1	4	14	-0.20	1.86
-1	4	-14	3.30	2.68
-2	-4	-14	0.20	1.78
2	-4	14	3.07	2.42
2	4	14	-1.73	1.71
-2	4	-14	2.28	2.44
3	-4	14	6.23	3.00
-3	-4	-14	9.75	3.22
3	4	14	2.16	2.51
-3	4	-14	5.21	2.70
-4	-4	-14	14.80	3.83
4	-4	14	7.16	3.07
-4	4	-14	12.50	3.56
4	4	14	10.21	3.47
-5	-4	-14	0.98	2.20
5	-4	14	1.55	2.29
-5	4	-14	-0.02	2.18
5	4	14	0.71	2.40
6	-4	14	11.73	3.60
-6	-4	-14	12.95	3.89
-6	4	-14	8.17	3.05
6	4	14	14.24	3.99
7	-4	14	2.79	2.59
-7	-4	-14	5.19	3.06
7	4	14	5.47	3.30
-7	4	-14	5.52	2.87
-1	-5	14	-0.46	2.15
1	5	-14	2.10	2.81
0	-5	14	2.99	2.92
0	5	-14	3.88	2.87
-1	-5	-14	1.62	2.22
13	0	-15	2.43	2.53
12	0	-15	2.39	2.41
-11	0	15	2.44	1.98
11	0	-15	3.64	2.38
-10	0	15	1.15	1.98
10	0	-15	-2.84	1.67
-9	0	15	0.55	1.76
9	0	-15	-1.29	1.61
-8	0	15	0.72	1.85
8	0	-15	-0.17	1.68
-7	0	15	-1.57	1.63
7	0	-15	2.60	2.02
6	0	-15	1.28	1.76
-6	0	15	-1.05	1.70

-5	0	15	-1.26	1.50
5	0	-15	-0.40	1.63
-4	0	15	-1.28	1.60
4	0	-15	1.49	1.95
-3	0	15	1.35	1.76
-2	0	15	5.10	2.28
1	0	-15	2.65	2.10
-1	0	15	1.78	1.88
0	0	15	1.75	1.90
0	0	-15	2.55	2.02
-1	0	-15	1.53	1.85
1	0	15	2.58	2.23
-2	0	-15	3.49	2.22
2	0	15	0.38	1.88
3	0	15	1.09	1.99
-3	0	-15	0.02	1.95
-4	0	-15	-1.00	1.75
4	0	15	1.83	1.99
-5	0	-15	-0.49	1.99
5	0	15	-2.81	1.63
-6	0	-15	0.25	2.02
6	0	15	0.32	2.24
7	0	15	-1.19	2.06
-7	0	-15	-1.19	2.08
8	0	15	-0.15	2.35
-8	0	-15	-1.50	1.87
-9	0	-15	3.25	2.63
9	0	15	2.27	2.63
10	0	15	-1.51	2.28
-10	0	-15	4.90	3.01
11	0	15	-0.17	2.44
-11	0	-15	-0.75	2.28
-12	0	-15	0.87	2.65
12	0	15	-1.30	2.64
-13	-1	15	26.60	4.71
13	1	-15	20.00	4.39
-12	-1	15	5.08	2.79
12	1	-15	9.71	3.38
11	-1	-15	7.24	2.56
-11	-1	15	6.88	2.96
11	1	-15	3.56	2.59
-10	-1	15	-0.39	1.87
10	-1	-15	1.80	2.10
10	1	-15	0.61	2.16
-9	-1	15	3.42	2.40
9	-1	-15	-0.88	1.84
-9	1	15	0.89	1.70
9	1	-15	3.73	2.60
-8	-1	15	2.49	2.14
8	-1	-15	-0.49	1.75

8	1	-15	5.40	2.69
-8	1	15	0.95	1.60
7	-1	-15	16.13	3.27
-7	-1	15	36.23	5.05
-7	1	15	22.44	3.43
7	1	-15	27.96	4.50
6	-1	-15	3.83	2.13
-6	-1	15	-1.19	1.67
6	1	-15	0.80	2.15
-6	1	15	0.83	1.71
-5	-1	15	104.54	7.01
5	-1	-15	103.41	6.19
5	1	-15	94.10	6.67
-5	1	15	96.22	6.03
-4	-1	15	155.53	8.33
4	1	-15	176.22	8.65
-4	1	15	164.45	7.67
-3	-1	15	19.16	3.78
3	1	-15	24.92	4.13
-3	1	15	37.60	4.48
2	-1	-15	401.24	12.97
-2	-1	15	364.64	13.02
-2	1	15	382.22	12.30
2	1	-15	419.76	13.62
-1	-1	15	0.54	1.85
1	-1	-15	0.53	1.81
-1	1	15	-0.38	1.60
0	-1	15	256.38	10.62
0	-1	-15	251.19	10.37
0	1	15	266.36	10.47
-1	-1	-15	182.34	9.05
1	-1	15	186.41	9.13
-1	1	-15	187.63	9.48
-2	-1	-15	1.40	1.79
2	-1	15	-0.14	1.64
2	1	15	4.04	2.36
-2	1	-15	2.15	2.21
3	-1	15	214.14	9.98
-3	-1	-15	226.68	10.56
-3	1	-15	221.83	10.54
3	1	15	227.58	10.17
4	-1	15	12.20	3.23
-4	-1	-15	13.76	3.48
-4	1	-15	10.24	3.24
4	1	15	7.98	2.93
5	-1	15	6.35	2.69
-5	-1	-15	4.18	2.53
5	1	15	10.14	3.10
-5	1	-15	9.61	3.21
-6	-1	-15	21.96	4.43

6	-1	15	33.40	4.94
6	1	15	29.38	4.91
-6	1	-15	20.06	4.41
7	-1	15	12.67	3.42
-7	-1	-15	8.59	3.27
7	1	15	17.29	4.05
-7	1	-15	15.03	3.97
-8	-1	-15	110.30	8.45
8	-1	15	124.13	8.36
8	1	15	121.89	8.59
-8	1	-15	114.65	8.38
-9	-1	-15	23.87	5.08
9	-1	15	33.40	5.36
9	1	15	22.02	4.99
-9	1	-15	25.01	5.12
10	-1	15	2.77	2.65
-10	-1	-15	2.58	2.81
10	1	15	7.33	3.49
-10	1	-15	7.37	3.40
11	-1	15	9.03	3.48
-11	-1	-15	7.51	3.83
-11	1	-15	10.55	3.89
11	1	15	9.04	3.68
12	-1	15	6.91	3.60
-12	-1	-15	6.90	3.73
12	1	15	15.75	4.55
-12	1	-15	10.04	3.96
-12	-2	15	16.11	4.28
12	2	-15	13.03	4.17
-11	-2	15	22.89	4.67
11	2	-15	22.12	4.74
-10	-2	15	17.15	4.18
10	2	-15	14.71	4.11
-9	-2	15	7.57	3.10
9	2	-15	0.27	2.75
8	-2	-15	-0.51	1.60
-8	-2	15	1.07	2.31
8	2	-15	-0.30	2.25
7	-2	-15	19.66	3.26
-7	-2	15	14.94	3.81
7	2	-15	9.87	3.39
6	-2	-15	24.27	3.66
-6	-2	15	19.10	3.99
-6	2	15	21.86	3.27
6	2	-15	21.44	4.53
-5	-2	15	0.45	2.07
-5	2	15	0.88	1.57
5	2	-15	-0.55	2.02
-4	-2	15	43.34	5.54
-4	2	15	45.83	4.62

4	2	-15	42.47	5.53
-3	-2	15	217.82	10.34
3	-2	-15	209.34	8.85
3	2	-15	211.96	10.03
-3	2	15	184.42	8.17
-2	-2	15	6.61	2.80
2	-2	-15	10.15	2.74
2	2	-15	10.63	3.33
-2	2	15	7.22	2.41
-1	-2	15	22.80	4.06
1	-2	-15	15.77	3.34
1	2	-15	13.56	3.53
0	-2	15	17.23	3.67
0	-2	-15	14.95	3.33
0	2	15	15.37	3.33
0	2	-15	13.38	3.49
-1	-2	-15	25.22	4.22
1	-2	15	15.26	3.44
-1	2	-15	13.79	3.57
1	2	15	20.59	3.75
2	-2	15	7.45	2.64
-2	-2	-15	4.51	2.54
2	2	15	3.79	2.39
-3	-2	-15	12.42	3.41
3	-2	15	10.23	3.08
3	2	15	5.01	2.61
-4	-2	-15	1.55	2.07
4	-2	15	0.64	1.87
4	2	15	-0.55	1.93
5	-2	15	-1.60	1.56
-5	-2	-15	-0.36	1.76
5	2	15	0.14	2.09
-5	2	-15	-2.11	1.67
6	-2	15	4.74	2.90
-6	-2	-15	2.63	2.37
6	2	15	4.10	2.75
-6	2	-15	4.11	2.69
7	-2	15	8.70	2.98
-7	-2	-15	11.55	3.54
-7	2	-15	10.10	3.38
7	2	15	8.07	3.19
8	-2	15	13.59	3.77
-8	-2	-15	21.10	4.72
8	2	15	13.41	3.93
-8	2	-15	19.37	4.47
-9	-2	-15	-1.09	1.80
9	-2	15	1.69	2.38
-9	2	-15	3.87	2.67
9	2	15	0.82	2.49
10	-2	15	-0.17	2.12

-10	-2	-15	-1.36	2.00
10	2	15	-0.10	2.77
-10	2	-15	5.24	3.13
-11	-2	-15	-0.60	2.35
11	-2	15	3.34	2.95
11	2	15	0.01	2.84
-11	2	-15	-2.63	2.03
-10	-3	15	21.56	4.89
10	3	-15	21.46	4.94
-9	-3	15	1.83	2.86
9	3	-15	1.39	2.73
-8	-3	15	32.42	5.38
8	3	-15	20.14	4.74
-7	-3	15	7.89	3.39
7	3	-15	8.90	3.59
-6	-3	15	28.20	5.03
6	3	-15	21.14	4.75
-5	-3	15	61.12	7.10
5	3	-15	53.29	6.71
-4	-3	15	0.90	2.52
4	3	-15	2.50	2.67
-4	3	15	-0.04	1.69
-3	-3	15	1.02	2.30
3	-3	-15	0.51	1.73
-3	3	15	2.19	1.85
3	3	-15	7.26	3.15
-2	-3	15	50.87	6.26
2	-3	-15	72.93	5.37
2	3	-15	58.85	6.80
1	-3	-15	-0.65	1.66
-1	-3	15	4.11	2.45
-1	3	15	4.53	2.17
1	3	-15	1.24	2.30
0	-3	-15	10.75	2.94
0	-3	15	7.63	3.00
0	3	15	8.68	2.89
0	3	-15	8.96	3.16
1	-3	15	69.44	7.04
-1	-3	-15	71.03	5.83
1	3	15	50.98	5.76
-1	3	-15	65.45	6.86
2	-3	15	1.99	2.18
-2	-3	-15	3.41	2.40
2	3	15	4.38	2.63
-2	3	-15	5.02	2.66
3	-3	15	152.24	8.94
-3	-3	-15	144.36	8.58
-3	3	-15	149.76	8.63
3	3	15	142.37	8.33
-4	-3	-15	11.80	3.56

4	-3	15	1.63	2.31
4	3	15	7.46	2.88
-4	3	-15	3.79	2.54
5	-3	15	43.94	5.87
-5	-3	-15	32.96	5.28
-5	3	-15	46.31	6.00
5	3	15	34.45	5.33
6	-3	15	122.36	8.20
-6	-3	-15	112.39	8.25
6	3	15	114.83	8.31
7	-3	15	2.32	2.53
-7	-3	-15	-0.06	2.34
7	3	15	-0.80	2.34
-8	-3	-15	29.73	5.53
8	-3	15	34.25	5.30
8	3	15	32.00	5.86
-9	-3	-15	25.62	5.52
9	-3	15	25.39	4.75
9	3	15	23.24	5.40
-7	-4	15	140.86	10.11
7	4	-15	151.60	10.29
-6	-4	15	32.07	5.69
6	4	-15	37.12	6.22
-5	-4	15	5.81	3.12
5	4	-15	7.23	3.69
-4	-4	15	89.66	8.95
4	4	-15	65.71	7.78
-3	-4	15	9.67	3.55
3	4	-15	7.19	3.59
2	-4	-15	-0.40	1.70
-2	-4	15	0.17	2.27
2	4	-15	-1.79	2.09
-2	4	15	-0.50	1.82
1	-4	-15	8.84	2.87
-1	-4	15	12.26	3.60
1	4	-15	5.35	3.16
-1	4	15	7.97	2.66
0	-4	-15	5.93	2.59
0	-4	15	-0.08	2.13
0	4	15	-0.40	2.04
0	4	-15	1.36	2.46
1	-4	15	11.39	3.57
-1	-4	-15	8.26	2.95
1	4	15	3.71	2.38
-1	4	-15	4.85	2.99
2	-4	15	7.27	3.14
-2	-4	-15	7.02	2.95
2	4	15	8.60	2.94
-2	4	-15	8.29	3.10
3	-4	15	-3.36	1.92

-3	-4	-15	-2.47	1.76
3	4	15	2.20	2.46
-3	4	-15	1.46	2.33
4	-4	15	49.51	6.46
-4	-4	-15	30.64	5.22
4	4	15	43.08	6.09
-4	4	-15	22.67	4.35
5	-4	15	17.79	4.29
-5	-4	-15	13.41	3.94
-5	4	-15	12.62	3.70
5	4	15	23.55	4.85
6	-4	15	0.67	2.36
-6	-4	-15	-0.91	2.06
6	4	15	-2.71	2.14
-6	4	-15	1.81	2.48
12	0	-16	30.62	4.80
-11	0	16	68.31	5.84
11	0	-16	77.89	6.19
10	0	-16	43.12	5.37
-10	0	16	47.59	5.55
9	0	-16	23.34	4.10
-9	0	16	22.90	4.00
-8	0	16	137.14	7.73
8	0	-16	139.74	7.81
7	0	-16	2.14	2.20
-7	0	16	0.52	1.95
6	0	-16	21.54	3.87
-6	0	16	19.43	3.68
-5	0	16	78.54	6.02
5	0	-16	70.12	5.72
4	0	-16	42.07	5.16
-4	0	16	35.58	4.72
-3	0	16	236.80	10.15
-2	0	16	68.05	5.55
-1	0	16	5.74	2.48
0	0	16	198.75	9.41
0	0	-16	205.34	9.93
1	0	16	254.11	10.87
-1	0	-16	244.42	11.01
2	0	16	69.72	6.84
-2	0	-16	66.13	6.97
-3	0	-16	129.21	8.24
3	0	16	108.55	7.37
4	0	16	14.73	3.74
-4	0	-16	15.72	4.03
-5	0	-16	15.44	3.84
5	0	16	12.76	3.69
6	0	16	-2.44	1.99
-6	0	-16	-3.43	1.75
7	0	16	167.27	9.70

-7	0	-16	155.70	9.87
8	0	16	4.54	2.95
-8	0	-16	0.26	2.16
9	0	16	91.89	9.06
-9	0	-16	80.58	8.80
-10	0	-16	62.99	7.88
10	0	16	53.91	7.07
11	0	16	50.58	7.05
-11	0	-16	40.81	6.77
-12	-1	16	8.62	3.41
12	1	-16	11.82	3.83
-11	-1	16	2.42	2.59
11	-1	-16	5.18	2.42
11	1	-16	1.51	2.41
10	-1	-16	6.39	2.80
-10	-1	16	3.05	2.48
10	1	-16	8.58	3.30
-9	-1	16	36.90	5.30
9	-1	-16	40.40	4.79
9	1	-16	44.05	5.89
8	-1	-16	11.47	2.84
-8	-1	16	5.07	2.53
-8	1	16	10.65	2.64
8	1	-16	5.19	2.98
-7	-1	16	26.75	4.51
7	-1	-16	33.64	4.50
7	1	-16	29.97	4.92
-7	1	16	26.70	3.98
6	-1	-16	9.92	2.74
-6	-1	16	16.87	3.73
6	1	-16	8.87	3.09
-6	1	16	10.81	2.79
5	-1	-16	9.93	2.89
-5	-1	16	9.56	3.10
5	1	-16	15.07	3.54
-5	1	16	10.29	2.85
-4	-1	16	1.89	2.13
-4	1	16	1.71	1.97
4	1	-16	3.96	2.50
-3	-1	16	50.29	5.85
-3	1	16	63.78	5.31
3	1	-16	56.06	6.32
-2	-1	16	19.97	3.96
2	-1	-16	10.30	2.95
2	1	-16	20.20	3.99
-2	1	16	16.68	3.44
-1	-1	16	-1.03	1.86
1	-1	-16	5.67	2.49
-1	1	16	-2.33	1.57
0	-1	-16	2.64	2.10

0	-1	16	-0.47	1.82
0	1	16	0.82	1.96
-1	-1	-16	2.07	2.21
1	-1	16	1.59	2.01
1	1	16	5.13	2.48
2	-1	16	72.82	6.14
-2	-1	-16	69.72	6.09
2	1	16	77.12	6.10
-2	1	-16	74.72	6.42
3	-1	16	7.59	2.78
-3	-1	-16	6.88	2.83
3	1	16	8.03	3.00
-3	1	-16	6.73	2.96
4	-1	16	8.61	2.95
-4	-1	-16	16.01	3.85
4	1	16	12.94	3.49
-4	1	-16	20.27	4.37
5	-1	16	2.24	2.41
-5	-1	-16	7.06	3.07
5	1	16	2.65	2.68
-5	1	-16	2.24	2.41
-6	-1	-16	15.43	4.16
6	-1	16	17.35	3.94
6	1	16	18.79	4.34
-6	1	-16	10.70	3.62
-7	-1	-16	8.11	3.30
7	-1	16	11.27	3.55
-7	1	-16	8.77	3.49
7	1	16	7.72	3.25
8	-1	16	33.19	5.42
-8	-1	-16	41.48	6.46
-8	1	-16	40.42	6.26
8	1	16	27.90	5.15
-9	-1	-16	10.38	3.78
9	-1	16	3.70	2.77
9	1	16	8.94	3.64
-9	1	-16	5.27	3.23
-10	-1	-16	-0.19	2.23
10	-1	16	0.69	2.43
10	1	16	-1.52	2.30
-10	1	-16	-2.75	2.24
-11	-1	-16	0.46	2.53
11	-1	16	-0.93	2.42
11	1	16	1.92	2.97
-11	1	-16	-2.78	2.22
-11	-2	16	14.48	4.15
11	2	-16	19.54	4.79
-10	-2	16	14.45	3.93
10	2	-16	9.95	3.66
-9	-2	16	-2.33	2.27

9	2	-16	3.48	2.86
-8	-2	16	10.96	3.55
8	-2	-16	12.52	2.95
8	2	-16	7.39	3.30
7	-2	-16	5.67	2.35
-7	-2	16	1.60	2.33
7	2	-16	4.46	3.04
-6	-2	16	25.03	4.62
6	-2	-16	37.28	4.45
6	2	-16	33.36	5.39
-6	2	16	35.42	4.17
-5	-2	16	36.37	5.33
5	2	-16	44.35	6.15
-5	2	16	37.30	4.41
-4	-2	16	1.14	2.30
-4	2	16	0.34	1.83
4	2	-16	0.78	2.27
3	-2	-16	312.24	11.38
-3	-2	16	296.95	12.60
3	2	-16	323.18	12.83
-3	2	16	326.81	11.22
2	-2	-16	59.04	6.03
-2	-2	16	47.02	5.87
2	2	-16	53.28	6.51
-2	2	16	48.47	5.18
1	-2	-16	1.24	1.89
-1	-2	16	-1.39	1.76
1	2	-16	2.57	2.49
-1	2	16	2.09	2.04
0	-2	16	139.56	8.47
0	-2	-16	128.71	7.65
0	2	-16	136.93	8.50
-1	-2	-16	2.53	2.32
1	-2	16	8.59	3.11
-1	2	-16	4.44	2.92
1	2	16	6.79	2.61
2	-2	16	35.23	5.18
-2	-2	-16	35.70	5.06
2	2	16	42.60	5.41
-3	-2	-16	-0.80	1.82
3	-2	16	0.01	1.97
3	2	16	0.72	2.07
-4	-2	-16	6.14	2.78
4	-2	16	3.96	2.44
4	2	16	-0.77	1.92
-5	-2	-16	17.92	4.01
5	-2	16	19.56	4.14
-5	2	-16	20.09	4.41
5	2	16	24.98	4.72
-6	-2	-16	-0.37	1.98

6	-2	16	2.67	2.57
-6	2	-16	-1.42	1.99
6	2	16	2.56	2.61
7	-2	16	1.81	2.31
-7	-2	-16	2.10	2.62
7	2	16	1.50	2.59
-7	2	-16	0.01	2.05
8	-2	16	2.82	2.48
-8	-2	-16	0.65	2.43
-8	2	-16	0.58	2.47
8	2	16	-0.26	2.39
-9	-2	-16	1.78	2.54
9	-2	16	-0.48	2.36
9	2	16	-1.66	2.41
-9	2	-16	1.28	2.47
10	-2	16	7.55	3.54
-10	-2	-16	5.80	3.35
-10	2	-16	-2.22	2.05
10	2	16	12.33	4.60
-9	-3	16	13.52	4.14
9	3	-16	12.37	4.34
-8	-3	16	-0.05	2.54
8	3	-16	1.13	2.83
-7	-3	16	19.27	4.53
7	3	-16	24.14	5.08
-6	-3	16	15.89	3.91
6	3	-16	11.81	4.06
-5	-3	16	-0.39	2.32
5	3	-16	2.17	2.72
-4	-3	16	20.77	4.32
4	3	-16	23.67	4.82
3	-3	-16	0.45	1.79
-3	-3	16	-0.85	2.14
3	3	-16	-1.96	1.90
-3	3	16	0.67	1.79
2	-3	-16	6.88	2.48
-2	-3	16	1.24	2.53
-2	3	16	4.77	2.30
2	3	-16	8.17	3.21
-1	-3	16	0.82	2.16
1	-3	-16	4.88	2.41
1	3	-16	2.68	2.71
0	-3	16	6.48	3.07
0	-3	-16	12.12	3.21
0	3	16	9.98	3.08
0	3	-16	4.97	2.71
1	-3	16	18.57	4.12
-1	-3	-16	9.84	3.14
-1	3	-16	13.93	3.83
1	3	16	4.92	2.68

2	-3	16	-0.52	2.01
-2	-3	-16	0.88	2.00
-2	3	-16	-0.62	2.13
2	3	16	-0.03	2.05
-3	-3	-16	-2.68	1.51
3	-3	16	-2.98	1.69
3	3	16	0.67	2.02
-3	3	-16	-1.14	2.07
-4	-3	-16	12.74	3.57
4	-3	16	5.29	2.88
4	3	16	4.27	2.83
-4	3	-16	2.08	2.64
-5	-3	-16	10.85	3.66
5	-3	16	14.42	3.76
5	3	16	19.42	4.41
-5	3	-16	17.99	3.95
6	-3	16	19.57	4.47
-6	-3	-16	29.97	5.42
6	3	16	21.98	5.03
-7	-3	-16	21.26	4.73
7	-3	16	28.82	5.15
7	3	16	34.21	5.85
-8	-3	-16	3.18	2.92
8	-3	16	11.32	3.89
8	3	16	4.73	3.30
-5	-4	16	15.27	4.62
5	4	-16	15.78	4.46
-4	-4	16	8.93	3.68
4	4	-16	6.21	3.33
-3	-4	16	8.07	3.49
3	4	-16	9.87	4.10
-2	-4	16	4.86	3.13
2	4	-16	4.02	3.05
1	-4	-16	46.55	5.57
-1	-4	16	51.08	6.83
-1	4	16	47.80	5.48
1	4	-16	41.52	6.14
0	-4	-16	81.88	6.36
0	-4	16	82.34	8.43
0	4	16	70.22	5.95
0	4	-16	70.20	7.93
1	-4	16	0.94	2.46
-1	-4	-16	-2.66	1.79
1	4	16	-0.68	2.12
-1	4	-16	2.05	2.85
-2	-4	-16	19.03	4.20
2	-4	16	14.08	4.08
2	4	16	12.66	3.57
-2	4	-16	13.40	4.10
-3	-4	-16	3.92	2.75

3	-4	16	9.74	3.60
3	4	16	5.23	3.10
-3	4	-16	10.59	3.53
4	-4	16	2.61	2.51
-4	-4	-16	-1.03	2.15
4	4	16	-2.55	2.26
-4	4	-16	1.30	2.59
11	0	-17	1.83	2.43
10	0	-17	0.35	2.22
-10	0	17	3.06	2.31
9	0	-17	-1.74	2.01
-9	0	17	-1.43	1.72
-8	0	17	0.00	1.79
8	0	-17	1.72	2.18
-7	0	17	2.10	2.02
7	0	-17	-1.55	1.81
6	0	-17	4.24	2.33
-6	0	17	-2.07	1.80
-5	0	17	-0.73	1.91
5	0	-17	-3.63	1.59
4	0	-17	2.42	2.39
-4	0	17	1.13	2.22
-3	0	17	0.17	1.90
-2	0	17	-0.76	1.79
-1	0	17	-2.12	1.75
0	0	17	0.18	1.96
0	0	-17	2.83	2.25
1	0	17	2.18	2.23
-1	0	-17	1.30	2.06
2	0	17	-1.06	1.99
-2	0	-17	-0.78	1.74
-3	0	-17	2.38	2.28
3	0	17	-1.38	1.69
4	0	17	0.01	1.97
-4	0	-17	-0.64	1.84
-5	0	-17	0.65	2.21
5	0	17	1.39	2.36
-6	0	-17	1.68	2.49
6	0	17	2.77	2.65
-7	0	-17	0.74	2.48
7	0	17	-0.65	2.35
-8	0	-17	4.97	3.08
8	0	17	-0.69	2.53
-9	0	-17	-0.26	2.49
9	0	17	1.10	2.69
10	0	17	2.47	3.14
-10	0	-17	0.02	2.49
-11	-1	17	56.77	6.62
11	-1	-17	61.02	6.04
11	1	-17	41.48	5.90

-10	-1	17	-1.06	2.23
10	-1	-17	3.14	2.62
10	1	-17	1.60	2.60
9	-1	-17	76.36	5.78
-9	-1	17	87.38	6.98
9	1	-17	94.19	7.26
8	-1	-17	32.98	4.67
-8	-1	17	26.24	4.62
-8	1	17	28.06	4.08
8	1	-17	23.47	4.60
-7	-1	17	6.40	2.88
7	-1	-17	0.86	2.16
7	1	-17	1.39	2.53
-7	1	17	1.99	2.05
-6	-1	17	17.08	3.97
6	-1	-17	14.15	3.35
-6	1	17	14.56	3.19
6	1	-17	15.53	3.83
-5	-1	17	-1.55	1.90
5	-1	-17	-0.32	2.08
-5	1	17	-1.32	1.78
5	1	-17	0.53	2.16
-4	-1	17	0.22	2.02
4	1	-17	5.13	2.75
-4	1	17	-0.53	1.91
-3	-1	17	17.67	3.85
3	1	-17	18.14	3.94
-3	1	17	17.19	3.49
2	-1	-17	1.69	1.90
-2	-1	17	-0.64	2.00
-2	1	17	1.09	2.06
2	1	-17	-5.26	1.43
-1	-1	17	23.75	4.37
1	-1	-17	26.66	4.53
-1	1	17	20.30	3.99
0	-1	17	17.31	3.89
0	-1	-17	13.71	3.55
0	1	17	12.93	3.27
-1	-1	-17	1.00	2.08
1	-1	17	6.84	2.83
1	1	17	8.26	2.88
-2	-1	-17	18.24	4.06
2	-1	17	21.88	4.21
-2	1	-17	31.14	5.14
2	1	17	32.19	4.89
3	-1	17	13.59	3.59
-3	-1	-17	2.79	2.32
3	1	17	6.51	2.89
-3	1	-17	10.59	3.52
-4	-1	-17	15.77	3.99

4	-1	17	19.33	4.18
-4	1	-17	5.18	2.81
4	1	17	14.51	3.72
5	-1	17	78.51	7.81
-5	-1	-17	66.53	7.61
5	1	17	55.56	6.77
-5	1	-17	76.18	8.35
6	-1	17	14.62	3.88
-6	-1	-17	12.41	3.75
-6	1	-17	13.16	3.86
6	1	17	8.57	3.34
7	-1	17	15.00	3.91
-7	-1	-17	3.83	3.05
7	1	17	10.72	3.72
-7	1	-17	10.30	3.71
-8	-1	-17	19.86	4.93
8	-1	17	25.07	4.66
8	1	17	35.51	6.03
-8	1	-17	31.99	5.90
-9	-1	-17	48.98	7.03
9	-1	17	64.58	7.42
-9	1	-17	52.20	7.42
9	1	17	62.08	8.15
-10	-1	-17	7.43	3.68
10	-1	17	2.49	2.83
-10	1	-17	3.52	3.34
10	1	17	2.67	3.25
-10	-2	17	11.29	3.91
10	2	-17	14.37	4.54
-9	-2	17	3.83	2.97
9	2	-17	0.82	2.58
-8	-2	17	-0.23	2.29
8	-2	-17	1.79	2.09
8	2	-17	3.69	3.21
7	-2	-17	-0.59	2.00
-7	-2	17	4.19	2.91
7	2	-17	7.70	3.37
6	-2	-17	3.40	2.32
-6	-2	17	4.68	3.06
6	2	-17	2.98	2.98
-5	-2	17	31.39	5.17
5	2	-17	30.87	5.27
-5	2	17	33.87	4.43
-4	-2	17	8.53	3.17
4	2	-17	8.71	3.38
-4	2	17	21.98	3.66
-3	-2	17	-0.55	1.98
3	2	-17	4.12	2.69
-3	2	17	1.92	2.02
-2	-2	17	7.22	3.19

2	-2	-17	7.54	2.99
-2	2	17	4.31	2.48
2	2	-17	8.02	3.25
-1	-2	17	1.63	2.40
1	-2	-17	0.36	1.93
1	2	-17	3.81	2.72
-1	2	17	-0.59	1.86
0	-2	-17	8.83	2.98
0	-2	17	11.86	3.52
0	2	17	7.22	2.90
0	2	-17	10.32	3.44
1	-2	17	5.95	2.98
-1	-2	-17	7.23	2.89
1	2	17	8.74	3.19
-1	2	-17	9.30	3.27
2	-2	17	0.60	2.27
-2	-2	-17	0.87	2.24
2	2	17	0.64	2.09
-3	-2	-17	15.82	3.86
3	-2	17	11.11	3.52
3	2	17	15.77	3.90
4	-2	17	69.32	7.13
-4	-2	-17	76.15	8.04
4	2	17	70.31	7.65
-5	-2	-17	5.23	2.91
5	-2	17	3.48	2.60
5	2	17	2.46	2.57
6	-2	17	6.11	3.03
-6	-2	-17	1.36	2.61
6	2	17	4.33	2.86
-6	2	-17	2.85	2.84
7	-2	17	5.17	3.03
-7	-2	-17	3.15	2.71
-7	2	-17	2.54	2.65
7	2	17	-1.58	2.31
8	-2	17	3.43	2.69
-8	-2	-17	1.41	2.54
8	2	17	-3.74	2.39
-8	2	-17	-2.04	2.04
9	-2	17	4.03	2.88
-9	-2	-17	6.61	3.42
-9	2	-17	-2.41	2.06
9	2	17	4.23	3.46
-7	-3	17	0.34	2.41
7	3	-17	-1.45	2.43
-6	-3	17	26.88	5.18
6	3	-17	30.03	5.77
-5	-3	17	7.69	3.63
5	3	-17	9.42	3.70
-4	-3	17	2.64	2.76

4	3	-17	-0.17	2.26
-3	-3	17	21.88	4.64
3	-3	-17	40.53	5.09
3	3	-17	23.40	4.96
2	-3	-17	8.59	2.88
-2	-3	17	6.23	3.12
2	3	-17	6.02	3.33
-2	3	17	5.84	2.56
-1	-3	17	37.83	5.90
1	-3	-17	35.88	5.14
1	3	-17	25.16	5.25
0	-3	17	7.63	3.11
0	-3	-17	15.70	3.93
0	3	17	10.20	3.34
0	3	-17	11.85	3.69
-1	-3	-17	0.43	2.03
1	-3	17	0.72	2.42
1	3	17	-0.36	2.15
-1	3	-17	-2.90	1.69
2	-3	17	11.16	3.61
-2	-3	-17	7.24	2.89
-2	3	-17	17.94	4.47
2	3	17	16.52	4.11
-3	-3	-17	13.11	3.68
3	-3	17	9.40	3.40
3	3	17	10.77	3.42
-3	3	-17	9.05	3.52
4	-3	17	-0.36	2.13
-4	-3	-17	6.36	2.94
-4	3	-17	1.83	2.43
4	3	17	-0.50	2.16
-5	-3	-17	6.34	2.95
5	-3	17	10.76	3.67
-5	3	-17	6.06	3.34
5	3	17	7.96	3.43
-6	-3	-17	20.87	5.00
6	-3	17	15.13	4.00
-6	3	-17	13.94	4.21
6	3	17	14.23	4.44
-7	-3	-17	1.93	2.73
7	-3	17	-0.23	2.40
7	3	17	2.74	3.19
-2	-4	17	26.33	5.35
2	4	-17	39.63	6.38
-1	-4	17	23.16	5.04
1	-4	-17	32.25	4.96
1	4	-17	46.17	6.79
0	-4	-17	22.14	4.27
0	-4	17	8.68	3.47
0	4	17	14.91	3.69

0	4	-17	15.02	4.34
1	-4	17	40.06	6.19
-1	-4	-17	37.90	5.44
-1	4	-17	50.79	7.14
1	4	17	47.26	6.01
10	0	-18	8.42	3.26
9	0	-18	1.04	2.37
-9	0	18	1.58	2.44
-8	0	18	-1.74	2.02
8	0	-18	1.04	2.24
-7	0	18	25.35	4.34
7	0	-18	33.51	5.14
-6	0	18	51.15	6.02
6	0	-18	50.41	5.97
5	0	-18	-0.95	2.09
-5	0	18	1.27	2.09
4	0	-18	25.50	4.53
-4	0	18	32.58	4.85
3	0	-18	223.88	10.54
-3	0	18	236.01	10.78
-2	0	18	1.49	2.17
-1	0	18	159.37	8.96
0	0	-18	38.33	5.44
0	0	18	58.47	6.78
1	0	18	61.19	6.77
-1	0	-18	44.48	5.98
-2	0	-18	44.81	6.16
2	0	18	67.41	7.11
-3	0	-18	28.39	5.22
3	0	18	25.67	4.80
4	0	18	26.38	4.80
-4	0	-18	19.41	4.58
5	0	18	74.42	7.85
-5	0	-18	63.10	7.47
-6	0	-18	29.70	5.71
6	0	18	31.60	5.62
-7	0	-18	3.89	3.06
7	0	18	-0.50	2.66
-8	0	-18	327.51	15.45
8	0	18	317.16	14.55
9	0	18	4.52	3.30
-9	0	-18	0.25	2.81
10	-1	-18	5.07	2.83
-10	-1	18	4.75	3.08
10	1	-18	5.94	3.33
9	-1	-18	2.90	2.59
-9	-1	18	3.31	2.71
9	1	-18	1.45	2.90
8	-1	-18	-1.69	2.15
-8	-1	18	1.06	2.39

8	1	-18	0.50	2.36
7	-1	-18	-0.17	2.14
-7	-1	18	-0.17	2.16
-7	1	18	-0.73	2.18
7	1	-18	2.17	2.67
-6	-1	18	12.33	3.62
6	-1	-18	13.38	3.29
-6	1	18	11.85	3.18
6	1	-18	20.44	4.41
5	-1	-18	34.87	4.93
-5	-1	18	27.53	5.05
5	1	-18	31.23	5.19
-5	1	18	31.93	4.62
-4	-1	18	30.60	5.08
-4	1	18	42.99	5.32
4	1	-18	32.93	5.31
-3	-1	18	4.48	2.80
-3	1	18	8.24	2.91
3	1	-18	0.23	2.36
-2	-1	18	91.00	7.22
-2	1	18	99.33	7.01
2	1	-18	106.45	7.79
-1	-1	18	5.23	2.77
1	-1	-18	4.46	2.59
-1	1	18	6.94	3.09
1	1	-18	9.19	3.44
0	-1	18	-1.31	1.83
0	-1	-18	3.14	2.39
0	1	18	-0.53	1.92
1	-1	18	-2.36	1.92
-1	-1	-18	0.96	2.01
1	1	18	-2.34	1.82
2	-1	18	21.26	4.34
-2	-1	-18	14.27	3.82
2	1	18	16.29	3.94
-3	-1	-18	7.46	3.02
3	-1	18	2.70	2.60
-3	1	-18	2.14	2.24
3	1	18	3.86	2.71
4	-1	18	-2.20	1.91
-4	-1	-18	-2.03	1.89
4	1	18	1.24	2.41
-4	1	-18	-1.43	1.95
5	-1	18	-0.26	2.32
-5	-1	-18	-1.36	2.04
-5	1	-18	-1.10	2.31
5	1	18	-0.81	2.24
-6	-1	-18	8.23	3.39
6	-1	18	15.84	4.08
-6	1	-18	12.14	3.98

6	1	18	10.06	3.81
-7	-1	-18	31.07	5.79
7	-1	18	27.22	5.27
-7	1	-18	36.87	6.22
7	1	18	28.69	5.48
8	-1	18	16.39	4.25
-8	-1	-18	14.25	4.55
8	1	18	12.05	4.22
-8	1	-18	16.02	4.63
-9	-1	-18	22.20	5.32
9	-1	18	17.39	4.63
-9	1	-18	18.74	4.94
9	1	18	30.11	6.16
8	-2	-18	8.16	2.85
-8	-2	18	10.55	3.87
8	2	-18	4.78	3.33
-7	-2	18	4.47	3.07
7	-2	-18	7.90	2.88
7	2	-18	13.43	4.36
6	-2	-18	8.71	3.02
-6	-2	18	17.59	4.33
6	2	-18	4.84	3.29
-5	-2	18	14.68	4.05
5	2	-18	5.27	3.03
-5	2	18	6.86	2.54
-4	-2	18	3.08	2.65
4	2	-18	2.44	2.66
-4	2	18	1.10	2.10
-3	-2	18	-0.40	2.00
-3	2	18	-0.04	2.11
3	2	-18	4.00	2.87
2	-2	-18	8.00	2.97
-2	-2	18	6.86	3.15
2	2	-18	2.94	2.73
-2	2	18	3.41	2.34
1	-2	-18	26.71	4.70
-1	-2	18	19.63	4.14
-1	2	18	16.40	3.68
1	2	-18	15.72	3.83
0	-2	18	14.39	3.82
0	-2	-18	13.41	3.72
0	2	18	6.97	3.05
0	2	-18	6.74	3.42
-1	-2	-18	-2.00	1.75
1	-2	18	-1.75	2.01
-1	2	-18	-1.89	2.16
2	-2	18	67.99	7.50
-2	-2	-18	54.75	6.78
2	2	18	53.28	6.45
3	-2	18	57.23	6.85

-3	-2	-18	72.48	7.88
3	2	18	62.19	7.15
4	-2	18	14.96	3.87
-4	-2	-18	11.08	3.72
4	2	18	10.43	3.80
-5	-2	-18	22.63	4.91
5	-2	18	19.13	4.53
5	2	18	16.93	4.53
6	-2	18	4.56	2.89
-6	-2	-18	1.55	2.82
6	2	18	8.24	3.84
-6	2	-18	5.34	3.18
-7	-2	-18	27.25	5.34
7	-2	18	26.90	5.28
-7	2	-18	24.05	5.05
7	2	18	32.96	6.04
-6	-3	18	-1.63	2.58
6	3	-18	-0.46	2.76
-5	-3	18	9.73	3.71
5	3	-18	15.64	4.38
-4	-3	18	21.92	4.95
4	3	-18	17.12	4.77
3	-3	-18	-1.92	1.78
-3	-3	18	-2.16	2.21
3	3	-18	0.71	2.55
2	-3	-18	42.18	5.58
-2	-3	18	22.88	4.74
-2	3	18	37.24	5.00
1	-3	-18	13.42	3.55
-1	-3	18	0.34	2.79
1	3	-18	12.00	3.95
-1	3	18	11.65	3.32
0	-3	18	3.19	2.85
0	-3	-18	16.47	3.99
0	3	-18	6.38	3.43
-1	-3	-18	2.33	2.46
1	-3	18	-0.09	2.22
1	3	18	1.35	2.43
-1	3	-18	-1.13	2.40
2	-3	18	2.79	2.86
-2	-3	-18	2.23	2.62
-2	3	-18	1.88	2.50
2	3	18	3.58	2.77
-3	-3	-18	5.16	3.01
3	-3	18	3.19	2.77
-3	3	-18	7.89	3.27
3	3	18	13.16	4.04
4	-3	18	0.58	2.52
-4	-3	-18	4.29	2.78
-4	3	-18	-1.94	2.03

4	3	18	4.38	3.06
5	-3	18	0.48	2.39
-5	-3	-18	1.71	2.50
-5	3	-18	-1.81	2.46
5	3	18	3.26	3.05
9	0	-19	-2.65	1.99
-9	0	19	2.82	2.51
8	0	-19	0.95	2.31
-8	0	19	1.71	2.48
7	0	-19	0.04	2.43
-7	0	19	1.74	2.33
-6	0	19	-1.60	2.02
6	0	-19	3.89	2.80
-5	0	19	-1.71	2.05
5	0	-19	-2.18	2.05
-4	0	19	0.80	2.42
4	0	-19	1.43	2.60
-3	0	19	1.96	2.57
3	0	-19	-1.10	2.04
-2	0	19	-1.68	1.99
-1	0	19	-0.53	2.07
0	0	19	1.62	2.38
-1	0	-19	1.37	2.19
1	0	19	0.29	2.37
-2	0	-19	2.27	2.24
2	0	19	2.04	2.52
3	0	19	-0.63	2.18
-3	0	-19	0.25	2.16
-4	0	-19	-2.36	1.76
4	0	19	1.62	2.53
5	0	19	2.03	2.61
-5	0	-19	-0.86	2.27
6	0	19	-1.40	2.39
-6	0	-19	-2.07	2.03
-7	0	-19	1.34	2.73
7	0	19	0.42	2.67
8	0	19	2.76	3.12
-8	0	-19	0.82	2.57
8	-1	-19	-3.94	2.17
-8	-1	19	-1.57	2.27
8	1	-19	-1.92	2.38
7	-1	-19	30.23	4.63
-7	-1	19	17.23	4.20
7	1	-19	22.58	5.02
-7	1	19	27.86	4.54
-6	-1	19	14.47	4.10
6	-1	-19	15.76	3.69
-6	1	19	8.19	2.93
6	1	-19	13.02	3.92
5	-1	-19	13.90	3.70

-5	-1	19	20.49	4.52
5	1	-19	16.75	4.44
-5	1	19	15.22	3.60
4	-1	-19	115.68	7.55
-4	-1	19	132.32	8.82
4	1	-19	126.26	8.53
-4	1	19	128.58	8.07
-3	-1	19	51.57	6.57
-3	1	19	53.60	6.26
3	1	-19	62.13	7.15
-2	-1	19	55.67	6.79
-2	1	19	60.50	6.71
2	1	-19	53.38	6.87
-1	-1	19	77.81	7.92
1	-1	-19	71.21	7.55
-1	1	19	66.38	6.89
1	1	-19	68.10	7.66
0	-1	19	47.29	6.22
0	-1	-19	39.51	5.76
0	1	19	34.04	5.27
1	-1	19	-1.87	2.07
-1	-1	-19	3.74	2.67
1	1	19	0.47	2.27
-2	-1	-19	35.07	5.68
2	-1	19	39.54	6.06
2	1	19	31.30	5.25
3	-1	19	-4.04	1.80
-3	-1	-19	2.02	2.48
3	1	19	2.13	2.68
-3	1	-19	1.24	2.45
-4	-1	-19	4.32	2.96
4	-1	19	1.12	2.69
-4	1	-19	-2.04	2.21
4	1	19	-0.13	2.58
5	-1	19	3.50	2.99
-5	-1	-19	1.47	2.72
-5	1	-19	6.22	3.49
5	1	19	0.04	2.83
6	-1	19	-4.38	2.14
-6	-1	-19	4.76	3.12
6	1	19	-1.86	2.44
-6	1	-19	-0.49	2.70
7	-1	19	8.77	3.70
-7	-1	-19	1.39	2.92
-7	1	-19	8.20	3.79
7	1	19	9.82	4.15
-7	-2	19	10.56	3.89
7	-2	-19	-0.26	2.37
7	2	-19	15.72	4.47
6	-2	-19	1.57	2.67

-6	-2	19	4.37	3.03
6	2	-19	-0.89	2.32
-5	-2	19	15.29	4.05
5	-2	-19	16.82	3.93
5	2	-19	18.97	4.63
-4	-2	19	23.35	4.79
-4	2	19	31.36	4.56
4	2	-19	19.84	4.77
-3	-2	19	4.18	2.79
3	2	-19	11.54	3.89
-3	2	19	7.91	2.99
-2	-2	19	2.86	2.66
2	-2	-19	2.62	2.50
2	2	-19	0.15	2.64
-2	2	19	1.09	2.26
1	-2	-19	1.32	2.23
-1	-2	19	0.18	2.38
1	2	-19	-1.38	2.44
-1	2	19	-0.55	2.19
0	-2	19	4.05	2.99
0	-2	-19	-0.63	2.27
0	2	-19	0.76	2.69
0	2	19	-2.46	2.01
-1	-2	-19	10.47	3.60
1	-2	19	11.58	3.90
-1	2	-19	4.22	3.15
1	2	19	7.62	3.31
-2	-2	-19	-2.74	2.05
2	-2	19	-2.19	2.37
-2	2	-19	-0.09	2.43
-3	-2	-19	-0.69	2.17
3	-2	19	-0.63	2.35
3	2	19	-1.93	2.02
-4	-2	-19	-3.13	1.79
4	-2	19	1.66	2.41
4	2	19	-5.56	1.81
5	-2	19	0.19	2.66
-5	-2	-19	2.31	2.65
5	2	19	0.50	2.76
6	-2	19	6.14	3.25
-6	-2	-19	-0.69	2.29
6	2	19	2.98	3.02
3	3	-19	-1.75	2.30
2	-3	-19	14.99	3.88
2	3	-19	19.80	4.80
-1	-3	19	16.00	4.49
1	-3	-19	19.11	4.23
-1	3	19	25.65	4.65
1	3	-19	21.05	5.02
0	-3	19	1.54	2.77

0	-3	-19	-1.49	2.27
0	3	-19	2.99	3.13
0	3	19	1.46	2.50
-1	-3	-19	10.72	3.52
1	-3	19	15.97	4.35
-1	3	-19	16.50	4.43
2	-3	19	7.45	3.38
-2	-3	-19	11.03	3.47
-2	3	-19	9.42	3.71
-7	0	20	49.49	6.25
7	0	-20	46.81	6.32
6	0	-20	10.21	3.74
-6	0	20	15.34	4.06
5	0	-20	3.46	2.79
-5	0	20	-0.49	2.26
4	0	-20	0.13	2.61
-4	0	20	-0.83	2.36
3	0	-20	81.89	8.24
-3	0	20	66.55	7.38
-2	0	20	10.94	3.85
-1	0	20	4.55	3.00
0	0	20	1.08	2.48
-1	0	-20	17.04	4.26
1	0	20	16.13	4.33
2	0	20	26.69	5.08
-2	0	-20	28.17	5.37
-3	0	-20	4.36	2.89
3	0	20	9.59	3.75
4	0	20	40.14	6.19
5	0	20	4.23	3.22
-5	0	-20	0.56	2.44
6	0	20	33.80	6.14
-6	0	-20	24.21	5.47
6	-1	-20	2.88	2.43
-6	-1	20	-1.70	2.44
-6	1	20	-0.22	2.31
6	1	-20	-1.00	2.59
5	-1	-20	0.90	2.42
-5	-1	20	-0.45	2.56
-5	1	20	2.89	2.55
5	1	-20	2.06	2.68
4	-1	-20	15.43	3.89
-4	-1	20	6.44	3.45
4	1	-20	2.88	3.28
-4	1	20	11.67	3.55
-3	-1	20	18.26	4.51
-3	1	20	13.17	3.72
3	1	-20	12.37	4.28
-2	-1	20	1.84	2.54
2	1	-20	5.95	3.23

-2	1	20	0.46	2.29
-1	-1	20	2.34	2.82
1	1	-20	-2.99	2.52
-1	1	20	1.63	2.42
0	-1	20	0.39	2.37
0	-1	-20	2.50	2.64
0	1	20	-0.69	2.51
1	-1	20	8.17	3.55
-1	-1	-20	1.48	2.46
1	1	20	7.70	3.40
2	-1	20	-0.22	2.41
-2	-1	-20	3.91	3.07
2	1	20	2.62	2.81
3	-1	20	23.15	5.10
-3	-1	-20	38.81	6.28
3	1	20	34.22	5.78
-4	-1	-20	8.46	3.49
4	-1	20	6.95	3.56
4	1	20	4.44	3.20
-4	1	-20	8.03	3.70
-5	-1	-20	2.54	3.16
5	-1	20	3.77	3.16
-5	1	-20	7.44	3.92
5	1	20	4.69	3.27
-4	-2	20	9.24	3.75
4	2	-20	12.65	4.01
-3	-2	20	-3.99	2.15
3	2	-20	0.11	2.58
-3	2	20	0.06	2.33
-2	-2	20	6.94	3.48
2	2	-20	8.17	3.68
-2	2	20	11.60	3.64
1	-2	-20	6.75	3.08
-1	-2	20	3.67	3.00
-1	2	20	1.25	2.71
1	2	-20	2.84	2.81
0	-2	-20	-0.36	2.28
0	-2	20	4.06	3.08
0	2	20	-0.64	2.27
0	2	-20	-0.54	2.99
1	-2	20	2.86	2.98
-1	-2	-20	0.37	2.45
1	2	20	5.38	3.18
-1	2	-20	4.44	3.26
-2	-2	-20	-3.45	1.71
2	-2	20	2.20	3.06
-2	2	-20	2.71	3.34
2	2	20	1.50	3.13
3	-2	20	0.43	2.71
-3	-2	-20	-2.07	1.86

```
  4   0 -21    1.75    2.80
 -4   0  21    1.08    2.73
  3   0 -21    4.75    3.23
 -3   0  21    0.55    2.59
 -2   0  21    4.40    3.21
  2   0 -21    5.22    3.14
 -1   0  21   -1.35    2.56
  0   0  21    2.71    2.67
  1   0  21   -1.07    2.32
  2   0  21   -2.91    2.16
 -2   0 -21   -0.73    2.21
  3   0  21   -0.53    2.61
 -3   0 -21    2.02    2.86
 -3  -1  21    3.52    3.03
  3   1 -21    6.76    3.21
 -3   1  21    1.51    2.82
 -2  -1  21    4.92    3.19
 -2   1  21    1.98    2.90
  2   1 -21    5.99    3.39
 -1  -1  21    2.74    3.11
  1   1 -21    2.21    3.29
 -1   1  21    1.09    2.73
  0  -1 -21   13.27    4.21
  0  -1  21   19.74    4.74
  0   1  21    9.77    3.97
  0   1 -21   18.37    4.78
 -1  -1 -21   15.30    4.23
  1  -1  21   23.97    5.15
  1   1  21   18.57    4.67
  2  -1  21    3.47    3.15
 -2  -1 -21    1.43    2.87
  2   1  21    7.24    3.57
  0   0   0    0.00    0.00
```

_computing_structure_solution   'SHELXT 2018/2 (Sheldrick, 2018)'
;

_shelx_hkl_checksum   34619

.... End of .HKL file ....
#--#

#--#
#===END OF CIF FILE FOR MH1X3   #
#--#
#   #
##
#####
_chemical_name_common   EDTA+.ClO4-
