# Supplementary material for: Synthesis, Crystal Structure, Hirshfeld Surface, Vibrational Thermal Analyses, and NLO Properties of a New Hybrid Material: 2,4-Diamino-6-Phenyl-1,3,5-Triazinium Perchlorate, Supported by DFT Calculations
Source: Molecules. 2026 Sep 22;31(19):3369. doi: 10.3390/molecules31193369 (PMC13665900; doi:10.3390/molecules31193369)
Supplement: Supplementary file 1 [file molecules-31-03369-s001.zip › Supplementary Tables.pdf]

Table S1. Selected characteristic geometric parameters (Å, °).

**Distances**			
C1—N4	1.309 (3)	C4—C9	1.385 (3)
C1—N2	1.327 (3)	C6—C5	1.380 (3)
C1—N1	1.365 (2)	C6—C7	1.362 (4)
C2—N5	1.313 (3)	C8—C7	1.369 (4)
C2—N2	1.347 (3)	C9—C8	1.385 (3)
C2—N3	1.366 (2)	Cl1—O1	1.4309 (17)
C3—N3	1.303 (2)	Cl1—O2	1.4154 (18)
C3—N1	1.356 (2)	Cl1—O4	1.4261 (15)
C4—C3	1.479 (3)	Cl1—O3	1.4331 (18)
C4—C5	1.385 (3)		
**Angles**			
N4—C1—N2	120.73 (18)	N3—C3—C4	119.59 (17)
N1—C1—N4	118.49 (19)	N1—C3—C4	119.22 (17)
N1—C1—N2	120.76 (17)	C5—C4—C9	118.7 (2)
N5—C2—N2	118.35 (17)	C8—C9—C4	120.0 (2)
N5—C2—N3	116.42 (18)	C3—N1—C1	120.19 (17)
N2—C2—N3	125.23 (17)	C1—N2—C2	115.93 (15)
N3—C3—N1	121.19 (17)	C2—N3—C3	116.54 (16)
C5—C4—C3	118.11 (19)	O2—Cl1—O1	108.66 (12)
C9—C4—C3	123.21 (19)	O2—Cl1—O4	110.18 (11)
C6—C5—C4	120.4 (2)	O2—Cl1—O3	110.27 (14)
C7—C6—C5	120.7 (2)	O4—Cl1—O1	110.37 (11)
C6—C7—C8	119.6 (2)	O4—Cl1—O3	108.80 (11)
C7—C8—C9	120.7 (2)	O1—Cl1—O3	108.54 (13)
**Torsions**			
N1—C3—C4—C5	174.53 (19)	N3—C3—N1—C1	−1.6 (3)
N3—C3—C4—C5	−5.4 (3)	C4—C3—N1—C1	178.54 (17)
N3—C3—C4—C9	173.9 (2)	N4—C1—N1—C3	179.98 (18)
N2—C1—N1—C3	−1.7 (3)	N1—C3—C4—C9	−6.1 (3)
C9—C4—C5—C6	1.4 (4)	N4—C1—N2—C2	−177.39 (19)
C3—C4—C5—C6	−179.3 (2)	N1—C1—N2—C2	4.3 (3)
C5—C6—C7—C8	0.5 (4)	N5—C2—N2—C1	174.78 (18)
C4—C5—C6—C7	−1.2 (4)	N3—C2—N2—C1	−4.3 (3)
C6—C7—C8—C9	0.0 (5)	N1—C3—N3—C2	1.8 (3)
C7—C8—C9—C4	0.2 (5)	C4—C3—N3—C2	−178.33 (16)
C3—C4—C9—C8	179.8 (2)	N5—C2—N3—C3	−177.84 (18)
C5—C4—C9—C8	−0.8 (4)	N2—C2—N3—C3	1.2 (3)

Table S2. Geometric properties of the hydrogen bonds (Å, °).

D—*H*···*A*	*D*—H (Å)	H···A (Å)	*D*···*A* (Å)	*D*—H···*A* (°)
N1—H1N···O3	0.751	2.189	2.923	165.46
N4—H4O···O1	0.828	2.270	3.051	157.40
N4—H4O···O3	0.828	2.725	2.752	135.17
N4—H4N···O1	0.817	2.703	3.248	125.66
N4—H4N···O4	0.817	2.407	3.114	145.47
N4—H4N···N5	0.817	2.892	3.479	130.56
N5—H5N···N2	0.804	2.295	3.091	170.56
N5—H5N···N4	0.804	2.949	3.479	125.57
N5—H5O···O2	0.790	2.325	3.088	162.49
C6—H6···O2	0.950	2.632	3.317	129.39
C9—H9···O3	0.950	2.417	3.352	167.87

https://doi.org/10.3390/xxxxx
